# Cervical cancer-produced neuromedin-B reprograms Schwann cells to initiate perineural invasion

**DOI:** 10.1038/s41419-024-07030-9

**Published:** 2024-08-30

**Authors:** Xiaoyan Gao, Qi Wang, Ting Huang, Chen Xu, Xiaoming Yang, Lin Zhang, Jing Wang, Linlin Yang, Xuan Zheng, Qiong Fan, Dan Cao, Lijuan Li, Ting Ni, Xiao Sun, Jin Hou, Yudong Wang

**Affiliations:** 1 Department of Gynecologic Oncology, the International Peace Maternity and Child Health Hospital, School of Medicine, Shanghai Jiao Tong University, Shanghai, China; 2 National Key Laboratory of Immunity and Inflammation, Institute of Immunology, Naval Medical University, Shanghai, China; 3 Shanghai Municipal Key Clinical Specialty, Female Tumor Reproductive Specialty, Shanghai, China

**Keywords:** Cancer microenvironment, Tumour biomarkers, Cervical cancer

## Abstract

Perineural invasion (PNI) is a new approach of cervical cancer invasion and metastasis, involving the cross-talk between tumor and nerve. However, the initiating signals and cellular interaction mechanisms of PNI remain largely elusive. The nerve-sparing radical hysterectomy (NSRH) proposed to improve postoperative quality of life is only applicable to cervical cancer patients without PNI. Therefore, it is important to elucidate the underlying mechanisms initiating PNI, and suggest the effective biomarkers to predict PNI before NSRH surgery. Here, we found that PNI is the characteristic of advanced cervical cancer, and Schwann cells were the antecedent cells that initiating PNI. Further, neuropeptide neuromedin B (NMB) produced by cervical cancer cells was determined to induce PNI by reprogramming Schwann cells, including driving their morphological and transcriptional changes, promoting their proliferation and migration, and initiating PNI by secreting CCL2 and directing axon regeneration. Mechanistically, cervical cancer cells-produced NMB activated its receptor NMBR in Schwann cells, and opened the T-type calcium channels to stimulate Ca^2+^ influx through PKA signaling, which could be blocked by the inhibitor. Clinically, combined examination of serum NMB and CCL2 levels was suggested to effectively predict PNI in cervical cancer patients. Our data demonstrate that cervical cancer-produced NMB initiates the reprograming of Schwann cells, which then direct axon regeneration, thus causing PNI onset. The elevated serum NMB and CCL2 levels may be useful for the decision-making to nerve sparing during hysterectomy surgery of cervical cancer patients.

## Introduction

Cervical cancer is one of the top five common cancers and cancer-related deaths worldwide in women. The operation of radical hysterectomy (RH) is preferred in the treatment of early-stage cervical cancer, but the resected nerve tissues may cause pelvic autonomic dysfunction, including lower urinary tract dysfunction and sexual dysfunction [1]. Thus, nerve-sparing radical hysterectomy (NSRH) has been used for the early cervical cancer patients, to compensate for the impact of traditional RH surgery on the quality of life [2]. However, perineural invasion (PNI), an important mechanism of tumor invasion and metastasis, is the key characteristic of advanced cervical cancer and is involved in some of the clinical symptoms such as pelvic pain [3]. Obviously, advanced cervical cancer with PNI is not suitable for NSRH, and the mechanisms for the initiation of PNI and its preoperative diagnosis are still the important scientific and clinical problems that require further investigation [4].

In the clinics, the preoperative diagnosis of PNI is of prior importance for the option of NSRH or RH for cervical cancer patients. The conventional approaches for the diagnosis of PNI mainly depends on pathological examination during and post-surgery, while the reliable method to identify PNI before surgery is still lacking [4]. Recent studies showed that preoperative CT or MRI imaging may be beneficial for diagnosing PNI before surgery of oral squamous cell carcinoma and prostate cancer [5], rectal cancer [6], and colorectal cancer [7], but it is not an ideal method for head and neck cancer patients [8]. However, there is little evidence showing the effectiveness of imaging techniques in the preoperative identification of PNI in cervical cancer patients. Only one patient with cervical cancer who was diagnosed with PNI by PET/CT imaging has been reported [9]. Hence, there is still a lack of applicable biomarkers for preoperative evaluation of PNI to determine whether cervical cancer patients could choose NSRH before surgery.

PNI is mainly manifested as tumor infiltration and metastasis along the nerve [10]. Axon regeneration and subsequent tumor directional migration and invasion along the nerve are the key steps during PNI. This process depends on the dynamic cross-talk between nerve and tumor, involving complex molecular and cellular mechanisms [11]. Among these, Schwann cells play an important role in PNI and are activated and reprogrammed by tumor invasion [12]. In addition to secreting neurotrophic factors and guiding axon outgrowth, activated Schwann cells directly contact tumor cells, which may initiate PNI [13]. The underlying mechanisms for the cross-talk between Schwann cells and tumor during PNI in cervical cancer are not well understood [12]. We previously reported that Schwann cell-derived CCL2 can promote the migration of tumor cells along the regenerated neurites, thereby forming PNI in cervical cancer [14]. However, it remains unknown how Schwann cells are activated, and whether they are activated by tumor cells to initiate PNI in cervical cancer.

In order to elucidate whether Schwann cells were activated by cervical cancer cells and then initiate PNI, together with the underlying mechanisms, we screened the expression of genes in cervical cancer cells in the co-culture system with nerve, and found the tumor-derived neuropeptide, neuromedin B (NMB), in the activation of Schwann cells, induction of axon regeneration, and initiation of PNI. The function and mechanism of cervical cancer-produced NMB in the initiation and progression of PNI were then determined both in vitro and in vivo, as well as in cervical cancer patients, suggesting its potential in the prediction of PNI and the targeted therapy.

## Results

### PNI is the characteristic of advanced cervical cancer

**Fig. 1:**
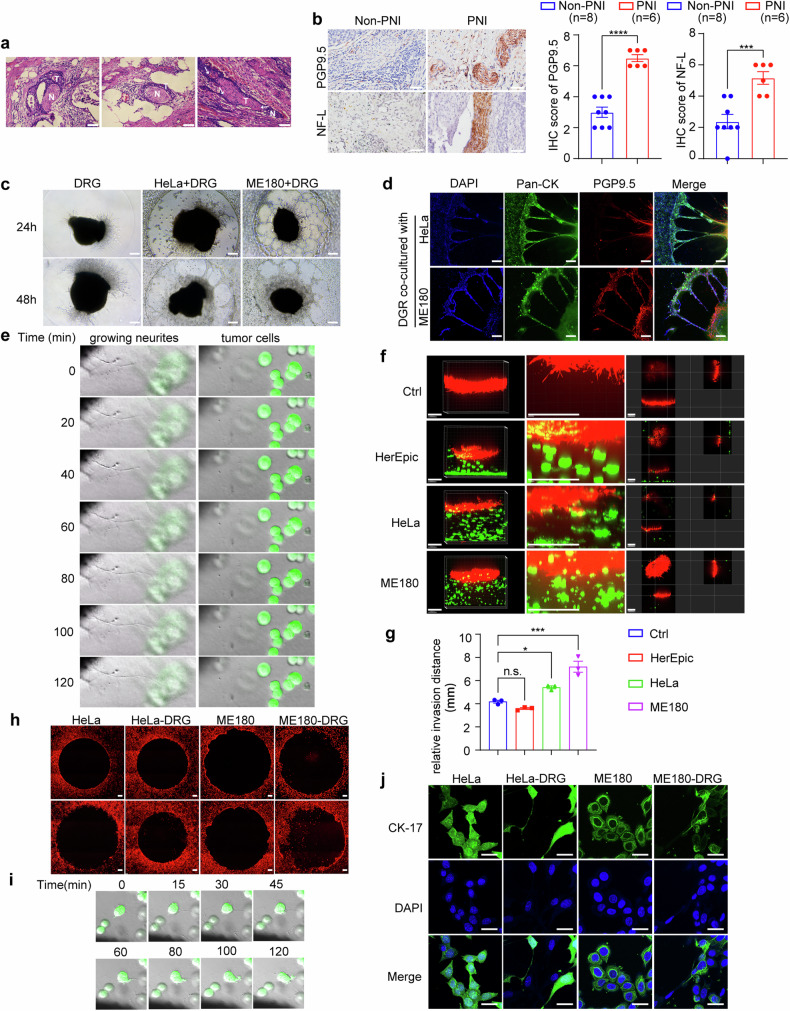
Cervical cancer promotes neurogenesis of axons through activating Schwann cells. **a** HE staining was analyzed in PNI-positive tumor tissues of cervical cancer patients. 200×magnification, scale bars: 50 μm; N, nerves, T, tumors; white arrows indicate areas of perineural invasion by cervical cancer cells. **b** Neurogenesis was analyzed by PGP9.5 and Neurofilament L (NF-L) staining. 400×magnification, scale bars: 50 μm. Relative IHC score was analyzed using unpaired *t*-test. **c** Representative brightfield images were shown as indicated in HeLa and ME180 cells induced PNI in an in vitro model. DRG was placed in the center of Matrigel. ×50 magnification, scale bars: 200 μm. **d** Double immunofluorescence staining of neurites (PGP9.5, red) and cervical cancer cells (Pan-CK, green) in the PNI model was analyzed. 100×magnification, upper: HeLa, scale bars: 100 μm; lower: ME180, scale bars: 100 μm. **e** Schwann cells are the antecedent cells of PNI in cervical cancer. Living cell images show nerve innervation in the in vitro PNI models with HeLa cells (Zsgreen, green) and DRG. The left column shows Schwann cells demyelinating from DRG and moving toward the tumor cell clusters, while the right column shows the latter remains stationary. Times are shown as minutes (see Supplementary Movie 1, 2). **f** Immunofluorescent staining was analyzed for the movement of RSC96 cells in a 3D PNI model. Red: mScarlet labeled RSC96 cells. Green: Zsgreen labeled HcerEpic, HeLa and ME180 cells. Scale bars: 80,000 μm. **g** Relative invasion distance of RSC96 cells in (**f**) was analyzed (n = 3). **h** Immunofluorescent staining of CM-Dil was analyzed in Hela and ME180 cells (red) co-cultured with DRG for 1 or 4 days. Top row: day 1; bottom row: day 4. ×100 magnification, scale bars: 200 μm. **i** Live-cell imaging was analyzed in the crosstalk between Schwann cells and tumor cells (Zsgreen, green). Tumor cells formed membrane protrusion and moved toward DRGs at a contact point with Schwann cells. Times are shown as minutes (see Supplemental Movie 3). **j** Immunofluorescent staining of CK-17 was analyzed in HeLa and ME180 co-cultured with or without DRGs. ×1000 magnification, scale bars: 20 μm. Data are shown as photographs from one representative of three independent experiments. *P < 0.05, **P < 0.01, *** *P* < 0.001, **** *P* < 0.0001.

We first examined the PNI phenomenon in tumor tissues of cervical cancer patients, and observed three patterns, including crescent-shaped incomplete encirclement, complete encirclement, and neural permeation (intraneural invasion by tumor cells) (Fig. 1a). The tumor tissues and clinical data from 423 cervical cancer patients in IPMCH from 2017 to 2021 were collected, and 16.5% (70/423) of them were diagnosed with PNI according to the HE staining (Table S1, Supporting Information). We found a positive correlation between PNI and advanced clinical stage (*p* = 0.003), larger tumor size (*p* = 0.001), deeper invasion (*p* < 0.001), present parametrial invasion (*p* = 0.013), lymphovascular space invasion (LVSI) (*p* < 0.001), and lymph node metastasis (*p* = 0.002) (Table S1, Fig. S1a–e, Supporting Information). High-risk HPV (hrHPV) infection is a major risk factor for cervical cancer, but no significant association was found between PNI and hrHPV infection (*p* = 0.397) (Table S1, Supporting Information), similar to the findings in PNI of HNSCC [15, 16]. Similarly, p16 expression, an hrHPV infection marker, showed no notable difference between Non-PNI (*n* = 8) and PNI (*n* = 6) tissues (*p* = 0.9588) (Fig. S1f, Supporting Information). This may be because hrHPV infection is crucial for cervical cancer carcinogenesis, while PNI is important for its spread [17, 18]. Thus, PNI is the characteristic of advanced cervical cancer.

Neurogenesis and increased tumor innervation occur in the PNI process of solid tumor [10], and we examined the expression of PGP9.5 (neuronal marker) and NF-L (newborn neuronal marker) to evaluate the neurogenesis during PNI in cervical cancer. Both PGP9.5 and NF-L were determined to be increased in PNI-positive cervical cancer tissues (Fig. 1b), suggesting the neurogenesis during PNI. However, staining of TH (tyrosine hydroxylase, dopaminergic neuron marker) or VAChT (choline acetyltransferase, cholinergic neuron marker) was not significantly increased in PNI-positive cervical cancer tissues (Fig. S1g, Supporting Information), indicating that sympathetic or parasympathetic nerve, resident in the cervix, were not directly involved in the neurogenesis during PNI of cervical cancer. Hence, neurogenesis is the characteristic of nerve infiltration and PNI in cervical cancer.

### Cervical cancer promotes neurogenesis of axons through activating Schwann cells

An in vitro model of co-culturing cervical cancer and dorsal root ganglion (DRG) was used to investigate their crosstalk. When co-cultured with cervical cancer, the neurites of DRG outgrew toward tumor cell clusters (Fig. 1c). Then, we performed staining of pan-cytokeratin (Pan-CK) (epithelial cell marker to indicate tumor) and PGP9.5 to trace their interaction, and found the formed bridges connecting DRG and tumor cluster, and cervical cancer cells migrated toward and along the bridges (Fig. 1d). The living cell imaging system with contentious capturing was then used, and the neurite outgrow of DRG toward tumor cluster was found to be induced by its co-culture with cervical cancer, and tumor cells were stationary before their direct interaction with the regenerated neurites (Fig. 1e; Supplementary Movie 1 and 2, Supporting Information). As axonal outgrowth and nerve regeneration is reported to be dependent on the activation of Schwann cells [19], we co-cultured Schwann cell RSC96 with cervical cancer cells in an in vitro 3D Matrigel system, and the invasion of Schwann cells (mScarlet^+^ red) was initiated by their co-culture with cancer cells (Zsgreen), but not cervix epithelial HerEpic cells (Zsgreen) (Fig. 1f, g). Thus, cervical cancer promotes neurogenesis of axons through the activation of Schwann cells.

The migration of cervical cancer cells toward their connection with the regenerated neurites was then investigated. When co-cultured with DRG, cervical cancer cells migrated toward DRG, which was in the central of dark-field imaging (Fig. 1h). The migrated Schwann cells and regenerated axon contacted the adjacent tumor cell cluster repeatedly at a high velocity (Supplementary Movies 1 and 2, Supporting Information), and then inserted into the intracellular space of tumor cells to disassociate their connections, causing the membrane protrusions of tumor cells and their migration along the regenerated neurites (Fig. 1i; Supplementary Movie 3, Supporting Information). Accordingly, we co-cultured cervical cancer cells and DRG, and the membrane protrusions morphological changes of cervical cancer cells were confirmed by confocal microscopy (Fig. 1j). Together, cervical cancer cells promote the activation of Schwann cells and regeneration of nitrites to build the bridges, and then migrate along the bridges to form PNI.

### Cervical cancer-produced NMB triggers PNI

**Fig. 2:**
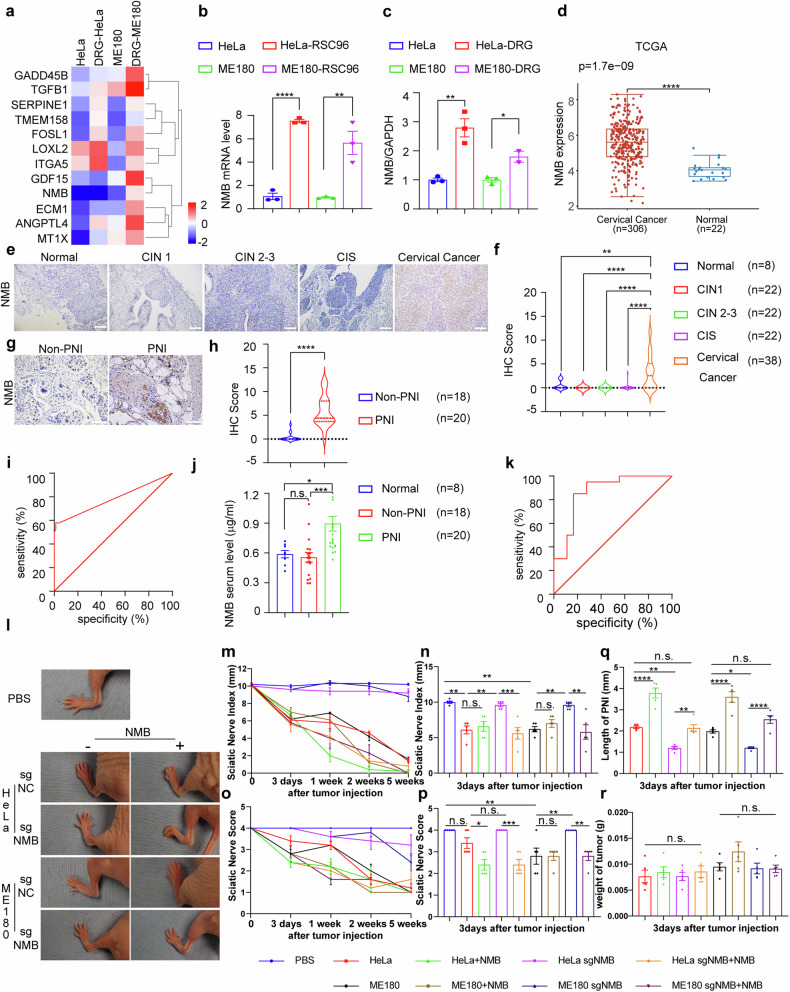
Cervical cancer-produced NMB triggers PNI. **a** mRNA levels of differential genes were analyzed using RNA-seq in tumor cells co-cultured with DRG. Quantification of NMB mRNA (**b**) and protein (**c**) in tumor cells co-cultured with RSC96 cells/DRG was shown (*n* = 3). **d** Quantification of NMB mRNA in specimens of cervical cancer patients (*n* = 306) and healthy women (*n* = 22) from TCGA and GTEx database was shown. **e**, **f** Immunohistochemistry (IHC) staining and relative IHC score of NMB were analyzed in normal (*n* = 8), CIN1 (*n* = 22), CIN2-3 (*n* = 22), CIS (*n* = 22) and cervical cancer (*n* = 38) tissues. ×25 magnification, scale bars: 1000 μm. **g**, **h** Immunohistochemistry (IHC) staining and relative IHC score of NMB were analyzed in patients with PNI (*n* = 20) and without PNI (*n* = 18). ×200 magnification, scale bars: 50 μm. **i** ROC curve for the sensitivity and specificity of NMB in detecting PNI by IHC staining. ROC, receiver operating characteristic. **j** Serum NMB was examined in healthy women (*n* = 8) and cervical cancer patients with (*n* = 20) or without (*n* = 18) PNI. **k** ROC curve for the sensitivity and specificity of NMB in serum tested by ELISA in detecting PNI. **l** Representative images of mice three days after tumor injection revealing left hind limb function in the normal PBS, sgNC, and sgNMB with/without recombinant NMB groups (*n* = 5). **m** Quantification of sciatic nerve function index (SFI) (paw span in millimeters) of mice in different groups at three days, one, two, and five weeks after tumor injection was shown (*n* = 5). **n** Quantification of SFI between different groups at day three after tumor implantation was shown (*n* = 5, one-way ANOVA and Tukey’s multiple comparisons test). **o** Quantification of the mean left sciatic nerve function scores of mice in different groups at three days, one, two, and five weeks after tumor injection was shown (*n* = 5). The score of 4 indicates normal function; score of 1 indicates total paralysis; a higher score indicates better sciatic nerve function. **p** Quantification of sciatic nerve function scores between different groups on day three after umor implantation was shown (*n* = 5, one-way ANOVA and Tukey’s multiple comparisons test). **q** Quantification of the length of PNI as measured by ultrasound imaging was shown (*n* = 5, one-way ANOVA and Tukey’s multiple comparisons test). **r** Quantification of tumor weight between different groups on day three after tumor implantation was shown (*n* = 5, one-way ANOVA and Tukey’s multiple comparisons test). *P < 0.05, **P < 0.01, ***P < 0.001, ****P < 0.0001.

We next examine the underlying mechanism responsible for nerve-tumor crosstalk in PNI of cervical cancer. The transcriptome profiling was performed in HeLa and ME180 cells with or without DRG in the non-contact co-culture system. Among the upregulated genes in both cell lines co-cultured with DRG, twelve genes annotated as neurotransmitters or neuropeptides were screened out (Fig. 2a; Table S2, Supporting Information). The expression of these genes in cervical cancer cells co-cultured with RSC96 cells or DRG were analyzed by qRT-PCR (Fig. S2a, Supporting Information), and we found that *NMB* mRNA and protein was the most increased in the co-culture system (Fig. 2b, c; Fig. S2b–d, Supporting Information), suggesting that cervical cancer-derived neuropeptide NMB may initiate PNI in cervical cancer.

To determine the potential role of NMB in PNI of cervical cancer, we examined the production of NMB in cervical cancer tissues, especially in those with PNI. NMB mRNA was significantly increased in cervical cancer tissues as compared to that in cervix epithelium in the TCGA database (https://portal.gdc.com) (Fig. 2d), and its protein level was also increased in cervical cancer tissues, while not significantly elevated in cervical intraepithelial neoplasia (CIN) and cervical carcinoma in situ (CIS) tissues (Fig. 2e, f). Furthermore, the NMB protein level was significantly higher in cervical cancer tissues with PNI than those without PNI (Fig. 2g, h). The diagnostic value of NMB in PNI of cervical cancer was then analyzed using receiver operating characteristic (ROC) curve, and the value of area under ROC curve (AUC) is 0.7800 (0.676–0.884, 95% CI) (Fig. 2i). Since NMB is a secretory protein, its serum level was also significantly increased in cervical cancer patients with PNI compared to those without PNI (Fig. 2j), with the activity in diagnosing PNI at an AUC value of 0.864 (0.741–0.987, 95% CI) (Fig. 2k). Previous studies indicate that NMB is influenced by hormonal balance, including stress hormones and estrogen [20–23]. We examined the impact of 9 chronic stress hormones and estrogen on NMB transcript levels in HeLa and ME180 cells and found that estrogen, l-glutamic acid, triiodothyronine (T3), and γ-GABA may upregulate NMB mRNA in these cells (Fig. S2e, f, Supporting Information). Thus, the heterogeneity of NMB expression in tumor tissues may be caused by the heterogeneity of hormone expression. Together, the clinical significance of NMB in the progression of PNI is suggested, and serum NMB level may be an indicator for the diagnosis of PNI.

To elucidate the role of NMB in the onset of PNI, we created *NMB*-deficient HeLa and ME180 cells by the CRISPR/Cas9 approaches (Fig. S3a-c, Supporting Information). ELISA showed a significant decrease in NMB concentration in the cell supernatant of RSC96 cells / DRG co-cultured with these NMB-deficient tumor cells (Fig. S3d, e, Supporting Information). Then, we established a heterotopic xenograft sciatic nerve model. The limb function of nude mice was damaged by the injection of cervical cancer cells, and the injured nerve function was alleviated in the groups of NMB-deficient cervical cancer cells three days after injection, which is reversed by the administration of recombinant NMB (Fig. 2l-p), suggesting that cervical cancer-produced NMB initiate the onset of PNI. The analysis of high-frequency ultrasound (HFUS), anatomical analysis, and HE staining were then performed, and the migration distance of cervical cancer cells along sciatic nerve decreased by NMB deficiency were observed (Fig. 2q, r; Fig. S3f–j, Supporting Information). Moreover, the innervation of cervical cancer was also analyzed by PGP9.5 staining, and it was also decreased by NMB deficiency, which is rescued by NMB administration (Fig. S3k, Supporting Information). The same effect was observed at one, two, and five weeks after tumor injection (Figs. S4, S5, S6, Supporting Information). Taken together, we conclude that cervical cancer-derived NMB triggers PNI.

### Cervical cancer-produced NMB reprograms and activates Schwann cells

**Fig. 3:**
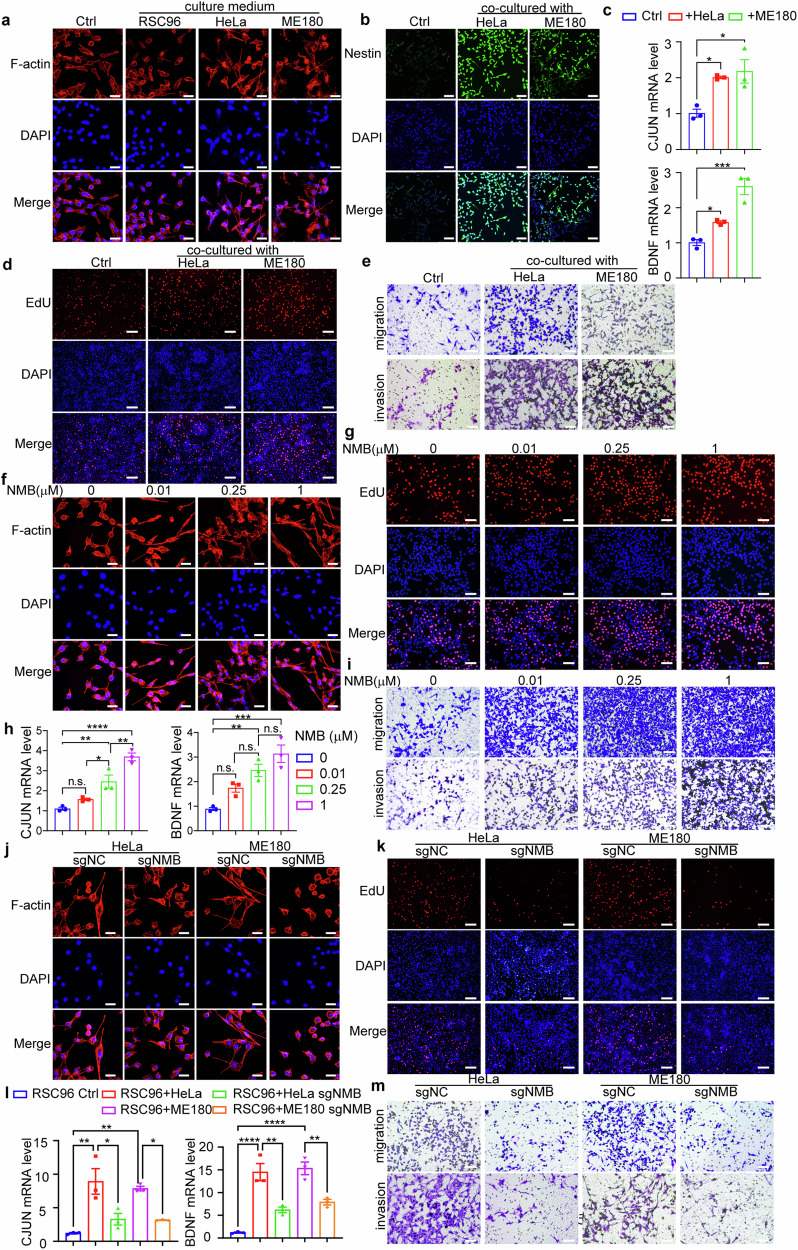
Cervical cancer-produced NMB reprograms and activates Schwann cells. **a** Phalloidin staining was analyzed in Schwann cells treated with supernatant from RSC96/HeLa/ME180 cells. Red, Phalloidin, F-actin staining. Blue, DAPI, Nuclei. Scale bars: 25 μm. **b** Immunofluorescent Nestin staining was analyzed in Schwann cells co-cultured with HeLa and ME180 cells. Scale bars: 500 μm. **c** cJUN and BDNF mRNA expression of cocultured RSC96 cells with cervical cancer cells were examined by qRT-PCR (*n* = 3, one-way ANOVA and Tukey’s multiple comparisons test). **d** EdU staining (red) was analyzed in RSC96 cells cocultured with HeLa and ME180 cells. Scale bars: 50 μm. **e** Cell migration and invasion of Schwann cells co-cultured with HeLa and ME180 cells were examined by Boyden chamber assay with or without Matrigel-coated inserts. Scale bars: 50 μm. **f** Phalloidin staining was analyzed in Schwann cells treated at gradient concentrations of NMB. Scale bars: 25 μm. **g** EdU staining was analyzed in RSC96 cells treated with NMB. Scale bars: 50 μm. **h** cJUN and BDNF mRNA expression of RSC96 cells upon treatment with different concentrations of NMB were examined by qRT-PCR (*n* = 3, one-way ANOVA and Tukey’s multiple comparisons test). **i** Cell migration and invasion assays of Schwann cells treated with NMB were examined by Boyden chamber assay with or without Matrigel-coated inserts. Scale bars: 50 μm. **j** Phalloidin staining was analyzed in Schwann cells treated with NMB knockout HeLa or ME180 cells. Scale bars: 25 μm. **k** EdU staining was analyzed in RSC96 cells treated with NMB knockout HeLa or ME180 cells. Scale bars: 50 μm. **l** cJUN and BDNF mRNA expression of cocultured RSC96 cells with NMB knockout cervical cancer cells were examined by qRT-PCR (*n* = 3, one-way ANOVA and Tukey’s multiple comparisons test). **m** Cell migration and invasion of Schwann cells treated with NMB knockout HeLa or ME180 cells were examined by Boyden chamber assay with or without Matrigel-coated inserts. Scale bars: 50 μm. Data are shown as photographs from one representative of three independent experiments. *P < 0.05, **P < 0.01, ***P < 0.001, ****P < 0.0001.

Since the regeneration of neurites is dependent on the reprogramming of Schwann cells [24], we then examined whether Schwann cells were activated by cervical cancer cells in vitro. Phalloidin staining showed that Schwann cells RSC96 acquired characteristic bipolar shape, rather than flattened fibroblast-like shape, after the culture with Hela/ME180 cells conditioned culture medium, and displayed lower actin cytoskeleton and filopodia (Fig. 3a; Fig. S7a, b, Supporting Information). Furthermore, protein and mRNA level of Nestin, a neural stem cell marker, was increased in RSC96 cells co-cultured with cervical cancer cells by transwell analysis (Fig. 3b; Fig. S7c, d, Supporting Information), suggesting the reprograming of Schwann cells. The reprogramming-related genes, including transcription factors (*CJUN*), myelin genes (*MBP*), neurotrophic factors and receptors (*BDNF, NGFR*) and axonal regenerative marker (*GAP43*) were significantly elevated upon RSC96 cells co-cultured with cervical cancer cells (Fig. 3c; Fig. S7e, Supporting Information). The proliferation and migration of RSC96 cells were also significantly promoted by the co-culture with cervical cancer cells by EdU staining and transwell assays respectively (Fig. 3d, e; Fig. S7f, g, Supporting Information). Thus, Schwann cells are reprogrammed and activated by the co-culture with cervical cancer cells.

To investigate whether the reprograming of Schwann cells is dependent on NMB, we treated RSC96 cells with recombinant NMB, and found the changed morphology, increased expression of reprogramming genes, and enhanced proliferation and motility (Fig. 3f–i; Fig. S7h–l, Supporting Information), suggesting the NMB-promoted reprogramming of Schwann cells. The activated glial cell marker GFAP was also increased in RSC96 cells upon NMB administration (Fig. S7m, Supporting Information). Furthermore, the reprograming and activation of Schwann cells was decreased by the co-culture with *NMB*-deficient HeLa or ME180 cells, confirming that cervical cancer-derived NMB promotes the reprogramming of Schwann cells (Fig. 3j–m; Fig. S7n–q, Supporting Information). Together, NMB derived from cervical cancer cells initiates the reprograming and activation of Schwann cells, which may in-turn trigger neurite regeneration and PNI onset.

### NMB receptor in Schwann cells mediates PNI of cervical cancer

**Fig. 4:**
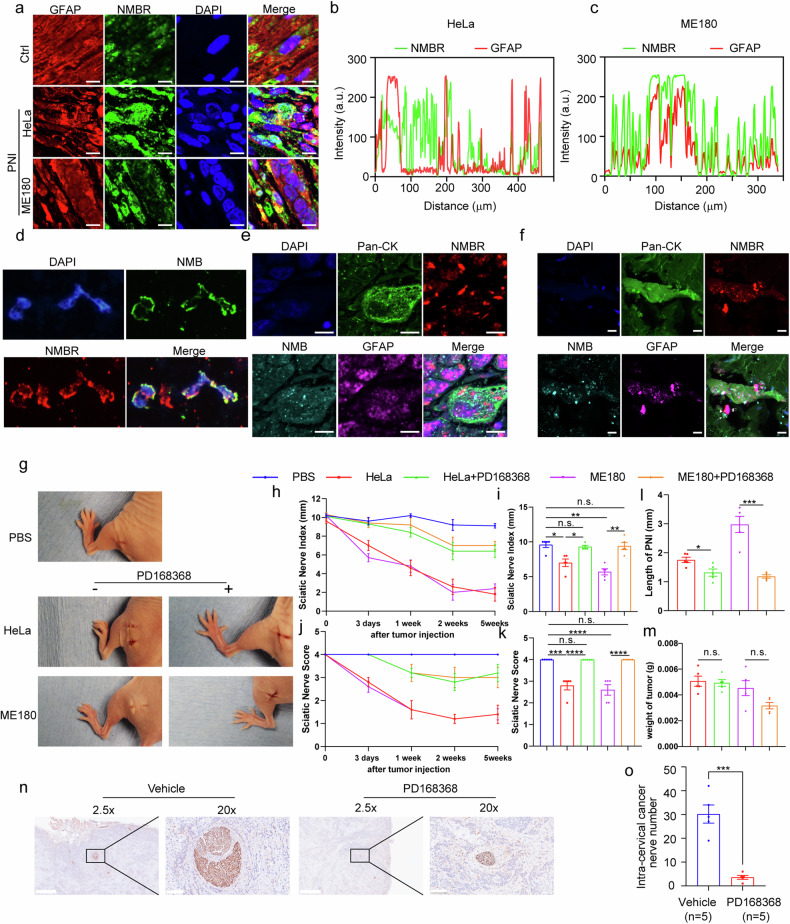
NMB receptor in Schwann cells mediates PNI of cervical cancer. **a** Immunofluorescent GFAP (red) and NMBR (green) staining were analyzed in sciatic nerve PNI model conducted with HeLa (middle) and ME180 cells (lower). Scale bars: 7.5 μm. **b**, **c** Pearson colocalization coefficient (Rr) analysis of GFAP and NMBR in (**a**) HeLa co-cultured group (**b**) and ME180 co-cultured group (**c**) were shown. **d** Immunofluorescent NMB (green) and NMBR (red) staining were analyzed in Schwann cells co-cultured with HeLa cells. **e**, **f** Multicolor fluorescence staining was analyzed in specimens of cervical cancer injected mice (**e**) and cervical cancer patients with PNI (**f**). Green: Pan-CK, cyan: NMB, purple: GFAP, red: NMBR. Scale bars: 10 μm. **g** Representative images of mice three days after tumor injection revealing left hind limb function in the normal PBS, HeLa/ME180 with or without PD168368 groups (*n* = 5). **h** Quantification of SFI (paw span in millimeters) in PNI mice treated with or without PD168368 at three days, one, two, and five weeks after tumor injection was shown (*n* = 5). **i** Quantification of SFI in PNI mice treated with or without PD168368 at day three after tumor implantation was shown (*n* = 5, one-way ANOVA and Tukey’s multiple comparisons test). **j** Quantification of sciatic nerve function scores in PNI mice treated with or without PD168368 at three days, one, two, and five weeks after tumor implantation was shown (n = 5). **k** Quantification of sciatic nerve function scores in PNI mice treated with or without PD168368 at day three after tumor implantation was shown (n = 5, one-way ANOVA and Tukey’s multiple comparisons test). **l** Quantification of the length of PNI as measured by ultrasound imaging in PNI mice treated with or without PD168368 was shown (*n* = 5, one-way ANOVA and Tukey’s multiple comparisons test). **m** Quantification of tumor weight in PNI mice treated with or without PD168368 at day three after tumor implantation was shown (*n* = 5, one-way ANOVA and Tukey’s multiple comparisons test). **n** Neurons were analyzed by PGP9.5 staining in ME180 subcutaneous tumor-bearing mice treated with NMBR antagonist PD168368. scale bars: left,1 mm; right, 100 μm. **o** Quantification of intra-cervical cancer nerve numbers in ME180 subcutaneous tumor-bearing mice treated with NMBR antagonist PD168368 was shown (*n* = 5, unpair *t*-test). Data are shown as photographs from one representative of three independent experiments. *P < 0.05, **P < 0.01, ***P < 0.001, ****P < 0.0001.

NMBR is the high-affinity receptor of NMB, and we presumed that NMB may trigger PNI via the NMBR of Schwann cells. The co-localization of NMBR and GFAP were observed in tumor-invading sites of sciatic nerve (Fig. 4a), with a Pearson’s colocalization coefficient of 0.41 and 0.81 in Hela and ME180 PNI models (Fig. 4b, c), presenting that NMBR is located in activated Schwann cells. The expression of NMBR was increased in the in vitro co-culture model, and human PNI tissues (Fig. S8a–c, Supporting Information). The co-localization of NMB and NMBR on the membrane of Schwann cells was then examined, and it was observed in RSC96 cells in vitro, PNI model in vivo, and human PNI tissues (Fig. 4d–f), and NMB and NMBR accumulated and co-localized in the cell contact area of NMB^+^ Pan-CK^+^ tumor cells and NMBR^+^GFAP^+^ Schwann cells in both mouse PNI model and human PNI tissues (Fig. 4e, f). Thus, these data present that NMBR in activated Schwann cells may medicate the NMB-initiated PNI.

The NMBR-specific antagonist PD168368 was then used to confirm the NMBR-initiated PNI of cervical cancer. When the binding between NMB and NMBR was blocked, the damaged limb function three days post cervical cancer inoculation was reduced (Fig. 4g), showed by the alleviated sciatic nerve index and sciatic nerve score (Fig. 4h–k). The ultrasonic imaging and anatomical analysis also presented that inhibition using PD168368 suppressed the spread of cervical cancer along sciatic nerves, with no significant difference in tumor weight between groups (Fig. 4l, m; Fig. S8d–f, Supporting Information). Moreover, NMBR antagonist reduced the damage of nerves by the inoculation of cervical cancer cells, showed by HE and PGP9.5 staining (Fig. S8g, h, Supporting Information). After tumor injection into sciatic nerves for one, two, and five weeks, as shown in mouse models, PD168368 could achieve the similar effect (Figs. S8i–o, S9a–n, Supporting Information). Moreover, NMBR antagonist inhibited the PNI of cervical cancer in the ME180 subcutaneous tumor-bearing model (Fig. 4n, o). Together, we conclude that NMBR mediates NMB-initiated PNI, which can be blocked by the antagonist.

### NMBR mediates the NMB-triggered reprogramming of Schwann cells

**Fig. 5:**
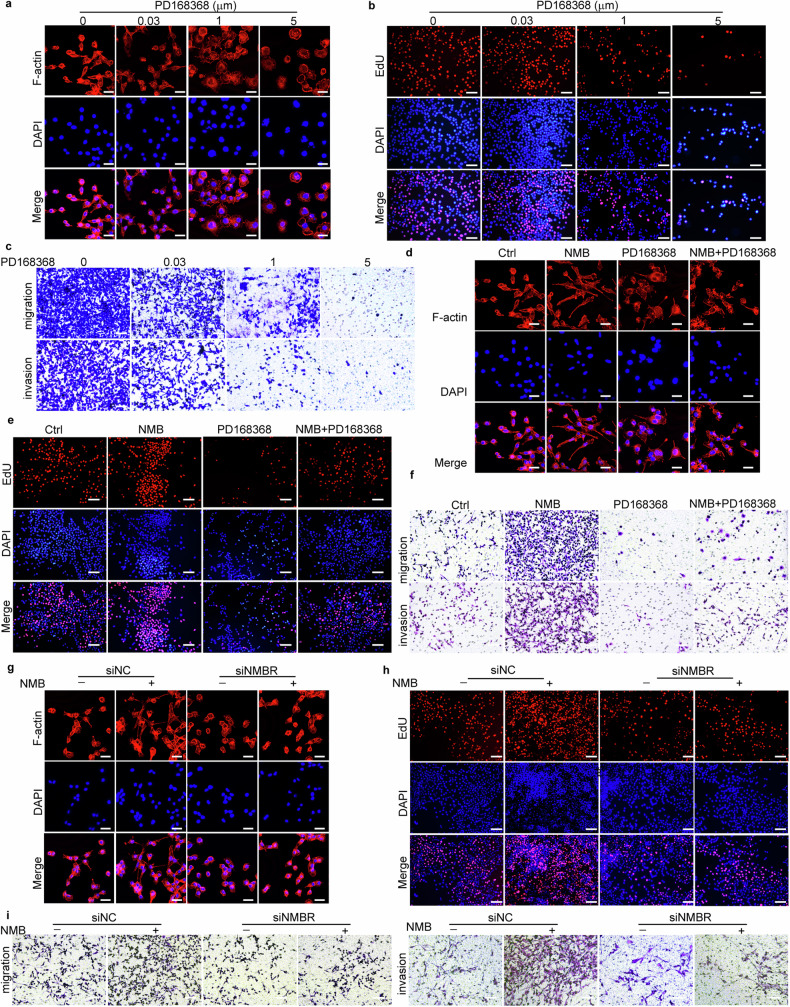
NMBR mediates the NMB-triggered reprogramming of Schwann cells. **a** Phalloidin staining was analyzed in Schwann cells treated with NMBR antagonist PD168368. Red, Phalloidin, F-actin staining. Blue, DAPI, Nuclei. Scale bars: 25 μm. **b** EdU staining was analyzed in Schwann cells treated with NMBR antagonist PD168368. Scale bars: 50 μm. **c** Cell migration and invasion of Schwann cells treated with NMBR antagonist PD168368 were examined by Boyden chamber assay with or without Matrigel-coated inserts. Scale bars: 50 μm. Phalloidin staining (**d**, scale bars: 25 μm), EdU staining (**e**), and cell migration and invasion (**f**) were analyzed in Schwann cells treated with NMB and PD168368. Phalloidin staining (**g**, scale bars: 25 μm), EdU staining (**h**), and cell migration and invasion (**i**) were analyzed in NMBR-knockdown Schwann cells treated with NMB. Photographs from one representative of three independent experiments were shown.

Schwann cells undergo extensive reprogramming to induce morphological changes and trigger axon regeneration, which is essential for the PNI of tumor cells [25]. We further investigated whether NMB-induced activation of Schwann cells was mediated by its membrane receptor NMBR. The inhibitor PD168368 was administrated into the Schwann RSC96 cells and cervical cancer ME180 cells co-culture system. The morphological transition, cell proliferation, migration and invasion, and induction of reprograming genes of co-cultured RSC96 cells were all suppressed by the NMBR inhibitor (Fig. 5a–c; Fig. S10a–c, Supporting Information). Similar results were observed in the cell model of NMB-induced Schwann cell activation (Fig. 5d–f; Fig. S10e–i, Supporting Information). We also knocked down the expression of NMBR in RSC96 cells, and determined the suppressed morphological transition, cell proliferation, migration and invasion, and induction of reprograming genes in the system of NMB-induced Schwann cell activation (Fig. 5g–i; Fig. S10j–m, Supporting Information). Thus, NMBR of Schwann cells mediates the NMB-triggered reprogramming.

### The NMB-NMBR ligation activates T-type calcium channels by PKA activation

**Fig. 6:**
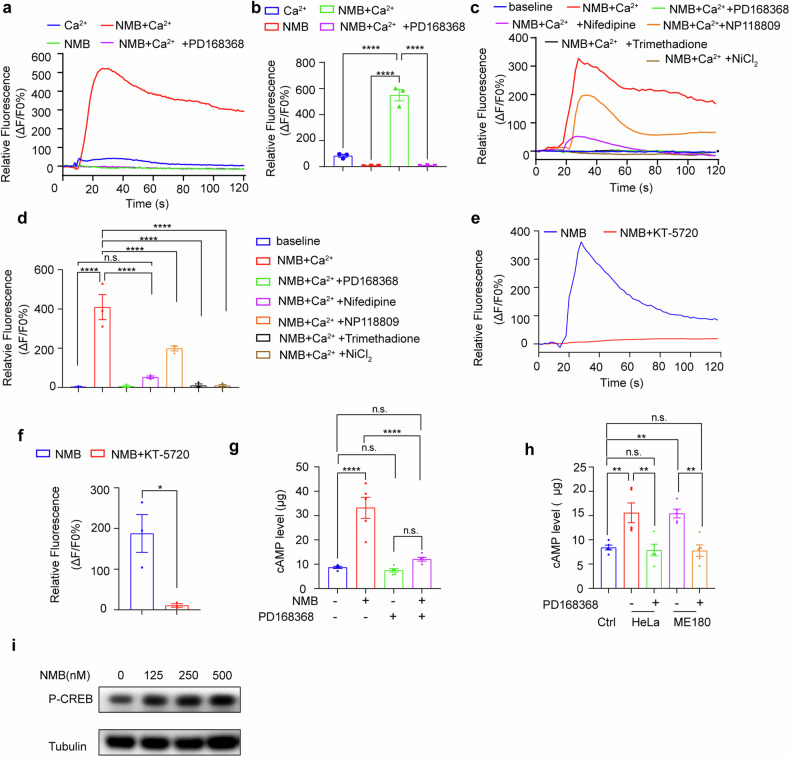
The NMB-NMBR ligation activates T-type calcium channels by PKA activation. **a** The intracellular Ca^2+^ concentration was analyzed in Schwann cells treated with NMB and PD168368 by measuring the fluorescence intensity of the calcium indicator Fluo-4/AM with a confocal microscope. **b** The statistics of peak calcium responses were analyzed in **a** (one-way ANOVA and Tukey’s multiple comparisons test). **c** The intracellular Ca^2+^ concentration was analyzed in Schwann cells treated with NMB, PD168368, Nifedipine, NP118809, Trimethadione and NiCL2 by measuring the fluorescence intensity of the calcium indicator Fluo-4/AM with a confocal microscope. **d** The statistics of peak calcium responses were analyzed in **c** (one-way ANOVA and Tukey’s multiple comparisons test**)**. **e** The intracellular Ca^2+^ concentration was analyzed in Schwann cells treated with KT-5720 by measuring the fluorescence intensity of the calcium indicator Fluo-4/AM with a confocal microscope. **f** The statistics of peak calcium responses were analyzed in **e** (unpair *t*-test). **g** The intracellular cAMP concentration was analyzed in RSC96 cells treated with NMB and PD168368 by ELISA (*n* = 5, one-way ANOVA and Tukey’s multiple comparisons test). **h** The intracellular cAMP concentration was analyzed in RSC96 cells cocultured with HeLa or Me180 with or without PD168368 (*n* = 5, one-way ANOVA and Tukey’s multiple comparisons test). **i** CREB phosphorylation were examined in RSC96 cells treated with a concentration gradient of NMB. Data are shown as photographs from one representative of three independent experiments. *P < 0.05, **P < 0.01, ***P < 0.001, ****P < 0.0001.

The molecular mechanism responsible for NMB-induced activation of Schwann cells was then studied. As NMB induces the open of calcium channels in neurons and NMBR belongs to the G protein-coupled receptor (GPCR) family [26], we examined whether NMB-NMBR ligation led to the open of calcium channel of Schwann cells. NMB induced a distinct Ca^2+^ influx in RSC96 cells in the existence of calcium, and it was blocked by PD168368 (Fig. 6a, b). Among the three types of calcium channels, T-type calcium channel was identified to mediate the Ca^2+^ influx stimulated by NMB (Fig. 6c, d). Since T-type calcium channel-mediated calcium influx depends on the activation of PKA [27], we further administered the PKA inhibitor KT-5720, and found that its pre-treatment blocked the NMB-induced Ca^2+^ influx (Fig. 6e, f), suggesting that NMB activates T-type calcium channels in a PKA-dependent manner. We then examined the levels of cAMP and cAMP response element-binding protein (CREB) to evaluate the effects downstream of NMBR, and found the elevated cAMP and activated CREB following NMB administration (Fig. 6g–i). Hence, NMB-NMBR ligation activates T-type calcium channels and induces the reprograming of Schwann cells.

### NMB-activated Schwann cells promote PNI by secreting CCL2

**Fig. 7:**
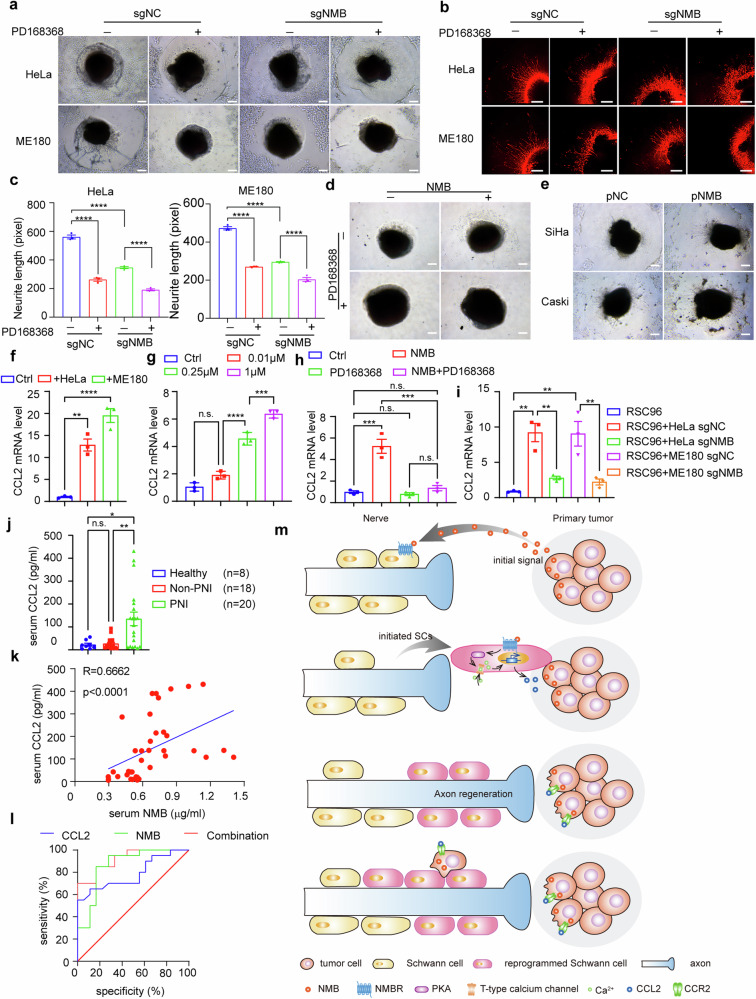
NMB-activated Schwann cells promote PNI by secreting CCL2. Representative brightfield (**a**) and PGP9.5 staining (**b**) images were shown as indicated in PD168368 suppressed PNI in DRG model co-cultured with NMB knockout tumor cells. DRG was placed in the center of Matrigel. Scale bars: 200 μm. **c** Quantification of neurite length in (**b**) was shown (*n* = 3, one-way ANOVA and Tukey’s multiple comparisons test). **d** Representative brightfield images were shown as indicated in cancer cell-DRG co-culture model treated with NMB or/and PD168368. DRG was placed in the center of Matrigel. 50×magnification, scale bars: 200 μm. **e** Representative brightfield images were shown as indicated in DRG model co-cultured with NMB overexpressed CaSki and SiHa cells. DRG was placed in the center of Matrigel. ×50 magnification, scale bars: 200 μm. **f**–**i** CCL2 mRNA expression of RSC96 cells cocultured with HeLa and ME180 cells (**f**), NMB recombinant protein (**g**), NMB and PD168368 (**h**), and NMB knockout tumor cells were examined by qRT-PCR (*n* = 3, one-way ANOVA and Tukey’s multiple comparisons test). **j** Serum CCL2 was examined in healthy women (*n* = 8), cervical cancer patients with and without PNI (Non-PNI patients, *n* = 18; PNI positive patients, *n* = 20. One-way ANOVA and Tukey’s multiple comparisons test). **k** The correlation between CCL2 and NMB expression in cervical cancer patients’ serum was analyzed by Pearson’s correlation coefficient assay (*n* = 38). **l** ROC curve for the sensitivity and specificity of CCL2 combined with or without NMB in serum tested by ELISA in detecting cervical cancer PNI (*n* = 38). **m** Working model for cervical cancer-produced NMB triggers PNI. First, cervical cancer cells produce neuropeptide NMB, which is the initial signal to induce PNI. Second, NMB activates Schwann cells through its receptor NMBR-mediated calcium influx by PKA activation, thus triggering Schwann cell reprogramming and secreting CCL2. Third, the activated Schwann cells promote axon regeneration to form the direct contact with cervical cancer cells. Fourth, CCL2 attracts and facilitates the invasion of cervical cancer cells along the regenerated neurites. All these sequential steps lead to the initiation and progression of PNI in cervical cancer. Data are shown as photographs from one representative of three independent experiments. *P < 0.05, **P < 0.01, ***P < 0.001, ****P < 0.0001.

We next examined whether the activated Schwann cells promoted PNI of cervical cancer. The effect of NMB on axon regeneration was analyzed in the DRG-cervical cancer co-culture model, and the regenerated neurites were significantly decreased by the deficiency of NMB or the inhibition of NMBR (Fig. 7a–c). The extension of DRG neurites was also observed following recombination NMB treatment, which was abolished by the inhibition using PD168368 (Fig. 7d). We previously found that PNI did not occur in cervical cancer SiHa and CaSki cells (Fig. 7e) [14], and NMB was determined to be not expressed by these two cell lines (Fig. S11a, Supporting Information). However, we overexpressed NMB in these two cell lines, and determined the induced neurite growth following NMB overexpression (Fig. 7e; Fig. S11b, Supporting Information). Thus, NMB-activated Schwann cells trigger axon regeneration, which may promote PNI of cervical cancer.

As we previously determined that CCL2 secreted by activated Schwann cells induced PNI of cervical cancer [14], and calcium influx is reported to activate the secretion of CCL2 in DRGs [28], we next examined whether NMB-activated Schwann cells could produce CCL2 to recruit cervical cancer cells to migrate toward the regenerated neurites. CCL2 expression in RSC96 cells was markedly induced by its co-culture with cervical cancer cells (Fig. 7f), and its expression could be induced by the administration of recombinant NMB (Fig. 7g), while this induction was abolished by the inhibition using PD168368 or deficiency of NMB (Fig. 7h, i). The serum level of CCL2 in cervical cancer patients was also examined, and it was significantly higher in patients with PNI, positively correlated with serum NMB, and significantly predicted PNI (Fig. 7j-l). Furthermore, using both serum CCL2 and NMB, it presented a better discrimination in predicting PNI with an AUC of 0.919 (95% CI 0.838-1.000), suggesting that the combined examination of NMB and CCL2 may be a useful approach to diagnose PNI in cervical cancer patients (Fig. 7l). Altogether, we conclude that NMB is produced by cervical cancer cell, causing the reprograming and activation of Schwann cells, which then secrets CCL2 to initiate PNI of cervical cancer.

## Discussion

The direct contact and dynamic crosstalk between nerve cells and tumor cells have been demonstrated to be the key step in PNI [11, 29], but whether the former or the latter is the first cell to trigger PNI onset is still controversial. Since Schwann cells can chemoattract cancer cells to travel along neurites by direct contact and induce the formation of cancer cell protrusions in their direction in pancreatic cancer [13], it seems that nerve innervation occurs earlier than tumor invasion in a Schwann cell-dependent manner. Therefore, we recorded continuous tracks of both nerve cells and tumor cells with a live cell imaging system to identify the initiation cell. Indeed, Schwann cells undergone extensive morphological changes and active movements even in the early phase of PNI, while tumor cells remained in their growth state. To some extent, this process is similar to the plasticity of Schwann cells that is observed after nerve injury. Schwann cell reprogramming is the key process that triggers axon regeneration, which is essential for nerve innervation during nerve repair [30]. In cervical cancer, Schwann cells were transcriptional reprogrammed, which allowed them to initiate PNI onset. First, Schwann cells separated from neurites and formed a “Büngner zone”-like structure, which could both guide axon regeneration and provide a “bridge” to direct the invasion of cancer cells. Then, the activated Schwann cells moved towards the tumor site and inserted themselves between tumor cells. Finally, the inserted Schwann cells were in direct contact with tumor cells and released the tumor cells from the tumor cluster, allowing further invasion. Interestingly, only tumor cells that were repeatedly and rapidly contacted by Schwann cells could be dissociated from the tumor cluster, which indicates that the separation of cancer cells occurred in a Schwann cells-dependent manner.

Although Schwann cells were thought to be the first cells to initiate PNI in cervical cancer, but the mechanisms underlying the activation of Schwann cells remained unclear. As previously reported, PNI did not occur in all cervical cancer cell lines [14], suggesting that its initiation may depend on the molecular characteristics of tumor cells. Neuropeptides have been shown to promote oncogenesis and axonogenesis [31]. In this study, NMB derived from cervical cancer cells was identified to initiate Schwann cell reprogramming and activation. NMB and its high affinity receptor, NMBR, are extensively distributed in the central nervous system as well as peripheral tissues, including the urogenital and reproductive systems, endocrine glands and immune cells. In addition to its physiological functions, the NMB-NMBR axis participates in a wide variety of pathological activities, especially in malignancies such as lung cancer, colorectal cancer, glioma, breast cancer, and cervical cancer [32–35]. NMB has been reported to promote the growth, differentiation and invasion of these tumor cells in an autocrine manner [36]. However, the role of NMB in cervical cancers is limited to tumor proliferation via ERK1/2 and NF-κB pathways and TNF-α upregulation [37]. Its potential link to PNI in cervical cancer has not been previously explored. Here, we propose a new paracrine effect via which tumor-derived NMB initiates the PNI process by directly activating Schwann cells. NMB secreted from tumor cells induced Schwann cell activation and axonal extension, which could be blocked by the selective NMBR antagonist PD168368. In addition to the promoted proliferation, migration and invasion of Schwann cells, NMB caused extensive morphological changes in Schwann cells by activating NMBR during PNI development. Furthermore, NMB stimulates the expression of a set of genes that are related to Schwann cell reprogramming, further confirming the promotive effect of NMBR on Schwann cell activation. Thus, tumor-derived NMB acts as a signal that initiates PNI by reprogramming Schwann cells via NMBR in cervical cancer.

Since NMBR belongs to the G protein-coupled receptor (GPCR) family, the NMB-NMBR axis generally performs its function by triggering the Ca^2+^ influx of target cells. A recent study observed that NMB enhanced the NMBR-mediated activation of Cav3.2 T-type calcium channel in neurons in persistent perineural pain hypersensitivity by activating the AMPK/PKA downstream signaling [38], but there is little evidence confirming the role of NMB in Schwann cell calcium channel and calcium influx. Indeed, our data also showed that NMB-NMBR signaling stimulated T-type calcium channel and induced a Ca^2+^ influx in Schwann cells through PKA signaling. In addition to its direct role in target tissues, NMB plays an indirect role by promoting the release of other gastrointestinal hormones and neurotransmitters [39]. It is worth noting that the NMB-NMBR axis can mediate the release of chemokines, such as IL-4 in the intestine, IL-8 in neuroblastoma, and VEGF in tumor [40]. We previously demonstrated that Schwann cells secrete the chemokine CCL2 to promote the invasion of tumor cells along neurites. In this study, we also found that NMB induced the secretion of CCL2 by Schwann cells via a NMBR-dependent induction of a Ca^2+^ influx, which determines this NMB-NMBR-CCL2 axis in the activation of Schwann cells and the initiation of PNI in cervical cancer. In this way a positive feedback loop could be formed that enhances PNI in cervical cancer.

PNI is an early pathological feature of cervical cancer metastasis that may be overlooked [41], and preoperative diagnosis of PNI is critical in the selection of NRSH for early-stage cervical cancer patients [4]. However, imaging examination, particularly MRI and PET/CT scanning, showed limited advantages for the diagnosis of nerve innervation before surgical treatment in some malignancies [6–8], and their diagnostic value for PNI in cervical cancer has not been determined. Recently, biomarkers that are involved in the tumor-nerve interaction of PNI have become the main focus of research on the possible underlying mechanisms and their potential use in preoperative diagnosis. The predominant molecular mediators of PNI include neurotrophins, chemokines, cell adhesion factors, axon guidance factors and neuropeptides [11]. Unfortunately, there are limited studies on cervical cancer that have put forward an ideal biomarker for PNI evaluation. In this study, both tumor-derived NMB and Schwann cell-derived CCL2 could be detected in the plasma of cervical cancer patients and were clearly increased in those with PNI. Moreover, combined examination of NMB and CCL2 showed the discrimination of PNI with the AUC value of 0.914 (95% CI 0.828-1.000) in cervical cancer as compared to that of NMB or CCL2 alone, which may be clinical effective for the preoperative evaluation of PNI.

However, the molecular mechanism underlying the high transcriptional expression of NMB in cervical cancer PNI remains unknown and requires further study. Previous studies have found that NMB can be regulated by hormonal balance, including the stress-related neuroendocrine system and estrogen [20–23]. Current clinical evidences also suggest that emotions, especially chronic stress, affect the outcome of cancer development [42]. Our preliminary findings suggest that estrogen, l-glutamic acid, triiodothyronine (T3), and γ-GABA can upregulate NMB mRNA in tumor cells. The subsequent step is to investigate how these hormones regulate NMB expression via transcription factors and histone modifications, which will enhance our understanding of the heterogeneity between PNI and non-PNI patients and further establish practical and precise clinical prediction models for PNI in cervical cancer.

In summary, we propose that cervical cancer-produced neuropeptide NMB to activate Schwann cells through the NMBR-mediated calcium influx and downstream PKA activation, thus triggering Schwann cell reprogramming and then promoting axon regeneration, which in turn facilitates tumor cell invasion along the neurites by secreting CCL2. All these lead to the occurrence of PNI in cervical cancer (Fig. 7m). The combined examination of serum NMB and CCL2 may serve as diagnostic indicators to predict PNI before operation in cervical cancer patients, and help to identify whether these patients could choose NSRH surgery.

## Materials and methods

### Patients and tissues

From the clinical records and pathology reports during 2017-2021, 70 PNI-positive and 353 PNI-negative cervical cancer patients were included, and the clinicopathological information was collected. Every patient underwent radical hysterectomy according to a uniform protocol, and determined by pathological analysis. Clinical pathological sections (*n* = 112) were processed for Immunohistochemistry (IHC) and Haematoxylin and eosin (HE) staining. The normal cervical tissue samples were obtained from eight patients who had benign pathologies and surgically treated. CIN I (*n* = 22), CIN II-III (*n* = 22), CIS (*n* = 22), and cervical cancer (*n* = 38, 20 were PNI-positive, and 18 were PNI-negative) tissues were from the corresponding patients during biopsy or surgery. Human serum samples were registered and stored in the biobank. All the above samples were collected from the International Peace Maternity and Child Health Hospital (IPMCH, Shanghai, China). Research ethics approval was approved by the Ethics Committee of IPMCH, School of Medicine, Shanghai Jiaotong University, and patient consent was received in writing.

Detailed methods for the other in vivo and in vitro experiments are described in Supplementary information.

### Supplementary information

Supplementary Materials

Original Raw Data

Full and uncropped western blots

Supplementary Movie 1

Supplementary Movie 2

Supplementary Movie 3

## Data Availability

The accession number of the RNA-seq data is SRA: PRJNA1045304. All the original unprocessed gels and images, and all the original source data of figures have been deposited and available at the public research database Mendeley Data Reserved https://data.mendeley.com/datasets/9synp2x7dj/2.
