# Supplementary material for: Cervical cancer-produced neuromedin-B reprograms Schwann cells to initiate perineural invasion
Source: Cell Death Dis. 2024 Aug 30;15(8):636. doi: 10.1038/s41419-024-07030-9 (PMC11364772; doi:10.1038/s41419-024-07030-9)
Supplement: Supplementary file 1 — Supplementary Materials [file 41419_2024_7030_MOESM1_ESM.docx]

**SUPPLEMENTARY MATERIALS**

**Cervical cancer-produced Neuromedin-B reprograms Schwann cells to initiate perineural invasion**

Xiaoyan Gao^1†^, Qi Wang^1†^, Ting Huang^1†^, Chen Xu^1^, Xiaoming Yang^1^, Lin Zhang^1^, Jing Wang^1^, Linlin Yang^1^, Xuan Zheng^1^, Qiong Fan^1^, Dan Cao^1^, Lijuan Li^1^, Ting Ni^1^, Xiao Sun^1*^, Jin Hou^3*^, Yudong Wang^1, 2*^

Correspondence to: [sunxiao@shsmu.edu.cn](mailto:sunxiao@shsmu.edu.cn); [houjin@immunol.org](mailto:houjin@immunol.org); [wangyudong@shsmu.edu.cn](mailto:wangyudong@shsmu.edu.cn)

**This PDF file includes:**

Supplementary Methods

Figures. S1 to S11

Tables S1 to S2

Captions for Movies S1 to S3

**Other Supplementary Materials for this manuscript include the following:**

Movies S1 to S3

1. **Supplementary Methods**

*Reagents*

Antibodies specific to PGP9.5 (PA5-16825), TH (PA5-85167), VAChT (MA5-27662), NMB (PA5-103966) were from Invitrogen (Carlsbad, CA). Antibodies specific to NF-L (223343), CK-17 (270902) were from Abcam (Cambridge, MA). Antibodies specific to Pan-CK (4545), Nestin (4760), GFAP (3670), p-CREB (9198) were from Cell Signaling Technology (Danvers, MA). Antibody specific to NMBR (135300) was from Absin (Shanghai, China). Antibodies specific to P16-INK4A (10883-1-AP) was from Proteintech (Rosemont, IL). The Geltrex LDEV-Free Reduced Growth Factor Basement Membrane Matrix (A1413202), Fluo-4, AM (F14201), Fetal Bovine Serum (FBS, 10099141C), DMEM (11965092), and McCOY's 5A (16600082) were from Gibco (Shanghai, China). The Schwann Cell Medium (SCM,1701) was from ScienCell Research Laboratories (CA, USA). The Rapid RNA Extraction kit was from ES Science (Shanghai, China). The All-in-One First-Strand cDNA Synthesis Super Mix Kit and Green qPCR Super Mix Kit were from TransGen Biotech (Shanghai, China). TRITC-conjugated phalloidin (40734ES75), Click-iT EdU Imaging Kits (40276ES60), Crystal Violet Stain Solution (0.5%, 60506ES60), DAPI Fluoromount-G (36308ES20) were from YEASEN (Shanghai, China). Recombinant Neuromedin B (HY-P0241), Nifedipine (HY-B0284), NP118809 (HY-14462), Trimethadione (HY-A0092), KT5720 (HY-N6789), Serotonin (HY-B1473A), Dopamine (HY-B0451), Leptin Protein (HY-P7232), Meprednisone (HY-B0243), Estradiol (HY-B0141), Lecirelin (BY-P0051), Aldosterone (HY-113313), γ-Aminobutyric acid (HY-N0067), Liothyronine (HY-A0070A), L-Glutamic acid ( HY-14608) were from MedChemExpress (Shanghai, China). NMBR antagonist PD168368 (17920) was from Cayman (MI, USA).

*Cell cultures*

All the cell lines were obtained from the Cell Bank of Type Culture Collection of Chinese Academy of Sciences (Shanghai, Chinese Academy of Sciences, China). Cells were grown at 37°C in a standard incubator containing 5% CO_2_ as routine. RSC96 cells, derived from Rattus norvegicus neuronal Schwann cells, was cultured in complete Schwann Cell Medium (SCM) (ScienCell, CA, USA) supplemented with 10% fetal bovine serum (FBS) (Gibco，USA) and 1% Schwann cell growth supplement (SCGS). The human cervical cancer cell lines, including HeLa, and SiHa, as well as the normal cervical epithelium cell line HcerEpic, were cultured in Dulbecco’s modified Eagle’s medium (DMEM) (Gibco, USA). The ME180 cell line was cultured in complete McCOY's 5A medium (Gibco, USA), while the CaSki and HCC94 cells were cultured in RPMI 1640 medium (Gibco, USA). All the cell lines were confirmed to be free of mycoplasma contamination using the Mycoplasma Detection Kit (YEASEN, China). Cells were used at passages 2-7.

Rat dorsal root ganglia (DRG) were isolated from female Sprague Dawley rats weighing 150~250 g and cultured according to a protocol that had been previously published[1].

*Indirect co-cultures of Schwann cells (RSC96) and tumor cells*

Co-culture system was established using transwell inserts with 0.4 µm pores and 6-well or 24-well culture plates (Costar, Cambridge, USA). Cervical cancer cell suspensions were loaded in the upper inserts, and RSC96 cell suspensions were put into the lower compartment of the well for 48 hours to initiate the co-cultivation process. According to experimental needs, recombinant Neuromedin B (250nM, or as indicated in the figures) or NMBR antagonist PD168368 (1µM, or as indicated in the figures) were added to cell co-cultures and controls received equivalent dilution with vehicle alone. After 48 hours in co-culture, the insert was removed, and the proliferation, the changed morphology, as well as the reprogramming related gene expression of RSC96 cells were assessed as described under the relevant methods section.

*Neural Invasion In Vitro Model for Assessment of Nerve-Cancer Cell Interactions*

This in vitro model of nerve invasion was performed according to the method described by Ayala, which was originally developed to study PNI of prostate cancer cells[2]. The DRGs were embedded at the core of 2.5 µl Matrigel, and subsequently placed in confocal dishes (801001, NEST, China). Following the solidification of the Matrigel, DMEM supplemented with 10% FBS was introduced into the wells. The cervical cancer cells were then labelled with Zsgreen fluorescence (green) and uniformly replated around the DRGs at a density of 6000 cells per dish two days later.

Real-time movies of live cell were captured within two hours by using a Leica TCS SP8 AOBS confocal microscope, equipped with temperature and CO_2_ controllers, to study the dynamic interactions between DRG neurites and cervical cancer cells in 3D. Images were acquired every 2 minutes for a total duration of two hours.

Neurite outgrowth was assessed by immunostaining with PGP9.5 antibody (also called Uchl1) (PA5-16825, ThermoFisher) followed by Alexa Fluor 594-labeled secondary antibodies (Invitrogen). Quantification of nerve outgrowth was carried out using ImageJ software (NIH systems).

*In vivo model of murine sciatic nerve invasion*

Five-week-old female BALB/c nude mice were randomly divided into experimental groups. The cervical cancer cell mixture (3×10^5 cells in 3μL PBS) or PBS was microscopically injected into the left sciatic nerve at a distal site using a 10μL gas-tight Hamilton syringe (Hamilton Company, Switzerland). NMBR antagonist PD168368 (1.2 mg/Kg) was given by intraperitoneal injection everyday and the mice in parallel groups were treated with an equal volume of vehicle. The mice were then monitored on a weekly basis to assess the development of sciatic neurological deficits. The paw print analysis was conducted to measure sciatic nerve function. The sciatic nerve score was graded from 4 (normal) to 1 (paralysis) and the sciatic nerve function index (SFI) indicates the distance between the first and fifth toes of the mouse hind limbs (millimeter, mm). At the indicated time points after the procedure, the nude mice were humanely euthanized, and their sciatic nerves were dissected for use in subsequent experiments.

*Establishment of Neuromedin B (NMB) knockout and overexpression cell lines*

The Cas9 stable-expressing HeLa and ME180 cells (HeLa-Cas9 and ME180-Cas9) were established by lentiviral transduction of Cas9 coding sequence into the genome of cervical cancer cells and selected with 5μg/mL Blasticidin. Cas9 expression was confirmed by qPCR. Then guide RNA (sgRNA) targeting cording sequence of NMB was incorporated into the lentiviral expression vector for sgRNA (lentiCRISPR) and HeLa/ME180-Cas9 cells were transfected with NMB-sgRNA and 5μg/mL of Polybrene. After 48 hours of viral transduction, the cells were selected by 2μg/ml puromycin until uninfected cells were eliminated. The plasmids carrying Cas9 and sgRNA were lentiCas9-Blast and lentiGuide-Puro. All the sgRNA sequences targeting NMB were listed as follows: sgNMB#1: 5’-GCG GGG GGC GCT CGG ATG TT-3’, sgNMB#2: 5’-TCC GGG AGA TCC CAG CTG AG-3’, sgNMB#3: 5’-GCA CTC GCG AGG CAA CCT CT-3’. The negative control sgRNA (sgNC) sequence was 5’-ACG GAG GCT AAG CGT CGC AA-3’. To amplify the sgRNA targeting region, primers were designed as follows:

Primer-NC-T: CACCGACGGAGGCTAAGCGTCGCAA,

Primer-NC-B: AAACTTGCGACGCTTAGCCTCCGTC,

Primer-T1: CACCGCGGGGGGCGCTCGGATGTT,

Primer-B1: AAACAACATCCGAGCGCCCCCCGC,

Primer-T2: CACCGTCCGGGAGATCCCAGCTGAG,

Primer-B2: AAACCTCAGCTGGGATCTCCCGGAC,

Primer-T3: CACCGCACTCGCGAGGCAACCTCT,

Primer-B3: AAACAGAGGTTGCCTCGCGAGTGC.

We performed Sanger sequencing on sgRNA inserts using the standard Hu6-F primers. Finally, gene knockout efficiency was examined by Western Blotting. In this study, genomic editing was performed by Genomeditech (Shanghai, China).

NMB overexpression lentivirus (NMB OE) was constructed by Genomeditech (Shanghai, China) with PGMLV-CMV-MCS-3×Flag-EF1-ZsGreen1-T2A-Puro vector. Virus titer was approximately 1 ×10E8 TU/ml and the transduction was conducted at a multiplicity of infection (MOI) ranging from 10 to 30 for cervical cancer cells. NMB OE cervical cancer cells were selected with 1μg/ml puromycin for 2 weeks. NMB OE was verified by Western blot.

*RNA interference*

Three duplex oligonucleotides encoding the small interfering (siRNA) designed against human NMB sequences and a negative control siRNA (siNC) were purchased from Genomeditech (Shanghai, China). The siRNA target sequences were as follows: siNMBR#1: 5’-AGU UGG UAA UCC GCU GUG UGA TT-3’; siNMBR#2: 5’-AGC AGU UUC ACA GCA UGC AUA TT-3’; siNMBR#3: 5’-AAG AAU GUG GUG ACC AAU UCU TT-3’. siNC: 5’-UUCUCCGAACGUGUCACGUATdT-3’. RSC96 Cells were transfected with 50nM siRNA using Lipofectamine 3000 (ThermoFisher Scientific, USA) simultaneously in 1 mL Opti-MEM Reduced Serum Medium according to the manufacturer’s instructions.

*3D invasion assay*

A total of 2,800 green fluorescent HeLa cells, ME180 cells, and HcerEpic cells were introduced into a 2-chamber insert (ibidi, Germany), containing 40μl of Matrigel matrix. Following a 6-day growth period in DMEM supplemented with 10% FBS, 50,000 red fluorescent RSC96 cells were introduced onto the top of the Matrigel. The combined cell culture was then incubated for an additional 72 hours. The invasion distance of RSC96 cells was visualized using a Leica TCS SP8 AOBS confocal microscope with a 2-μm step. Subsequently, the image files were imported into Imaris software (Bitplane) to quantify the traversed distance of invading RSC96 cells. Specifically, the three longest distances from each of the four quadrants were meticulously recorded, and subsequently, the averages were calculated.

*Immunohistochemistry*

All the forementioned tissues were embedded in paraffin and sliced into sections measuring 4μM. Haematoxylin and eosin (HE) staining were performed following a standardized procedure to visualize tumor and nerve pathology. Immunohistochemical (IHC) staining examined the expression of PGP9.5, NF-L, TH, VAChT, NMB, and NMBR. Images were acquired and scanned by a NanoZoomer®S360 (Hamamatsu Photonics).

*Immunofluorescence assay and confocal microscopy*

The cells and tissues were fixed in 4% paraformaldehyde/PBS solution at room temperature for a duration of one hour, and the slices were deparaffinized and hydrated. Subsequently, the samples were permeabilized with 0.3% Triton X-100 for two hours and blocked in 5% BSA solution or 10% newborn calf serum (NCS) for another two hours at room temperature. Primary antibodies diluted in 10% NCG/PBS were incubated overnight at 4°C. Signal detection was performed using appropriate Alexa Fluor fluorescent secondary antibodies (Invitrogen). All samples utilized in the immunofluorescence assay were mounted using DAPI Fluoromount-G™ (YEASEN, China) and stored in a light-protected environment. The Leica inverted fluorescence confocal microscope was used to capture the confocal images and quantified manually the acquired images with ImageJ software.

*ELISA assay*

A total of 46 human serum samples were included. ELISA was conducted in adherence to the manufacturer’s instructions. The levels of human/mouse serum NMB, CCL2, and cAMP were quantified using ELISA kits procured from MultiSciences Biotech (Shanghai, China) and Elabscience Biotechnology (Wuhan, China) according to the supplier’s instructions.

*RNA extraction and real-time quantitative PCR (qRT-PCR)*

The isolation of total RNA was performed using a rapid RNA extraction kit (ES Science, China), followed by reverse transcription using the All-in-One First-Strand cDNA Synthesis Super Mix Kit (TransGen Biotech, China). The evaluation of RNA expression was conducted through qPCR utilizing the Green qPCR Super Mix (TransGen Biotech, China). The qPCR primers for gene expression analysis were human NMB (Forward: 5’-CGCACCCCAAATCCAGTACA-3’, Reverse: 5’-TCAACAGGGTCCCATTCAGC-3’); human NMBR (Forward: 5’-GTTTCTGTGTTCACTCTCAC-3’, Reverse: 5’-ATCCAAGCTACCAATGCGTG-3’); human GADD45B (Forward: 5’-GCCCTGCAAATCCACTTCAC-3’, Reverse: 5’-GTGTGAGGGTTCGTGACCA-3’); human TGFB1 (Forward: 5’-GGAAATTGAGGGCTTTCGCC-3’, Reverse: 5’-CCGGTAGTGAACCCGTTGAT-3’); human SERPINE1 (Forward: 5’-CCGCCTCTTCCACAAATCAG-3’, Reverse: 5’-AATGTTGGTGAGGGCAGAGA-3’); human TMEM158 (Forward: 5’-TGTGCTTCGTGCTGTAGTTATC-3’, Reverse : 5’-TCAGTCCAAGGGCTTAAACATC-3’); human fosl1 (Forward: 5’-GGAGGAAGGAACTGACCGACTT-3’, Reverse: 5’-CTCTAGGCGCTCCTTCTGCTTC-3’); human ITGA5 (Forward: 5’-GCTTCAACTTAGACGCGGAG-3’,Reverse: 5’-GCACACTGACCCCGTCTG-3’); human loxl2 (Forward: 5’-GGGTGGAGGTGTACTATGATGG-3’, Reverse: 5’-CTTGCCGTAGGAGGAGCTG-3’); human GDF15 (Forward: 5’-CAACCAGAGCTGGGAAGATTCG-3’, Reverse: 5’-CCCGAGAGATACGCAGGTGCA-3’); human ECM1 (Forward: 5’-GCTTCACGGCTACAGGACAG-3’, Reverse: 5’-GAGGCTTCGGGATAGGGGT-3’); human ANGPTL4 (Forward: 5’-GTCCACCGACCTCCCGTTA-3’, Reverse: 5’-CCTCATGGTCTAGGTGCTTGT-3’); human MT1X (Forward: 5’-TTGATCGGGAACTCCTGCTT-3’, Reverse: 5’-CAGGAGCCAACAGGCGA-3’); human Cas9 (Forward: 5’-CTACGACGACGACCTGGACAA-3’, Reverse: 5’-GCCTTGGTGATCTCGGTGTTC-3’); internal control 18S (Forward: 5’-GTAACCCGTTGAACCCCATT-3’, Reverse: 5’-CCATCCAATCGGTAGTAGCG-3’); mouse CCL2 (Forward: 5’-ACCACTATGCAGGTCTCTGTCA-3’, Reverse: 5’-GGCATTAACTGCATCTGGCTGA-3’); mouse GDNF (Forward: 5’-GCTGACCAGTGACTCCAATATGC-3’, Reverse: 5’-CCTCTGCGACCTTTCCCTCTG-3’); mouse BDNF (Forward: 5’- CGATTAGGTGGCTTCATAGGAGAC-3’, Reverse: 5’-CAGAACAGAACAGAACAGAACAGG-3’); mouse cJUN (Forward: 5’- TGAAGTGACCGACTGTTCTAT-3’, Reverse: 5’-CTTAGGGTTACTGTAGCCGTAG-3’); mouse MBP (Forward: 5’-GAGAACCCCAGTCTAATAATGTCC-3’, Reverse: 5’-AACCATCACCTGCCTTCCT-3’); mouse GFAP (Forward: 5’- GATCCGAGAAACCAGCCTGGAC-3’, Reverse: 5’-TGGGCACACCTCACATCACAT-3’); mouse NGFR (Forward: 5’-CACCGACAACCTCATTCCTGTC-3’, Reverse: 5’-TTGTTTGCAGCTGTTCCACCTC-3’); mouse NES (Forward: 5’- AGGGCGTGGAACAGAGATT-3’, Reverse: 5’-GCCTCCAGCAGAGTCCTGTA-3’); mouse GAP43 (Forward: 5’-GGCCGCAACCAAAATTCAGG-3’,Reverse: 5’-CGGCAGTAGTGGTGCCTTC-3’).The relative expression of the target genes was normalized to that of the 18S internal control using 2 -ΔΔCt cycle threshold method.

*Western Blotting (WB)*

The cells and tissues were lysed using RIPA Lysis Buffer (Epizyme, China) supplemented with phosphatase inhibitor cocktail (100×) and the protease inhibitor cocktail (100×, EDTA-free). After three rounds of ultrasonication (5 seconds each) on ice, the samples were collected and stored at -80°C. Lysates were quantified and equal amounts of total proteins were fractionated with SDS-PAGE and then transferred onto 0.22/0.45μm polyvinylidene fluoride (PVDF) membranes (Millipore, USA). The membranes were blocked in Protein Free Rapid Blocking Buffer (Epizyme, China) per the manufacturer’s instructions. GAPDH was used as a loading control. Signal detection was conducted using a Leica imaging system. Protein levels were quantified using Image J and normalized to the internal control GAPDH.

*5-Ethynyl-2’-deoxyuridine (EDU) incorporation assay*

The Yefluor 594 EdU Imaging Kit (YEASEN, China) was utilized to conduct EdU proliferation assays according the guidelines provided by the manufacturer. Nucleic acids were labelled using DAPI (YEASEN, China), and visualized through a 405 nm excitation wavelength. The Leica inverted fluorescence microscope was employed to capture photographs. The ratio of EdU-positive cells to the total number of DAPI-positive cells was computed as an indicative measure of the extent of EdU incorporation.

*Staining of actin filaments with phalloidin*

The morphological changes in the cytoskeleton and microfilaments of RSC96 cells were examined using the FITC-phalloidin F-actin staining technique as we described previously[3]. In brief, the cells (50% confluence) were incubated in 100nM TRITC-conjugated phalloidin for 30 minutes at ambient temperature in the absence of light. And a fluorescence mounting medium with anti-fade properties, incorporating4′,6-diamidino-2-phenylindole (DAPI), was employed to provide a complementary staining of the cells.

*Invasion and migration assay*

A transparent polyethylene terephthalate (PET) membrane with a pore size of 8.0 µm (Corning, USA) was utilized in 24-well plates. For the invasion assay, 50 µl Matrigel coating at a 1:3 dilution was applied. The upper insert contained 1×10^5^ RSC96 cells cultured in 0.2 mL DMEM without serum, while the lower compartment contained cancer cells in 0.5 mL complete culture medium. Subsequently, following a period of 36 hours for the migration assay and 48 hours for the invasion assay, the cells that had invaded the upper insert were immobilized and stained with Crystal Violet Stain Solution (0.5%) (YEASEN, China). Five arbitrary fields of membranes were captured and the number of cells was determined and averaged.

*Intracellular Ca^2+^ imaging*

Cells were seeded in 96-well confocal microscope plates at a density of 3,000 cells per well, treated with 2.5μM Fluo-4 acetoxymethyl ester (Fluo-4 a.m., molecular probes) (ThermoFisher, USA), and incubated in the dark for 60 minutes. Fluo-4 fluorescence was induced using Argon lasers operating at a wavelength of 488 nm. A baseline measurement was initially obtained, followed by the addition of the corresponding reagent to the well. Subsequently, transient Ca^2+^ fluxes were continuously monitored for 2 minutes using a Leica confocal laser-scanning microscope, with a frame rate of one per second. The resulting data was then analyzed using time series techniques in the ImageJ software.

*Statistics*

All experiments were repeated at least 3 times in a triplicate manner.Statistical analysis was conducted using GraphPad Prism software 9.0 or SPSS 20.0. Significance was determined using either Student’s t-test or one way ANOVA with Tukey post hoc test. A significance probability below 0.05 was deemed statistically for all tests. Adobe illustrator software was used for graphing.

*Data availability*

The TCGA database (https://portal.gdc.com) was utilized to acquire TCGA datasets encompassing RNA-seq expression profiles and associated clinical information for cervical cancer. The GTEx data was obtained from the GTEx data portal (https://www.gtexportal.org/home/datasets). It should be noted that protected patient information, governed by privacy and ethical constraints, is not publicly accessible in this study. However, interested parties may request access to this information from the corresponding authors.

**References**

1. Enes J, Langwieser N, Ruschel J, Carballosa-Gonzalez MM, Klug A, Traut MH, et al. Electrical activity suppresses axon growth through Ca(v)1.2 channels in adult primary sensory neurons. Curr Biol. 2010;20:1154-64.

2. Ayala GE, Wheeler TM, Shine HD, Schmelz M, Frolov A, Chakraborty S, et al. In vitro dorsal root ganglia and human prostate cell line interaction: redefining perineural invasion in prostate cancer. Prostate. 2001;49:213-23.

3. Huang T, Fan Q, Wang Y, Cui Y, Wang Z, Yang L, et al. Schwann Cell-Derived CCL2 Promotes the Perineural Invasion of Cervical Cancer. Front Oncol. 2020;10:19.

1. **Supplementary Figures and legends**


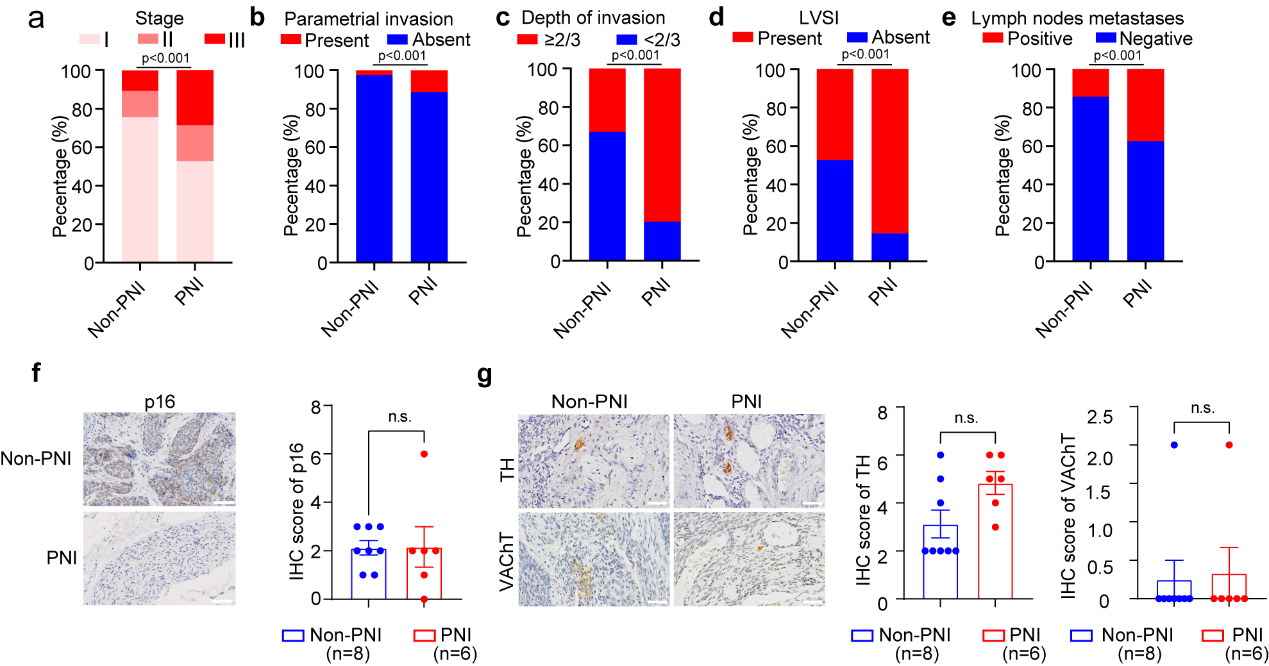


**Fig. S1** **Associations between PNI and established risk factors for poor outcome in cervical cancer.**

**a** The proportion of the clinical stage was analyzed in cervical cancer patients with (n=70) or without (n=353) PNI (chi-square test).

**b** The incidence of parametrial invasion was analyzed in cervical cancer patients with (n=70) or without (n=353) PNI (chi-square test).

**c** The depth of invasion was analyzed in cervical cancer patients with (n=70) or without (n=353) PNI (chi-square test).

**d** The incidence of LVSI was analyzed in cervical cancer patients with (n=70) or without (n=353) PNI (chi-square test).

**e** The incidence of lymph node metastases was analyzed in cervical cancer patients with (n=70) or without (n=353) PNI (chi-square test).

**f** The infection of hrHPV was analyzed by p16 staining. Scale bars: 100 μm. Relative IHC score was analyzed using unpaired t-test (non-PNI, n=8; PNI, n=6).

**g** Nerve density was analyzed by tyrosine kinase (TH) and vesicular acetylcholine transporter (VAChT) staining. Scale bars: 50 μm. Relative IHC score was analyzed using unpaired t-test (non-PNI, n=8; PNI, n=6).

Data are shown as photographs from one representative of three independent experiments. *P < 0.05, **P < 0.01, *** P <0.001, **** P <0.0001.


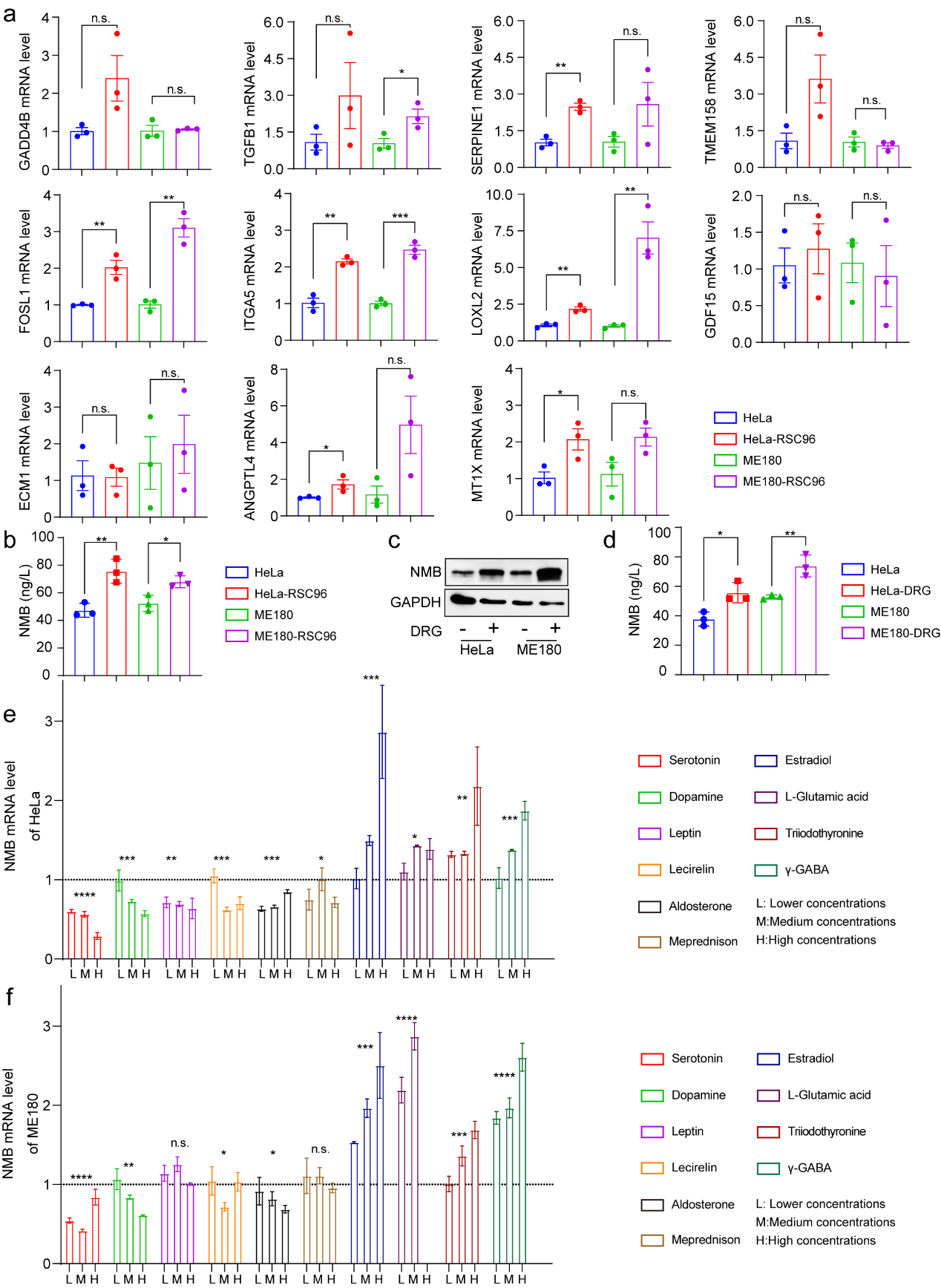


**Fig. S2** **Cervical cancer-produced NMB triggers PNI.**

**a** Genes screened out by RNA-seq mRNA expression of tumor cells co-cultured with DRG were examined by qRT-PCR (n=3, one-way ANOVA and Tukey's multiple comparisons test).

**b** Quantification of secreted NMB, by ELISA assay, in cervical cancer cells cocultured with or without RSC96 cells (n=3, t test).

**c** NMB expression in cervical cancer cells co-cultured with DRG was confirmed by Western blot.

**d** Quantification of secreted NMB, by ELISA assay, in cervical cancer cells cocultured with or without DRG (n=3, t test).

**e,f** NMB mRNA expression of tumor cells treated with 9 chronic stress related hormones, estrogen, and hypoxia were examined by qRT-PCR (n=3, one-way ANOVA and Tukey's multiple comparisons test). L: Lower concentrations, M: Medium concentrations, H: High concentrations. For ME180 treated with L-Glutamic acid, high concentration (20mM) become too high to survive, therefore only the low and medium concentration data shown.

Data are shown as photographs from one representative of three independent experiments. *P < 0.05, **P < 0.01, *** P <0.001, **** P <0.0001.


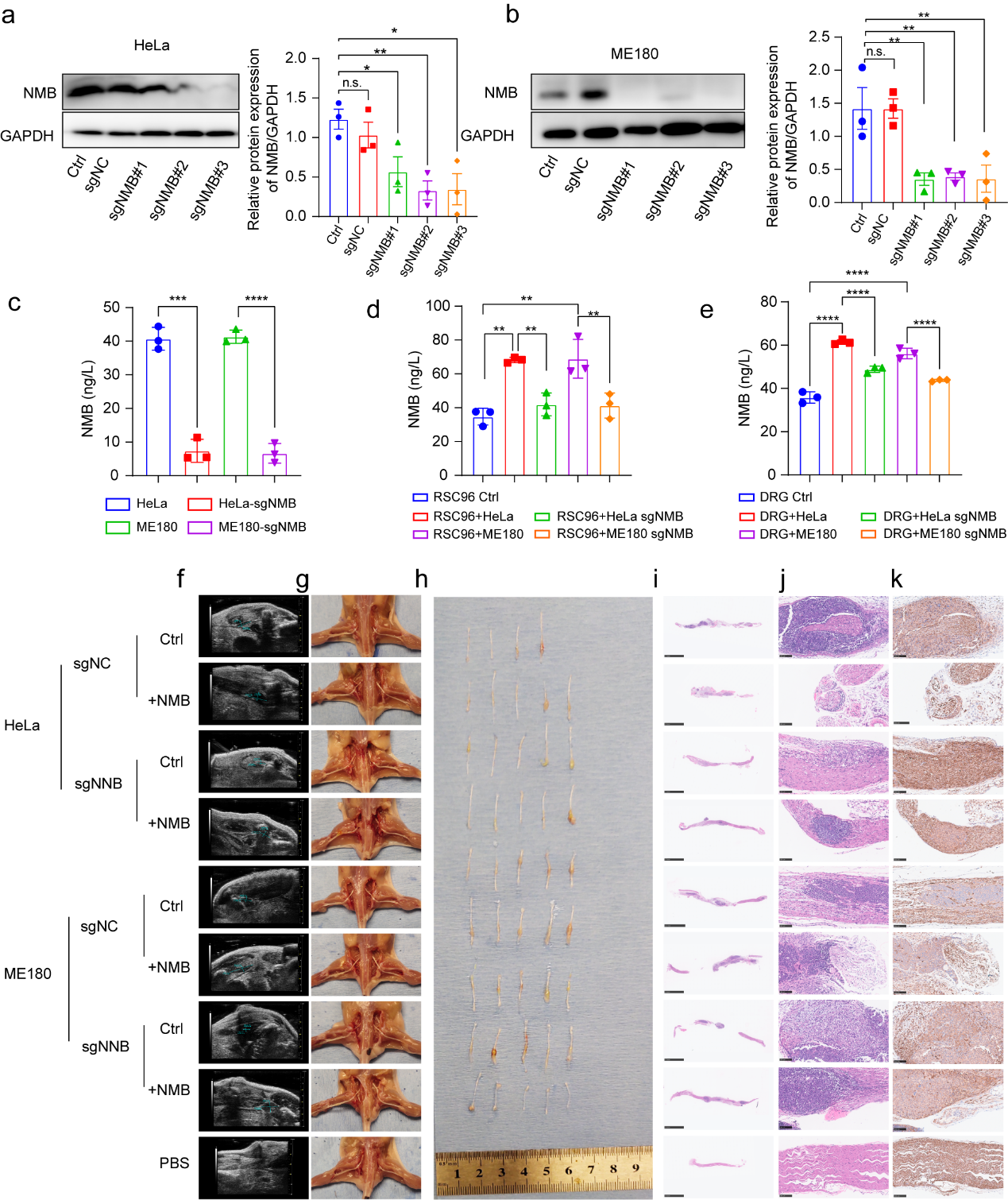


**Fig S3. Knockout of NMB expression suppresses PNI in vivo three days after tumor injection.**

**a**,**b** Quantified NMB protein level in NMB knockout HeLa (**a**) and ME180 (**b**) cells by CRISPR-Cas9 system were shown (n=3, one-way ANOVA and Tukey's multiple comparisons test).

**c** Quantification of secreted NMB, by ELISA assay, in NMB knockout cervical cancer cells (n=3, t test).

**d,e** Quantification of secreted NMB, by ELISA assay, in RSC96 cells (**d**) and DRG (**e**) co-cultured with NMB knockout HeLa or ME180 cells (n=3, one-way ANOVA and Tukey's multiple comparisons test).

**f**-**k** The influences of NMB knockout in perineural invasion three days after tumor cell injection were shown. The images from left to right represented the high-resolution ultrasound image (**f**), anatomical image (**k**), macroscopic images (**h**), HE staining (**i**, Left column:12.5×magnification, scale bars: 2.5mm; **j**, Right column: 200×magnification, scale bars: 100μm), PGP9.5 staining (**k**, 200×magnification, scale bars: 100μm).

Data are shown as photographs from one representative of three independent experiments. *P < 0.05, **P < 0.01, *** P <0.001, **** P <0.0001.


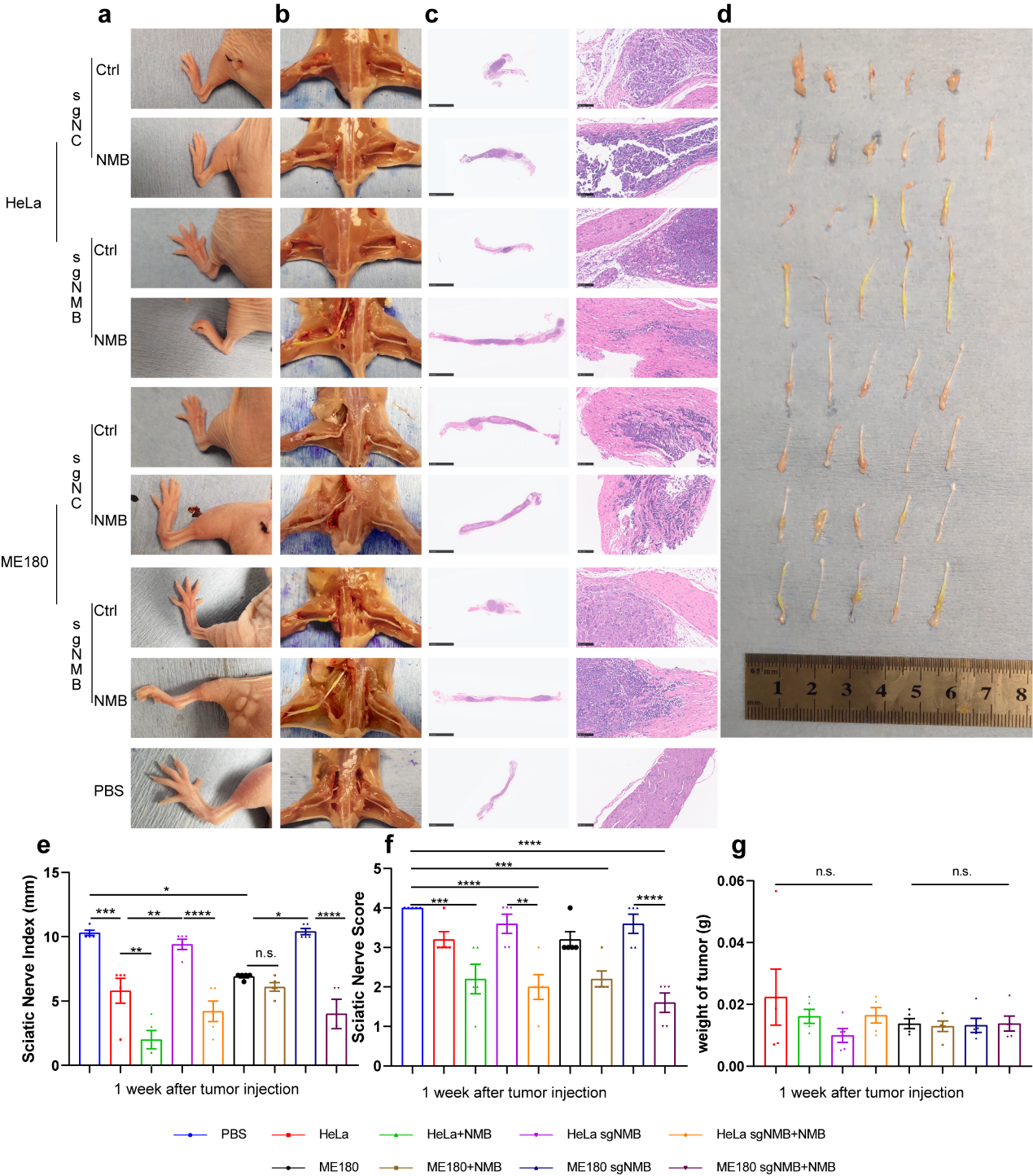


**Fig. S4 Knockout of NMB expression suppresses PNI in vivo one week after tumor injection.**

**a** Representative images of mice one week after tumor injection revealing left hind limb function in the normal PBS, sgNC, and sgNMB cervical cancer cells with/without recombinant NMB groups were shown (n=5).

**b** In situ images of the sciatic nerve injected with PBS, sgNC, and sgNMB cervical cancer cells with/without recombinant NMB groups were shown (n=5).

**c** HE staining was analyzed in cancer cells invasion along the nerve in the murine model (n=5). Left column:12.5×magnification, scale bars: 2.5 mm; Right column: 200×magnification, scale bars: 100 μm.

**d** Macroscopic images of the PNI mice model result from the groups in (**b**) were shown (n=5).

**e** Quantification of SFI between different groups at one week after tumor implantation was shown (n=5, one-way ANOVA and Tukey's multiple comparisons test).

**f** Quantification of nerve function scores between different groups at one week after tumor implantation was shown (n=5, one-way ANOVA and Tukey's multiple comparisons test).

**g** Quantification of tumor weight between different groups at one week after tumor implantation was shown (n=5, one-way ANOVA and Tukey's multiple comparisons test).

Data are shown as the photographs from one representative of three independent experiments. *P < 0.05, **P < 0.01, *** P <0.001, **** P <0.0001.


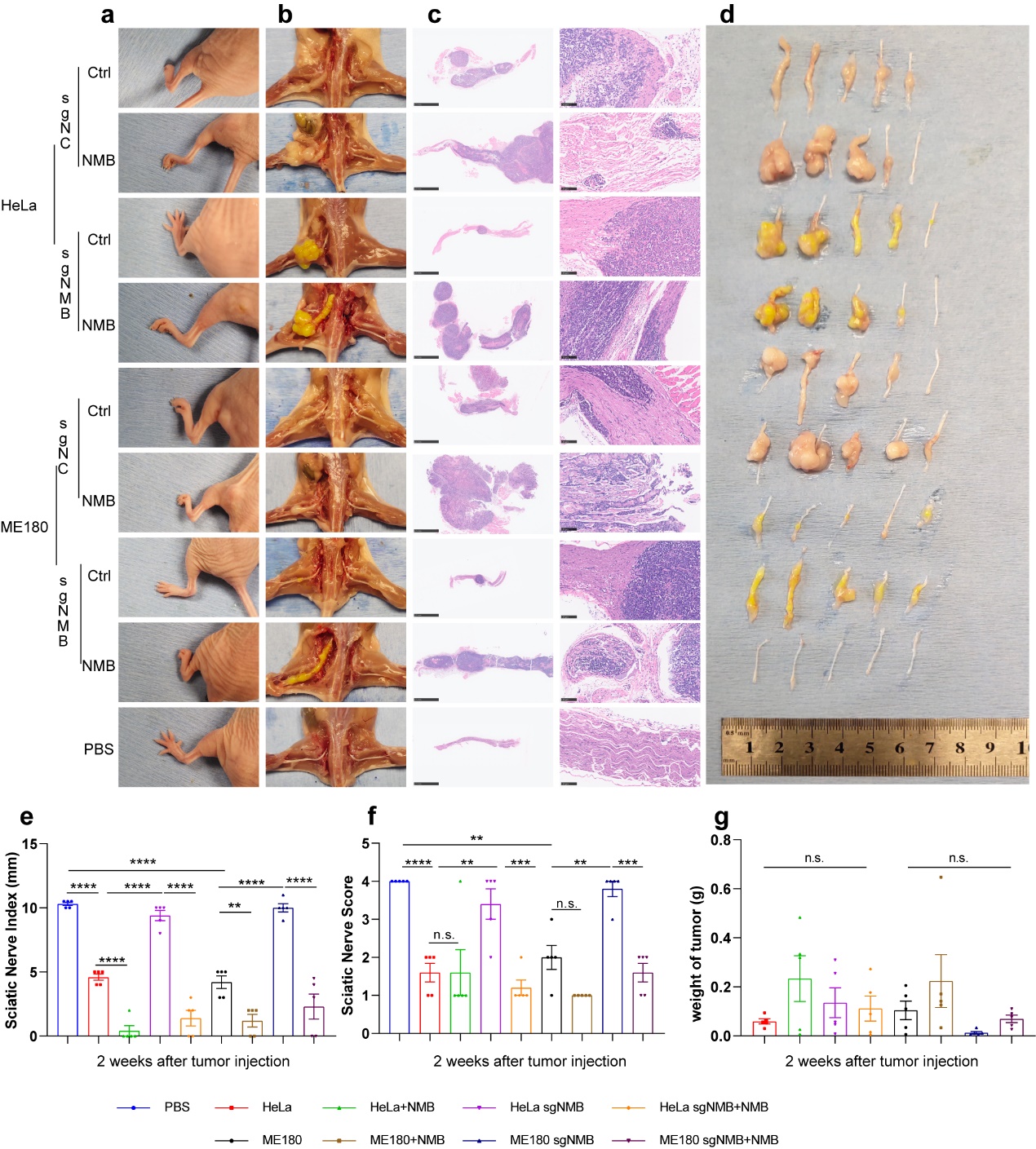


**Fig. S5 Knockout of NMB expression suppresses PNI in vivo two weeks after tumor injection.**

**a** Representative images of mice two weeks after tumor injection revealing left hind limb function in the normal PBS, sgNC, and sgNMB cervical cancer cells with/without recombinant NMB groups were shown (n=5).

**b** In situ images of the sciatic nerve injected with PBS, sgNC, and sgNMB cervical cancer cells with/without recombinant NMB groups were shown (n=5).

**c** HE staining was analyzed in cancer cells invasion along the nerve in the murine model (n=5). Left column:12.5×magnification, scale bars: 2.5 mm; Right column: 200×magnification, scale bars: 100 μm.

**d** Macroscopic images of the PNI mice model result from the groups in (**b**) were shown (n=5).

**e** Quantification of SFI between different groups at two weeks after tumor implantation was shown (n=5, one-way ANOVA and Tukey's multiple comparisons test).

**f** Quantification of nerve function scores between different groups at two weeks after tumor implantation was shown (n=5, one-way ANOVA and Tukey's multiple comparisons test).

**g** Quantification of tumor weight between different groups at two weeks after tumor implantation was shown (n=5, one-way ANOVA and Tukey's multiple comparisons test).

Data are shown as the photographs from one representative of three independent experiments. *P < 0.05, **P < 0.01, *** P <0.001, **** P <0.0001.


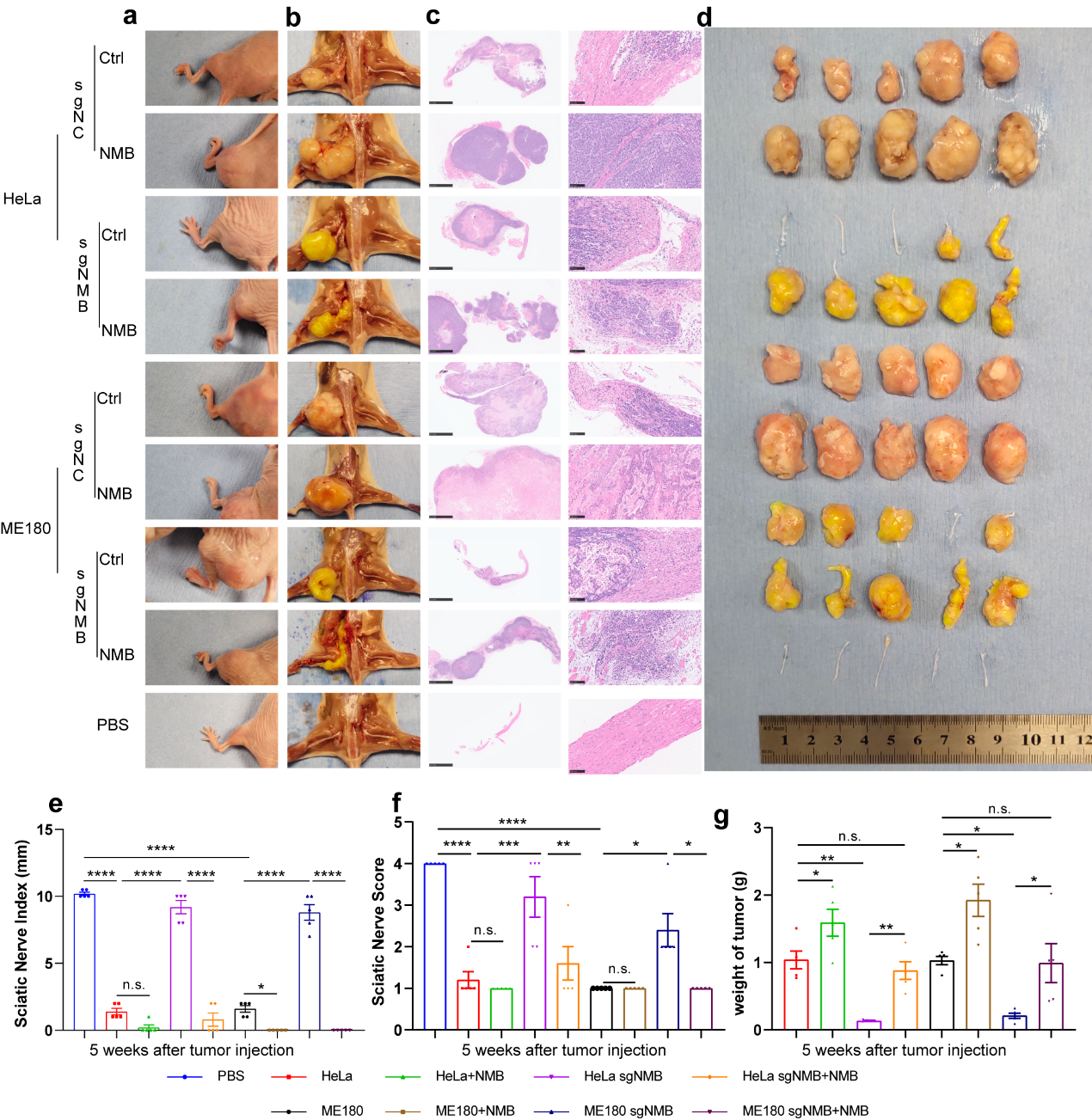


**Fig. S6 Knockout of NMB expression suppresses PNI in vivo five weeks after tumor injection.**

**a** Representative images of mice five weeks after tumor injection revealing left hind limb function in the normal PBS, sgNC, and sgNMB cervical cancer cells with/without recombinant NMB groups were shown (n=5).

**b** In situ images of the sciatic nerve injected with PBS, sgNC, and sgNMB cervical cancer cells with/without recombinant NMB groups were shown (n=5).

**c** HE staining was analyzed in cancer cells invasion along the nerve in the murine model (n=5). Left column:12.5×magnification, scale bars: 2.5 mm; Right column: 200×magnification, scale bars: 100 μm.

**d** Macroscopic images of the PNI mice model result from the groups in (**b**) were shown (n=5).

**e** Quantification of SFI between different groups at five weeks after tumor implantation was shown (n=5, one-way ANOVA and Tukey's multiple comparisons test).

**f** Quantification of nerve function scores between different groups at five weeks after tumor implantation was shown (n=5, one-way ANOVA and Tukey's multiple comparisons test).

**g** Quantification of tumor weight between different groups at five weeks after tumor implantation was shown (n=5, one-way ANOVA and Tukey's multiple comparisons test).

Data are shown as the photographs from one representative of three independent experiments. *P < 0.05, **P < 0.01, *** P <0.001, **** P <0.0001.


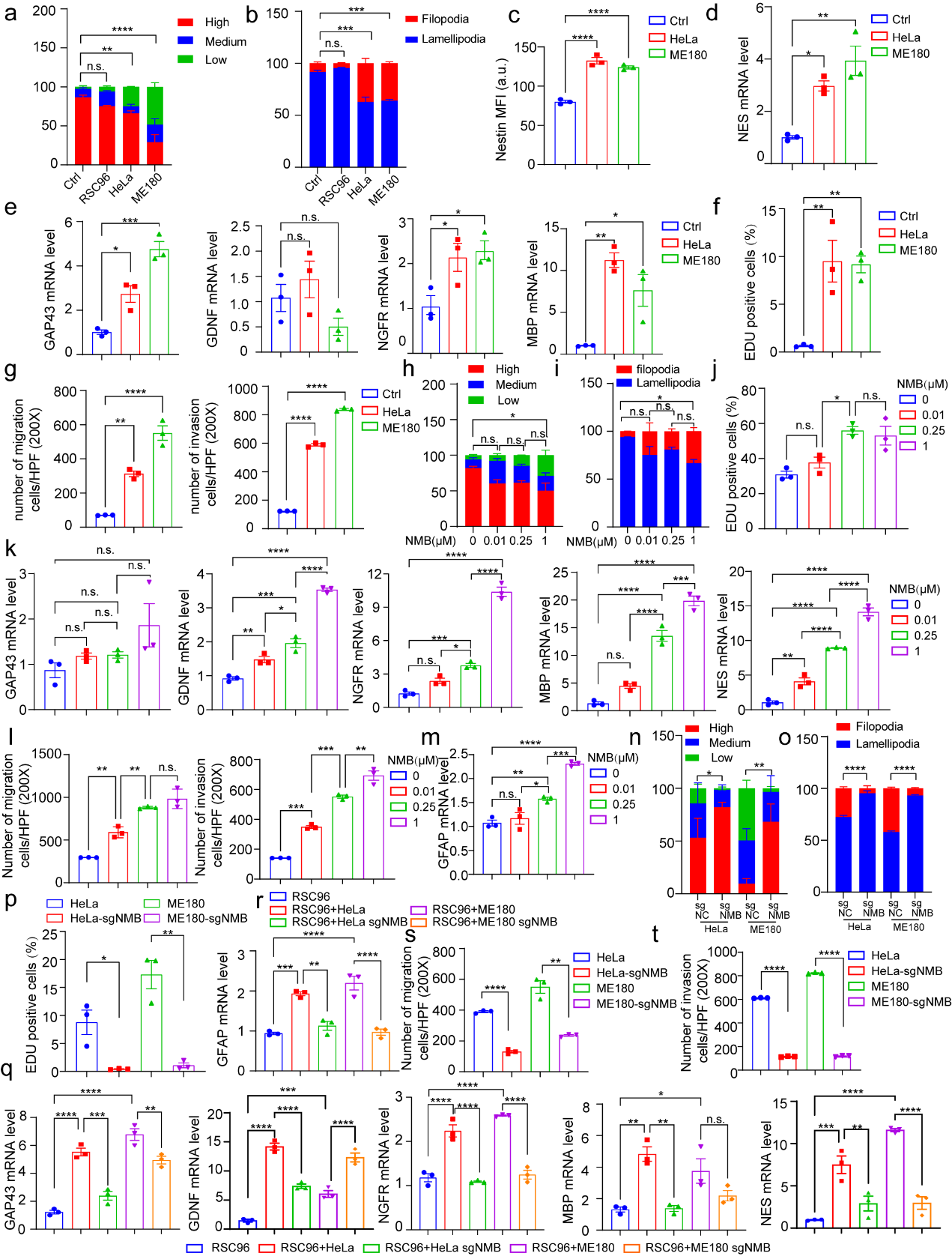


**Fig. S7 Cervical cancer-produced NMB reprograms and activates Schwann cells.**

**a** Percentages of cells with low (green), medium (blue), or high (red) numbers of focal adhesion points were analyzed in RSC96 cells treated with supernatant from RSC96 or HeLa or ME180 cells (n=3, chi-square test).

**b** Percentages of cells characterized by lamellipodium (blue) and filopodia (red) protrusions were analyzed in RSC96 cells treated with supernatant from RSC96 or HeLa or ME180 cells (n=3, chi-square test).

**c** MFI of Nestin was analyzed in RSC96 cells co-cultured with HeLa and ME180 cells (n=3, one-way ANOVA and Tukey's multiple comparisons test).

**d,e** NES (**d**), GAP43, GDNF, NGFR, and MBP **(e)** mRNA expression of RSC96 cells co-cultured with HeLa and ME180 cells were examined by qRT-PCR (n=3, one-way ANOVA and Tukey's multiple comparisons test).

**f** Quantification of EDU-positive cells was analyzed in RSC96 cells co-cultured with HeLa and ME180 cells (n=3, one-way ANOVA and Tukey's multiple comparisons test).

**g** Quantification of migration and invasion cells was analyzed in RSC96 cells co-cultured with HeLa and ME180 cells (n=3, one-way ANOVA and Tukey's multiple comparisons test).

**h** Percentages of cells with low (green), medium (blue), or high (red) numbers of focal adhesion points were analyzed in RSC96 cells treated with NMB (n=3, chi-square test).

**i** Percentages of cells characterized by lamellipodium (blue) and filopodia (red) protrusions were analyzed in RSC96 cells treated with NMB (n=3, chi-square test).

**j** Quantification of EDU-positive cells was analyzed in RSC96 cells treated with NMB (n=3, one-way ANOVA and Tukey's multiple comparisons test).

**k** GAP43, GDNF, NGFR, MBP, and MBP mRNA expression of RSC96 cells treated with NMB were examined by qRT-PCR (n=3, one-way ANOVA and Tukey's multiple comparisons test).

**l** Quantification of migration and invasion cells was analyzed in RSC96 cells treated with NMB (n=3, one-way ANOVA and Tukey's multiple comparisons test).

**m** GFAP mRNA expression of RSC96 cells treated with NMB were examined by qRT-PCR (n=3, one-way ANOVA and Tukey's multiple comparisons test).

**n** Percentages of cells with low (green), medium (blue), or high (red) numbers of focal adhesion points were analyzed in RSC96 cells co-cultured with NMB knockout HeLa or ME180 cells (n=3, chi-square test).

**o** Percentage of cells characterized by lamellipodium (blue) and filopodia (red) protrusions were analyzed in RSC96 cells co-cultured with NMB knockout HeLa or ME180 cells (n=3, chi-square test).

**p** Quantification of EDU-positive cells was analyzed in RSC96 cells co-cultured with NMB knockout HeLa or ME180 cells (n=3, one-way ANOVA and Tukey's multiple comparisons test).

**q** GAP43, GDNF, NGFR, MBP, and NES mRNA expression of RSC96 cells co-cultured with NMB knockout HeLa or ME180 cells were examined by qRT-PCR (n=3, one-way ANOVA and Tukey's multiple comparisons test).

**r** GFAP mRNA expression of RSC96 cells co-cultured with NMB knockout HeLa or ME180 cells were examined by qRT-PCR (n=3, one-way ANOVA and Tukey's multiple comparisons test).

**s,t** Quantification of migration and invasion cells was analyzed in RSC96 cells co-cultured with NMB knockout HeLa or ME180 cells (n=3, one-way ANOVA and Tukey's multiple comparisons test).

*P < 0.05, **P < 0.01, *** P <0.001, **** P <0.0001.


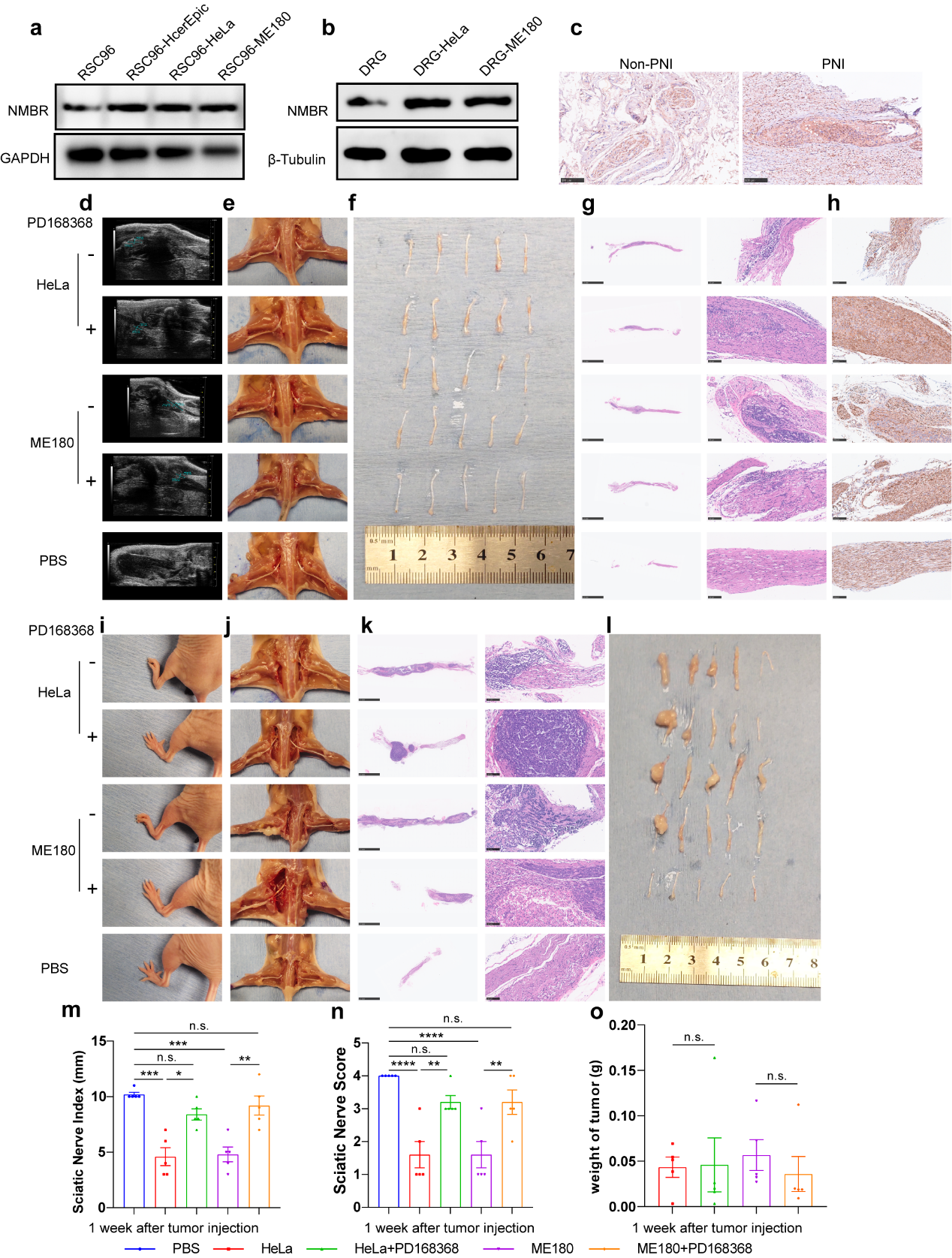


**Fig. S8 NMBR mediates the NMB-triggered reprogramming of Schwann cells.**

**a** NMBR expression in RSC96 cells co-cultured with HcerEpic, HeLa, and ME180 was examined by Western blot.

**b** NMBR expression in DRG cells co-cultured with HeLa and ME180 was examined by Western blot.

**c** NMBR staining was analyzed in cervical cancer patients with PNI (n=20) and without PNI (n=18).

**d-h** The influences of NMBR antagonist PD168368 in perineural invasion three days after tumor cell injection were shown. The images from left to right represented the high-resolution ultrasound image (**d**), anatomical image (**e**), macroscopic images (**f**), HE staining (**g**, Left column:12.5×magnification, scale bars: 2.5 mm; Right column: 200×magnification, scale bars: 100 μm), PGP9.5 staining (**h**, 200×magnification, scale bars: 100 μm).

**i-l** The influences of NMBR antagonist PD168368 in perineural invasion one week after tumor injection were shown. Representative images of mice one week after tumor injection revealing improved left hind limb paralysis in PD168368 groups were shown (n=5) (**i**). In situ images (**j**), HE staining (**k**, Left column:12.5×magnification, scale bars: 2.5 mm; Right column: 200×magnification, scale bars: 100 μm), and macroscopic images (**l**) of the PNI mice model result from the groups in (**i**) were shown.

**m-o** Quantification of SFI (**m**), mean left sciatic nerve function scores (**n**), and the tumor weight (**o**) of different groups at one weeks after tumor injection were shown (n=5, one-way ANOVA and Tukey's multiple comparisons test).

Data are shown as the photographs from one representative of three independent experiments. *P < 0.05, **P < 0.01, *** P <0.001, **** P <0.0001.


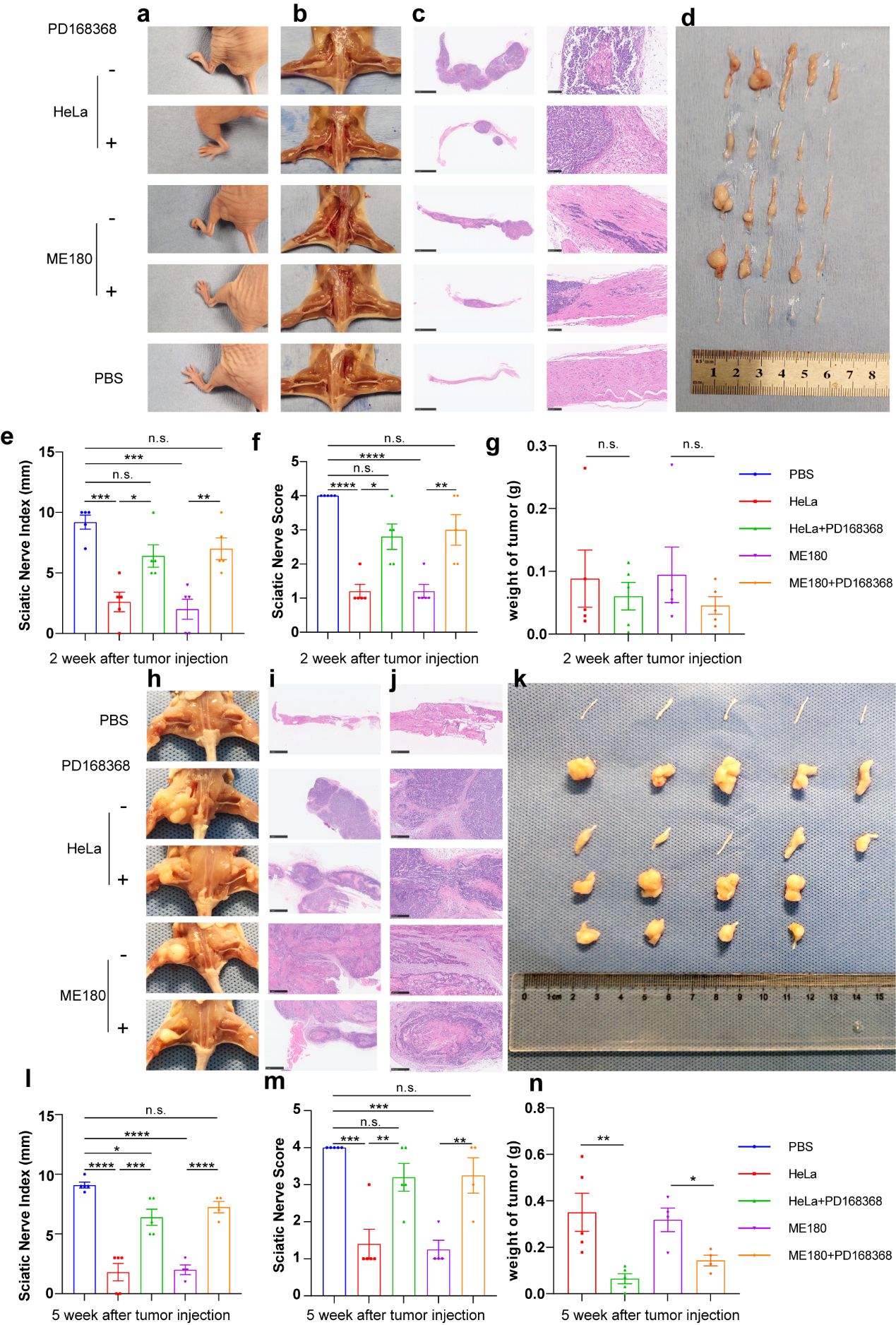


**Fig. S9 In vivo administration of NMBR antagonist PD168368 promotes sciatic nerve functional recovery after cervical cancer induced injury.**

**a** Representative images of mice two weeks after tumor injection revealing improved left hind limb paralysis in PD168368 groups were shown (n=5).

**b-d** In situ images (**b**), HE staining (**c**, Left column:12.5×magnification, scale bars: 2.5 mm; Right column: 200×magnification, scale bars: 100 μm), Macroscopic images (**d**) of the PNI mice model result from the groups in (b) were shown (n=5).

**e** Quantification of SFI in PNI mice treated with or without PD168368 two weeks after tumor implantation was shown (n=5, one-way ANOVA and Tukey's multiple comparisons test).

**f** Quantification of sciatic nerve function scores in PNI mice treated with or without PD168368 two weeks after tumor implantation was shown (n=5, one-way ANOVA and Tukey's multiple comparisons test).

**g** Quantification of tumor weight in PNI mice treated with or without PD168368 two weeks after tumor implantation was shown (n=5, one-way ANOVA and Tukey's multiple comparisons test).

**h-k** In situ images (**h**), HE staining (**i**, Left column:12.5×magnification, scale bars: 2.5 mm; **j**, Right column: 200×magnification, scale bars: 100 μm), macroscopic images (**k**) of the PNI mice treated with NMBR antagonist PD168368 were shown (n=5).

**l-n** Quantification of SFI (**l**), score (**m**), tumor weight (**n**) of the PNI mice treated with or without PD168368 five weeks after tumor implantation was shown (n=5, one-way ANOVA and Tukey's multiple comparisons test).

Data are shown as the photographs from one representative of three independent experiments. *P < 0.05, **P < 0.01, *** P <0.001, **** P <0.0001.


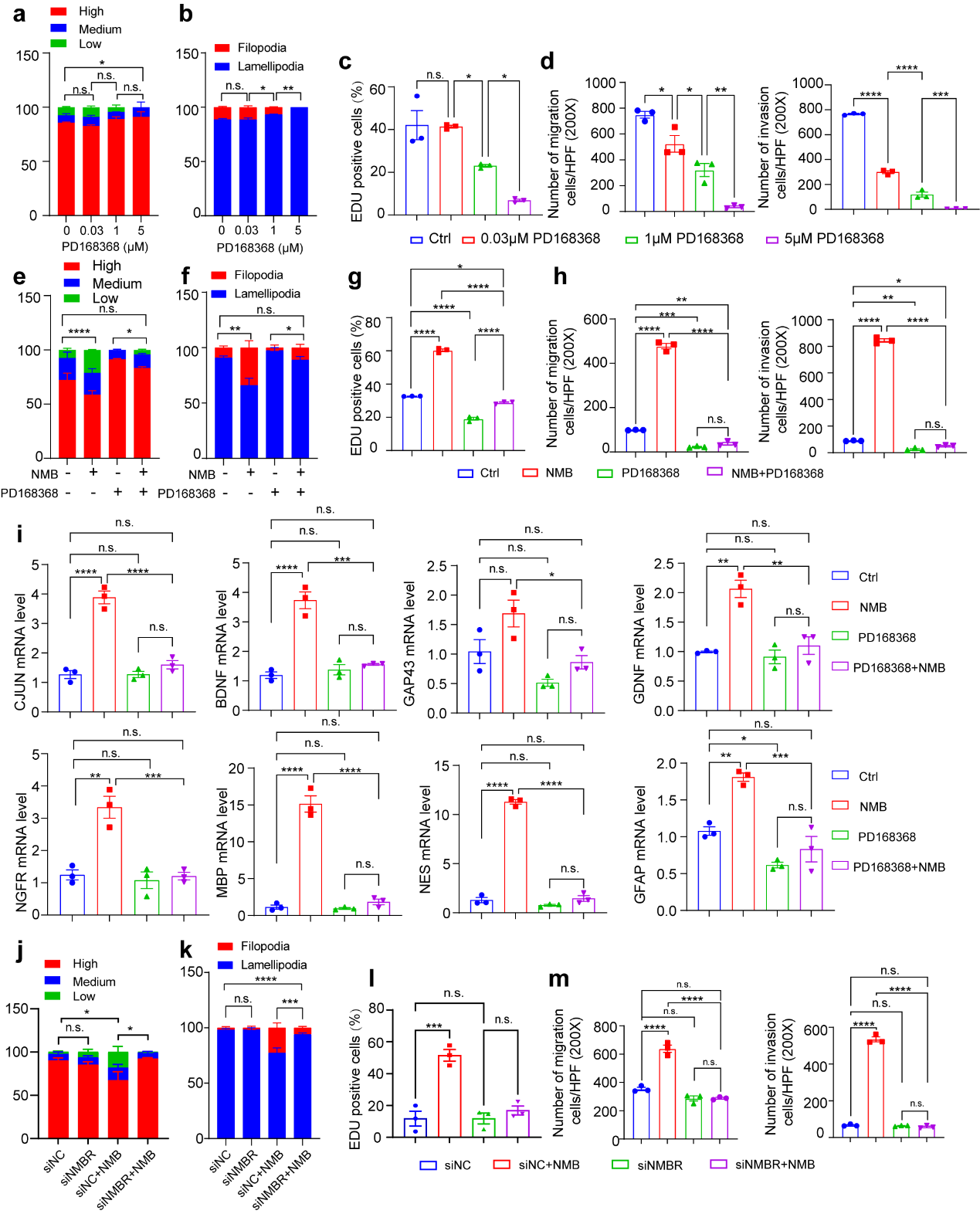

**Fig. S10 NMBR mediates the NMB-triggered reprogramming of Schwann cells.**

**a** Percentages of cells with low (green), medium (blue), or high (red) numbers of focal adhesion points were analyzed in RSC96 cells treated with PD168368 (n=3, chi-square test).

**b** Percentages of cells characterized by lamellipodium (blue) and filopodia (red) protrusions were analyzed in RSC96 cells treated with PD168368 (n=3, chi-square test).

**c** Quantification of EDU-positive cells was analyzed in RSC96 cells treated with PD168368 (n=3, one-way ANOVA and Tukey's multiple comparisons test).

**d** Quantification of migration and invasion cells was analyzed in RSC96 cells treated with PD168368 (n=3, one-way ANOVA and Tukey's multiple comparisons test).

**e** Percentages of cells with low (green), medium (blue), or high (red) numbers of focal adhesion points were analyzed in RSC96 cells treated with NMB and PD168368 (n=3, chi-square test).

**f** Percentages of cells characterized by lamellipodium (blue) and filopodia (red) protrusions were analyzed in RSC96 cells treated with NMB and PD168368 (n=3, chi-square test).

**g** Quantification of EDU-positive cells was analyzed in RSC96 cells treated with NMB and PD168368 (n=3, one-way ANOVA and Tukey's multiple comparisons test).

**h** Quantification of migration and invasion cells was analyzed in RSC96 cells treated with NMB and PD168368 (n=3, one-way ANOVA and Tukey's multiple comparisons test).

**i** cJUN, BDNF, GAP43, GDNF, NGFR, MBP, NES, and GFAP mRNA expression of RSC96 cells treated with NMB and PD168368 were examined by qRT-PCR (n=3, one-way ANOVA and Tukey's multiple comparisons test).

**j** Percentages of cells with low (green), medium (blue), or high (red) numbers of focal adhesion points were analyzed in NMBR-knockdown RSC96 cells treated with NMB (n=6, chi-square test).

**k** Percentages of NMBR-knockdown RSC96 cells characterized by lamellipodium (blue) and filopodia (red) protrusions were analyzed treated with NMB (n=6, chi-square test).

**l** Quantification of EDU-positive cells was analyzed in NMBR-knockdown SCs treated with NMB (n=3, one-way ANOVA and Tukey's multiple comparisons test).

**m** Quantification of migration and invasion cells was analyzed in NMBR-knockdown SCs treated with NMB (n=3, one-way ANOVA and Tukey's multiple comparisons test).

*P < 0.05, **P < 0.01, *** P <0.001, **** P <0.0001.


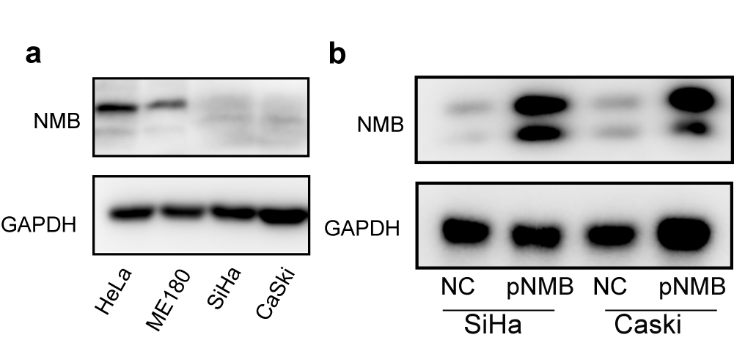


**Fig. S11 NMB-activated Schwann cells promote PNI.**

**a** NMB expression in cervical cancer cell lines HeLa, ME180, SiHa, and CaSki were confirmed by Western blot.

**b** Overexpression of NMB in SiHa and CaSki was confirmed by Western blot.

1. **Supplementary Tables**

**Supplementary Table 1** **Association between PNI and clinicopathologic characteristics of patients with cervical cancer**

|  | Subjects with PNI (N=70) | Subjects without PNI(N=353) | P value |
| --- | --- | --- | --- |
| Mean age (years) | 51.5 (± 10.58) | 49.31 (± 9.8) | 0.339 |
| FIGO stage |  |  | **＜0.001** |
| IA | 0 (0%) | 58(16.4%) |  |
| IB1 | 21(30.0%) | 112(31.7%) |  |
| IB2 | 12(17.1%) | 66(18.7%) |  |
| IB3 | 4(5.7%) | 31(8.8%) |  |
| IIA1 | 8(11.4%) | 32(9.1%) |  |
| IIA2 | 3(4.3%) | 12(3.4%) |  |
| IIB | 2(2.9%) | 4(1.1%) |  |
| IIIA | 0(0%) | 2(0.6%) |  |
| IIIB | 0(0%) | 0(0%) |  |
| IIIC1 | 20(28.6%) | 32(9.1%) |  |
| IIIC2 | 0(0%) | 4(1.1%) |  |
| Pathology |  |  | 0.549 |
| SCC | 55(78.6%) | 267(75.6%) |  |
| Adeno/adenosquamous carcinoma | 15 (21.4%) | 88 (24.9%) |  |
| Histological grade |  |  | 0.918 |
| Well | 7 (12.7%) | 30 (12.3%) |  |
| Moderate | 23 (41.8%) | 109 (44.9%) |  |
| Poor | 25 (45.5%) | 104 (42.8%) |  |
| Tumour size (cm) |  |  | 0.619 |
| ＜4 | 59 （84.3%） | 283 （81.8%） |  |
| ≥4 | 11 （15.7%） | 63 （18.2%） |  |
| Tumour size (cm) |  |  | **0.002** |
| ＜2 | 17 （24.3%） | 152 （45.9%） |  |
| ≥2 | 53 （75.7%） | 194 （56.1%） |  |
| Depth of invasion |  |  | ＜0.001 |
| ＜2/3 | 14 （20.3%） | 235 （67.0%） |  |
| ≥2/3 | 55 （79.7%） | 116 （33.0%） |  |
| Parametrial invasion |  |  | **＜0.001** |
| Present | 8 (11.4%) | 8 (2.4%) |  |
| Absent | 62 (88.6%) | 330 (97.6%) |  |
| LVSI |  |  | ＜0.001 |
| Present | 59 (85.5%) | 160 (47.3%) |  |
| Absent | 10 (14.5%) | 178 (52.7%) |  |
| Lymph nodes metastases |  |  | **＜0.001** |
| Positive | 18 (37.5%) | 32 (14.3%) |  |
| Negative | 30 (62.5%) | 191 (85.7%) |  |
| Human Papilloma Virus |  |  | 0.397 |
| Positive | 50 (71.4%) | 269 (76.2%) |  |
| Negative | 20 (28.6%) | 84 (23.8%) |  |

PNI indicates perineural invasion.

Abbreviations: LVSI: lymph-vascular space invasion

**Supplementary Table 2** **Top 12 genes of the differential gene analysis in HeLa and ME180 cells in an *in vitro* PNI model**

|  | HeLa | | | | ME180 | | | |
| --- | --- | --- | --- | --- | --- | --- | --- | --- |
| gene name | LogFC | P value | adj.P | Regulation | LogFC | P value | adj.P | Regulation |
| GADD45B | 1.3255123 | 3.93E-58 | 3.92E-57 | up | 4.18464299 | 0 | 0 | up |
| TGFB1 | 1.541794187 | 2.72E-177 | 8.27E-176 | up | 4.114348291 | 0 | 0 | up |
| SERPINE1 | 2.874850891 | 0 | 0 | up | 3.646527634 | 0 | 0 | up |
| TMEM158 | 4.003018879 | 0 | 0 | up | 3.211787855 | 1.26E-170 | 1.99E-169 | up |
| FOSL1 | 4.511206978 | 0 | 0 | up | 4.640072112 | 0 | 0 | up |
| LOXL2 | 1.732301441 | 0 | 0 | up | 4.401727904 | 0 | 0 | up |
| ITGA5 | 2.965845423 | 0 | 0 | up | 4.030179599 | 0 | 0 | up |
| GDF15 | -0.975590147 | 4.94E-15 | 1.89E-14 | down | 4.771351179 | 0 | 0 | up |
| NMB | 0.362038176 | 0.340530089 | 0.497878775 | up | 4.544423785 | 0 | 0 | up |
| ECM1 | 2.224749323 | 6.94E-48 | 5.82E-47 | up | 4.377960721 | 0 | 0 | up |
| ANGPTL4 | 4.677187738 | 0 | 0 | up | 4.414494684 | 0 | 0 | up |
| MT1X | 3.903780148 | 8.83E-156 | 2.33E-154 | up | 1.872036782 | 0 | 0 | up |

1. **Supplementary Captions for Movies**

**Movie S1.** **The neurite outgrows of DRG toward tumor cluster.**

The living cell imaging system with contentious capturing showed that the neurite outgrow of DRG toward tumor cluster was be induced by its co-culture with cervical cancer.

**Movie S2. The tumor cells were stationary before their direct interaction with the regenerated neurites.**

Before regenerated neurites interacted directly, the Zsgreen-labelled tumor cells were stationary and the morphology remained unchanged.

**Movie S3. The crosstalk between tumor and nerve initiated the PNI of cervical cancer.**

The migrated Schwann cells and regenerated axon contacted the adjacent tumor cell cluster repeatedly at a high velocity, causing the membrane protrusions of tumor cells (green) and their migration along the regenerated neurites.
