# Supplementary material for: Cervical cancer-produced neuromedin-B reprograms Schwann cells to initiate perineural invasion
Source: Cell Death Dis. 2024 Aug 30;15(8):636. doi: 10.1038/s41419-024-07030-9 (PMC11364772; doi:10.1038/s41419-024-07030-9)
Supplement: Supplementary file 2 — Original Raw Data [file 41419_2024_7030_MOESM2_ESM.docx]

Figure 1a

crescent-shaped incomplete encirclement


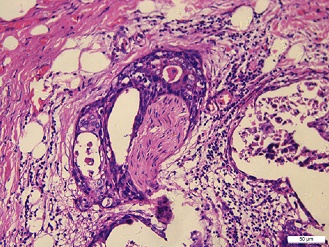


complete encirclement


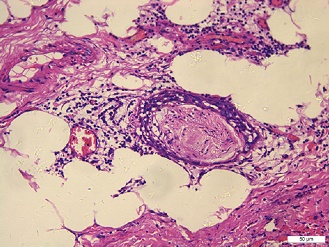


neural permeation


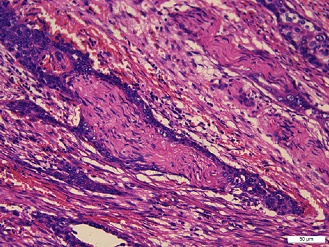


Figure 1b

PGP9.5 non-PNI


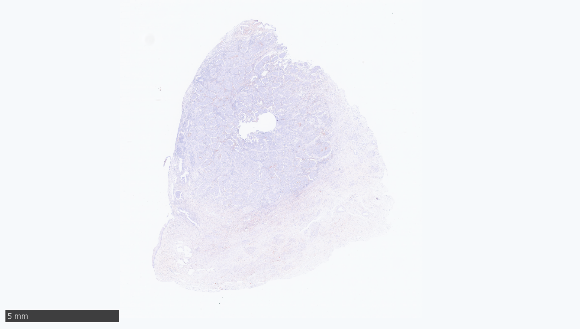


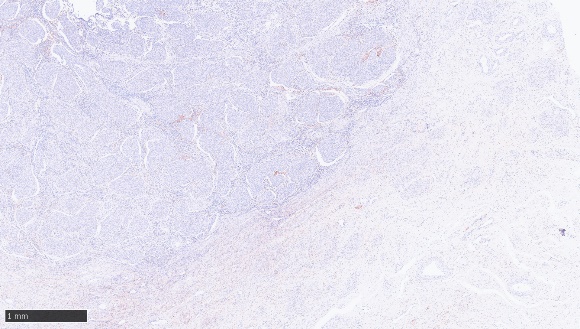


PGP9.5 PNI


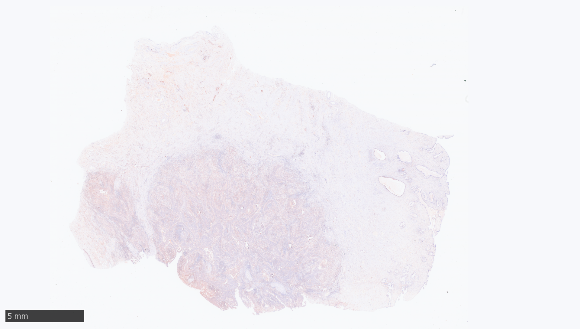


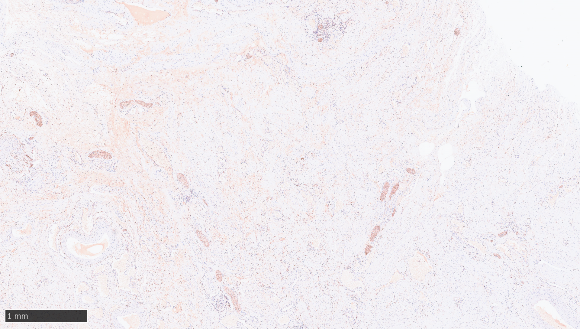


NF-L non-PNI


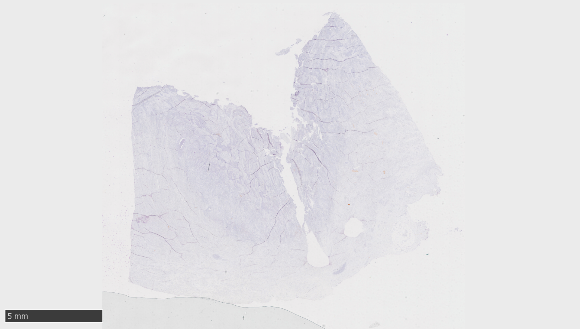


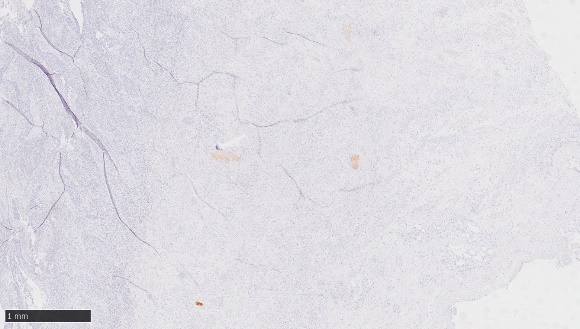


NF-L PNI


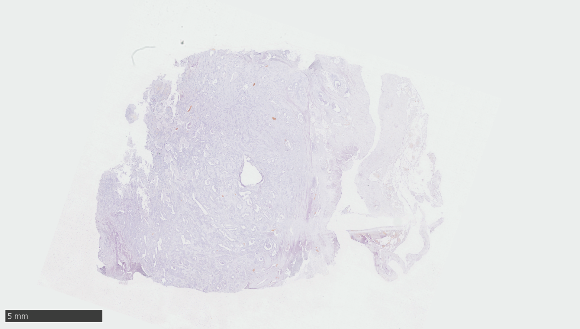


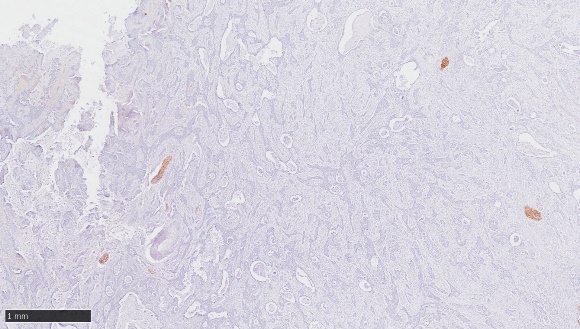


IHC score

| PGP9.5 | | NF-L | |
| --- | --- | --- | --- |
| Non-PNI  (n=8) | PNI  (n=6) | Non-PNI  (n=8) | PNI  (n=6) |
| 2 | 7 | 4 | 6 |
| 3 | 7 | 3 | 6 |
| 3 | 6 | 2 | 5 |
| 4 | 6 | 0 | 4 |
| 4 | 6 | 4 | 6 |
| 2 | 7 | 2 | 4 |
| 2 |  | 2 |  |
| 4 |  | 2 |  |

Figure 1c

DRG-day1


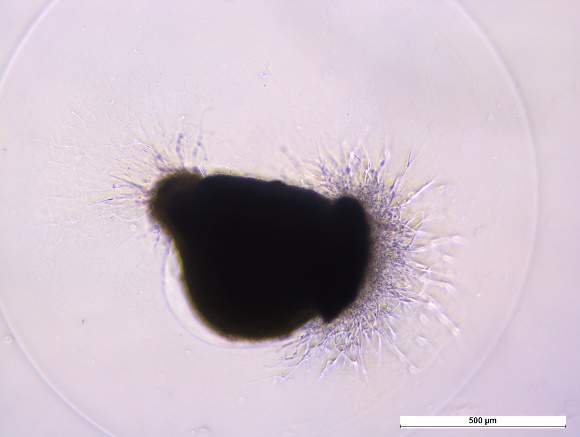


DRG-day2


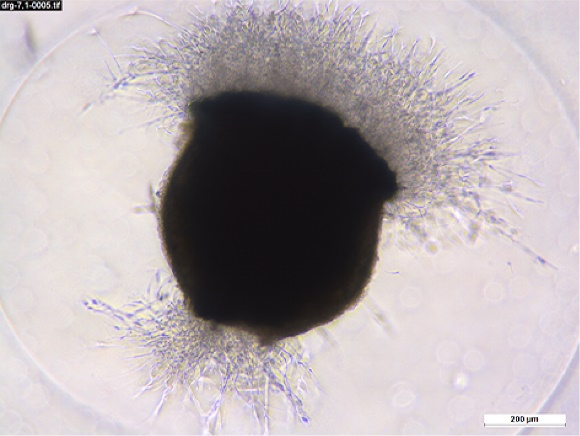


Hela+DRG-day1


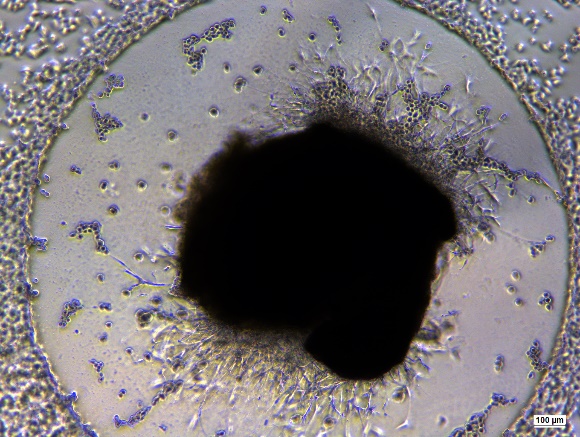


Hela+DRG-day2


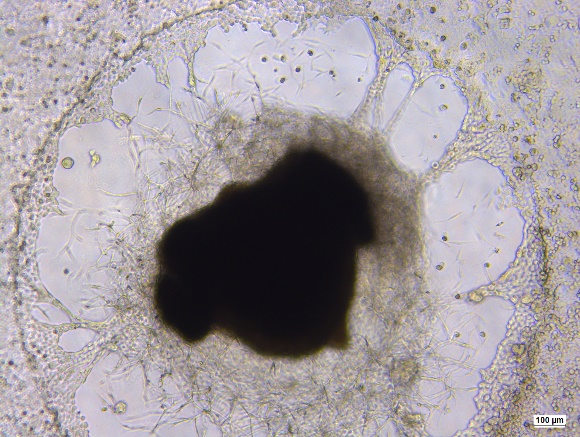


ME180+DRG-day1


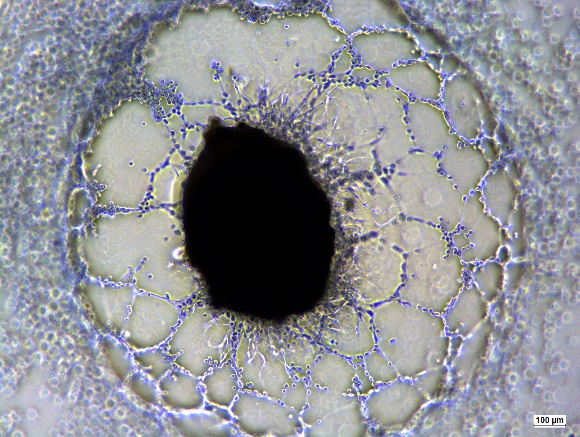


ME180+DRG-day2


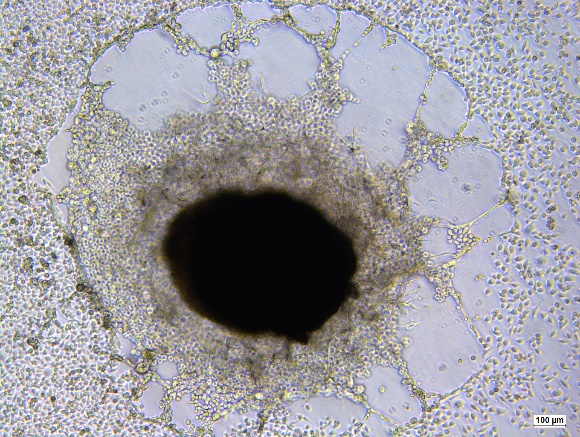


Figure 1d

HeLa


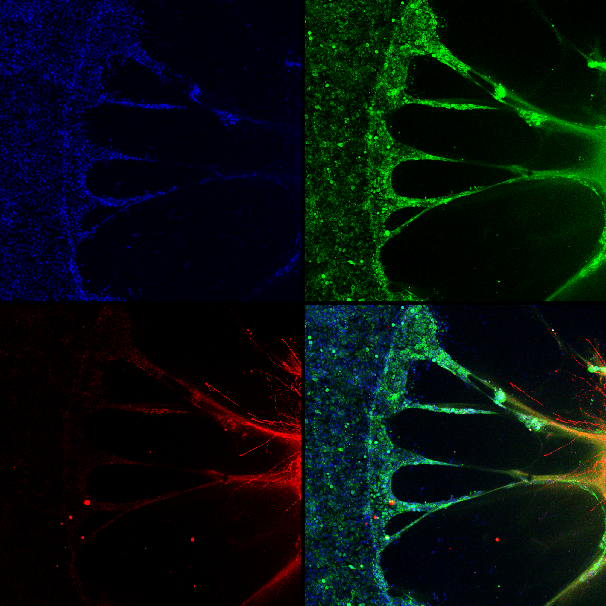


ME180


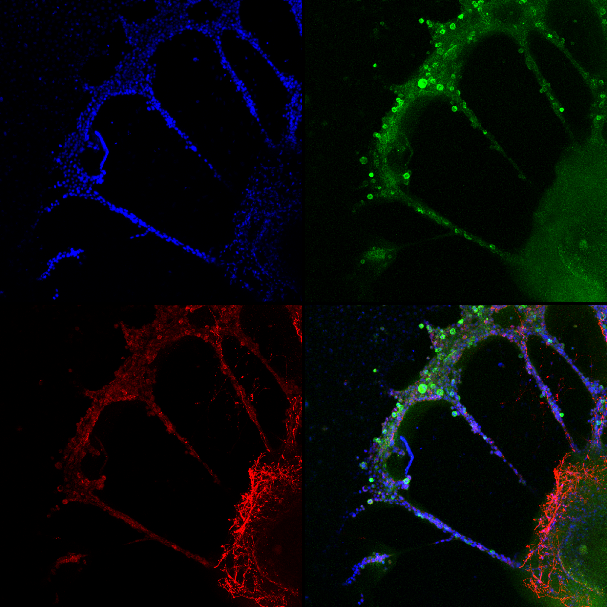


Figure 1f

Ctrl


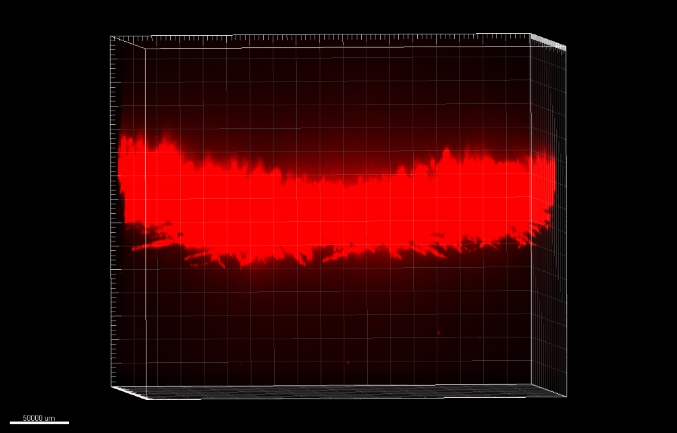


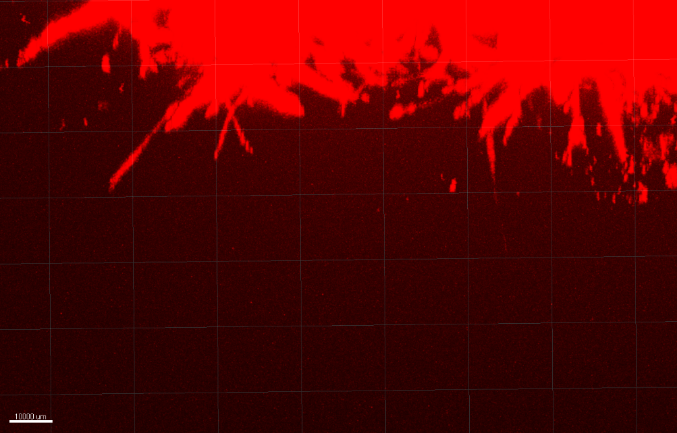


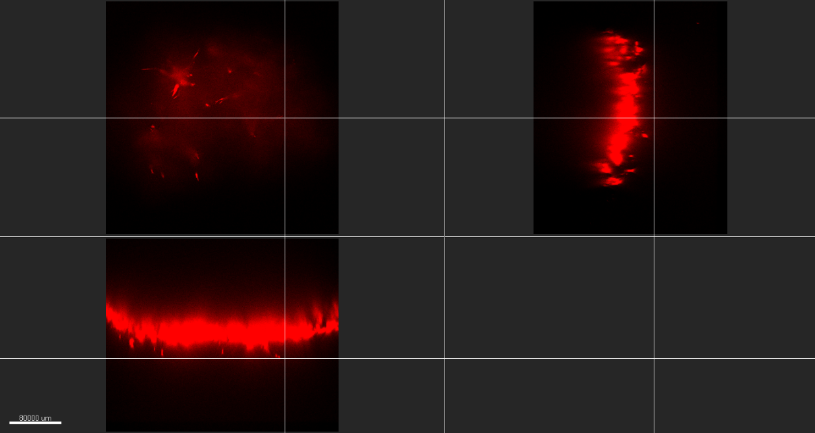


HcerEpic


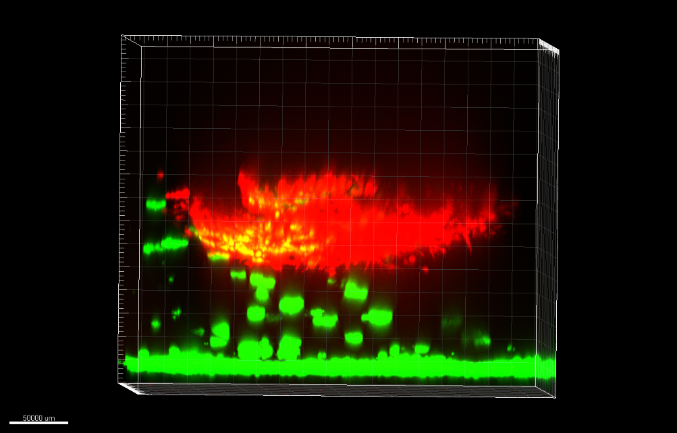


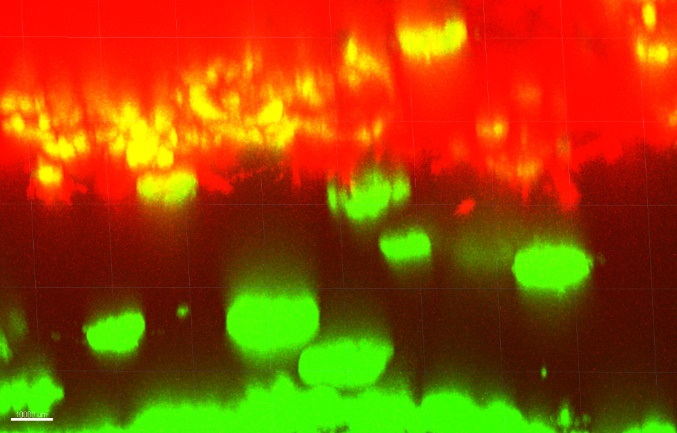


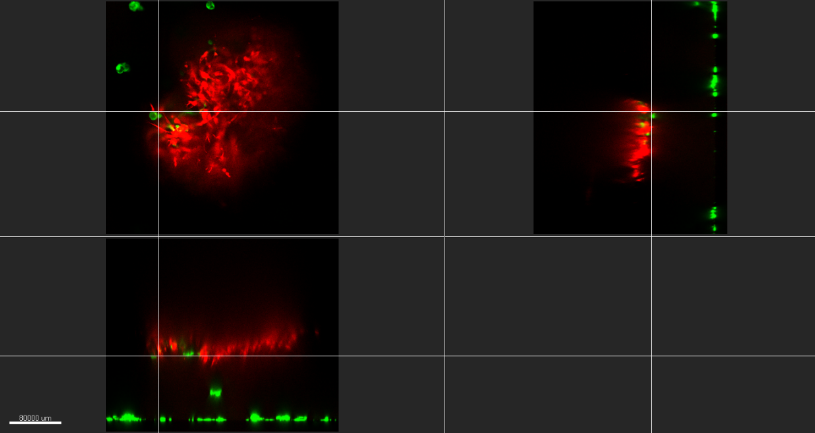


HeLa


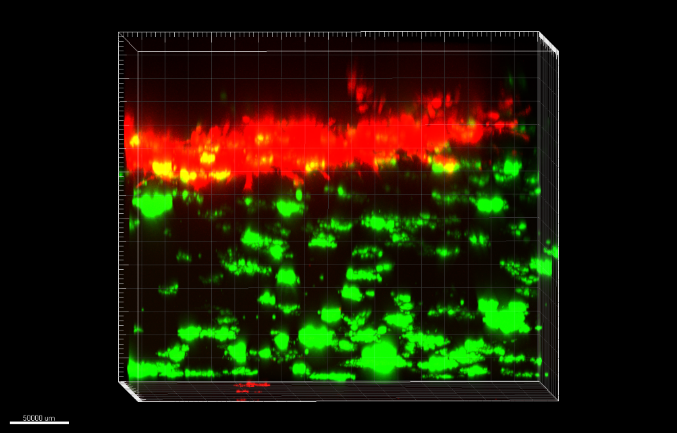


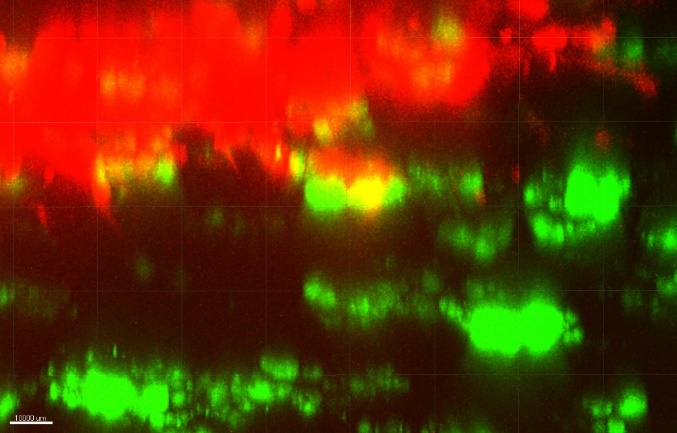


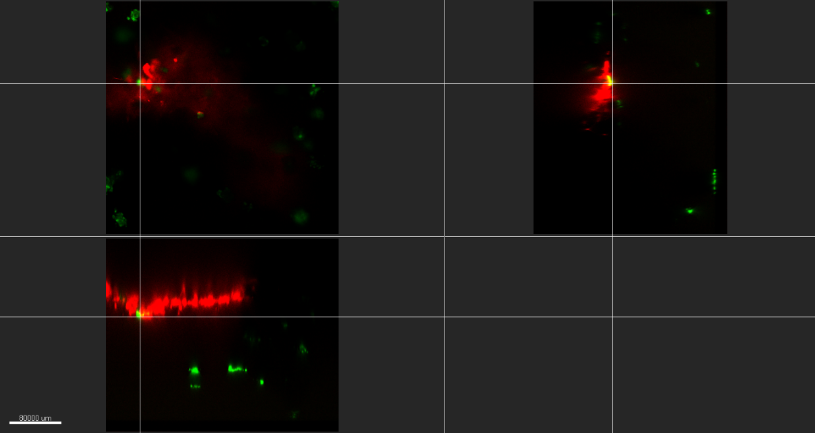


ME180


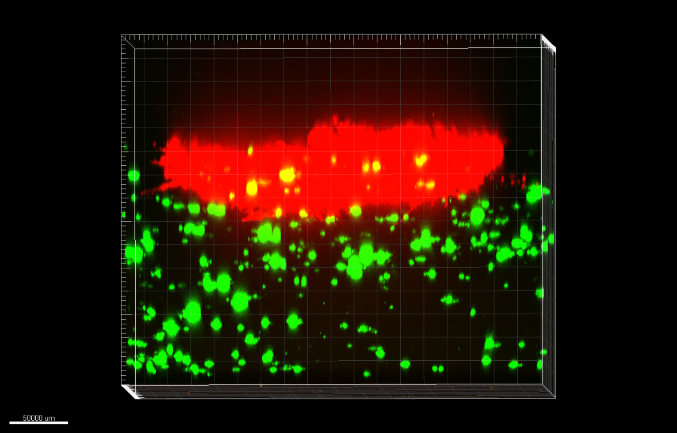


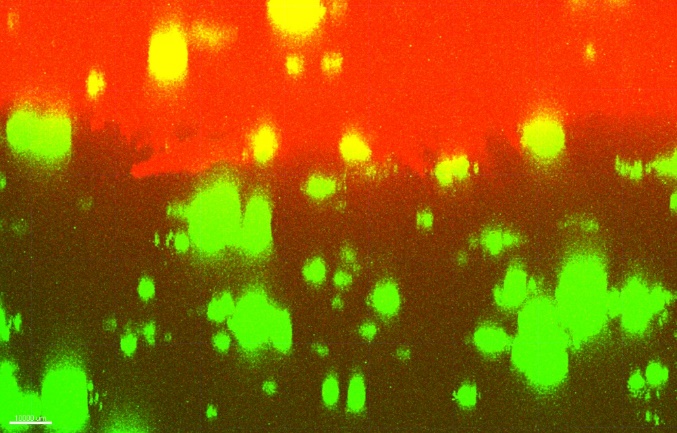


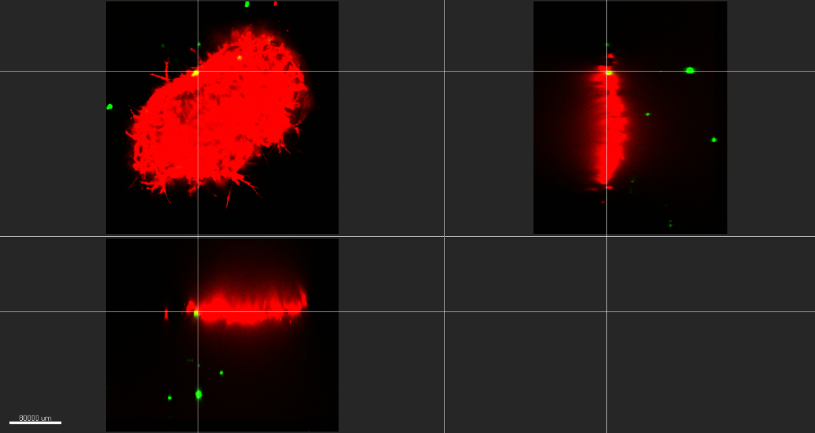


Figure 1g

Relative invasion distance (mm)

| Ctrl (n=3) | HcerEpic (n=3) | HeLa (n=3) | ME180 (n=3) |
| --- | --- | --- | --- |
| 4.325166667 | 3.694583333 | 5.52575 | 7.035416667 |
| 3.9495 | 3.630 | 5.56025 | 8.08575 |
| 4.27575 | 3.440666667 | 5.1785 | 6.469083333 |

Figure 1h

HeLa con-day1


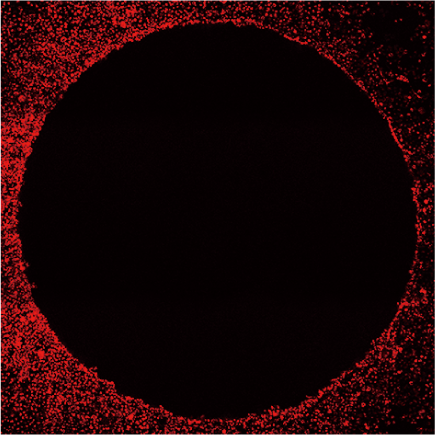


HeLa DRG-day1


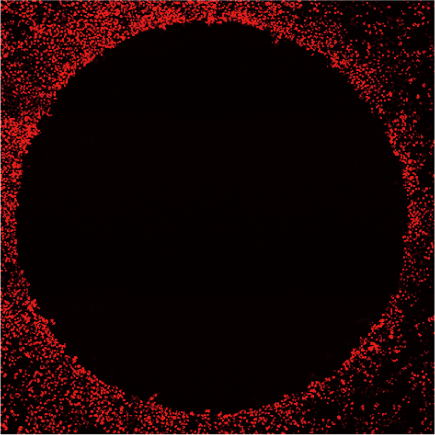


ME180 con-day1


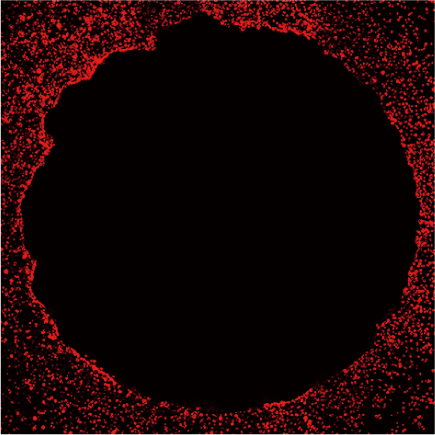


ME180 DRG-day1


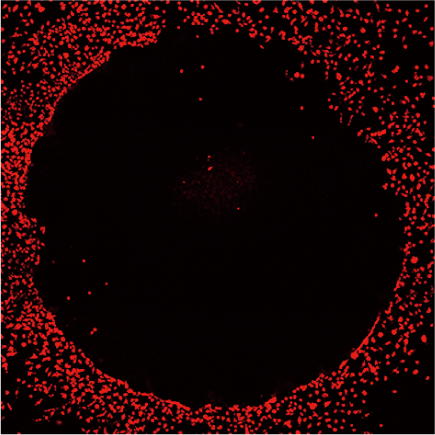


HeLa con-day4


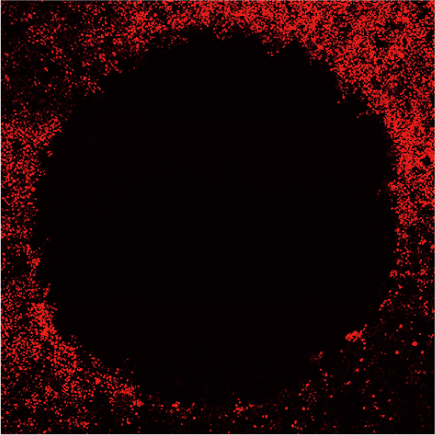


HeLa DRG-day4


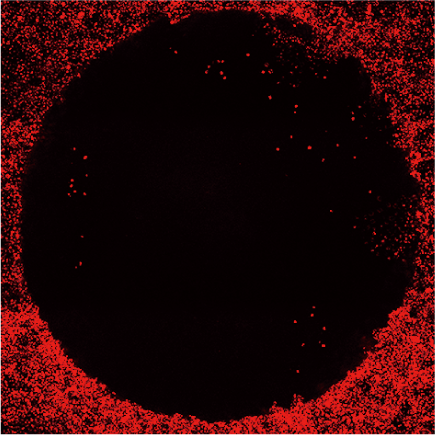


ME180 con-day4


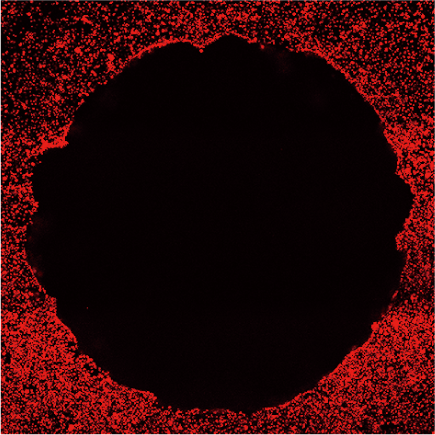


ME180 DRG-day4


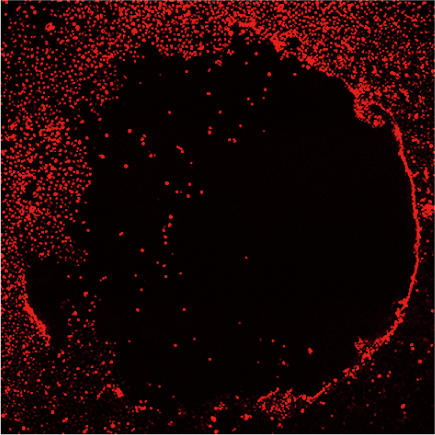


Figure 1j

HeLa

CK-17


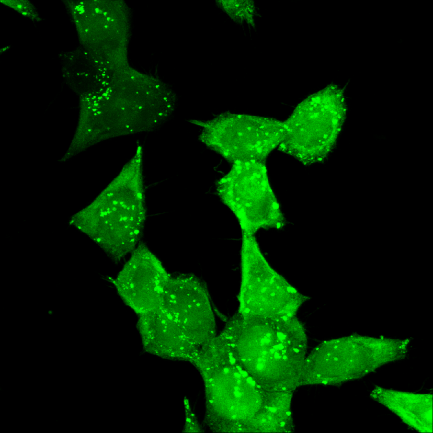


DAPI


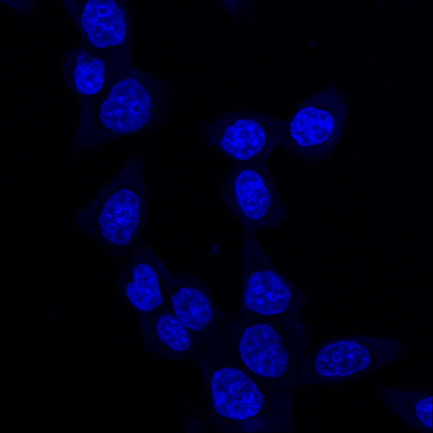


Merge


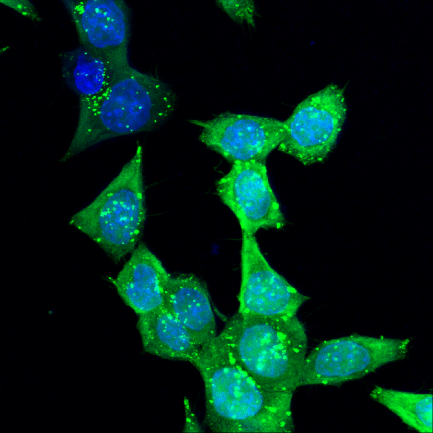


HeLa +DRG

CK-17


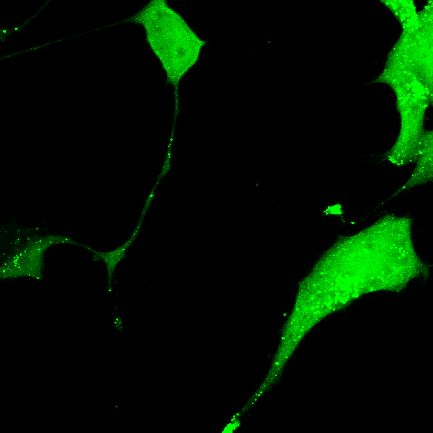


DAPI


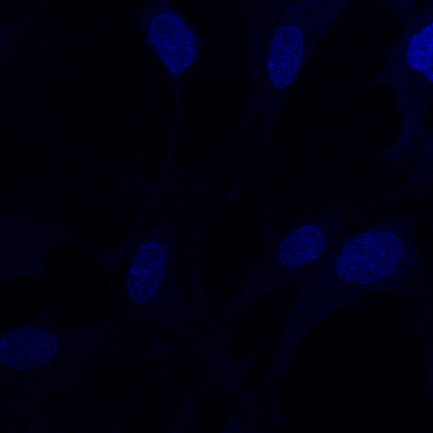


Merge


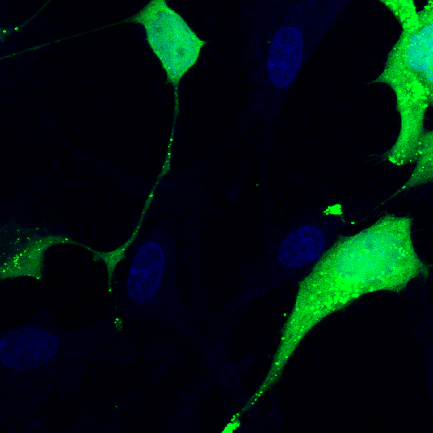


ME180

CK-17


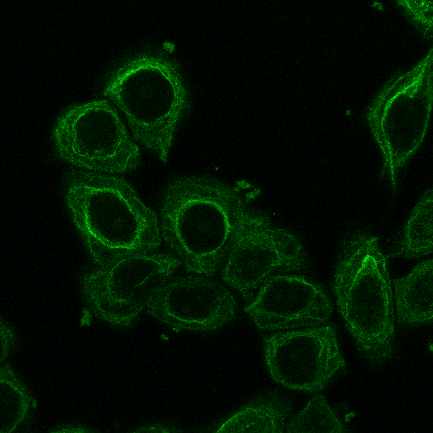


DAPI


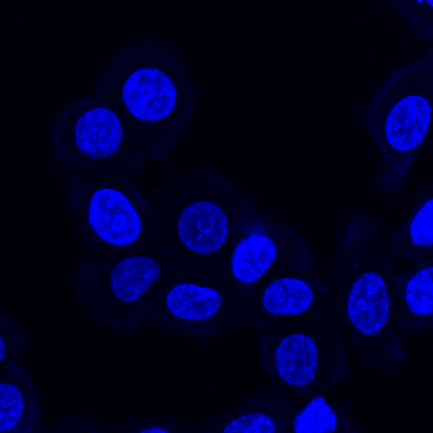


Merge


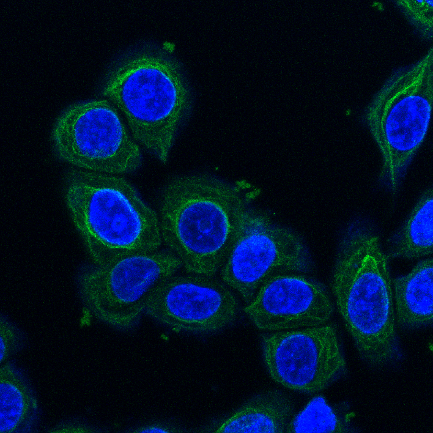


ME180 +DRG

CK-17


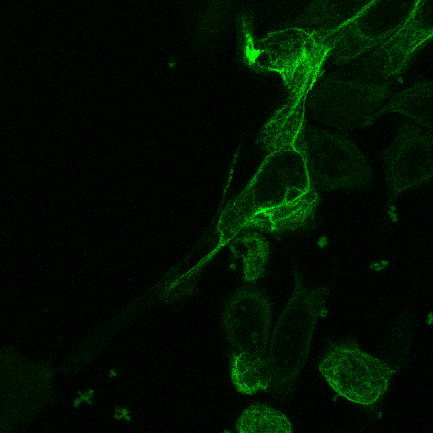


DAPI


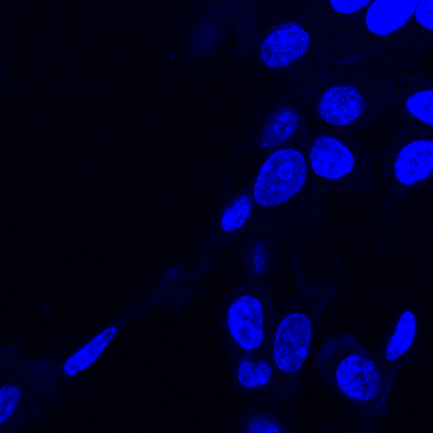


Merge


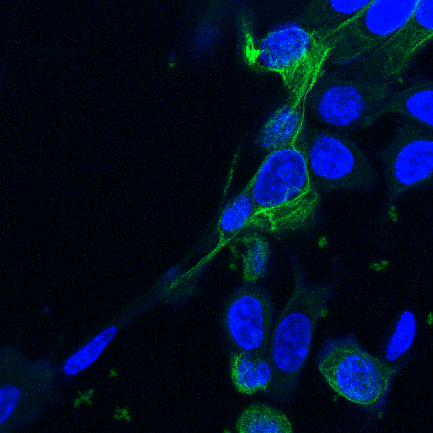


Figure S1a

| Stage (%) | I | II | III |
| --- | --- | --- | --- |
| Non-PNI | 75.6 | 13.6 | 10.8 |
| PNI | 52.8 | 18.6 | 28.6 |

Figure S1b

| Parametrial invasion (%) | Absent | Present |
| --- | --- | --- |
| Non-PNI | 97.6 | 2.4 |
| PNI | 88.6 | 11.4 |

Figure S1c

| Depth of invasion (%) | <2/3 | ≥2/3 |
| --- | --- | --- |
| Non-PNI | 67 | 33 |
| PNI | 20.3 | 79.7 |

Figure S1d

| LVSI (%) | Absent | Present |
| --- | --- | --- |
| Non-PNI | 52.7 | 47.3 |
| PNI | 14.5 | 85.5 |

Figure S1e

| Lymph nodes metastases (%) | Negative | Positive |
| --- | --- | --- |
| Non-PNI | 85.7 | 14.3 |
| PNI | 62.5 | 37.5 |

Figure S1f

p16 non-PNI


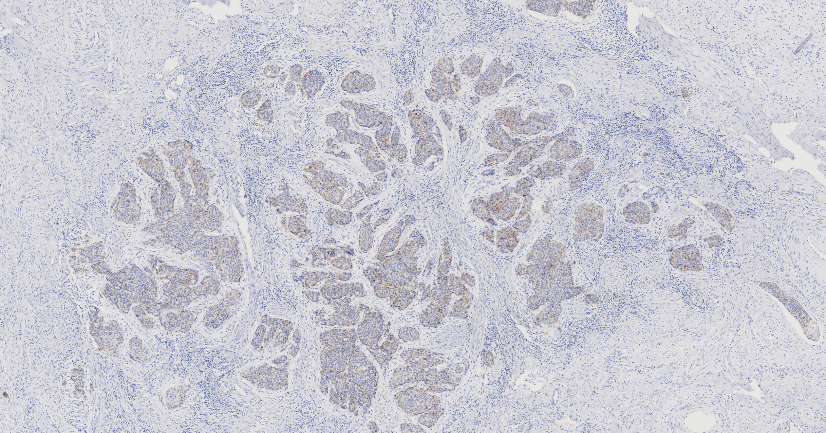


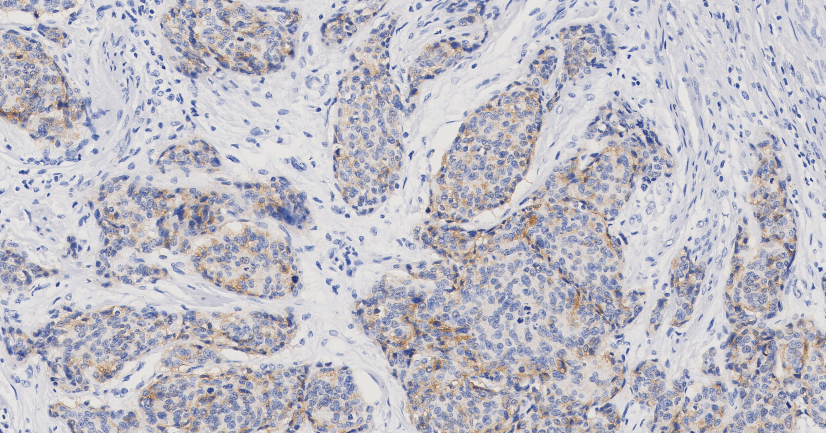


p16 PNI


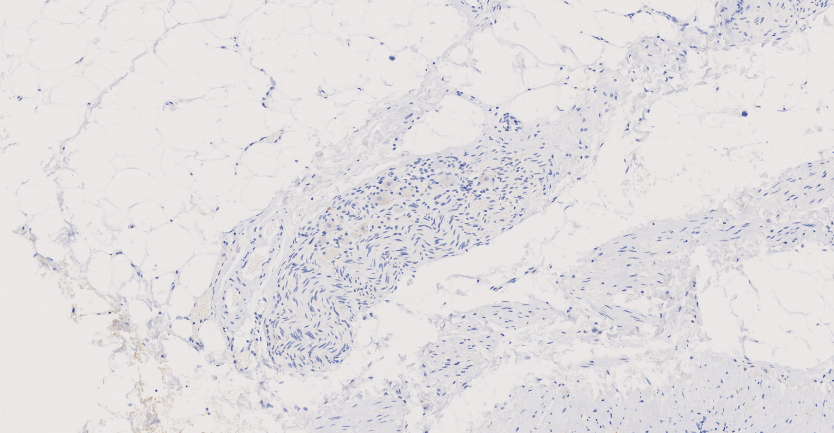


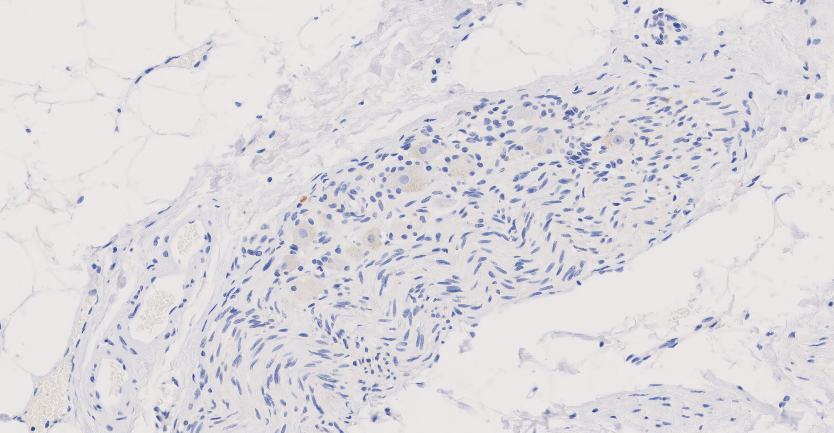


IHC score

| P16 | |
| --- | --- |
| Non-PNI  (n=8) | PNI  (n=6) |
| 3 | 2 |
| 3 | 0 |
| 2 | 1 |
| 2 | 2 |
| 1 | 2 |
| 2 | 6 |
| 1 |  |
| 3 |  |

Figure S1g

TH non-PNI


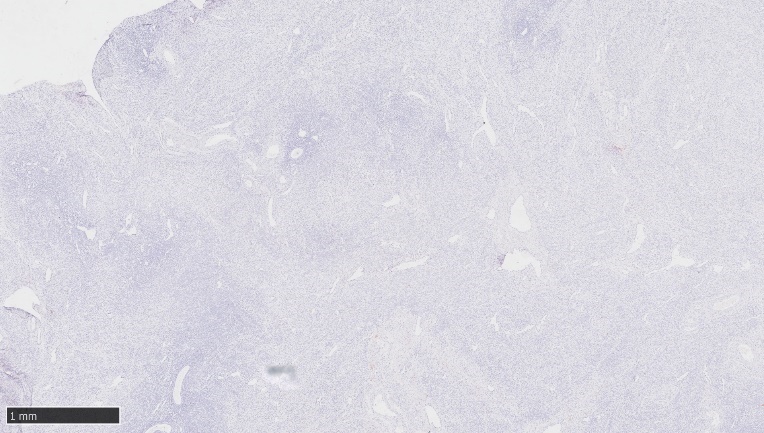


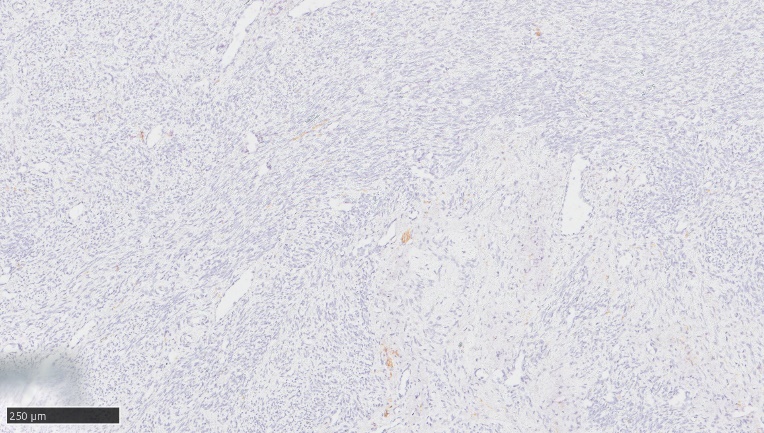


TH PNI


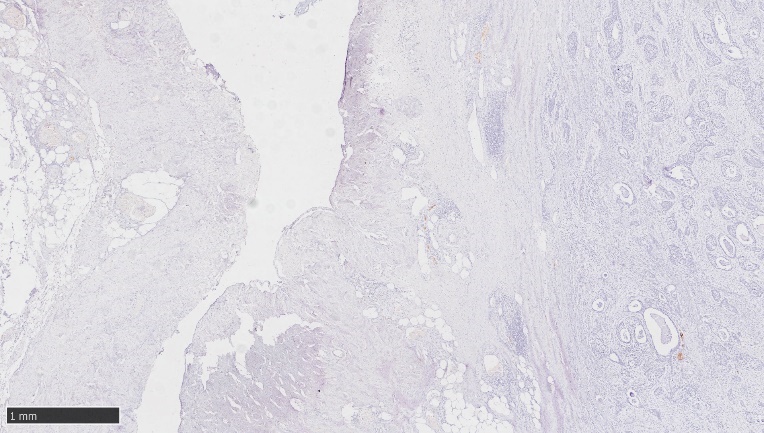


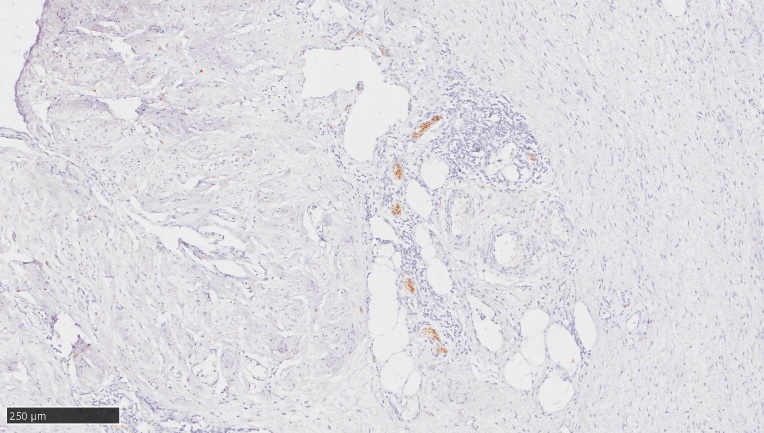


VAChT non-PNI


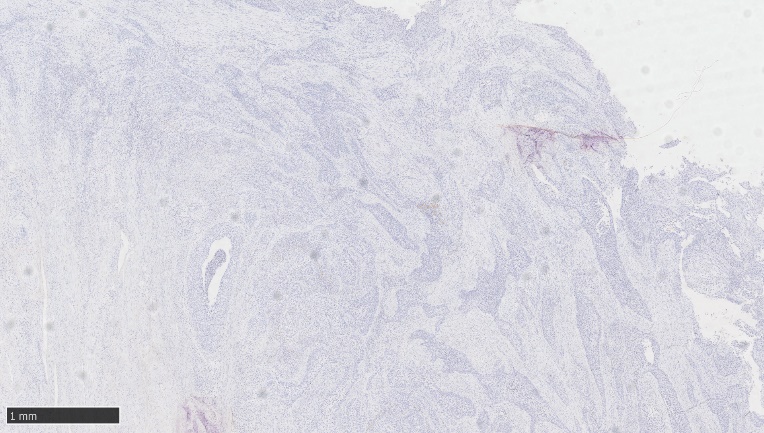


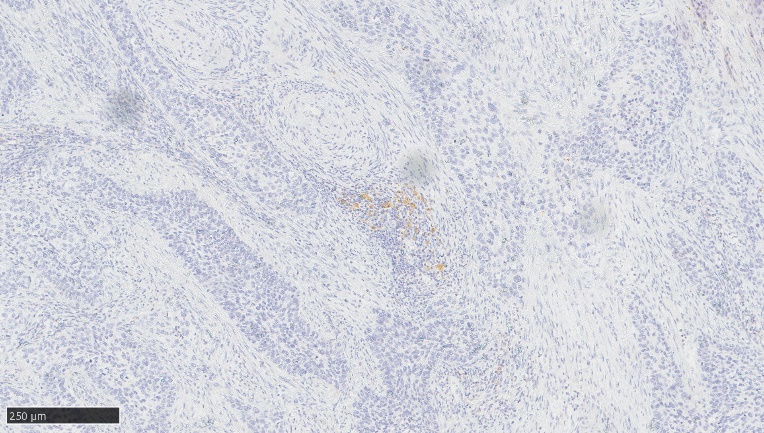


VAChT PNI


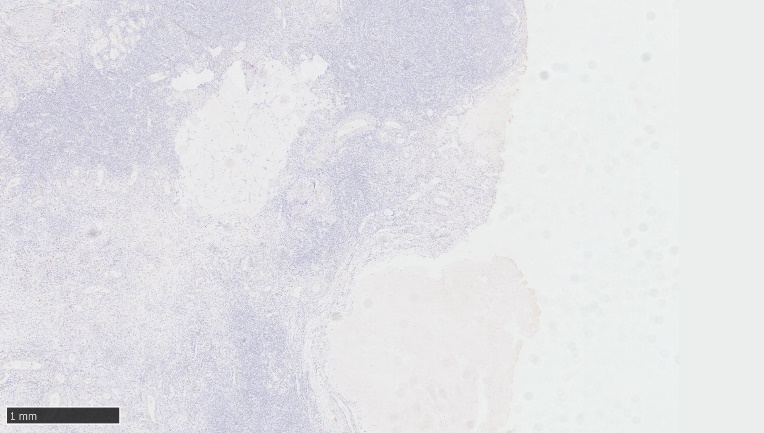


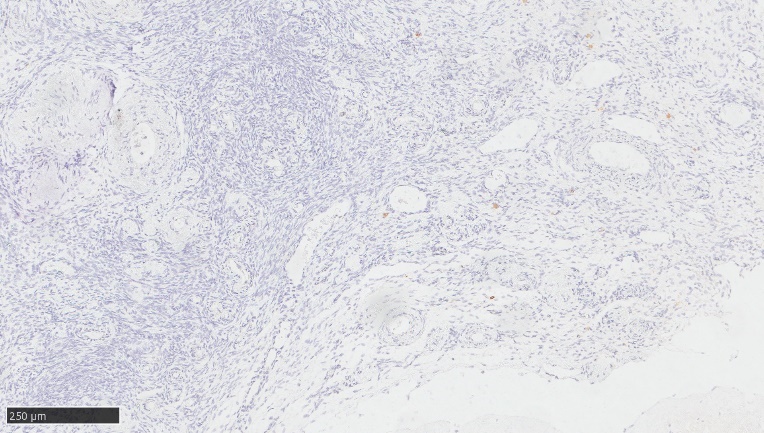


IHC score

| TH | | VAChT | |
| --- | --- | --- | --- |
| Non-PNI  (n=8) | PNI  (n=6) | Non-PNI  (n=8) | PNI  (n=6) |
| 5 | 6 | 0 | 0 |
| 2 | 5 | 0 | 2 |
| 2 | 4 | 0 | 0 |
| 2 | 3 | 0 | 0 |
| 6 | 6 | 0 | 0 |
| 2 | 5 | 2 | 0 |
| 4 |  | 0 |  |
| 2 |  | 0 |  |

Figure 2a

| log2 transformed | | HeLa | ME180 |
| --- | --- | --- | --- |
| Gene ID | Symbol |  |  |
| 4616 | GADD45B | 1.3255123 | 4.18464299 |
| 7040 | TGFB1 | 1.541794187 | 4.114348291 |
| 5054 | SERPINE1 | 2.874850891 | 3.646527634 |
| 25907 | TMEM158 | 4.003018879 | 3.211787855 |
| 8061 | FOSL1 | 4.511206978 | 4.640072112 |
| 4017 | LOXL2 | 1.732301441 | 4.401727904 |
| 3678 | ITGA5 | 2.965845423 | 4.030179599 |
| 9518 | GDF15 | -0.975590147 | 4.771351179 |
| 4828 | NMB | 0.362038176 | 4.544423785 |
| 1893 | ECM1 | 2.224749323 | 4.377960721 |
| 51129 | ANGPTL4 | 4.677187738 | 4.414494684 |
| 4501 | MT1X | 3.903780148 | 1.872036782 |

Figure 2b

|  | *18s* Ct value | *NMB* Ct value | 2^-∆∆Ct^ |
| --- | --- | --- | --- |
| HeLa | 12.19482708 | 29.13464546 | 0.774796842 |
| HeLa | 9.678668499 | 25.56107235 | 1.61250598 |
| HeLa | 13.3757844 | 30.26868725 | 0.8004069 |
|  |  |  |  |
| HeLa-RSC96 | 11.57088661 | 25.25379848 | 7.406523317 |
| HeLa-RSC96 | 11.56727695 | 25.176507 | 7.794617995 |
| HeLa-RSC96 | 10.87870169 | 24.56662178 | 7.380856635 |
|  |  |  |  |
| ME180 | 10.26761246 | 26.9590044 | 0.920389491 |
| ME180 | 10.05526972 | 26.77836418 | 0.900384982 |
| ME180 | 10.07729483 | 26.54048729 | 1.078118604 |
|  |  |  |  |
| ME180-RSC96 | 11.57088661 | 25.25379848 | 7.406523317 |
| ME180-RSC96 | 11.47398567 | 25.56452847 | 5.583483797 |
| ME180-RSC96 | 11.11619568 | 25.70014477 | 3.966205068 |

Figure 2c

NMB


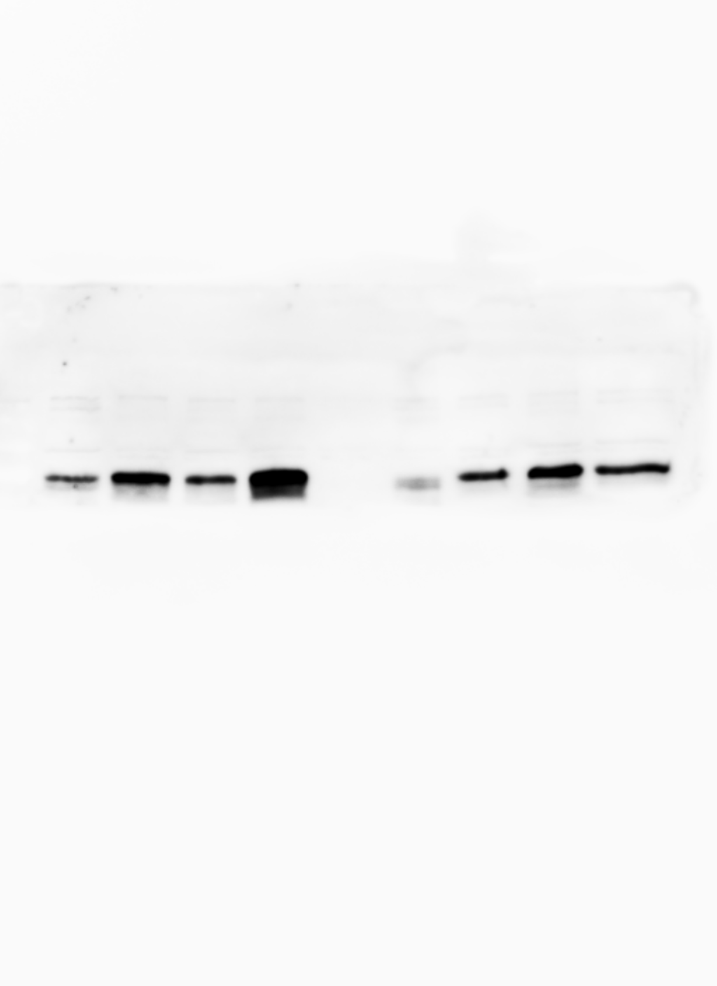


GAPDH


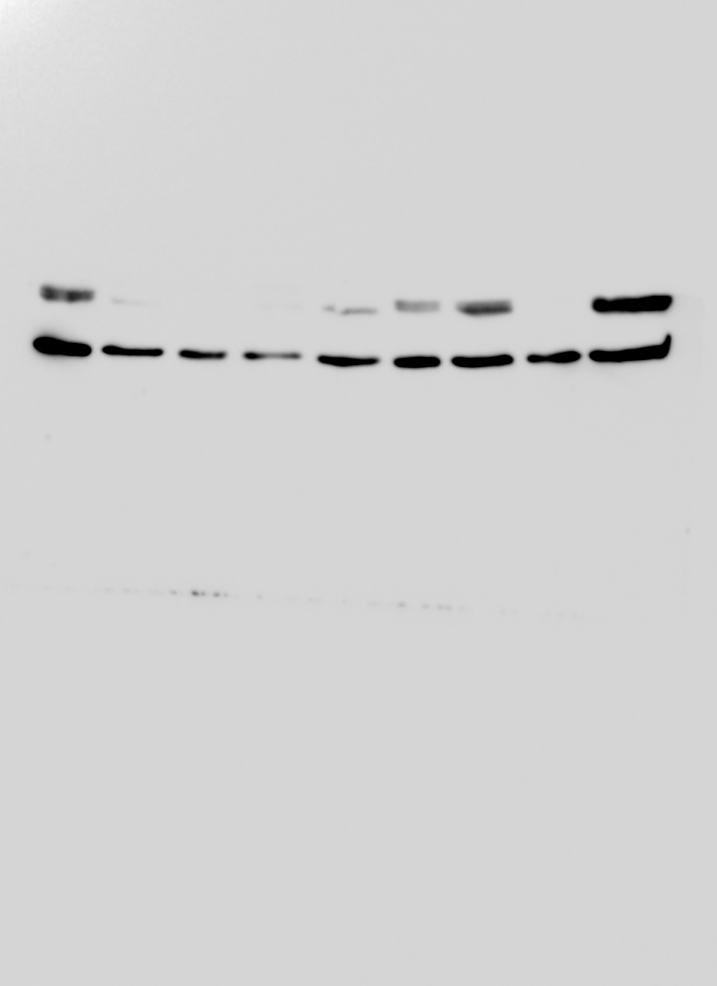


| HeLa | HeLa-DRG | ME180 | ME180-DRG |
| --- | --- | --- | --- |
| 0.957626 | 2.26584 | 1.044636 | 1.986991 |
| 1.124614 | 2.765904 | 0.856471 | 1.604360 |
| 0.922937 | 3.348437 | 1.098892 |  |

Figure 2d

Cohort 1: 306 CC tissues

| Row names(data) | NMB values |
| --- | --- |
| TCGA-VS-A9UJ-01 | 7.366788173 |
| TCGA-XS-A8TJ-01 | 5.252718409 |
| TCGA-EK-A2IR-01 | 4.294782469 |
| TCGA-VS-A9U5-01 | 4.880259412 |
| TCGA-ZJ-AAXN-01 | 4.463530912 |
| TCGA-DS-A0VL-01 | 6.690140487 |
| TCGA-C5-A1ML-01 | 4.96737153 |
| TCGA-VS-A8QM-01 | 5.52364977 |
| TCGA-Q1-A73P-01 | 4.682831701 |
| TCGA-UC-A7PF-01 | 5.464759739 |
| TCGA-FU-A23L-01 | 5.782513408 |
| TCGA-C5-A1MI-01 | 4.461515463 |
| TCGA-4J-AA1J-01 | 6.318775972 |
| TCGA-C5-A1BJ-01 | 6.627989055 |
| TCGA-EA-A411-01 | 6.186670901 |
| TCGA-EA-A3HT-01 | 5.921745746 |
| TCGA-GH-A9DA-01 | 4.398076692 |
| TCGA-C5-A7CM-01 | 5.482922481 |
| TCGA-FU-A3NI-01 | 5.411216063 |
| TCGA-C5-A905-01 | 5.893985852 |
| TCGA-C5-A2LY-01 | 5.476857971 |
| TCGA-JX-A3Q8-01 | 4.831005687 |
| TCGA-DS-A7WI-01 | 4.759912145 |
| TCGA-JX-A5QV-01 | 3.004033901 |
| TCGA-ZJ-AAXJ-01 | 6.149216679 |
| TCGA-C5-A8YR-01 | 5.206103988 |
| TCGA-DS-A1O9-01 | 7.731450735 |
| TCGA-C5-A7UH-01 | 7.930186762 |
| TCGA-EX-A1H5-01 | 6.296879508 |
| TCGA-C5-A1BK-01 | 6.246668403 |
| TCGA-DR-A0ZL-01 | 7.624739172 |
| TCGA-JW-A5VI-01 | 4.967625143 |
| TCGA-ZJ-AAXI-01 | 4.561350578 |
| TCGA-C5-A3HF-01 | 4.969113643 |
| TCGA-EX-A449-01 | 3.633861281 |
| TCGA-FU-A3WB-01 | 5.448881134 |
| TCGA-MU-A5YI-01 | 4.766107467 |
| TCGA-JW-AAVH-01 | 6.471252504 |
| TCGA-C5-A1MH-01 | 5.345186996 |
| TCGA-C5-A902-01 | 6.424186449 |
| TCGA-C5-A1BQ-01 | 6.423462199 |
| TCGA-C5-A3HD-01 | 6.063673346 |
| TCGA-ZJ-AAXB-01 | 6.28266405 |
| TCGA-VS-A8EL-01 | 6.331429347 |
| TCGA-ZJ-AAXF-01 | 4.690059451 |
| TCGA-JW-A852-01 | 7.714534028 |
| TCGA-VS-A8QH-01 | 2.989938264 |
| TCGA-DG-A2KH-01 | 6.249885213 |
| TCGA-VS-A9V5-01 | 3.060704577 |
| TCGA-EX-A69M-01 | 5.16560247 |
| TCGA-EA-A97N-01 | 5.211031668 |
| TCGA-VS-A9U6-01 | 7.301446809 |
| TCGA-EK-A2IP-01 | 6.61148373 |
| TCGA-C5-A1ME-01 | 5.645240513 |
| TCGA-EA-A3QE-01 | 5.081539893 |
| TCGA-C5-A7UC-01 | 6.412266669 |
| TCGA-LP-A4AV-01 | 6.987403869 |
| TCGA-ZJ-AAXT-01 | 5.034862775 |
| TCGA-JW-A5VL-01 | 5.516765305 |
| TCGA-EK-A2RM-01 | 8.303436926 |
| TCGA-EK-A2GZ-01 | 6.123132287 |
| TCGA-Q1-A6DT-01 | 6.129761088 |
| TCGA-VS-A8Q8-01 | 5.319654059 |
| TCGA-MA-AA43-01 | 5.090332645 |
| TCGA-LP-A7HU-01 | 6.626237613 |
| TCGA-EA-A556-01 | 5.611880567 |
| TCGA-C5-A1MJ-01 | 4.907256032 |
| TCGA-C5-A8YQ-01 | 3.617521829 |
| TCGA-VS-A958-01 | 4.880866708 |
| TCGA-VS-A9UV-01 | 6.244196363 |
| TCGA-EK-A2RO-01 | 7.373076553 |
| TCGA-VS-A9UR-01 | 5.10522968 |
| TCGA-Q1-A6DV-01 | 4.241420735 |
| TCGA-MA-AA3Y-01 | 4.212475958 |
| TCGA-FU-A770-01 | 5.076259785 |
| TCGA-ZJ-AAXA-01 | 6.194217097 |
| TCGA-FU-A3EO-01 | 5.958464556 |
| TCGA-C5-A2M2-01 | 7.041609875 |
| TCGA-HM-A3JJ-01 | 5.835853352 |
| TCGA-C5-A0TN-01 | 6.308826824 |
| TCGA-C5-A907-01 | 4.676882319 |
| TCGA-C5-A7X8-01 | 4.161476577 |
| TCGA-EX-A1H6-01 | 4.404467197 |
| TCGA-C5-A7X5-01 | 6.537566314 |
| TCGA-FU-A3YQ-01 | 4.731281011 |
| TCGA-VS-A8QC-01 | 6.703828842 |
| TCGA-FU-A2QG-01 | 6.619423282 |
| TCGA-MA-AA42-01 | 4.039919204 |
| TCGA-IR-A3LB-01 | 4.343279893 |
| TCGA-DG-A2KJ-01 | 5.761322511 |
| TCGA-ZJ-AAXD-01 | 4.818380218 |
| TCGA-HM-A6W2-01 | 5.319816609 |
| TCGA-DS-A1OB-01 | 4.218998098 |
| TCGA-EK-A2RN-01 | 3.802606834 |
| TCGA-VS-A9UY-01 | 6.219275024 |
| TCGA-LP-A5U3-01 | 7.270045783 |
| TCGA-Q1-A73Q-01 | 5.503622236 |
| TCGA-LP-A4AW-01 | 5.834501389 |
| TCGA-JW-A69B-01 | 5.429569101 |
| TCGA-Q1-A5R3-01 | 8.046231292 |
| TCGA-DS-A7WF-01 | 4.813334849 |
| TCGA-VS-A8Q9-01 | 6.369618283 |
| TCGA-EK-A2RL-01 | 5.770038782 |
| TCGA-FU-A3HY-01 | 6.316024426 |
| TCGA-C5-A7CG-01 | 3.649052066 |
| TCGA-VS-A94W-01 | 3.881703769 |
| TCGA-FU-A3TX-01 | 2.575312331 |
| TCGA-DR-A0ZM-01 | 5.230998398 |
| TCGA-EK-A2H0-01 | 4.490422516 |
| TCGA-EA-A3HR-01 | 5.005584131 |
| TCGA-EA-A5O9-01 | 7.154891001 |
| TCGA-IR-A3LF-01 | 3.981843522 |
| TCGA-VS-A950-01 | 8.069102781 |
| TCGA-EA-A439-01 | 4.351289098 |
| TCGA-EA-A5FO-01 | 5.735668389 |
| TCGA-VS-A9V4-01 | 3.578431568 |
| TCGA-DG-A2KM-01 | 5.83205706 |
| TCGA-JX-A3PZ-01 | 4.802937642 |
| TCGA-EA-A3HS-01 | 6.048809435 |
| TCGA-C5-A1MK-01 | 8.066657005 |
| TCGA-ZJ-A8QQ-01 | 4.376373742 |
| TCGA-VS-A94X-01 | 4.434287945 |
| TCGA-ZJ-AAXU-01 | 7.421554054 |
| TCGA-C5-A7CK-01 | 3.20565838 |
| TCGA-ZJ-AB0H-01 | 6.326603858 |
| TCGA-C5-A2LZ-01 | 7.022027075 |
| TCGA-VS-A957-01 | 6.091494627 |
| TCGA-VS-A8EH-01 | 8.04537744 |
| TCGA-VS-A8EJ-01 | 5.363213138 |
| TCGA-C5-A1BE-01 | 6.176588067 |
| TCGA-ZX-AA5X-01 | 5.637824676 |
| TCGA-DG-A2KL-01 | 4.712419697 |
| TCGA-MU-A8JM-01 | 6.503541145 |
| TCGA-IR-A3LC-01 | 6.272019455 |
| TCGA-JW-A5VJ-01 | 6.791600516 |
| TCGA-MU-A51Y-01 | 5.610115355 |
| TCGA-MA-AA41-01 | 4.726531532 |
| TCGA-VS-A9UO-01 | 5.357024093 |
| TCGA-C5-A3HE-01 | 5.05853297 |
| TCGA-C5-A2LT-01 | 4.374865602 |
| TCGA-EK-A2PM-01 | 3.735002193 |
| TCGA-C5-A1MQ-01 | 2.845550267 |
| TCGA-C5-A1M7-01 | 5.521650688 |
| TCGA-EA-A5ZE-01 | 6.063921367 |
| TCGA-VS-A94Z-01 | 6.013471193 |
| TCGA-VS-A9UC-01 | 8.195440126 |
| TCGA-C5-A1BN-01 | 6.759161161 |
| TCGA-EA-A43B-01 | 6.360929584 |
| TCGA-DS-A1OA-01 | 5.719807706 |
| TCGA-MY-A5BE-01 | 4.441722492 |
| TCGA-EK-A2RC-01 | 6.173273375 |
| TCGA-VS-A8EB-01 | 5.265174318 |
| TCGA-MY-A5BF-01 | 4.793843874 |
| TCGA-ZJ-AB0I-01 | 6.614051648 |
| TCGA-JW-A5VK-01 | 6.825246297 |
| TCGA-UC-A7PG-06 | 5.93213442 |
| TCGA-C5-A8XI-01 | 5.064693192 |
| TCGA-EK-A2RB-01 | 5.258639521 |
| TCGA-IR-A3LK-01 | 5.776622528 |
| TCGA-BI-A0VR-01 | 6.133748454 |
| TCGA-VS-A9UP-01 | 4.786141528 |
| TCGA-C5-A2M1-01 | 4.557771546 |
| TCGA-EA-A1QS-01 | 5.169804772 |
| TCGA-C5-A7CO-01 | 3.549299517 |
| TCGA-VS-A959-01 | 7.127424866 |
| TCGA-VS-A9UB-01 | 4.727631887 |
| TCGA-FU-A40J-01 | 5.247570592 |
| TCGA-C5-A3HL-01 | 6.694958174 |
| TCGA-C5-A1BI-01 | 5.427213937 |
| TCGA-ZJ-AAX4-01 | 4.368768349 |
| TCGA-EK-A3GK-01 | 6.469270616 |
| TCGA-VS-A9V2-01 | 5.084995421 |
| TCGA-HM-A3JK-01 | 5.273344308 |
| TCGA-C5-A1M9-01 | 6.112343585 |
| TCGA-UC-A7PI-01 | 4.634570036 |
| TCGA-BI-A20A-01 | 6.051493916 |
| TCGA-Q1-A73O-01 | 4.940519157 |
| TCGA-ZJ-A8QR-01 | 5.426764579 |
| TCGA-ZJ-AAX8-01 | 7.0023346 |
| TCGA-MA-AA3Z-01 | 7.200637744 |
| TCGA-C5-A7UI-01 | 7.309092494 |
| TCGA-EX-A3L1-01 | 6.238334036 |
| TCGA-VS-A9UU-01 | 4.603460184 |
| TCGA-C5-A7UE-01 | 6.013361759 |
| TCGA-EA-A78R-01 | 7.549441229 |
| TCGA-VS-A8QA-01 | 6.51956392 |
| TCGA-C5-A1M8-01 | 7.719781016 |
| TCGA-EA-A1QT-01 | 6.642173026 |
| TCGA-JW-A5VH-01 | 3.909629115 |
| TCGA-HM-A6W2-06 | 5.340259599 |
| TCGA-WL-A834-01 | 6.117160997 |
| TCGA-Q1-A73S-01 | 6.606251218 |
| TCGA-VS-A9V0-01 | 5.127435184 |
| TCGA-LP-A5U2-01 | 5.78505091 |
| TCGA-DS-A7WH-01 | 4.297961725 |
| TCGA-EX-A8YF-01 | 2.53652543 |
| TCGA-C5-A2LX-01 | 5.473621336 |
| TCGA-DS-A5RQ-01 | 6.836670233 |
| TCGA-EA-A3Y4-01 | 3.923691159 |
| TCGA-EA-A3QD-01 | 6.650613792 |
| TCGA-C5-A7CL-01 | 7.724526813 |
| TCGA-VS-A8EC-01 | 6.075911225 |
| TCGA-VS-A9UH-01 | 3.985655257 |
| TCGA-EK-A3GJ-01 | 4.004159779 |
| TCGA-ZJ-A8QO-01 | 2.170918516 |
| TCGA-EK-A2RE-01 | 5.400729183 |
| TCGA-VS-A9UM-01 | 5.938650419 |
| TCGA-EA-A3HU-01 | 5.501958167 |
| TCGA-IR-A3LL-01 | 6.366976088 |
| TCGA-UC-A7PG-01 | 5.332296188 |
| TCGA-HG-A2PA-01 | 6.898843202 |
| TCGA-Q1-A5R2-01 | 7.54607104 |
| TCGA-HM-A4S6-01 | 6.685058512 |
| TCGA-LP-A4AX-01 | 3.460441152 |
| TCGA-EA-A5ZF-01 | 4.189666447 |
| TCGA-FU-A3HZ-01 | 4.645597914 |
| TCGA-Q1-A6DW-01 | 7.408814592 |
| TCGA-VS-A9V3-01 | 7.121984127 |
| TCGA-C5-A7X3-01 | 5.045827359 |
| TCGA-RA-A741-01 | 5.074214265 |
| TCGA-VS-A9U7-01 | 6.225945831 |
| TCGA-IR-A3LH-01 | 2.789165833 |
| TCGA-EK-A3GN-01 | 5.211273133 |
| TCGA-VS-A9UQ-01 | 5.945731761 |
| TCGA-Q1-A73R-01 | 5.113408797 |
| TCGA-MA-AA3X-01 | 5.858247123 |
| TCGA-EA-A6QX-01 | 7.600213248 |
| TCGA-VS-A94Y-01 | 6.81402739 |
| TCGA-C5-A7XC-01 | 5.289782834 |
| TCGA-LP-A4AU-01 | 4.621735417 |
| TCGA-C5-A8YT-01 | 4.759917469 |
| TCGA-VS-A953-01 | 6.250912321 |
| TCGA-EK-A2R9-01 | 5.59726299 |
| TCGA-EA-A3HQ-01 | 6.220374458 |
| TCGA-EA-A410-01 | 5.482957965 |
| TCGA-DS-A1OC-01 | 6.349901305 |
| TCGA-C5-A1BF-01 | 5.339550746 |
| TCGA-MY-A913-01 | 5.798436807 |
| TCGA-MA-AA3W-01 | 5.763207054 |
| TCGA-VS-A952-01 | 3.223716002 |
| TCGA-DS-A1OD-01 | 4.198886991 |
| TCGA-EK-A2PI-01 | 7.170605112 |
| TCGA-C5-A1M5-01 | 5.053563194 |
| TCGA-C5-A1MP-01 | 6.679026811 |
| TCGA-JX-A3Q0-01 | 6.156728288 |
| TCGA-EK-A2PL-01 | 6.146993212 |
| TCGA-FU-A3TQ-01 | 7.547821499 |
| TCGA-EK-A2R7-01 | 4.659056854 |
| TCGA-EK-A2PK-01 | 2.29166249 |
| TCGA-VS-A8EK-01 | 5.969406093 |
| TCGA-2W-A8YY-01 | 4.691645824 |
| TCGA-EA-A50E-01 | 5.848713067 |
| TCGA-EA-A5ZD-01 | 4.343727596 |
| TCGA-EK-A2H1-01 | 5.144340101 |
| TCGA-VS-AA62-01 | 4.965728884 |
| TCGA-MY-A5BD-01 | 5.299391206 |
| TCGA-EX-A69L-01 | 6.044641105 |
| TCGA-DS-A0VK-01 | 7.67082476 |
| TCGA-UC-A7PD-01 | 5.544788286 |
| TCGA-EK-A3GM-01 | 4.667818343 |
| TCGA-VS-A8QF-01 | 6.77697908 |
| TCGA-EK-A2RK-01 | 5.903831245 |
| TCGA-C5-A1M6-01 | 7.671128455 |
| TCGA-JW-A5VG-01 | 5.392674617 |
| TCGA-VS-A8EI-01 | 6.076326066 |
| TCGA-VS-A9UT-01 | 3.220051296 |
| TCGA-Q1-A5R1-01 | 5.241100346 |
| TCGA-VS-A8EG-01 | 6.998951411 |
| TCGA-DS-A0VM-01 | 7.604430914 |
| TCGA-FU-A57G-01 | 3.921188751 |
| TCGA-EA-A4BA-01 | 4.79634337 |
| TCGA-DS-A0VN-01 | 4.447440342 |
| TCGA-PN-A8MA-01 | 3.906159445 |
| TCGA-DG-A2KK-01 | 5.009969662 |
| TCGA-FU-A5XV-01 | 6.506221086 |
| TCGA-C5-A1MF-01 | 6.646884112 |
| TCGA-VS-A9UI-01 | 5.72767817 |
| TCGA-EK-A2RJ-01 | 3.200912333 |
| TCGA-C5-A8XK-01 | 7.338408369 |
| TCGA-C5-A8ZZ-01 | 3.998746114 |
| TCGA-VS-A9UZ-01 | 3.94858216 |
| TCGA-R2-A69V-01 | 6.344640338 |
| TCGA-C5-A7CH-01 | 6.705575715 |
| TCGA-C5-A1BM-01 | 5.926820192 |
| TCGA-C5-A2LS-01 | 4.71918344 |
| TCGA-IR-A3L7-01 | 4.956200729 |
| TCGA-C5-A8XH-01 | 4.977943868 |
| TCGA-VS-A9V1-01 | 4.766563361 |
| TCGA-VS-A9UD-01 | 6.650847833 |
| TCGA-C5-A1MN-01 | 6.777064587 |
| TCGA-IR-A3LI-01 | 5.822549162 |
| TCGA-C5-A8XJ-01 | 5.700262145 |
| TCGA-VS-A954-01 | 5.867562926 |
| TCGA-EK-A2PG-01 | 6.256676692 |
| TCGA-FU-A23K-01 | 7.180799306 |
| TCGA-C5-A2LV-01 | 4.785670858 |
| TCGA-DS-A3LQ-01 | 5.40895976 |
| TCGA-C5-A1BL-01 | 6.709559462 |
| TCGA-EK-A2RA-01 | 6.265888793 |
| TCGA-IR-A3LA-01 | 3.438492473 |
| TCGA-C5-A7CJ-01 | 5.898783961 |
| TCGA-BI-A0VS-01 | 5.755448441 |
| TCGA-EA-A44S-01 | 5.902186964 |
| TCGA-C5-A901-01 | 5.285990275 |
| TCGA-VS-A9UL-01 | 5.969562656 |
| TCGA-EK-A2R8-01 | 6.949283957 |

Cohort 2: 22 normal tissues

| Row names(data) | NMB values |
| --- | --- |
| TCGA-FU-A3EO-11 | 4.739081197 |
| TCGA-HM-A3JJ-11 | 4.033863452 |
| TCGA-MY-A5BF-11 | 4.868622569 |
| GTEX-N7MT-0826-SM-EV794 | 3.521050737 |
| GTEX-OHPN-2226-SM-E9TI7 | 4.054848477 |
| GTEX-P78B-2326-SM-EZ6KO | 4.172327492 |
| GTEX-P78B-2426-SM-EWRM2 | 3.508428653 |
| GTEX-PLZ4-2226-SM-EZ6KS | 4.09085343 |
| GTEX-Q2AG-2326-SM-EZ6KY | 3.412781525 |
| GTEX-S32W-1526-SM-4AD6Z | 3.868884273 |
| GTEX-S32W-1626-SM-4AD6G | 3.625270489 |
| GTEX-S341-1126-SM-4AD6T | 3.950468414 |
| GTEX-S341-1326-SM-4AD72 | 4.075532631 |
| GTEX-S4UY-1426-SM-4AD6Y | 5.269407241 |
| GTEX-T5JW-0726-SM-4DM6D | 4.13422094 |
| GTEX-T5JW-0826-SM-EYYVD | 3.885574364 |
| GTEX-T6MO-1426-SM-4DM73 | 3.475084883 |
| GTEX-TML8-0726-SM-4DXTT | 4.338424415 |
| GTEX-TSE9-2726-SM-4DXSQ | 4.146492307 |
| GTEX-TSE9-2826-SM-4DXTF | 4.27351589 |
| GTEX-U3ZN-1626-SM-4DXTZ | 3.629939409 |
| GTEX-ZPIC-1326-SM-DO91Y | 3.802193217 |

Figure 2e

NMB Normal


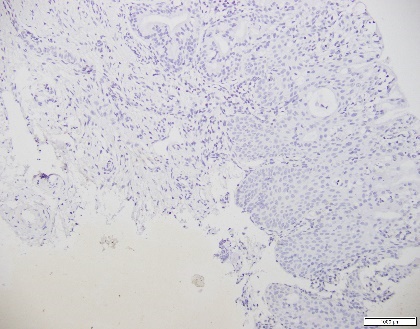


NMB CIN 1


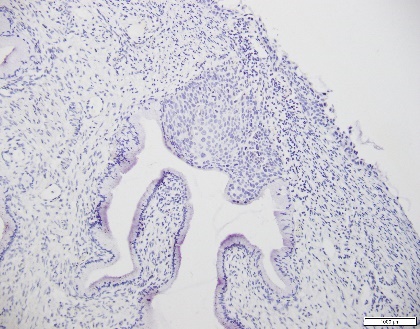


NMB CIN 2-3


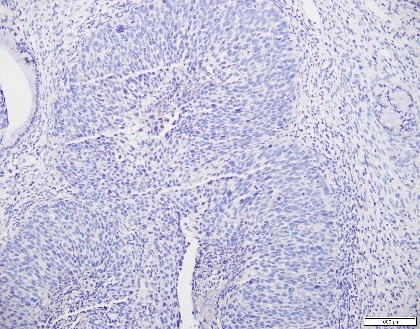


NMB CIS


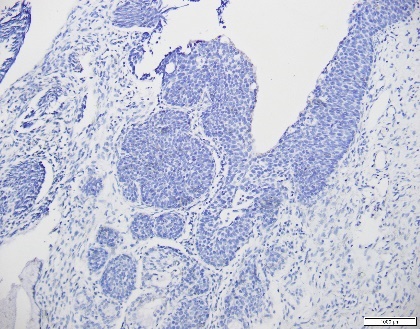


NMB CC


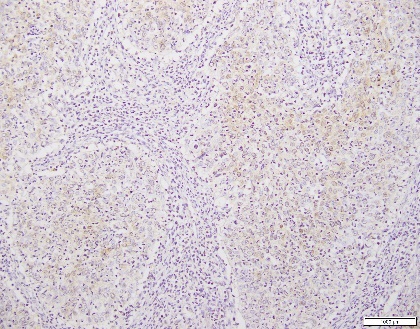


Figure 2f

NMB IHC score

| Normal  (n=8) | CIN 1  (n=22) | CIN 2-3  (n=22) | CIS  (n=22) | CC  (n=38) |
| --- | --- | --- | --- | --- |
| 0 | 0 | 0 | 0 | 0 |
| 2 | 0 | 0 | 0 | 1.4 |
| 0 | 0 | 0 | 0 | 0 |
| 0 | 0 | 0 | 0 | 0 |
| 0 | 0 | 0 | 0 | 0 |
| 2 | 0 | 0 | 0 | 0 |
| 0 | 0 | 0 | 0 | 0 |
| 0 | 0 | 0 | 0 | 0 |
|  | 0 | 0 | 0 | 0 |
|  | 0 | 0 | 0 | 0 |
|  | 0 | 0 | 0 | 0 |
|  | 0 | 0 | 0 | 0 |
|  | 0 | 0 | 0 | 0 |
|  | 0 | 0 | 0 | 0 |
|  | 0 | 0 | 3 | 0 |
|  | 0 | 0 | 0 | 3.1 |
|  | 0 | 0 | 0 | 1.6 |
|  | 0 | 0 | 0 | 0 |
|  | 0 | 0 | 0 | 8 |
|  | 0 | 0 | 0 | 4 |
|  | 0 | 0 | 0 | 12 |
|  | 0 | 0 | 0 | 2 |
|  |  |  |  | 4 |
|  |  |  |  | 3.2 |
|  |  |  |  | 4 |
|  |  |  |  | 4.8 |
|  |  |  |  | 12 |
|  |  |  |  | 8.4 |
|  |  |  |  | 8 |
|  |  |  |  | 3.6 |
|  |  |  |  | 7.2 |
|  |  |  |  | 6 |
|  |  |  |  | 3.6 |
|  |  |  |  | 10.8 |
|  |  |  |  | 4 |
|  |  |  |  | 4 |
|  |  |  |  | 6.4 |
|  |  |  |  | 0 |

Figure 2g

NMB non-PNI


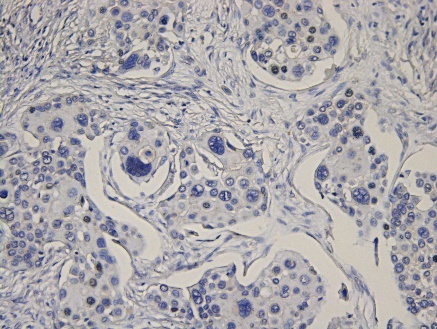


NMB PNI


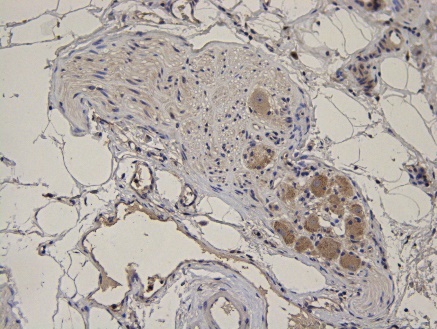


Figure 2h

NMB IHC score

| Non-PNI  (n=18) | PNI  (n=20) |
| --- | --- |
| 0 | 8 |
| 1.4 | 4 |
| 0 | 12 |
| 0 | 2 |
| 0 | 4 |
| 0 | 3.2 |
| 0 | 4 |
| 0 | 4.8 |
| 0 | 12 |
| 0 | 8.4 |
| 0 | 8 |
| 0 | 3.6 |
| 0 | 7.2 |
| 0 | 6 |
| 0 | 3.6 |
| 3.1 | 10.8 |
| 1.6 | 4 |
| 0 | 4 |
|  | 6.4 |
|  | 0 |

Figure 2i

| Negative (n=74) | Positive (n=38) |
| --- | --- |
| 0 | 0 |
| 1 | 1.4 |
| 0 | 0 |
| 0 | 0 |
| 0 | 0 |
| 0 | 0 |
| 0 | 0 |
| 1 | 0 |
| 0 | 0 |
| 0 | 0 |
| 0 | 0 |
| 0 | 0 |
| 0 | 0 |
| 0 | 0 |
| 0 | 0 |
| 0 | 3.1 |
| 0 | 1.6 |
| 0 | 0 |
| 0 | 8 |
| 0 | 4 |
| 0 | 12 |
| 0 | 2 |
| 0 | 4 |
| 0 | 3.2 |
| 0 | 4 |
| 0 | 4.8 |
| 0 | 12 |
| 0 | 8.4 |
| 0 | 8 |
| 0 | 3.6 |
| 0 | 7.2 |
| 0 | 6 |
| 0 | 3.6 |
| 0 | 10.8 |
| 0 | 4 |
| 0 | 4 |
| 0 | 6.4 |
| 0 | 0 |
| 0 |  |
| 0 |  |
| 0 |  |
| 0 |  |
| 0 |  |
| 0 |  |
| 0 |  |
| 0 |  |
| 0 |  |
| 0 |  |
| 0 |  |
| 0 |  |
| 0 |  |
| 0 |  |
| 0 |  |
| 0 |  |
| 0 |  |
| 0 |  |
| 0 |  |
| 0 |  |
| 0 |  |
| 0 |  |
| 0 |  |
| 0 |  |
| 0 |  |
| 0 |  |
| 0 |  |
| 0 |  |
| 0 |  |
| 0 |  |
| 0 |  |
| 0 |  |
| 0 |  |
| 0 |  |
| 0 |  |
| 2 |  |

Figure 2j

Serum level of NMB (μg/mL)

| Normal  (n=8) | Non-PNI  (n=18) | PNI  (n=20) |
| --- | --- | --- |
| 0.475747 | 0.592949 | 1.162893 |
| 0.685736 | 0.55167 | 1.407368 |
| 0.542249 | 0.5097 | 0.698268 |
| 0.589348 | 0.495895 | 1.334361 |
| 0.718795 | 0.329488 | 0.704194 |
| 0.602405 | 0.640853 | 0.720533 |
| 0.417926 | 0.566757 | 0.612742 |
| 0.665337 | 0.55167 | 0.655414 |
|  | 0.506535 | 0.88473 |
|  | 0.339409 | 0.861074 |
|  | 0.379379 | 0.604607 |
|  | 0.889206 | 0.666141 |
|  | 0.295497 | 0.786052 |
|  | 0.635826 | 0.740826 |
|  | 0.296099 | 0.668661 |
|  | 1.089168 | 1.352579 |
|  | 0.735477 | 0.678323 |
|  | 0.595879 | 0.534943 |
|  |  | 1.136179 |
|  |  | 1.678359 |

Figure 2l

Three days after tumor injection.

PBS


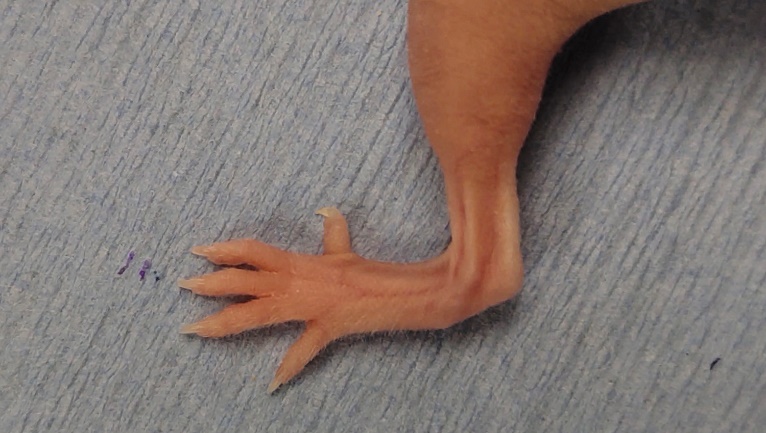


HeLa sgNC


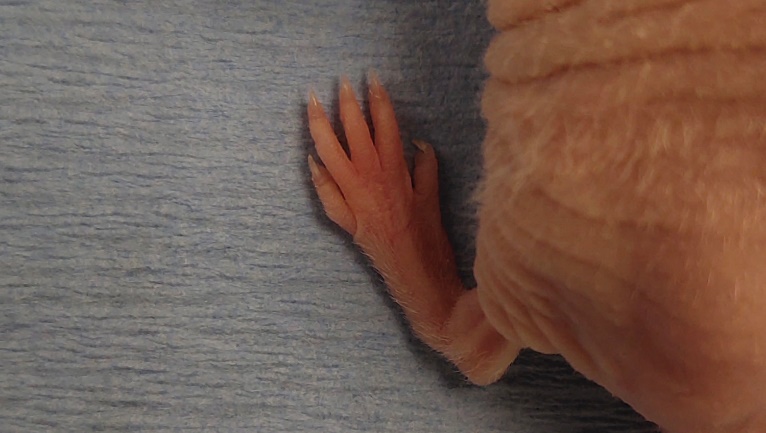


HeLa sgNC + NMB


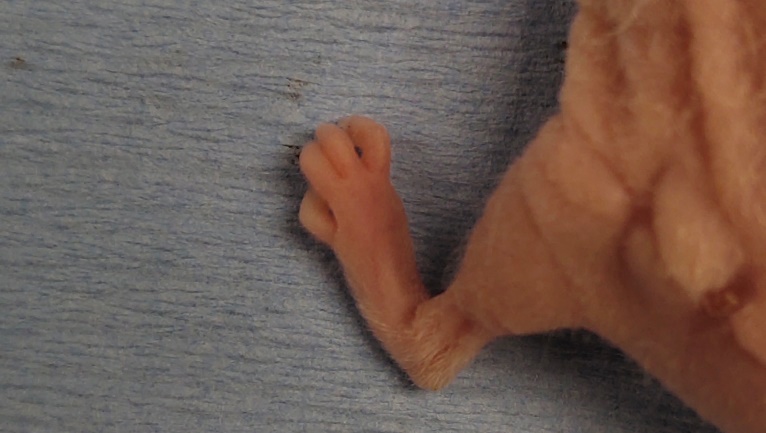


HeLa sgNMB


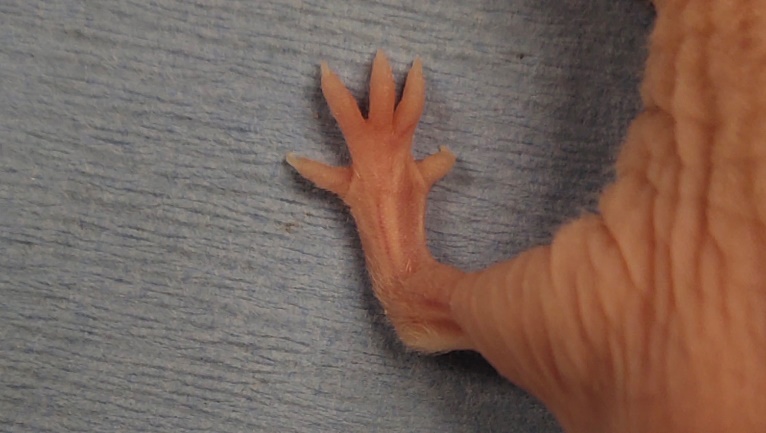


HeLa sgNMB+ NMB


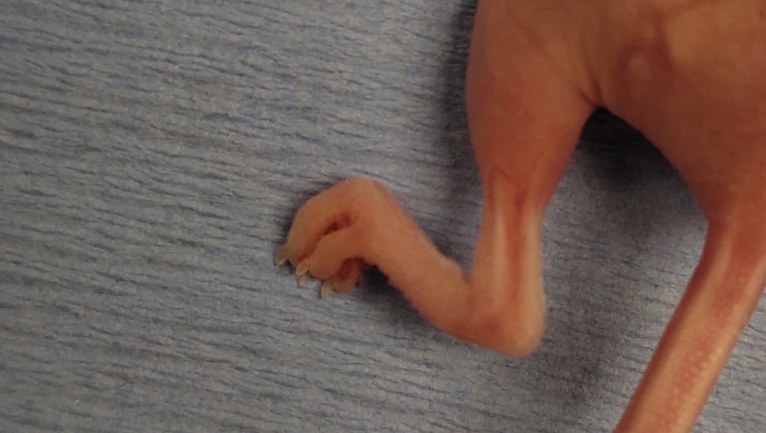


ME180 sgNC


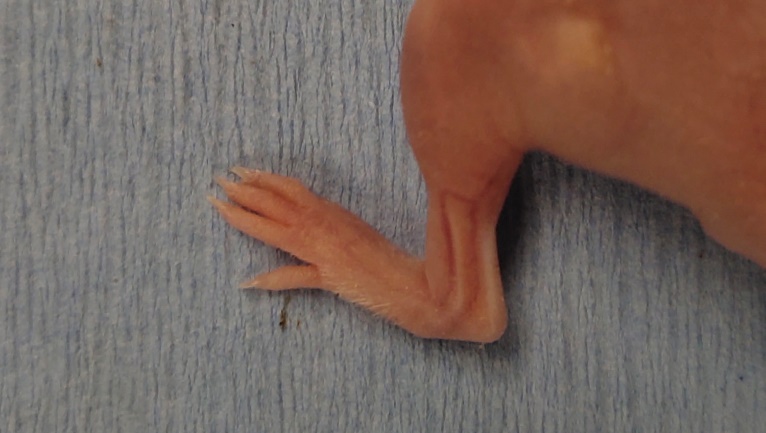


ME180 sgNC+ NMB


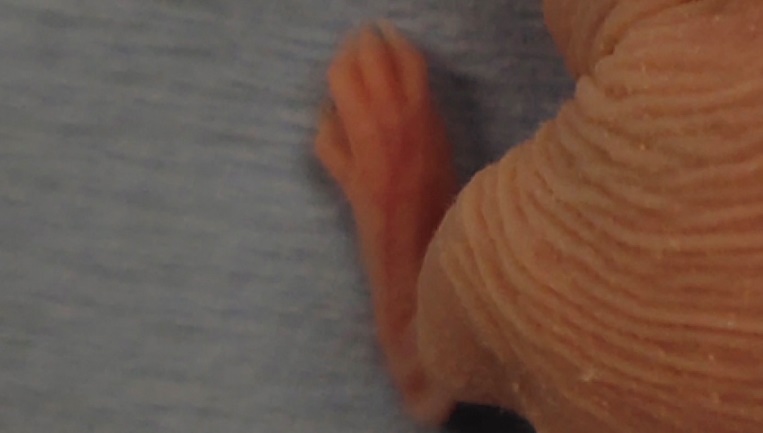


ME180 sgNMB


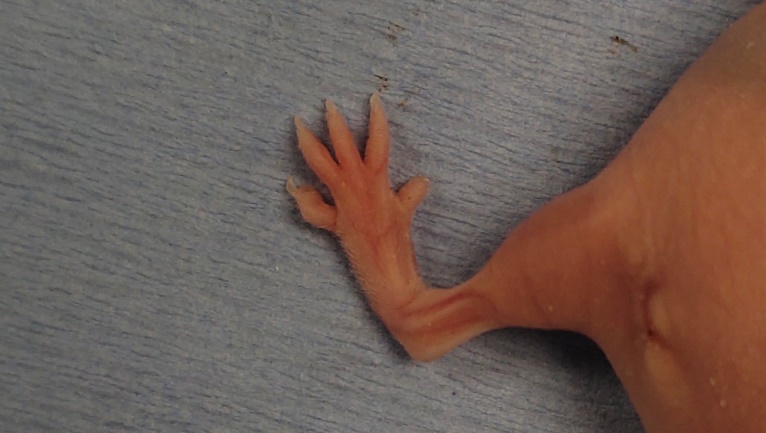


ME180 sgNMB+ NMB


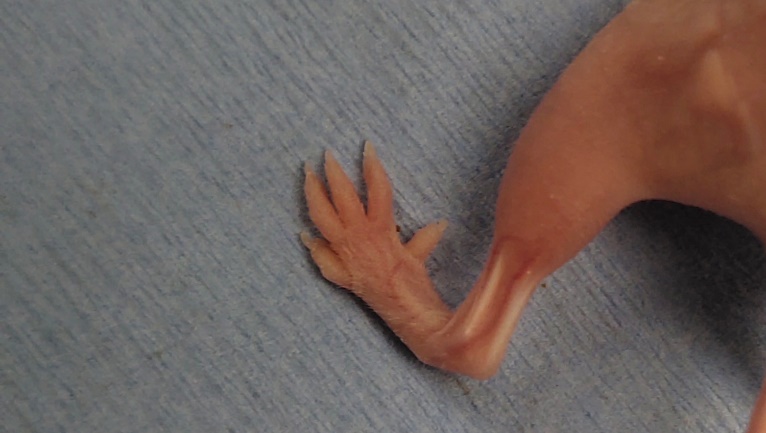


Figure 2m

| SFI | PBS (n=5) | HeLa sgNC (n=5) | HeLa sgNC+ NMB (n=5) | HeLa sgNMB (n=5) | HeLa sgNMB+ NMB (n=5) | ME180 sgNC (n=5) | ME180 sgNC+ NMB (n=5) | ME180 sgNMB (n=5) | ME180 sgNMB+ NMB (n=5) |
| --- | --- | --- | --- | --- | --- | --- | --- | --- | --- |
| 0 days | 10 | 10 | 10 | 10 | 10 | 10 | 10 | 10 | 10 |
|  | 10.5 | 11 | 10 | 10 | 10 | 10 | 10 | 10 | 10 |
|  | 10.5 | 10 | 10 | 10 | 10 | 10 | 10 | 10 | 10 |
|  | 10 | 10 | 11 | 10 | 10 | 10 | 10 | 10 | 10 |
|  | 10 | 10 | 10 | 10 | 10 | 10 | 10 | 10 | 10 |
| 3days | 10 | 6 | 7 | 9 | 5 | 7 | 7 | 9 | 5 |
|  | 10.5 | 7 | 8 | 9 | 6 | 7 | 7 | 10 | 5 |
|  | 10 | 6.5 | 8 | 10 | 8 | 6 | 5 | 10 | 3 |
|  | 10 | 7 | 5 | 10 | 6 | 6 | 8 | 10 | 7 |
|  | 9.5 | 4 | 5 | 10 | 3 | 5 | 8 | 9 | 9 |
| 1week | 10 | 6 | 2 | 10 | 6 | 7 | 7 | 10 | 6 |
|  | 10 | 2 | 0 | 10 | 2 | 7 | 6 | 11 | 3 |
|  | 10 | 7 | 4 | 9 | 6 | 6.5 | 6.5 | 10 | 5 |
|  | 11 | 7 | 3 | 10 | 4 | 7 | 6 | 11 | 0 |
|  | 10.5 | 7 | 1 | 8 | 3 | 7 | 5 | 10 | 6 |
| 2weeks | 10.5 | 4 | 0 | 8 | 2 | 5 | 0 | 9 | 0 |
|  | 10.5 | 5 | 0 | 10 | 3 | 5 | 2 | 11 | 4.5 |
|  | 10 | 5 | 0 | 9 | 2 | 5 | 2 | 10 | 4 |
|  | 10.5 | 4 | 0 | 10 | 0 | 3 | 0 | 10 | 0 |
|  | 10 | 5 | 2 | 10 | 0 | 3 | 2 | 10 | 3 |
| 5weeks | 10 | 2 | 1 | 10 | 2 | 2 | 0 | 8 | 0 |
|  | 10.5 | 2 | 0 | 10 | 0 | 1 | 0 | 7 | 0 |
|  | 10.5 | 1 | 0 | 10 | 0 | 2 | 0 | 10 | 0 |
|  | 10 | 1 | 0 | 8 | 2 | 2 | 0 | 9 | 0 |
|  | 10 | 1 | 0 | 8 | 0 | 1 | 0 | 10 | 0 |

Figure 2o

| Sciatic nerve score | PBS (n=5) | HeLa sgNC (n=5) | HeLa sgNC+ NMB (n=5) | HeLa sgNMB (n=5) | HeLa sgNMB+ NMB (n=5) | ME180 sgNC (n=5) | ME180 sgNC+ NMB (n=5) | ME180 sgNMB (n=5) | ME180 sgNMB+ NMB (n=5) |
| --- | --- | --- | --- | --- | --- | --- | --- | --- | --- |
| 0 days | 4 | 4 | 4 | 4 | 4 | 4 | 4 | 4 | 4 |
|  | 4 | 4 | 4 | 4 | 4 | 4 | 4 | 4 | 4 |
|  | 4 | 4 | 4 | 4 | 4 | 4 | 4 | 4 | 4 |
|  | 4 | 4 | 4 | 4 | 4 | 4 | 4 | 4 | 4 |
|  | 4 | 4 | 4 | 4 | 4 | 4 | 4 | 4 | 4 |
| 3days | 4 | 4 | 2 | 4 | 2 | 2 | 3 | 4 | 3 |
|  | 4 | 4 | 3 | 4 | 3 | 3 | 3 | 4 | 2 |
|  | 4 | 3 | 3 | 4 | 3 | 4 | 2 | 4 | 3 |
|  | 4 | 3 | 2 | 4 | 2 | 3 | 3 | 4 | 3 |
|  | 4 | 3 | 2 | 4 | 2 | 2 | 3 | 4 | 3 |
| 1week | 4 | 3 | 3 | 4 | 2 | 3 | 3 | 4 | 2 |
|  | 4 | 4 | 3 | 4 | 3 | 3 | 2 | 3 | 2 |
|  | 4 | 3 | 2 | 4 | 2 | 4 | 2 | 4 | 1 |
|  | 4 | 3 | 1 | 3 | 2 | 3 | 2 | 3 | 1 |
|  | 4 | 3 | 2 | 3 | 1 | 3 | 2 | 4 | 2 |
| 2weeks | 4 | 1 | 4 | 2 | 1 | 2 | 1 | 4 | 1 |
|  | 4 | 2 | 1 | 4 | 2 | 1 | 1 | 4 | 2 |
|  | 4 | 2 | 1 | 3 | 1 | 3 | 1 | 4 | 2 |
|  | 4 | 2 | 1 | 4 | 1 | 2 | 1 | 3 | 1 |
|  | 4 | 1 | 1 | 4 | 1 | 2 | 1 | 4 | 2 |
| 5weeks | 4 | 1 | 1 | 4 | 2 | 1 | 1 | 2 | 1 |
|  | 4 | 1 | 1 | 4 | 3 | 1 | 1 | 2 | 1 |
|  | 4 | 2 | 1 | 4 | 1 | 1 | 1 | 2 | 1 |
|  | 4 | 1 | 1 | 2 | 1 | 1 | 1 | 4 | 1 |
|  | 4 | 1 | 1 | 2 | 1 | 1 | 1 | 2 | 1 |

Figure 2q

| Length of PNI (mm) | HeLa sgNC (n=5) | HeLa sgNC+ NMB (n=5) | HeLa sgNMB (n=5) | HeLa sgNMB+ NMB (n=5) | ME180 sgNC (n=5) | ME180 sgNC+ NMB (n=5) | ME180 sgNMB (n=5) | ME180 sgNMB+ NMB (n=5) |
| --- | --- | --- | --- | --- | --- | --- | --- | --- |
| 3 days | 2.084 | 3.456 | 1.256 | 1.984 | 1.916 | 2.716 | 1.256 | 2.684 |
|  | 2.03 | 3.045 | 1.355 | 2.02 | 2.01 | 3.917 | 1.257 | 1.878 |
|  | 2.303 | 3.956 | 1.278 | 1.983 | 1.71 | 3.856 | 1.257 | 2.685 |
|  | 2.3 | 4.259 | 1.157 | 1.917 | 2.107 | 4.123 | 1.064 | 2.965 |
|  | 2.152 | 4.214 | 0.957 | 2.78 | 2.155 | 3.373 | 1.201 | 2.497 |

Figure 2r

| Weight of tumor (g) | HeLa sgNC (n=5) | HeLa sgNC+ NMB (n=5) | HeLa sgNMB (n=5) | HeLa sgNMB+ NMB (n=5) | ME180 sgNC (n=5) | ME180 sgNC+ NMB (n=5) | ME180 sgNMB (n=5) | ME180 sgNMB+ NMB (n=5) |
| --- | --- | --- | --- | --- | --- | --- | --- | --- |
| 3 days | 0.00614 | 0.00877 | 0.00876 | 0.00722 | 0.00904 | 0.00919 | 0.0094 | 0.00776 |
|  | 0.01163 | 0.008 | 0.00701 | 0.00663 | 0.00927 | 0.0086 | 0.00843 | 0.01161 |
|  | 0.00502 | 0.00612 | 0.00508 | 0.00658 | 0.00717 | 0.01365 | 0.0074 | 0.00918 |
|  | 0.00917 | 0.01223 | 0.00758 | 0.01225 | 0.01 | 0.01143 | 0.013 | 0.00766 |
|  | 0.00628 | 0.00712 | 0.00976 | 0.00999 | 0.012 | 0.01915 | 0.0076 | 0.00936 |

Figure S2a

|  | *18s* Ct value | *GADD4B* Ct value | 2^-∆∆Ct^ |
| --- | --- | --- | --- |
| HeLa | 12.71790441 | 27.776 | 0.871953258 |
| HeLa | 12.57726796 | 27.195 | 1.183055528 |
| HeLa | 10.92251428 | 25.827 | 0.969396988 |
|  |  |  |  |
| HeLa-RSC96 | 20.22136116 | 33.246 | 3.569332394 |
| HeLa-RSC96 | 12.36697038 | 26.217 | 2.014229174 |
| HeLa-RSC96 | 16.39669037 | 30.571 | 1.608516722 |
|  |  |  |  |
| ME180 | 13.21766281 | 28.095 | 0.881411823 |
| ME180 | 11.71585401 | 26.024 | 1.307475696 |
| ME180 | 12.05604839 | 26.956 | 0.867735776 |
|  |  |  |  |
| ME180-RSC96 | 11.05842463 | 25.660 | 1.066642413 |
| ME180-RSC96 | 11.83860175 | 26.508 | 1.018193218 |
| ME180-RSC96 | 11.50527827 | 26.092 | 1.077539157 |

|  | *18s* Ct value | *TGFB1* Ct value | 2^-∆∆Ct^ |
| --- | --- | --- | --- |
| HeLa | 12.71790441 | 24.639 | 1.713362174 |
| HeLa | 12.57726796 | 25.930 | 0.63504613 |
| HeLa | 13.34260082 | 26.162 | 0.91906357 |
|  |  |  |  |
| HeLa-RSC96 | 20.22136116 | 30.450 | 5.538590085 |
| HeLa-RSC96 | 12.36697038 | 25.120 | 0.962400679 |
| HeLa-RSC96 | 12.56604067 | 23.961 | 2.4669407 |
|  |  |  |  |
| ME180 | 13.21766281 | 24.945 | 0.800755976 |
| ME180 | 11.4921519 | 23.110 | 0.864092957 |
| ME180 | 12.05604839 | 22.932 | 1.445237915 |
|  |  |  |  |
| ME180-RSC96 | 12.9254144 | 23.585 | 1.678145264 |
| ME180-RSC96 | 10.07532056 | 20.448 | 2.047738281 |
| ME180-RSC96 | 11.83860175 | 21.816 | 2.694012581 |

|  | *18s* Ct value | *SERPINE1* Ct value | 2^-∆∆Ct^ |
| --- | --- | --- | --- |
| HeLa | 12.71790441 | 24.531 | 1.275062182 |
| HeLa | 12.57726796 | 25.048 | 0.808093424 |
| HeLa | 11.58690802 | 23.793 | 0.97052575 |
|  |  |  |  |
| HeLa-RSC96 | 20.22136116 | 30.934 | 2.734067079 |
| HeLa-RSC96 | 12.56604067 | 23.566 | 2.239041582 |
| HeLa-RSC96 | 11.4891332 | 22.347 | 2.471079721 |
|  |  |  |  |
| ME180 | 13.33663114 | 28.803 | 1.112634076 |
| ME180 | 12.05604839 | 28.299 | 0.649765305 |
| ME180 | 11.48151875 | 26.634 | 1.383219572 |
|  |  |  |  |
| ME180-RSC96 | 12.9254144 | 28.633 | 0.941245942 |
| ME180-RSC96 | 12.50801945 | 26.129 | 3.998152756 |
| ME180-RSC96 | 11.83860175 | 25.970 | 2.80790023 |

|  | *18s* Ct value | *TMEM158* Ct value | 2^-∆∆Ct^ |
| --- | --- | --- | --- |
| HeLa | 12.71790441 | 26.784 | 1.701884937 |
| HeLa | 13.34260082 | 28.300 | 0.917552255 |
| HeLa | 11.58690802 | 27.063 | 0.640381827 |
|  |  |  |  |
| HeLa-RSC96 | 20.22136116 | 32.609 | 5.446812126 |
| HeLa-RSC96 | 12.56604067 | 25.668 | 3.321924334 |
| HeLa-RSC96 | 11.4891332 | 25.263 | 2.083757453 |
|  |  |  |  |
| ME180 | 13.21766281 | 27.128 | 1.416460536 |
| ME180 | 12.22063573 | 26.651 | 0.987804156 |
| ME180 | 11.48151875 | 26.379 | 0.714701464 |
|  |  |  |  |
| ME180-RSC96 | 11.50527827 | 25.916 | 1.001369944 |
| ME180-RSC96 | 11.83860175 | 26.224 | 1.018882967 |
| ME180-RSC96 | 11.06636588 | 26.075 | 0.661555674 |

|  | *18s* Ct value | *Fosl1* Ct value | 2^-∆∆Ct^ |
| --- | --- | --- | --- |
| HeLa | 12.71790441 | 26.995 | 0.981328521 |
| HeLa | 12.57726796 | 26.838 | 0.992525236 |
| HeLa | 10.92251428 | 25.134 | 1.026701084 |
|  |  |  |  |
| HeLa-RSC96 | 12.56604067 | 25.820 | 1.99390295 |
| HeLa-RSC96 | 11.46402359 | 24.470 | 2.367941048 |
| HeLa-RSC96 | 10.12679513 | 23.605 | 1.706807328 |
|  |  |  |  |
| ME180 | 13.21766281 | 26.818 | 1.102050501 |
| ME180 | 13.33663114 | 27.374 | 0.814095984 |
| ME180 | 11.71585401 | 25.300 | 1.114609888 |
|  |  |  |  |
| ME180-RSC96 | 11.83860175 | 24.170 | 2.656293977 |
| ME180-RSC96 | 11.06636588 | 23.163 | 3.12443075 |
| ME180-RSC96 | 12.50801945 | 24.432 | 3.523072163 |

|  | *18s* Ct value | *ITGA5* Ct value | 2^-∆∆Ct^ |
| --- | --- | --- | --- |
| HeLa | 12.71790441 | 24.288 | 1.019095297 |
| HeLa | 12.57726796 | 23.864 | 1.240619853 |
| HeLa | 13.34260082 | 25.279 | 0.790945347 |
|  |  |  |  |
| HeLa-RSC96 | 12.56604067 | 22.973 | 2.283059922 |
| HeLa-RSC96 | 11.46402359 | 22.028 | 2.047447873 |
| HeLa-RSC96 | 10.12679513 | 20.643 | 2.116423549 |
|  |  |  |  |
| ME180 | 13.21766281 | 28.924 | 0.913138162 |
| ME180 | 12.05604839 | 27.665 | 0.976769813 |
| ME180 | 13.33663114 | 28.747 | 1.121169507 |
|  |  |  |  |
| ME180-RSC96 | 12.9254144 | 27.203 | 2.458181105 |
| ME180-RSC96 | 11.83860175 | 25.989 | 2.684270438 |
| ME180-RSC96 | 12.50801945 | 26.906 | 2.261171812 |

|  | *18s* Ct value | *Loxl2* Ct value | 2^-∆∆Ct^ |
| --- | --- | --- | --- |
| HeLa | 12.57726796 | 23.682 | 1.172006863 |
| HeLa | 10.92251428 | 22.427 | 0.888451994 |
| HeLa | 10.81333192 | 21.986 | 1.118315333 |
|  |  |  |  |
| HeLa-RSC96 | 12.36697038 | 22.410 | 2.44567924 |
| HeLa-RSC96 | 12.56604067 | 22.853 | 2.06609073 |
| HeLa-RSC96 | 11.4891332 | 21.826 | 1.995530225 |
|  |  |  |  |
| ME180 | 12.05604839 | 29.852 | 1.081301869 |
| ME180 | 11.48151875 | 29.268 | 1.088558734 |
| ME180 | 13.33663114 | 31.481 | 0.849573935 |
|  |  |  |  |
| ME180-RSC96 | 12.9254144 | 28.318 | 5.721572372 |
| ME180-RSC96 | 11.06636588 | 26.359 | 6.130038433 |
| ME180-RSC96 | 12.50801945 | 27.215 | 9.200637334 |

|  | *18s* Ct value | *GDF15* Ct value | 2^-∆∆Ct^ |
| --- | --- | --- | --- |
| HeLa | 13.19725704 | 28.105 | 1.518771853 |
| HeLa | 12.57726796 | 28.482 | 0.760492263 |
| HeLa | 13.34260082 | 29.061 | 0.865790188 |
|  |  |  |  |
| HeLa-RSC96 | 18.2813549 | 33.005 | 1.724934802 |
| HeLa-RSC96 | 12.36697038 | 27.298 | 1.494064675 |
| HeLa-RSC96 | 12.56604067 | 28.800 | 0.605477875 |
|  |  |  |  |
| ME180 | 11.19839287 | 26.591 | 0.546077777 |
| ME180 | 11.74248648 | 25.863 | 1.319672717 |
| ME180 | 12.05604839 | 26.104 | 1.387647824 |
|  |  |  |  |
| ME180-RSC96 | 10.79187616 | 27.437 | 0.229211386 |
| ME180-RSC96 | 10.37853909 | 25.189 | 0.817634946 |
| ME180-RSC96 | 11.83860175 | 25.624 | 1.663785643 |

|  | *18s* Ct value | *ECM1* Ct value | 2^-∆∆Ct^ |
| --- | --- | --- | --- |
| HeLa | 13.19725704 | 28.670 | 0.859858736 |
| HeLa | 12.57726796 | 26.884 | 1.929676719 |
| HeLa | 10.92251428 | 26.908 | 0.602682158 |
|  |  |  |  |
| HeLa-RSC96 | 18.2813549 | 33.171 | 1.288652967 |
| HeLa-RSC96 | 12.56604067 | 28.566 | 0.596937894 |
| HeLa-RSC96 | 16.39669037 | 31.183 | 1.384110546 |
|  |  |  |  |
| ME180 | 11.19839287 | 29.885 | 0.253489261 |
| ME180 | 12.05604839 | 27.308 | 2.739823013 |
| ME180 | 11.71585401 | 27.896 | 1.439852234 |
|  |  |  |  |
| ME180-RSC96 | 10.79187616 | 27.928 | 0.742559465 |
| ME180-RSC96 | 11.83860175 | 26.755 | 3.4595372 |
| ME180-RSC96 | 11.05842463 | 26.948 | 1.761473979 |

|  | *18s* Ct value | *ANGPTL4* Ct value | 2^-∆∆Ct^ |
| --- | --- | --- | --- |
| HeLa | 13.19725704 | 33.053 | 1.066611546 |
| HeLa | 12.57726796 | 32.583 | 0.960723893 |
| HeLa | 11.58690802 | 31.570 | 0.975877102 |
|  |  |  |  |
| HeLa-RSC96 | 18.2813549 | 37.822 | 1.326829443 |
| HeLa-RSC96 | 12.56604067 | 31.810 | 1.629535258 |
| HeLa-RSC96 | 11.46402359 | 30.278 | 2.194897837 |
|  |  |  |  |
| ME180 | 12.05604839 | 32.910 | 0.864464378 |
| ME180 | 13.33663114 | 34.824 | 0.557298436 |
| ME180 | 12.22063573 | 31.811 | 2.075702335 |
|  |  |  |  |
| ME180-RSC96 | 11.83860175 | 29.556 | 7.59912986 |
| ME180-RSC96 | 11.06636588 | 29.356 | 5.112718463 |
| ME180-RSC96 | 12.50801945 | 32.019 | 2.192193722 |

|  | *18s* Ct value | *MT1X* Ct value | 2^-∆∆Ct^ |
| --- | --- | --- | --- |
| HeLa | 13.19725704 | 28.854 | 0.859192516 |
| HeLa | 13.34260082 | 28.987 | 0.866293148 |
| HeLa | 11.58690802 | 26.598 | 1.343521545 |
|  |  |  |  |
| HeLa-RSC96 | 18.2813549 | 33.107 | 1.528531712 |
| HeLa-RSC96 | 12.36697038 | 26.687 | 2.169515565 |
| HeLa-RSC96 | 11.46402359 | 25.571 | 2.514950967 |
|  |  |  |  |
| ME180 | 12.05604839 | 25.156 | 0.486701834 |
| ME180 | 11.48151875 | 23.135 | 1.325852635 |
| ME180 | 12.22063573 | 23.649 | 1.549679048 |
|  |  |  |  |
| ME180-RSC96 | 11.83860175 | 22.558 | 2.534097982 |
| ME180-RSC96 | 11.06636588 | 22.372 | 1.687322365 |
| ME180-RSC96 | 12.50801945 | 23.445 | 2.179770646 |

Figure S2b

NMB concentration in the cell supernatant (ng/L)

| HeLa | HeLa-RSC96 | ME180 | ME180-RSC96 |
| --- | --- | --- | --- |
| 43.96624 | 67.584075 | 51.683072 | 66.469041 |
| 45.059699 | 84.810674 | 47.029128 | 64.68567 |
| 53.205135 | 74.587271 | 58.604936 | 72.973115 |

Figure S2c

NMB


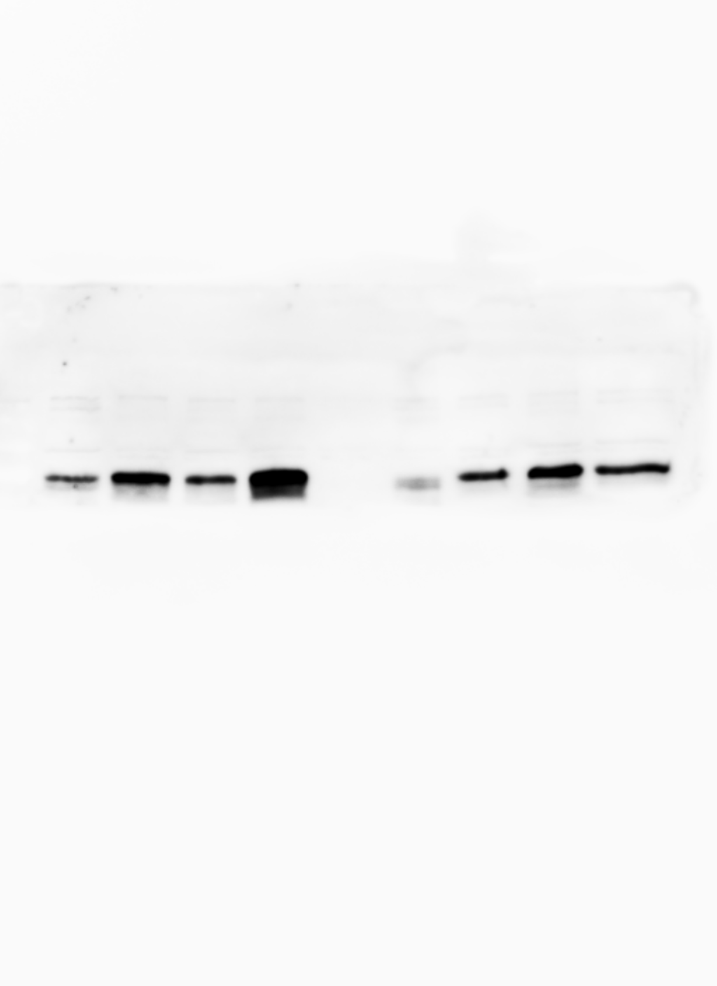


GAPDH


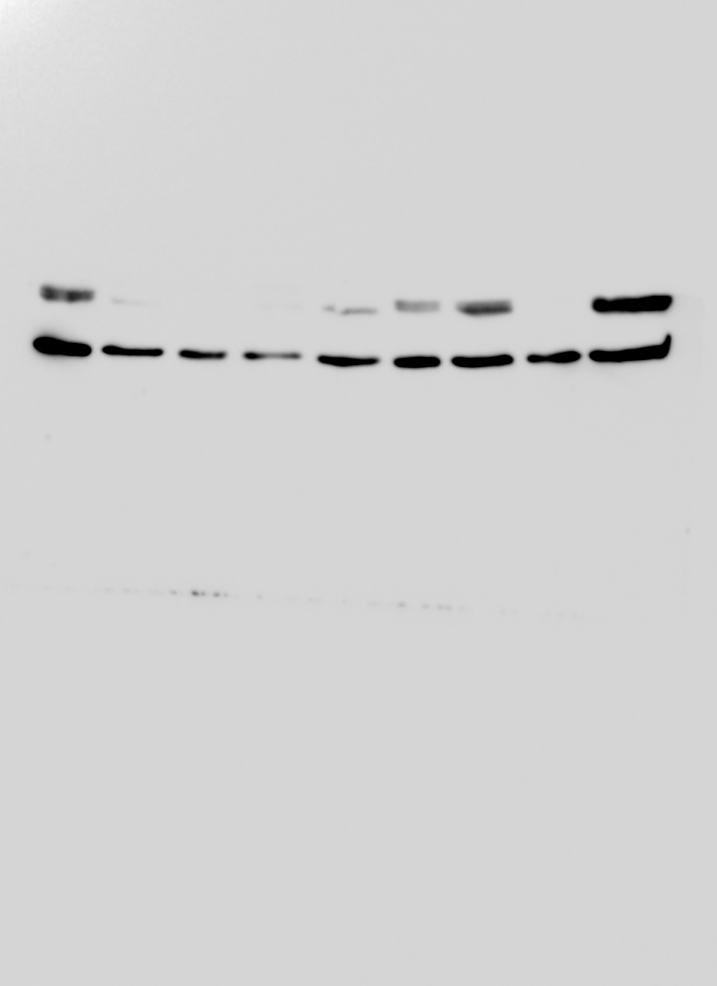


figure S2d

NMB concentration in the cell supernatant (ng/L)

| HeLa | HeLa-DRG | ME180 | ME180-DRG |
| --- | --- | --- | --- |
| 42.815822 | 51.71123 | 54.105382 | 81.880494 |
| 33.210429 | 51.76052 | 51.205314 | 72.519025 |
| 37.516624 | 63.710358 | 52.934836 | 67.219271 |

figure S2e HeLa

| HeLa | *18s* Ct value | *NMB* Ct value | 2^-∆∆Ct^ |
| --- | --- | --- | --- |
| Ctrl | 8.217 | 22.870 | 1.369214049 |
| Ctrl | 8.110 | 23.218 | 0.998985713 |
| Ctrl | 8.104 | 22.996 | 1.160411617 |
|  |  |  |  |
| Serotonin-5μM | 6.590 | 22.489 | 0.577260838 |
| Serotonin-5μM | 6.889 | 22.713 | 0.608115647 |
| Serotonin-5μM | 6.944 | 22.739 | 0.620376198 |
| Serotonin-10μM | 7.233 | 23.087 | 0.595568044 |
| Serotonin-10μM | 7.083 | 23.109 | 0.528607114 |
| Serotonin-10μM | 7.369 | 23.286 | 0.570342418 |
| Serotonin-25μM | 7.706 | 24.649 | 0.279957615 |
| Serotonin-25μM | 7.827 | 24.498 | 0.338104023 |
| Serotonin-25μM | 7.703 | 24.780 | 0.255107045 |
|  |  |  |  |
| Dopamine-1μM | 7.439 | 22.795 | 0.841248536 |
| Dopamine-1μM | 7.622 | 22.661 | 1.047633589 |
| Dopamine-1μM | 7.981 | 22.970 | 1.085171887 |
| Dopamine-5μM | 7.764 | 23.370 | 0.707505294 |
| Dopamine-5μM | 7.705 | 23.263 | 0.731450318 |
| Dopamine-5μM | 7.670 | 23.190 | 0.75074959 |
| Dopamine-10μM | 7.400 | 23.389 | 0.542422415 |
| Dopamine-10μM | 7.349 | 23.279 | 0.564870231 |
| Dopamine-10μM | 7.680 | 23.488 | 0.615154256 |
|  |  |  |  |
| Leptin-50ng/mL | 7.770 | 23.216 | 0.790187645 |
| Leptin-50ng/mL | 7.430 | 23.134 | 0.660753183 |
| Leptin-50ng/mL | 7.324 | 22.958 | 0.693443954 |
| Leptin-100ng/mL | 7.320 | 22.920 | 0.710072397 |
| Leptin-100ng/mL | 7.428 | 23.140 | 0.657410673 |
| Leptin-100ng/mL | 7.275 | 22.865 | 0.71523718 |
| Leptin-500ng/mL | 7.327 | 23.428 | 0.501862737 |
| Leptin-500ng/mL | 7.431 | 23.155 | 0.651636376 |
| Leptin-500ng/mL | 7.461 | 22.961 | 0.761110405 |
|  |  |  |  |
| Lecirelin-1μM | 7.868 | 22.777 | 1.146977582 |
| Lecirelin-1μM | 7.792 | 22.927 | 0.980010036 |
| Lecirelin-1μM | 7.782 | 22.859 | 1.02090013 |
| Lecirelin-2μM | 7.277 | 23.121 | 0.599916227 |
| Lecirelin-2μM | 7.435 | 23.148 | 0.657007296 |
| Lecirelin-2μM | 7.424 | 23.223 | 0.619059155 |
| Lecirelin-5μM | 7.494 | 23.298 | 0.616839234 |
| Lecirelin-5μM | 7.739 | 23.202 | 0.781160499 |
| Lecirelin-5μM | 7.568 | 23.169 | 0.709546412 |
|  |  |  |  |
| Aldosterone-0.1μM | 7.849 | 23.560 | 0.657665382 |
| Aldosterone-0.1μM | 7.697 | 23.438 | 0.644340255 |
| Aldosterone-0.1μM | 7.502 | 23.349 | 0.59867032 |
| Aldosterone-1μM | 7.736 | 23.437 | 0.662124352 |
| Aldosterone-1μM | 7.893 | 23.638 | 0.641970476 |
| Aldosterone-1μM | 7.886 | 23.550 | 0.679147844 |
| Aldosterone-2μM | 7.909 | 23.296 | 0.823192657 |
| Aldosterone-2μM | 8.034 | 23.347 | 0.866671404 |
| Aldosterone-2μM | 7.981 | 23.306 | 0.859626008 |
|  |  |  |  |
| Meprednison-1μM | 8.265 | 23.715 | 0.788043468 |
| Meprednison-1μM | 8.151 | 23.476 | 0.859444757 |
| Meprednison-1μM | 7.874 | 23.705 | 0.604720133 |
| Meprednison-5μM | 7.650 | 23.004 | 0.842734345 |
| Meprednison-5μM | 8.187 | 23.139 | 1.112880452 |
| Meprednison-5μM | 7.986 | 23.002 | 1.064698452 |
| Meprednison-10μM | 7.484 | 23.234 | 0.64000528 |
| Meprednison-10μM | 7.767 | 23.274 | 0.757609207 |
| Meprednison-10μM | 7.792 | 23.321 | 0.746176277 |
|  |  |  |  |
| Estradiol-100nM | 8.130 | 23.365 | 0.915049906 |
| Estradiol-100nM | 8.141 | 23.297 | 0.966357609 |
| Estradiol-100nM | 8.442 | 23.333 | 1.160853536 |
| Estradiol-1μM | 8.490 | 23.054 | 1.456432004 |
| Estradiol-1μM | 8.437 | 22.894 | 1.568247813 |
| Estradiol-1μM | 8.917 | 23.476 | 1.460979441 |
| Estradiol-10μM | 9.906 | 23.212 | 3.481792622 |
| Estradiol-10μM | 8.022 | 21.920 | 2.310987227 |
| Estradiol-10μM | 9.557 | 23.176 | 2.802910969 |
|  |  |  |  |
| L-Glutamic acid-5mM | 8.218 | 23.345 | 0.986100601 |
| L-Glutamic acid-5mM | 8.197 | 23.129 | 1.128734597 |
| L-Glutamic acid-5mM | 8.374 | 23.229 | 1.190311194 |
| L-Glutamic acid-10mM | 8.244 | 22.837 | 1.427487508 |
| L-Glutamic acid-10mM | 8.417 | 23.000 | 1.437423348 |
| L-Glutamic acid-10mM | 8.369 | 22.956 | 1.433856493 |
| L-Glutamic acid-20mM | 8.158356667 | 22.88944435 | 1.297047742 |
| L-Glutamic acid-20mM | 8.524521828 | 23.00896645 | 1.538873508 |
| L-Glutamic acid-20mM | 8.216262817 | 22.91061401 | 1.330499596 |
|  |  |  |  |
| Triiodothyronine-50nM | 8.243 | 22.932 | 1.336044108 |
| Triiodothyronine-50nM | 8.550 | 23.228 | 1.34561296 |
| Triiodothyronine-50nM | 8.292 | 23.047 | 1.275674294 |
| Triiodothyronine-100nM | 8.541 | 23.238 | 1.328102457 |
| Triiodothyronine-100nM | 8.547 | 23.261 | 1.312734449 |
| Triiodothyronine-100nM | 8.380 | 23.040 | 1.362896584 |
| Triiodothyronine-1μM | 9.510 | 23.255 | 2.570053029 |
| Triiodothyronine-1μM | 9.860 | 23.733 | 2.350028502 |
| Triiodothyronine-1μM | 8.489 | 22.895 | 1.62419301 |
|  |  |  |  |
| γ-GABA-1μM | 8.444 | 23.560 | 0.993315453 |
| γ-GABA-1μM | 8.286 | 23.533 | 0.906970094 |
| γ-GABA-1μM | 8.570 | 23.456 | 1.164525811 |
| γ-GABA-5μM | 8.637 | 23.286 | 1.373454664 |
| γ-GABA-5μM | 8.748 | 23.387 | 1.383065613 |
| γ-GABA-5μM | 8.593 | 23.246 | 1.368563437 |
| γ-GABA-10μM | 9.182 | 23.332 | 1.939631692 |
| γ-GABA-10μM | 8.985 | 23.135 | 1.94054481 |
| γ-GABA-10μM | 8.961 | 23.272 | 1.735992782 |

figure 2f ME180

| ME180 | *18s* Ct value | *NMB* Ct value | 2^-∆∆Ct^ |
| --- | --- | --- | --- |
| Ctrl | 6.863 | 22.581 | 1.201834139 |
| Ctrl | 6.060 | 21.801 | 1.183046664 |
| Ctrl | 6.042 | 22.135 | 0.926502841 |
|  |  |  |  |
| Serotonin-5μM | 6.288 | 23.055 | 0.580706029 |
| Serotonin-5μM | 5.698 | 22.578 | 0.537216036 |
| Serotonin-5μM | 5.630 | 22.574 | 0.513952079 |
| Serotonin-10μM | 6.132 | 23.350 | 0.425004534 |
| Serotonin-10μM | 6.632 | 23.949 | 0.396832775 |
| Serotonin-10μM | 6.654 | 23.861 | 0.42832173 |
| Serotonin-25μM | 6.235 | 22.431 | 0.862819587 |
| Serotonin-25μM | 6.267 | 22.360 | 0.927481751 |
| Serotonin-25μM | 6.281 | 22.717 | 0.730526018 |
|  |  |  |  |
| Dopamine-1μM | 6.235 | 22.431 | 1.219156176 |
| Dopamine-1μM | 6.339 | 22.318 | 1.002954231 |
| Dopamine-1μM | 6.267 | 22.360 | 0.979467419 |
| Dopamine-5μM | 5.786 | 21.992 | 0.857011334 |
| Dopamine-5μM | 5.785 | 22.001 | 0.851264969 |
| Dopamine-5μM | 6.312 | 22.602 | 0.808826146 |
| Dopamine-10μM | 5.854 | 22.547 | 0.611484009 |
| Dopamine-10μM | 5.961 | 22.645 | 0.615218983 |
| Dopamine-10μM | 5.900 | 22.595 | 0.610982788 |
|  |  |  |  |
| Leptin-50ng/mL | 6.365 | 22.218 | 1.094731513 |
| Leptin-50ng/mL | 6.034 | 21.919 | 1.070349582 |
| Leptin-50ng/mL | 6.181 | 21.835 | 1.256784407 |
| Leptin-100ng/mL | 6.359 | 21.936 | 1.325296653 |
| Leptin-100ng/mL | 6.475 | 22.259 | 1.148345927 |
| Leptin-100ng/mL | 6.467 | 22.078 | 1.294248083 |
| Leptin-500ng/mL | 6.464 | 22.423 | 1.017016786 |
| Leptin-500ng/mL | 5.962 | 21.920 | 1.017422927 |
| Leptin-500ng/mL | 6.411 | 22.403 | 0.994034163 |
|  |  |  |  |
| Lecirelin-1μM | 5.860 | 21.881 | 0.974369973 |
| Lecirelin-1μM | 6.393 | 22.504 | 0.91525569 |
| Lecirelin-1μM | 6.609 | 22.272 | 1.249253603 |
| Lecirelin-2μM | 6.396 | 22.891 | 0.701630264 |
| Lecirelin-2μM | 5.668 | 22.013 | 0.778313327 |
| Lecirelin-2μM | 6.354 | 22.901 | 0.676505216 |
| Lecirelin-5μM | 5.863 | 21.986 | 0.907720529 |
| Lecirelin-5μM | 6.622 | 22.527 | 1.055908289 |
| Lecirelin-5μM | 6.674 | 22.472 | 1.137205508 |
|  |  |  |  |
| Aldosterone-0.1μM | 6.379 | 22.801 | 0.737552047 |
| Aldosterone-0.1μM | 6.477 | 22.571 | 0.926331371 |
| Aldosterone-0.1μM | 6.598 | 22.464 | 1.08439469 |
| Aldosterone-1μM | 5.901 | 22.294 | 0.7525548 |
| Aldosterone-1μM | 6.600 | 22.703 | 0.920672344 |
| Aldosterone-1μM | 5.906 | 22.238 | 0.785449717 |
| Aldosterone-2μM | 6.195 | 22.655 | 0.718269338 |
| Aldosterone-2μM | 5.773 | 22.246 | 0.712402086 |
| Aldosterone-2μM | 5.469 | 22.112 | 0.632831091 |
|  |  |  |  |
| Meprednison-1μM | 5.739 | 21.955 | 0.851183098 |
| Meprednison-1μM | 6.256 | 21.958 | 1.215729048 |
| Meprednison-1μM | 6.916 | 22.566 | 1.259705914 |
| Meprednison-5μM | 6.333 | 22.301 | 1.01088962 |
| Meprednison-5μM | 6.878 | 22.724 | 1.099976808 |
| Meprednison-5μM | 6.621 | 22.322 | 1.216483499 |
| Meprednison-10μM | 5.744 | 21.901 | 0.886531255 |
| Meprednison-10μM | 6.519 | 22.514 | 0.992133698 |
| Meprednison-10μM | 6.544 | 22.539 | 0.992004834 |
|  |  |  |  |
| Estradiol-100nM | 6.994 | 22.361 | 1.532875474 |
| Estradiol-100nM | 7.015 | 22.389 | 1.525627397 |
| Estradiol-100nM | 6.818 | 22.174 | 1.544481809 |
| Estradiol-1μM | 6.611 | 21.712 | 1.843084275 |
| Estradiol-1μM | 6.745 | 21.747 | 1.974568397 |
| Estradiol-1μM | 7.178 | 22.107 | 2.077035468 |
| Estradiol-10μM | 6.934 | 21.824 | 2.134082588 |
| Estradiol-10μM | 7.923 | 22.627 | 2.427069356 |
| Estradiol-10μM | 7.926 | 22.347 | 2.95454373 |
|  |  |  |  |
| L-Glutamic acid-5mM | 7.026 | 21.779 | 2.34753058 |
| L-Glutamic acid-5mM | 6.976 | 21.943 | 2.022878786 |
| L-Glutamic acid-5mM | 7.435 | 22.275 | 2.209054909 |
| L-Glutamic acid-10mM | 7.040 | 21.405 | 3.07113318 |
| L-Glutamic acid-10mM | 7.472 | 21.998 | 2.746606407 |
| L-Glutamic acid-10mM | 7.472 | 21.971 | 2.799341303 |
| L-Glutamic acid-20mM |  |  |  |
| L-Glutamic acid-20mM |  |  |  |
| L-Glutamic acid-20mM |  |  |  |
|  |  |  |  |
| Triiodothyronine-50nM | 6.129 | 21.964 | 1.108343867 |
| Triiodothyronine-50nM | 6.193 | 22.301 | 0.917257762 |
| Triiodothyronine-50nM | 5.988 | 21.984 | 0.991570821 |
| Triiodothyronine-100nM | 6.354 | 21.923 | 1.332734251 |
| Triiodothyronine-100nM | 6.287 | 21.948 | 1.251042332 |
| Triiodothyronine-100nM | 7.315 | 22.714 | 1.498795606 |
| Triiodothyronine-1μM | 6.669 | 22.008 | 1.563312927 |
| Triiodothyronine-1μM | 7.638 | 22.828 | 1.733518888 |
| Triiodothyronine-1μM | 7.544 | 22.704 | 1.770518039 |
|  |  |  |  |
| γ-GABA-1μM | 6.651 | 21.731 | 1.869476869 |
| γ-GABA-1μM | 7.355 | 22.529 | 1.752597823 |
| γ-GABA-1μM | 7.395 | 22.450 | 1.903797111 |
| γ-GABA-5μM | 7.114 | 22.223 | 1.833875945 |
| γ-GABA-5μM | 7.708 | 22.700 | 1.987440901 |
| γ-GABA-5μM | 7.811 | 22.736 | 2.083359487 |
| γ-GABA-10μM | 7.191 | 21.693 | 2.792346962 |
| γ-GABA-10μM | 7.575 | 22.271 | 2.441824604 |
| γ-GABA-10μM | 7.769 | 22.379 | 2.592031982 |

Figure S3a

| **测序结果** | **H_NMB sgRNA1-ZsGreen1-T2A-Puro(lenti-SpCas9)** |
| --- | --- |
| CCCAATTTGAAATACGATACAGGCTGTTAGAGAGATAATTAGAATTAATTTGACTGTAAACACAAAGATATTAGTACAAAATACGTGACGTAGAAAGTAATAATTTCTTGGGTAGTTTGCAGTTTTAAAATTATGTTTTAAAATGGACTATCATATGCTTACCGTAACTTGAAAGTATTTCGATTTCTTGGCTTTATATATCTTGTGGAAAGGACGAAACACCGCGGGGGGCGCTCGGATGTTGTTTTAGAGCTAGAAATAGCAAGTTAAAATAAGGCTAGTCCGTTATCAACTTGAAAAAGTGGCACCGAGTCGGTGCTTTTTTAAGCTTGGCGTAACTAGATCTTGAGACAAATGGCAGTATTCATCCACAATTTTAAAAGAAAAGGGGGGATTGGGGGGTACAGTGCAGGGGAAAGAATAGTAGACATAATAGCAACAGACATACAAACTAAAGAATTACAAAAACAAATTACAAAAATTCAAAATTTTCGGGTTTATTACAGGGACAGCAGAGATCCACTTTGGCGCCGGCTCGAGGTAATCAATTACGGGGTCATTAGTTCATAGCCCATATATGGAGTTCCGCGTTACATAACTTACGGTAAATGGCCCGCCTGGCTGACCGCCCAACGACCCCCGCCCATTGACGTCAATAATGACGTATGTTCCCATAGTAACGCCAATAGGGACTTTCCATTGACGTCAATGGGTGGAGTATTTACGGTAAACTGCCCACTTGGCAGTACATCAAGTGTATCATATGCCAAGTACGCCCCCTATTGACGTCAATGACGGTAAATGGCCCGCCTGGCATTATGCCCAGTACATGACCTTATGGGACTTTCCTACTTGGCAGTACATCTACGTATTAGTCATCGCTATTACCATGGTGATGCGGTTTTGGCAGTACATCAATGGGCGTGGATAGCGGTTTGACTCACGGGGATTTCCAAGTCTCCACCCCATTGACGTCAATGGGAGTTTGTTTGGCACCAAATCAACGGGACTTTCCAAATGTCGTAACAACTCCGCCCCATTGACGCAAATGGGCGGTAGGCGTTGTACGGTGGGA | |
| 图例 | 引物  靶位点 |
| 测序引物 | HU6_F：GAGGGCCTATTTCCCATGATT |
| 测序结果 | 0155_31121121004127_(47674GH-101)_[hU6-F] |


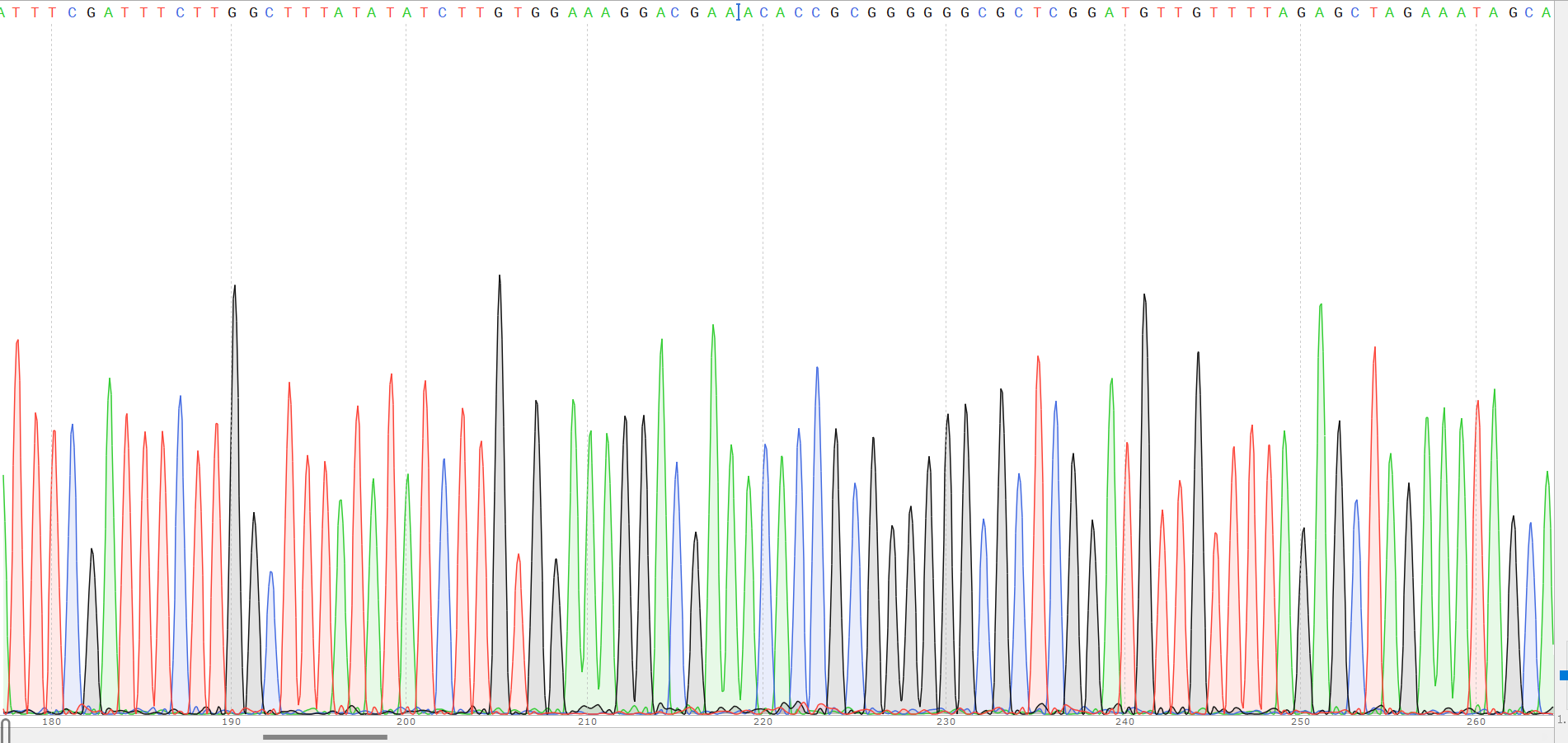


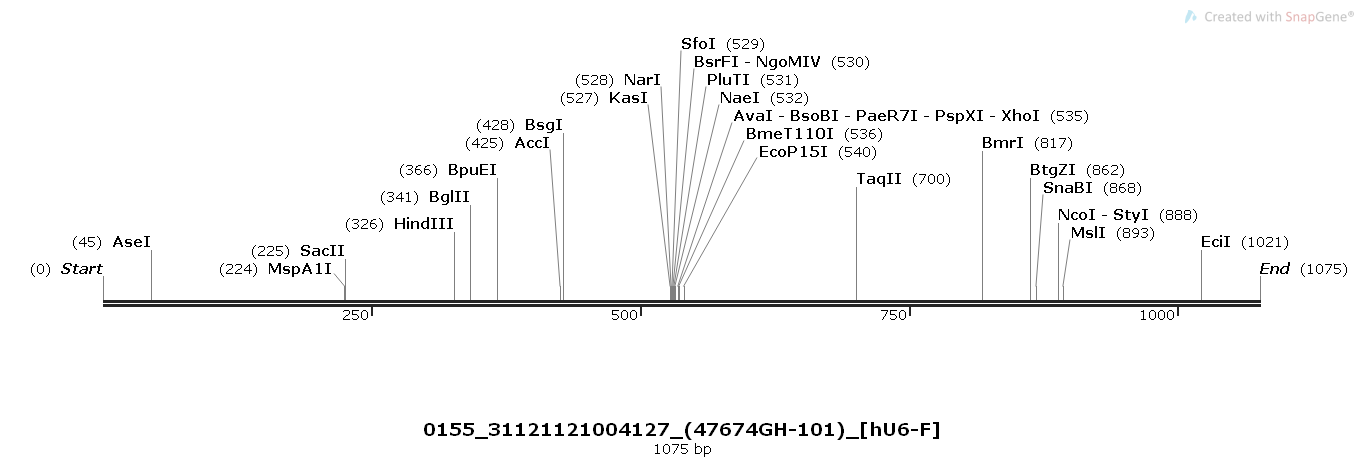


| **测序结果** | **H_NMB sgRNA2-ZsGreen1-T2A-Puro(lenti-SpCas9)** |
| --- | --- |
| ACAATTTGATTACGATACAGGCTGTTAGAGAGATAATTAGAATTAATTTGACTGTAAACACAAAGATATTAGTACAAAATACGTGACGTAGAAAGTAATAATTTCTTGGGTAGTTTGCAGTTTTAAAATTATGTTTTAAAATGGACTATCATATGCTTACCGTAACTTGAAAGTATTTCGATTTCTTGGCTTTATATATCTTGTGGAAAGGACGAAACACCGTCCGGGAGATCCCAGCTGAGGTTTTAGAGCTAGAAATAGCAAGTTAAAATAAGGCTAGTCCGTTATCAACTTGAAAAAGTGGCACCGAGTCGGTGCTTTTTTAAGCTTGGCGTAACTAGATCTTGAGACAAATGGCAGTATTCATCCACAATTTTAAAAGAAAAGGGGGGATTGGGGGGTACAGTGCAGGGGAAAGAATAGTAGACATAATAGCAACAGACATACAAACTAAAGAATTACAAAAACAAATTACAAAAATTCAAAATTTTCGGGTTTATTACAGGGACAGCAGAGATCCACTTTGGCGCCGGCTCGAGGTAATCAATTACGGGGTCATTAGTTCATAGCCCATATATGGAGTTCCGCGTTACATAACTTACGGTAAATGGCCCGCCTGGCTGACCGCCCAACGACCCCCGCCCATTGACGTCAATAATGACGTATGTTCCCATAGTAACGCCAATAGGGACTTTCCATTGACGTCAATGGGTGGAGTATTTACGGTAAACTGCCCACTTGGCAGTACATCAAGTGTATCATATGCCAAGTACGCCCCCTATTGACGTCAATGACGGTAAATGGCCCGCCTGGCATTATGCCCAGTACATGACCTTATGGGACTTTCCTACTTGGCAGTACATCTACGTATTAGTCATCGCTATTACCATGGTGATGCGGTTTTGGCAGTACATCAATGGGCGTGGATAGCGGTTTGACTCACGGGGATTTCCAAGTCTCCACCCCCATTGACGTCAATGGGAGTTTGTTTTGGCACCAAAATCAACGGGACTTTCCAAATGTCGTAACAACTCGCCCCATTGACGCAAATGGGCGGTAGGCGTGTACGGTGGGGAGG | |
| 图例 | 引物  靶位点 |
| 测序引物 | HU6_F：GAGGGCCTATTTCCCATGATT |
| 测序结果 | 0157_31121121004129_(47675GI-101)_[hU6-F] |


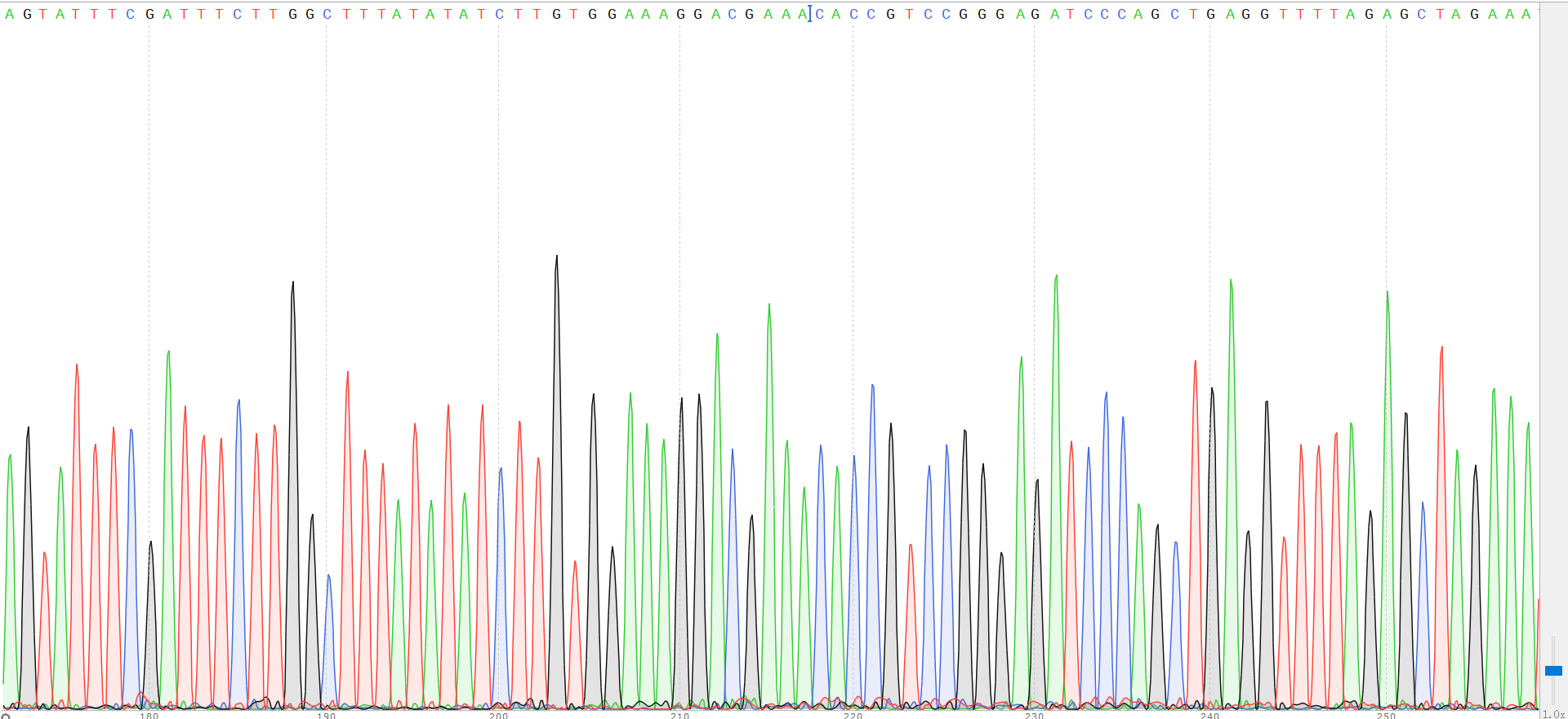


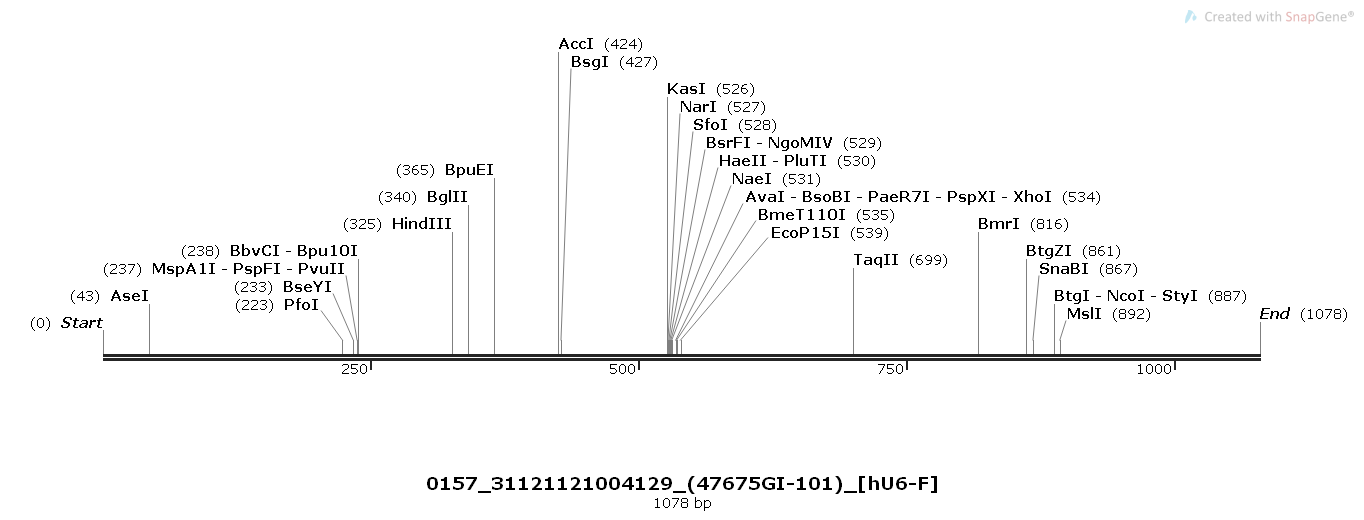


| **测序结果** | **H_NMB sgRNA3-ZsGreen1-T2A-Puro(lenti-SpCas9)** |
| --- | --- |
| ACAAATTGCATTTACGATACAGGCTGTTAGAGAGATAATTAGAATTAATTTGACTGTAAACACAAAGATATTAGTACAAAATACGTGACGTAGAAAGTAATAATTTCTTGGGTAGTTTGCAGTTTTAAAATTATGTTTTAAAATGGACTATCATATGCTTACCGTAACTTGAAAGTATTTCGATTTCTTGGCTTTATATATCTTGTGGAAAGGACGAAACACCGCACTCGCGAGGCAACCTCTGTTTTAGAGCTAGAAATAGCAAGTTAAAATAAGGCTAGTCCGTTATCAACTTGAAAAAGTGGCACCGAGTCGGTGCTTTTTTAAGCTTGGCGTAACTAGATCTTGAGACAAATGGCAGTATTCATCCACAATTTTAAAAGAAAAGGGGGGATTGGGGGGTACAGTGCAGGGGAAAGAATAGTAGACATAATAGCAACAGACATACAAACTAAAGAATTACAAAAACAAATTACAAAAATTCAAAATTTTCGGGTTTATTACAGGGACAGCAGAGATCCACTTTGGCGCCGGCTCGAGGTAATCAATTACGGGGTCATTAGTTCATAGCCCATATATGGAGTTCCGCGTTACATAACTTACGGTAAATGGCCCGCCTGGCTGACCGCCCAACGACCCCCGCCCATTGACGTCAATAATGACGTATGTTCCCATAGTAACGCCAATAGGGACTTTCCATTGACGTCAATGGGTGGAGTATTTACGGTAAACTGCCCACTTGGCAGTACATCAAGTGTATCATATGCCAAGTACGCCCCCTATTGACGTCAATGACGGTAAATGGCCCGCCTGGCATTATGCCCAGTACATGACCTTATGGGACTTTCCTACTTGGCAGTACATCTACGTATTAGTCATCGCTATTACCATGGTGATGCGGTTTTGGCAGTACATCAATGGGCGTGGATAGCGGTTTGACTCACGGGGATTTCCAAGTCTCCACCCCATTGACGTCAATGGAGTTTGTTTGGCACCAAAATCAACGGGACTTTCCCAAATGTCGTAACAACTCCCGCCCCATTGACGCAAATGGGCGGTAGGCGTGTACGGTGGGAGGTCTTTTATAG | |
| 图例 | 引物  靶位点 |
| 测序引物 | HU6_F：GAGGGCCTATTTCCCATGATT |
| 测序结果 | 0159_31121121004131_(47676GJ-101)_[hU6-F] |


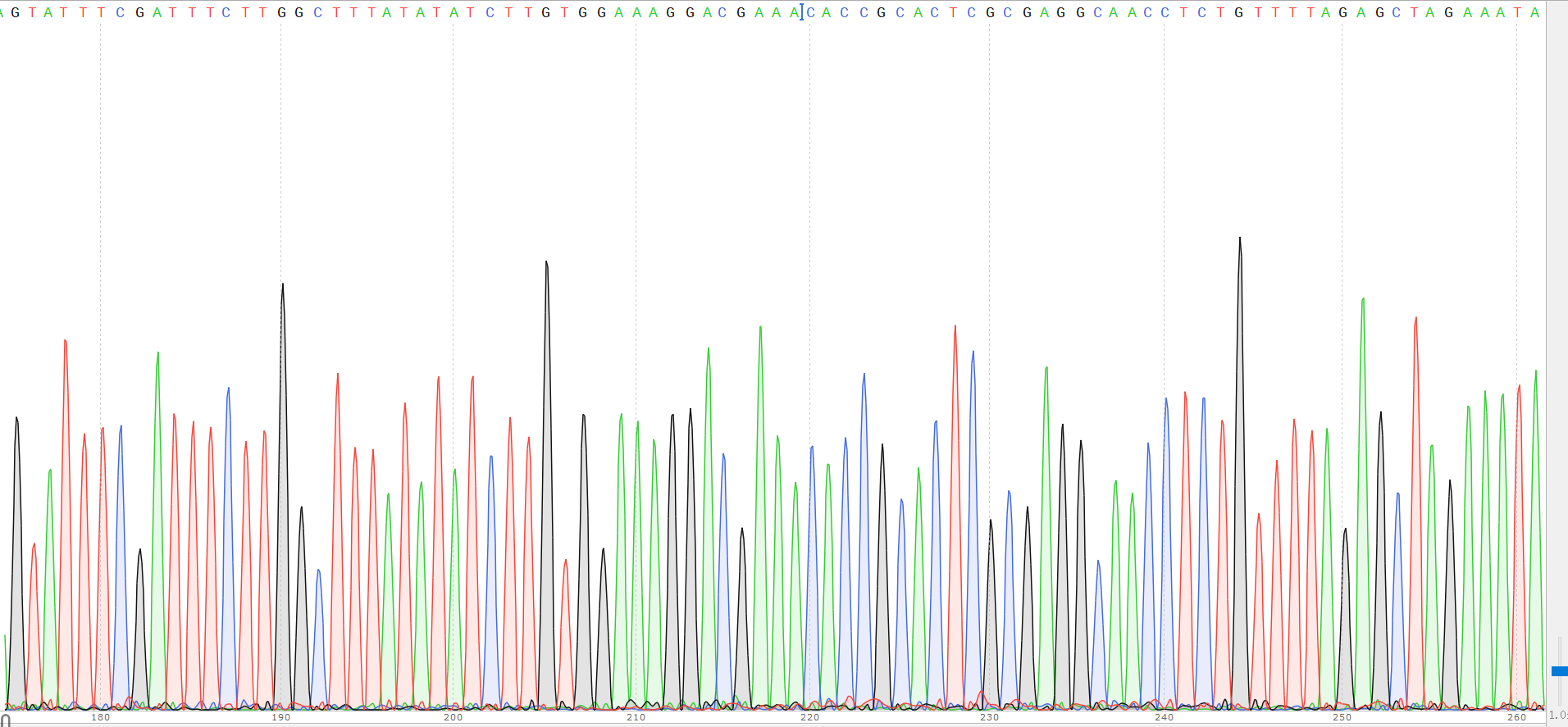


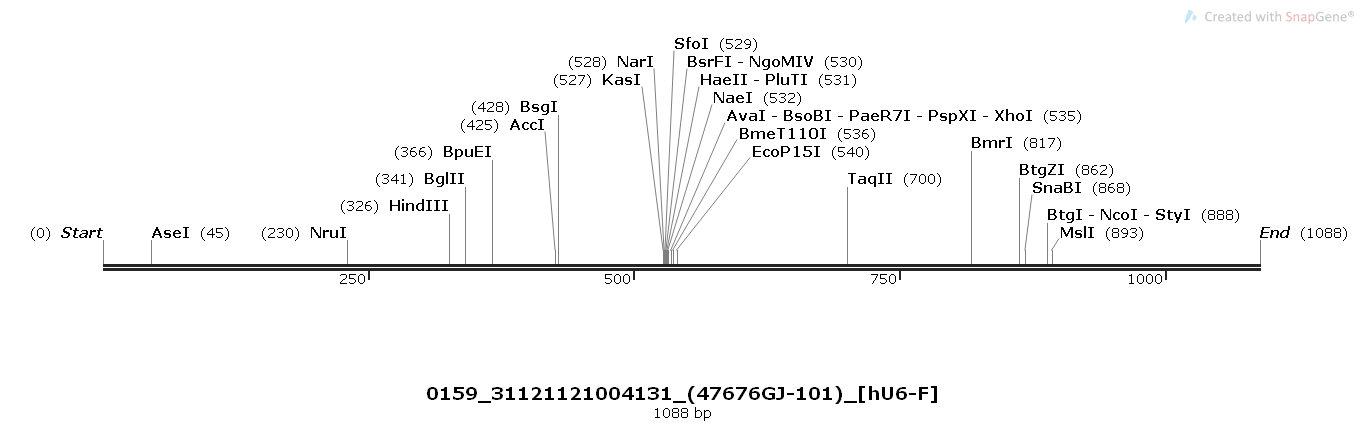


HeLa - NMB


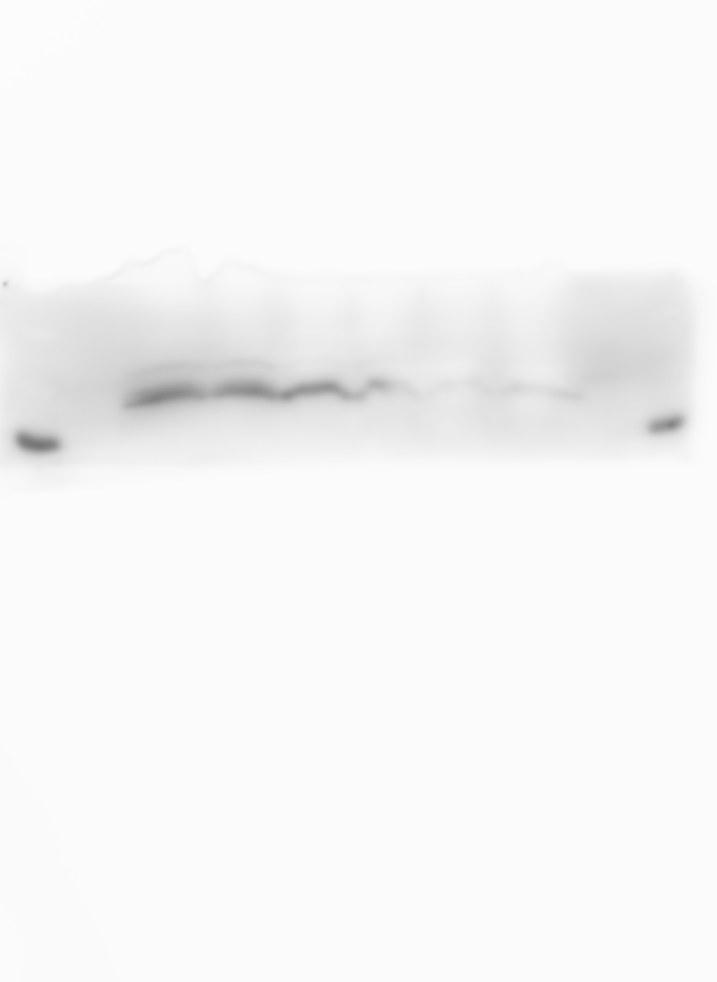


HeLa - GAPDH


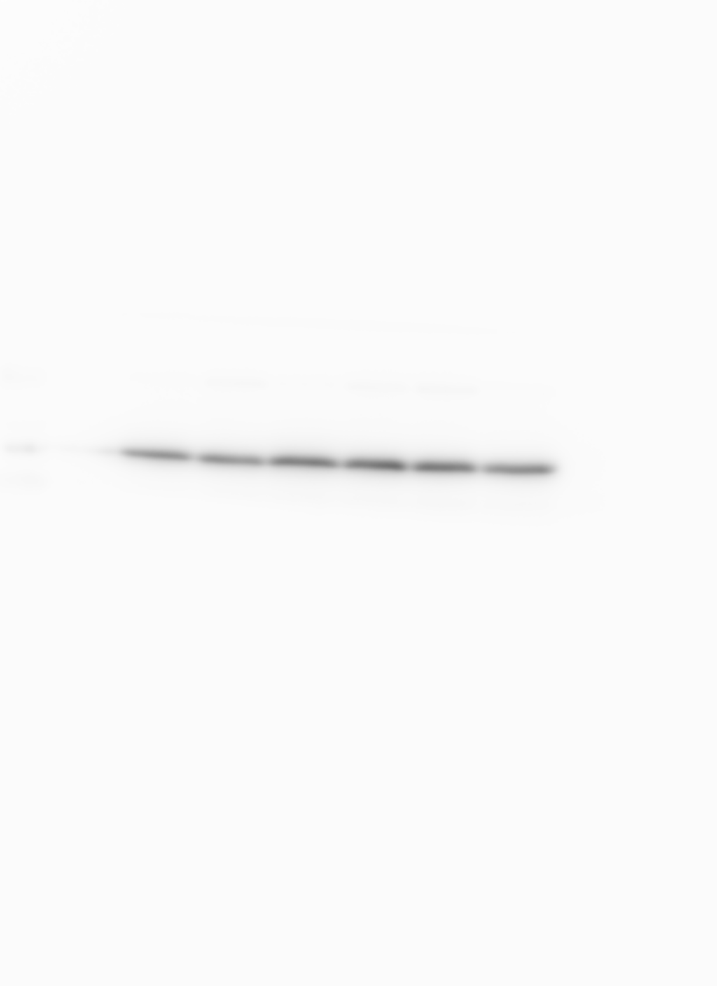


| HeLa Ctrl | HeLa sgNC | HeLa sgNMB #1 | HeLa sgNMB #2 | HeLa sgNMB #3 |
| --- | --- | --- | --- | --- |
| 1 | 0.896124755 | 0.325026634 | 0.139791629 | 0.029301237 |
| 1.263252108 | 1.355527845 | 0.938428009 | 0.296369449 | 0.302341198 |
| 1.435664472 | 0.843980506 | 0.438160519 | 0.552906691 | 0.706303622 |

Figure S3b

ME180 - NMB


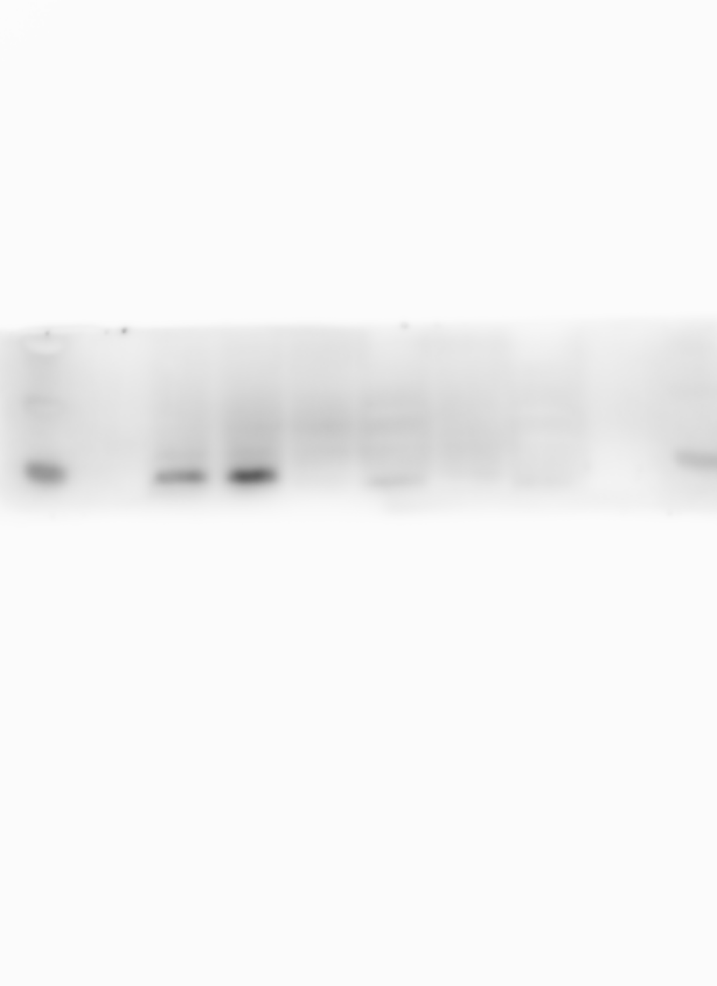


ME180 - GAPDH


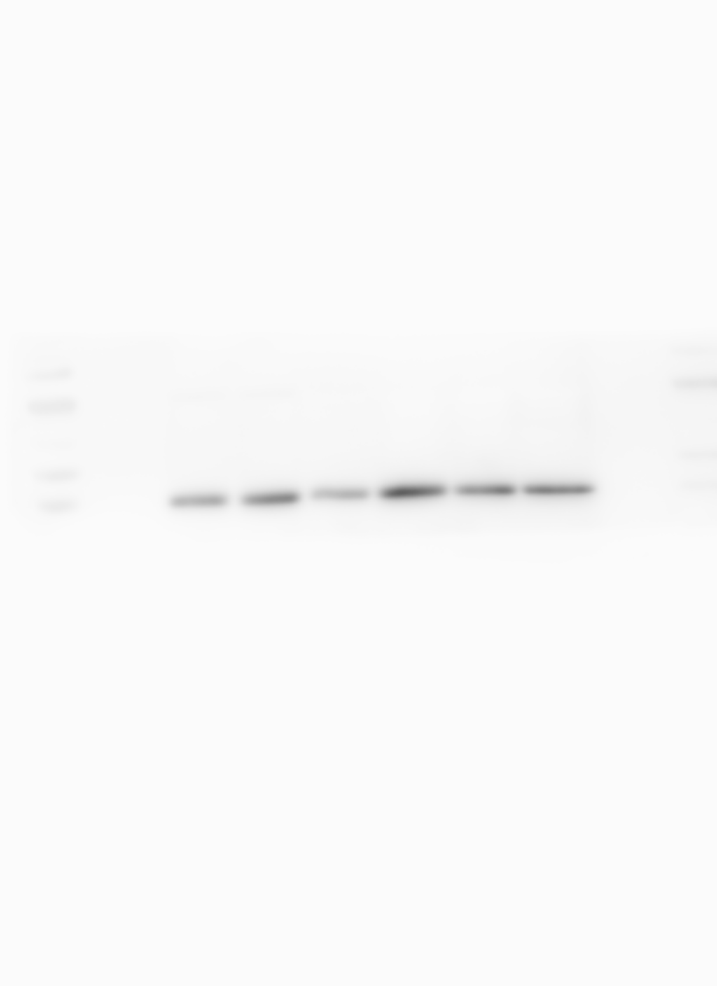


| ME180 Ctrl | ME180 sgNC | ME180 sgNMB #1 | ME180 sgNMB #2 | ME180 sgNMB #3 |
| --- | --- | --- | --- | --- |
| 2.040137189 | 1.429663035 | 0.445351142 | 0.468960965 | 0.034115826 |
| 1 | 1.66923978 | 0.168362235 | 0.288858083 | 0.737572699 |
| 1.226380903 | 1.162485656 | 0.452800431 | 0.425929338 | 0.313978494 |

Figure S3c

NMB concentration in the cell supernatant (ng/L)

| HeLa | HeLa-sgNMB | ME180 | ME180-sgNMB |
| --- | --- | --- | --- |
| 40.14993 | 5.330966 | 39.955721 | 3.864868 |
| 37.681306 | 5.450886 | 40.52478 | 9.67691 |
| 44.391889 | 11.355603 | 43.60153 | 6.410875 |

Figure S3d

NMB concentration in the cell supernatant (ng/L)

| RSC96 Ctrl | RSC96+HeLa | RSC96+HeLa-sgNMB | RSC96+ME180 | RSC96+ME180-sgNMB |
| --- | --- | --- | --- | --- |
| 37.434358 | 69.72159 | 41.271113 | 61.560895 | 42.519904 |
| 28.990233 | 68.39408 | 49.045708 | 63.117865 | 48.007248 |
| 37.605275 | 66.519318 | 35.566069 | 82.128173 | 33.356034 |

Figure S3e

NMB concentration in the cell supernatant (ng/L)

| DRG Ctrl | DRG +HeLa | DRG +HeLa-sgNMB | DRG +ME180 | DRG +ME180-sgNMB |
| --- | --- | --- | --- | --- |
| 35.905611 | 61.114004 | 49.782339 | 56.157388 | 43.999445 |
| 33.210429 | 60.495409 | 49.581294 | 58.545599 | 43.158589 |
| 38.380245 | 62.703932 | 47.178701 | 53.718807 | 44.06588 |

Figure S3f

PBS


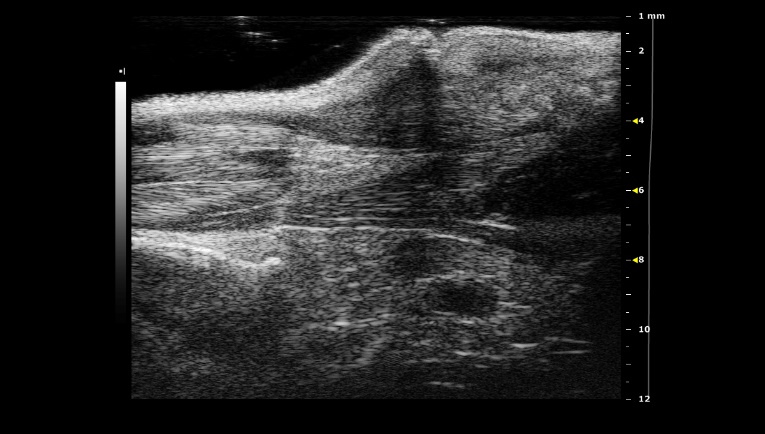


HeLa sgNC Ctrl


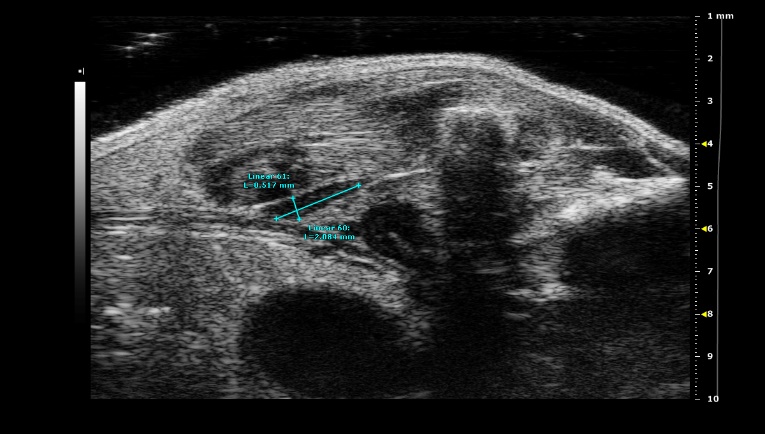


HeLa sgNC +NMB


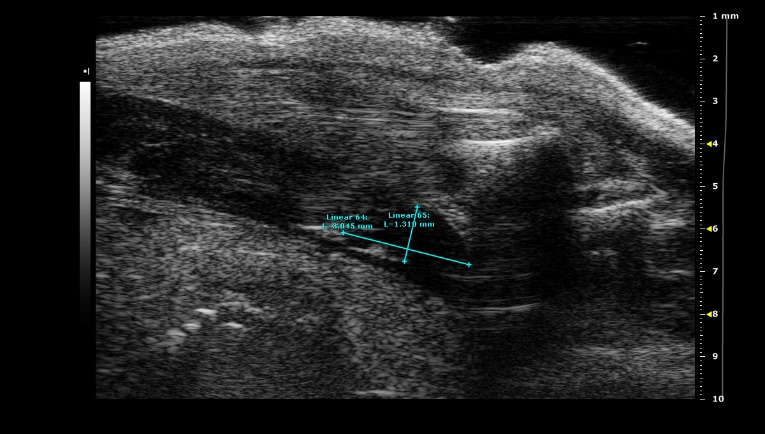


HeLa sgNMB Ctrl


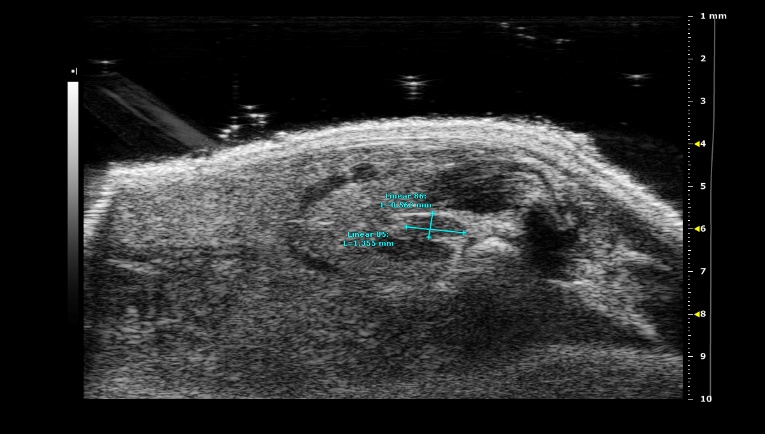


HeLa sgNMB +NMB


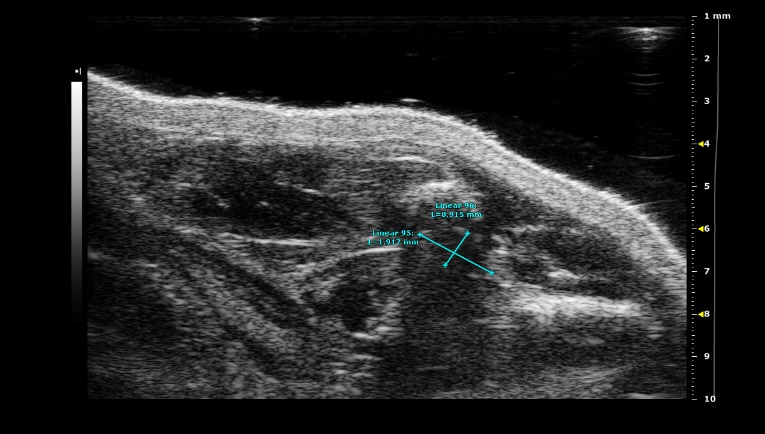


ME180 sgNC Ctrl


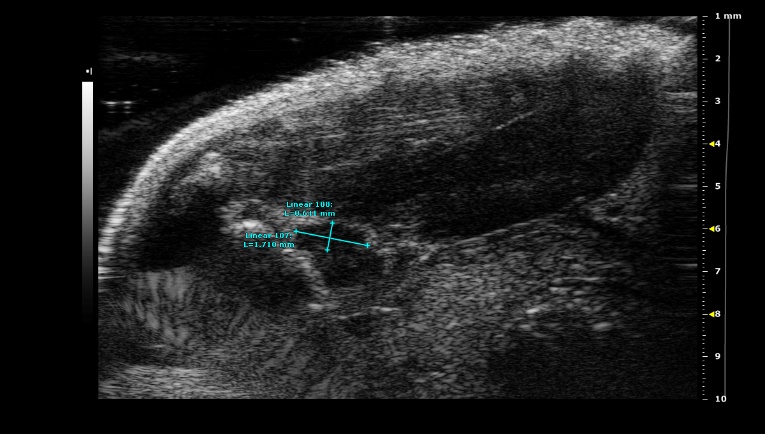


ME180 sgNC +NMB


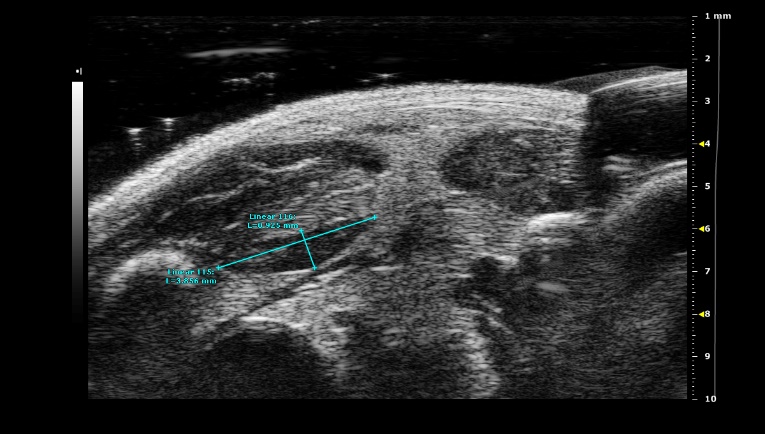


ME180 sgNMB Ctrl

ME180 sgNMB +NMB

Figure S3g

PBS

HeLa sgNC Ctrl

HeLa sgNC +NMB

HeLa sgNMB Ctrl

HeLa sgNMB +NMB

ME180 sgNC Ctrl

ME180 sgNC +NMB

ME180 sgNMB Ctrl

ME180 sgNMB +NMB

Figure S3h

Figure S3i,j

PBS

HeLa sgNC Ctrl

HeLa sgNC +NMB

HeLa sgNMB Ctrl

HeLa sgNMB +NMB

ME180 sgNC Ctrl

ME180 sgNC +NMB

ME180 sgNMB Ctrl

ME180 sgNMB +NMB

Figure S3k

PBS

HeLa sgNC Ctrl

HeLa sgNC +NMB

HeLa sgNMB Ctrl

HeLa sgNMB +NMB

ME180 sgNC Ctrl

ME180 sgNC +NMB

ME180 sgNMB Ctrl

ME180 sgNMB +NMB

Figure S4a

PBS

HeLa sgNC Ctrl

HeLa sgNC +NMB

HeLa sgNMB Ctrl

HeLa sgNMB +NMB

ME180 sgNC Ctrl

ME180 sgNC +NMB

ME180 sgNMB Ctrl

ME180 sgNMB +NMB

Figure S4b

PBS

HeLa sgNC Ctrl

HeLa sgNC +NMB

HeLa sgNMB Ctrl

HeLa sgNMB +NMB

ME180 sgNC Ctrl

ME180 sgNC +NMB

ME180 sgNMB Ctrl

ME180 sgNMB +NMB

Figure S4c

PBS

HeLa sgNC Ctrl

HeLa sgNC +NMB

HeLa sgNMB Ctrl

HeLa sgNMB +NMB

ME180 sgNC Ctrl

ME180 sgNC +NMB

ME180 sgNMB Ctrl

ME180 sgNMB +NMB

Figure S4d

Figure S4g

| Weight of tumor (g) | HeLa sgNC (n=5) | HeLa sgNC+ NMB (n=5) | HeLa sgNMB (n=5) | HeLa sgNMB+ NMB (n=5) | ME180 sgNC (n=5) | ME180 sgNC+ NMB (n=5) | ME180 sgNMB (n=5) | ME180 sgNMB+ NMB (n=5) |
| --- | --- | --- | --- | --- | --- | --- | --- | --- |
| One week | 0.05666 | 0.01309 | 0.0051 | 0.02082 | 0.01671 | 0.01456 | 0.01205 | 0.01361 |
|  | 0.0186 | 0.02041 | 0.00545 | 0.01003 | 0.01026 | 0.01285 | 0.02202 | 0.00974 |
|  | 0.00749 | 0.01071 | 0.01109 | 0.01141 | 0.01122 | 0.01723 | 0.01238 | 0.01342 |
|  | 0.00707 | 0.02259 | 0.01097 | 0.01756 | 0.0122 | 0.00693 | 0.01077 | 0.00952 |
|  | 0.02222 | 0.01403 | 0.01737 | 0.02256 | 0.01844 | 0.01323 | 0.00905 | 0.02283 |

Figure S5a

PBS

HeLa sgNC Ctrl

HeLa sgNC +NMB

HeLa sgNMB Ctrl

HeLa sgNMB +NMB

ME180 sgNC Ctrl

ME180 sgNC +NMB

ME180 sgNMB Ctrl

ME180 sgNMB +NMB

Figure S5b

PBS

HeLa sgNC Ctrl

HeLa sgNC +NMB

HeLa sgNMB Ctrl

HeLa sgNMB +NMB

ME180 sgNC Ctrl

ME180 sgNC +NMB

ME180 sgNMB Ctrl

ME180 sgNMB +NMB

Figure S5c

PBS

HeLa sgNC Ctrl

HeLa sgNC +NMB

HeLa sgNMB Ctrl

HeLa sgNMB +NMB

ME180 sgNC Ctrl

ME180 sgNC +NMB

ME180 sgNMB Ctrl

ME180 sgNMB +NMB

Figure S5d

Figure S5g

| Weight of tumor (g) | HeLa sgNC (n=5) | HeLa sgNC+ NMB (n=5) | HeLa sgNMB (n=5) | HeLa sgNMB+ NMB (n=5) | ME180 sgNC (n=5) | ME180 sgNC+ NMB (n=5) | ME180 sgNMB (n=5) | ME180 sgNMB+ NMB (n=5) |
| --- | --- | --- | --- | --- | --- | --- | --- | --- |
| two weeks | 0.09404 | 0.00605 | 0.05652 | 0.27204 | 0.20541 | 0.03294 | 0.01048 | 0.07694 |
|  | 0.06292 | 0.31905 | 0.00822 | 0.08907 | 0.11006 | 0.17124 | 0.00515 | 0.04326 |
|  | 0.05613 | 0.33357 | 0.31006 | 0.17724 | 0.006412 | 0.64678 | 0.0101 | 0.02718 |
|  | 0.03066 | 0.02576 | 0.25487 | 0.00402 | 0.03439 | 0.1255 | 0.03298 | 0.09006 |
|  | 0.05212 | 0.48274 | 0.04675 | 0.01468 | 0.16392 | 0.14194 | 0.00482 | 0.11067 |

Figure S6a

PBS

HeLa sgNC Ctrl

HeLa sgNC +NMB

HeLa sgNMB Ctrl

HeLa sgNMB +NMB

ME180 sgNC Ctrl

ME180 sgNC +NMB

ME180 sgNMB Ctrl

ME180 sgNMB +NMB

Figure S6b

PBS

HeLa sgNC Ctrl

HeLa sgNC +NMB

HeLa sgNMB Ctrl

HeLa sgNMB +NMB

ME180 sgNC Ctrl

ME180 sgNC +NMB

ME180 sgNMB Ctrl

ME180 sgNMB +NMB

Figure S6c

PBS

HeLa sgNC Ctrl

HeLa sgNC +NMB

HeLa sgNMB Ctrl

HeLa sgNMB +NMB

ME180 sgNC Ctrl

ME180 sgNC +NMB

ME180 sgNMB Ctrl

ME180 sgNMB +NMB

Figure S6d

Figure S6g

| Weight of tumor (g) | HeLa sgNC (n=5) | HeLa sgNC+ NMB (n=5) | HeLa sgNMB (n=5) | HeLa sgNMB+ NMB (n=5) | ME180 sgNC (n=5) | ME180 sgNC+ NMB (n=5) | ME180 sgNMB (n=5) | ME180 sgNMB+ NMB (n=5) |
| --- | --- | --- | --- | --- | --- | --- | --- | --- |
| Five weeks | 1.50604 | 0.99375 | 0.15403 | 0.84968 | 0.80903 | 2.56351 | 0.09125 | 1.03128 |
|  | 1.07766 | 1.36324 | 0.13 | 0.71275 | 1.15114 | 1.26285 | 0.17 | 0.42537 |
|  | 0.77 | 2.12955 | 0.14 | 1.29705 | 1.10811 | 1.50725 | 0.21 | 2.01532 |
|  | 1.03 | 1.56328 | 0.13 | 0.99561 | 0.97 | 2.25872 | 0.32 | 0.45239 |
|  | 0.81 | 1.89365 | 0.12 | 0.53578 | 1.101735 | 2.02303 | 0.26 | 1.02493 |

Figure 3a

RSC96 Ctrl

RSC96 RSC96-CM

RSC96 HeLa-CM

RSC96 ME180-CM

Figure 3b

RSC96 Ctrl

RSC96 cocultured with HeLa

RSC96 cocultured with ME180

Figure 3c

|  | *18s* Ct value | *cJUN* Ct value | 2^-∆∆Ct^ |
| --- | --- | --- | --- |
| RSC96 Ctrl | 8.329032898 | 19.25847308 | 0.941118123 |
| RSC96 Ctrl | 8.430188179 | 19.48362414 | 0.863610049 |
| RSC96 Ctrl | 9.070634842 | 19.61342239 | 1.23037692 |
| RSC96-HeLa | 9.062072754 | 18.8990593 | 2.006806278 |
| RSC96-HeLa | 9.023713748 | 18.8990593 | 1.954151541 |
| RSC96-HeLa | 8.977903366 | 18.77136993 | 2.068265408 |
| RSC96-ME180 | 10.24610424 | 19.59244156 | 2.819717401 |
| RSC96-ME180 | 10.22454166 | 20.25128047 | 1.759479976 |
| RSC96-ME180 | 9.629910469 | 19.50498486 | 1.954518867 |

|  | *18s* Ct value | *BDNF* Ct value | 2^-∆∆Ct^ |
| --- | --- | --- | --- |
| RSC96 Ctrl | 8.329032898 | 20.6789093 | 1.081785486 |
| RSC96 Ctrl | 8.430188179 | 21.14685631 | 0.83893022 |
| RSC96 Ctrl | 9.070634842 | 21.39396286 | 1.101876728 |
| RSC96-HeLa | 9.062072754 | 20.80456352 | 1.648095773 |
| RSC96-HeLa | 9.023713748 | 20.89196587 | 1.510513138 |
| RSC96-HeLa | 8.977903366 | 20.76070595 | 1.602682078 |
| RSC96-ME180 | 10.24610424 | 21.20444298 | 2.838152504 |
| RSC96-ME180 | 10.22454166 | 21.18872452 | 2.826678878 |
| RSC96-ME180 | 9.629910469 | 20.99171829 | 2.14575153 |

Figure 3d

RSC96 Ctrl

RSC96 cocultured with HeLa

RSC96 cocultured with ME180

Figure 3e

Migration

RSC96 Ctrl

RSC96 cocultured with HeLa

RSC96 cocultured with ME180

Invasion

RSC96 Ctrl

RSC96 cocultured with HeLa

RSC96 cocultured with ME180

Figure 3f

NMB 0 μM

NMB 0.01 μM

NMB 0.25 μM

NMB 1 μM

Figure 3g

RSC96 +NMB 0 μM

RSC96 +NMB 0.01 μM

RSC96 +NMB 0.25 μM

RSC96 +NMB 1 μM

Figure 3h

|  | *18s* Ct value | *cJUN* Ct value | 2^-∆∆Ct^ |
| --- | --- | --- | --- |
| RSC96 Ctrl | 19.6038812 | 7.617802461 | 1 |
| RSC96 Ctrl | 19.72621473 | 8.049489975 | 1.239152706 |
| RSC96 Ctrl | 20.412 | 8.506700516 | 1.057234962 |
| RSC96+0.01μM NMB | 20.72765923 | 9.623436292 | 1.678987877 |
| RSC96+0.01μM NMB | 19.60415395 | 8.256453832 | 1.556578807 |
| RSC96+0.01μM NMB | 21.276 | 9.851142883 | 1.475113357 |
| RSC96+0.25μM NMB | 19.72014364 | 8.822463671 | 2.126379019 |
| RSC96+0.25μM NMB | 19.59820557 | 8.709063848 | 2.139000798 |
| RSC96+0.25μM NMB | 20.015 | 19.968 | 3.106109028 |
| RSC96+1μM NMB | 19.49953906 | 9.26765728 | 3.373384956 |
| RSC96+1μM NMB | 19.08042272 | 8.96778965 | 3.664066455 |
| RSC96+1μM NMB | 20.052 | 20.121 | 4.039959632 |

|  | *18s* Ct value | *BDNF* Ct value | 2^-∆∆Ct^ |
| --- | --- | --- | --- |
| RSC96 Ctrl | 21.81139946 | 7.617802461 | 1 |
| RSC96 Ctrl | 22.57113647 | 8.049489975 | 0.796612763 |
| RSC96 Ctrl | 22.921 | 8.506700516 | 0.858438252 |
| RSC96+0.01μM NMB | 22.10934766 | 8.822463671 | 1.874769213 |
| RSC96+0.01μM NMB | 21.95697149 | 8.256453832 | 1.407445779 |
| RSC96+0.01μM NMB | 23.091 | 9.851142883 | 1.936306466 |
| RSC96+0.25μM NMB | 22.18430328 | 9.26765728 | 2.423263007 |
| RSC96+0.25μM NMB | 21.86742528 | 8.709063848 | 2.049448254 |
| RSC96+0.25μM NMB | 22.882 | 10.22934055 | 2.908898875 |
| RSC96+1μM NMB | 22.45776113 | 9.623436292 | 2.565557148 |
| RSC96+1μM NMB | 21.54753113 | 8.96778965 | 3.060687011 |
| RSC96+1μM NMB | 21.778 | 9.504295349 | 3.783203221 |

Figure 3i

Migration

RSC96+0μM NMB

RSC96+0.01μM NMB

RSC96+0.25μM NMB

RSC96+1μM NMB

Invasion

RSC96+0μM NMB

RSC96+0.01μM NMB

RSC96+0.25μM NMB

RSC96+1μM NMB

Figure 3j

RSC96 +HeLa sgNC

RSC96 +HeLa sgNMB

RSC96 +ME180 sgNC

RSC96 +ME180 sgNMB

Figure 3k

RSC96 +HeLa sgNC

RSC96 +HeLa sgNMB

RSC96 +ME180 sgNC

RSC96 +ME180 sgNMB

Figure 3l

|  | *18s* Ct value | *cJUN* Ct value | 2^-∆∆Ct^ |
| --- | --- | --- | --- |
| RSC96 Ctrl | 21.8250351 | 8.491376559 | 1 |
| RSC96 Ctrl | 21.93383853 | 8.956150055 | 1.279845857 |
| RSC96 Ctrl | 20.85614522 | 7.914620717 | 1.312333169 |
| RSC96+HeLa | 18.74512863 | 8.338922501 | 7.607658072 |
| RSC96+ HeLa | 18.78309441 | 9.114169439 | 12.68220387 |
| RSC96+ HeLa | 18.52231312 | 7.879453182 | 6.45670718 |
| RSC96+HeLa sgNMB | 20.47287305 | 8.147723516 | 2.011830834 |
| RSC96+HeLa sgNMB | 20.13098399 | 9.09617424 | 4.920649608 |
| RSC96+HeLa sgNMB | 19.47052574 | 7.683895588 | 2.922146245 |
| RSC96+ME180 | 18.20589161 | 7.789795081 | 7.555682128 |
| RSC96+ME180 | 19.82909775 | 9.587158839 | 8.525116961 |
| RSC96+ME180 | 18.07535744 | 7.644901911 | 7.480854185 |
| RSC96+ME180 sgNMB | 19.54714489 | 7.851986567 | 3.113420009 |
| RSC96+ME180 sgNMB | 21.51470757 | 9.821755091 | 3.118184 |
| RSC96+ME180 sgNMB | 19.238451 | 7.606717428 | 3.253347569 |

|  | *18s* Ct value | *BDNF* Ct value | 2^-∆∆Ct^ |
| --- | --- | --- | --- |
| RSC96 Ctrl | 25.57941532 | 8.491376559 | 1 |
| RSC96 Ctrl | 25.53746351 | 8.956150055 | 1.420821493 |
| RSC96 Ctrl | 24.79779816 | 7.914620717 | 1.152575552 |
| RSC96+HeLa | 21.23570315 | 8.338922501 | 18.26814338 |
| RSC96+ HeLa | 22.5445563 | 9.114169439 | 12.62010406 |
| RSC96+ HeLa | 21.30872218 | 7.879453182 | 12.62988648 |
| RSC96+HeLa sgNMB | 22.38098272 | 8.147723516 | 7.233929617 |
| RSC96+HeLa sgNMB | 23.54509989 | 9.09617424 | 6.229485905 |
| RSC96+HeLa sgNMB | 22.42524433 | 7.683895588 | 5.086558999 |
| RSC96+ME180 | 20.80177752 | 7.851986567 | 17.60908215 |
| RSC96+ME180 | 22.94566091 | 9.821755091 | 15.60712552 |
| RSC96+ME180 | 21.01837667 | 7.606717428 | 12.7849935 |
| RSC96+ME180 sgNMB | 21.85860189 | 7.789795081 | 8.10735859 |
| RSC96+ME180 sgNMB | 23.53180059 | 9.587158839 | 8.836022016 |
| RSC96+ME180 sgNMB | 21.97948329 | 7.644901911 | 6.743312175 |

Figure 3m

Migration

RSC96+HeLa sgNC

RSC96+HeLa sgNMB

RSC96+ME180 sgNC

RSC96+ME180 sgNMB

Invasion

RSC96+HeLa sgNC

RSC96+HeLa sgNMB

RSC96+ME180 sgNC

RSC96+ME180 sgNMB

Figure S7a

|  | High (%) | Medium (%) | Low (%) |
| --- | --- | --- | --- |
| RSC96 Ctrl | 91.67 | 8.33 | 0 |
| RSC96 Ctrl | 86.21 | 10.34 | 3.45 |
| RSC96 Ctrl | 81.82 | 13.64 | 4.55 |
|  |  |  |  |
| RSC96-HeLa | 76.92 | 19.23 | 3.85 |
| RSC96-HeLa | 75 | 16.67 | 8.33 |
| RSC96-HeLa | 73.53 | 20.59 | 5.88 |
|  |  |  |  |
| RSC96-ME180 | 72.41 | 3.45 | 24.14 |
| RSC96-ME180 | 62.5 | 12.5 | 25 |
| RSC96-ME180 | 63.16 | 10.53 | 26.32 |

Figure S7b

|  | Filopodia (%) | Lamellipodia (%) |
| --- | --- | --- |
| RSC96 Ctrl | 10 | 90 |
| RSC96 Ctrl | 9.09 | 90.91 |
| RSC96 Ctrl | 5.56 | 94.44 |
|  |  |  |
| RSC96-RSC96 | 4.167 | 95.83 |
| RSC96-RSC96 | 5.556 | 94.44 |
| RSC96-RSC96 | 3.846 | 96.15 |
|  |  |  |
| RSC96-HeLa | 28 | 72 |
| RSC96-HeLa | 40.909 | 59.09 |
| RSC96-HeLa | 42.857 | 57.14 |
|  |  |  |
| RSC96-ME180 | 37.5 | 62.5 |
| RSC96-ME180 | 37.5 | 62.5 |
| RSC96-ME180 | 33.333 | 66.67 |

Figure S7c

|  | Nestin MFI (a.u.) |
| --- | --- |
| RSC96 Ctrl | 83.183 |
| RSC96 Ctrl | 79.80133333 |
| RSC96 Ctrl | 76.97533333 |
|  |  |
| RSC96-HeLa | 126.8493333 |
| RSC96-HeLa | 130.067 |
| RSC96-HeLa | 140.3603333 |
|  |  |
| RSC96-ME180 | 127.5406667 |
| RSC96-ME180 | 123.3946667 |
| RSC96-ME180 | 120.87 |

Figure S7d

|  | *18s* Ct value | *NES* Ct value | 2^-∆∆Ct^ |
| --- | --- | --- | --- |
| RSC96 Ctrl | 10.23585415 | 33.474 | 1.017790133 |
| RSC96 Ctrl | 8.335653146 | 31.440 | 1.116288472 |
| RSC96 Ctrl | 8.033972263 | 31.482 | 0.880167492 |
|  |  |  |  |
| RSC96-HeLa | 8.831083934 | 30.354 | 3.340914014 |
| RSC96-HeLa | 8.066020807 | 29.901 | 2.692326554 |
| RSC96-HeLa | 9.023713748 | 30.755 | 2.891430281 |
|  |  |  |  |
| RSC96-ME180 | 8.920184453 | 30.401 | 3.439390735 |
| RSC96-ME180 | 7.974963347 | 29.508 | 3.319206052 |
| RSC96-ME180 | 10.03351879 | 30.959 | 5.057246491 |

Figure S7e

|  | *18s* Ct value | *GAP43* Ct value | 2^-∆∆Ct^ |
| --- | --- | --- | --- |
| RSC96 Ctrl | 8.235854149 | 29.497 | 1.021954869 |
| RSC96 Ctrl | 8.335653146 | 29.896 | 0.830609097 |
| RSC96 Ctrl | 8.033972263 | 29.090 | 1.178071364 |
|  |  |  |  |
| RSC96-HeLa | 10.83108393 | 30.504 | 3.072508451 |
| RSC96-HeLa | 7.066020807 | 26.707 | 3.14075877 |
| RSC96-HeLa | 9.023713748 | 29.325 | 1.988397922 |
|  |  |  |  |
| RSC96-ME180 | 8.920184453 | 28.044 | 4.495066248 |
| RSC96-ME180 | 6.974963347 | 25.824 | 5.437331158 |
| RSC96-ME180 | 10.03351879 | 29.211 | 4.331911267 |

|  | *18s* Ct value | *GDNF* Ct value | 2^-∆∆Ct^ |
| --- | --- | --- | --- |
| RSC96 Ctrl | 9.070634842 | 22.53344536 | 1.528034051 |
| RSC96 Ctrl | 8.430188179 | 22.37901878 | 1.091004162 |
| RSC96 Ctrl | 7.506136417 | 22.31795692 | 0.599847096 |
|  |  |  |  |
| RSC96-HeLa | 11.46764088 | 24.56889725 | 1.96323223 |
| RSC96-HeLa | 11.09848785 | 24.48137093 | 1.615078331 |
| RSC96-HeLa | 9.92712307 | 24.43479729 | 0.74062389 |
|  |  |  |  |
| RSC96-ME180 | 8.920184453 | 24.2101841 | 0.4306201 |
| RSC96-ME180 | 6.974963347 | 23.04289627 | 0.251138355 |
| RSC96-ME180 | 10.03351879 | 24.37871742 | 0.828910692 |

|  | *18s* Ct value | *NGFR* Ct value | 2^-∆∆Ct^ |
| --- | --- | --- | --- |
| RSC96 Ctrl | 10.23585415 | 31.052 | 0.874314923 |
| RSC96 Ctrl | 8.335653146 | 28.410 | 1.462607117 |
| RSC96 Ctrl | 8.033972263 | 29.011 | 0.78199581 |
|  |  |  |  |
| RSC96-HeLa | 10.83108393 | 30.109 | 2.54058027 |
| RSC96-HeLa | 8.066020807 | 28.088 | 1.516130103 |
| RSC96-HeLa | 10.02371375 | 29.417 | 2.345042978 |
|  |  |  |  |
| RSC96-ME180 | 9.920184453 | 29.521 | 2.030313114 |
| RSC96-ME180 | 7.974963347 | 27.165 | 2.69861791 |
| RSC96-ME180 | 10.03351879 | 29.585 | 2.101767733 |

|  | *18s* Ct value | *MBP* Ct value | 2^-∆∆Ct^ |
| --- | --- | --- | --- |
| RSC96 Ctrl | 8.235854149 | 34.662 | 1.046649667 |
| RSC96 Ctrl | 8.335653146 | 34.946 | 0.921337918 |
| RSC96 Ctrl | 8.033972263 | 34.474 | 1.03700229 |
|  |  |  |  |
| RSC96-HeLa | 9.831083934 | 32.635 | 12.8851283 |
| RSC96-HeLa | 7.066020807 | 30.108 | 10.92759622 |
| RSC96-HeLa | 9.023713748 | 32.204 | 9.930042532 |
|  |  |  |  |
| RSC96-ME180 | 8.920184453 | 33.466 | 3.852758908 |
| RSC96-ME180 | 6.974963347 | 30.152 | 9.952369199 |
| RSC96-ME180 | 9.033518791 | 32.348 | 9.045017422 |

Figure S7f

|  | EdU positive cells (%) |
| --- | --- |
| RSC96 Ctrl | 0.56 |
| RSC96 Ctrl | 0.82 |
| RSC96 Ctrl | 0.58 |
|  |  |
| RSC96-HeLa | 8.66 |
| RSC96-HeLa | 6.23 |
| RSC96-HeLa | 13.64 |
|  |  |
| RSC96-ME180 | 10.42 |
| RSC96-ME180 | 9.63 |
| RSC96-ME180 | 7.47 |

Figure S7g

|  | Number of migration cells/HPF | Number of invasion cells/HPF |
| --- | --- | --- |
| RSC96 Ctrl | 71.425 | 124.4285714 |
| RSC96 Ctrl | 70.7 | 123.2 |
| RSC96 Ctrl | 68 | 122.5 |
|  |  |  |
| RSC96-HeLa | 339 | 574.25 |
| RSC96-HeLa | 282 | 589.9090909 |
| RSC96-HeLa | 317.1428571 | 603.3 |
|  |  |  |
| RSC96-ME180 | 479.2 | 839.6 |
| RSC96-ME180 | 548.1818182 | 848.5 |
| RSC96-ME180 | 625.8 | 826 |

Figure S7h

|  | High (%) | Medium (%) | Low (%) |
| --- | --- | --- | --- |
| RSC96 Ctrl | 83.78 | 8.11 | 8.11 |
| RSC96 Ctrl | 77.14 | 17.14 | 5.71 |
| RSC96 Ctrl | 85 | 10 | 5 |
|  |  |  |  |
| RSC96-0.01μM NMB | 54.55 | 36.36 | 9.09 |
| RSC96-0.01μM NMB | 70.83 | 25 | 4.17 |
| RSC96-0.01μM NMB | 55.56 | 33.33 | 11.11 |
|  |  |  |  |
| RSC96-0.25μM NMB | 57.89 | 26.32 | 15.79 |
| RSC96-0.25μM NMB | 66.67 | 18.52 | 14.81 |
| RSC96-0.25μM NMB | 59.26 | 25.93 | 14.81 |
|  |  |  |  |
| RSC96-1μM NMB | 28.57 | 28.57 | 42.86 |
| RSC96-1μM NMB | 61.9 | 14.29 | 23.81 |
| RSC96-1μM NMB | 60 | 20 | 20 |

Figure S7i

|  | Filopodia (%) | Lamellipodia (%) |
| --- | --- | --- |
| RSC96 Ctrl | 6.25 | 93.75 |
| RSC96 Ctrl | 5.56 | 94.44 |
| RSC96 Ctrl | 6.25 | 93.75 |
|  |  |  |
| RSC96-0.01μM NMB | 21.429 | 78.57 |
| RSC96-0.01μM NMB | 11.111 | 88.89 |
| RSC96-0.01μM NMB | 41.667 | 58.33 |
|  |  |  |
| RSC96-0.25μM NMB | 14.286 | 85.71 |
| RSC96-0.25μM NMB | 23.077 | 76.92 |
| RSC96-0.25μM NMB | 20 | 80 |
|  |  |  |
| RSC96-1μM NMB | 40 | 60 |
| RSC96-1μM NMB | 33.333 | 66.67 |
| RSC96-1μM NMB | 26.667 | 73.33 |

Figure S7j

|  | EdU positive cells (%) |
| --- | --- |
| RSC96 Ctrl | 34.77563286 |
| RSC96 Ctrl | 28.38720151 |
| RSC96 Ctrl | 29.48142467 |
|  |  |
| RSC96-0.01μM NMB | 41.9551954 |
| RSC96-0.01μM NMB | 39.59018779 |
| RSC96-0.01μM NMB | 31.54543892 |
|  |  |
| RSC96-0.25μM NMB | 55.70124019 |
| RSC96-0.25μM NMB | 52.36861254 |
| RSC96-0.25μM NMB | 59.92918011 |
|  |  |
| RSC96-1μM NMB | 62.97289283 |
| RSC96-1μM NMB | 51.21619346 |
| RSC96-1μM NMB | 44.81894305 |

Figure S7k

|  | *18s* Ct value | *GAP43* Ct value | 2^-∆∆Ct^ |
| --- | --- | --- | --- |
| RSC96 Ctrl | 7.617802461 | 30.37137604 | 0.545767665 |
| RSC96 Ctrl | 8.049489975 | 29.92942238 | 1 |
| RSC96 Ctrl | 8.506700516 | 30.302 | 1.06071044 |
|  |  |  |  |
| RSC96-0.01μM NMB | 9.623436292 | 31.26164341 | 1.182405828 |
| RSC96-0.01μM NMB | 8.256453832 | 30.04285145 | 1.066981223 |
| RSC96-0.01μM NMB | 10.14922047 | 31.647 | 1.303342681 |
|  |  |  |  |
| RSC96-0.25μM NMB | 8.822463671 | 30.58856678 | 1.082096599 |
| RSC96-0.25μM NMB | 8.709063848 | 30.30631383 | 1.21645455 |
| RSC96-0.25μM NMB | 9.851142883 | 31.324 | 1.32619186 |
|  |  |  |  |
| RSC96-1μM NMB | 9.26765728 | 30.63849163 | 1.423160183 |
| RSC96-1μM NMB | 8.96778965 | 30.41614405 | 1.348707969 |
| RSC96-1μM NMB | 10.22934055 | 30.616 | 2.814638198 |

|  | *18s* Ct value | *GDNF* Ct value | 2^-∆∆Ct^ |
| --- | --- | --- | --- |
| RSC96 Ctrl | 7.617802461 | 22.19355392 | 1 |
| RSC96 Ctrl | 8.049489975 | 22.71936099 | 0.936843812 |
| RSC96 Ctrl | 8.506700516 | 23.351 | 0.82999413 |
|  |  |  |  |
| RSC96-0.01μM NMB | 8.155412356 | 22.25721232 | 1.388908442 |
| RSC96-0.01μM NMB | 8.96778965 | 23.07684898 | 1.381937271 |
| RSC96-0.01μM NMB | 9.616583824 | 23.460 | 1.661606122 |
|  |  |  |  |
| RSC96-0.25μM NMB | 8.512650172 | 22.31061459 | 1.714498984 |
| RSC96-0.25μM NMB | 7.981579622 | 21.45141919 | 2.152348796 |
| RSC96-0.25μM NMB | 10.14922047 | 23.716 | 2.012833602 |
|  |  |  |  |
| RSC96-1μM NMB | 9.623436292 | 22.42082405 | 3.430368837 |
| RSC96-1μM NMB | 8.075476011 | 20.80583572 | 3.593505142 |
| RSC96-1μM NMB | 9.851142883 | 22.591 | 3.571009971 |

|  | *18s* Ct value | *NGFR* Ct value | 2^-∆∆Ct^ |
| --- | --- | --- | --- |
| RSC96 Ctrl | 7.617802461 | 30.17326355 | 1.196640776 |
| RSC96 Ctrl | 8.049489975 | 30.86394119 | 1 |
| RSC96 Ctrl | 7.506700516 | 29.708 | 1.530054043 |
|  |  |  |  |
| RSC96-0.01μM NMB | 9.623436292 | 31.34512424 | 2.132821554 |
| RSC96-0.01μM NMB | 8.075476011 | 29.79647446 | 2.133841136 |
| RSC96-0.01μM NMB | 8.504295349 | 29.807 | 2.852429316 |
|  |  |  |  |
| RSC96-0.25μM NMB | 8.822463671 | 29.58800062 | 4.137944438 |
| RSC96-0.25μM NMB | 8.709063848 | 29.66738796 | 3.620344794 |
| RSC96-0.25μM NMB | 7.616583824 | 28.594 | 3.573089057 |
|  |  |  |  |
| RSC96-1μM NMB | 28.60022608 | 9.26765728 | 11.17251761 |
| RSC96-1μM NMB | 28.49538136 | 8.96778965 | 6.493409521 |
| RSC96-1μM NMB | 28.609 | 9.149220467 | 10.22895316 |

|  | *18s* Ct value | *MBP* Ct value | 2^-∆∆Ct^ |
| --- | --- | --- | --- |
| RSC96 Ctrl | 7.617802461 | 34.25085195 | 1 |
| RSC96 Ctrl | 8.049489975 | 34.50726064 | 1.129182615 |
| RSC96 Ctrl | 8.506700516 | 34.249 | 1.854570546 |
|  |  |  |  |
| RSC96-0.01μM NMB | 9.623436292 | 33.9940656 | 4.797956832 |
| RSC96-0.01μM NMB | 8.075476011 | 32.83759435 | 3.657685794 |
| RSC96-0.01μM NMB | 9.504295349 | 33.830 | 4.949492824 |
|  |  |  |  |
| RSC96-0.25μM NMB | 8.822463671 | 31.73450216 | 13.18669393 |
| RSC96-0.25μM NMB | 8.709063848 | 31.75362523 | 12.02936099 |
| RSC96-0.25μM NMB | 10.14922047 | 32.843 | 15.3440538 |
|  |  |  |  |
| RSC96-1μM NMB | 9.26765728 | 31.55250613 | 20.36755133 |
| RSC96-1μM NMB | 8.96778965 | 31.42356586 | 18.0919157 |
| RSC96-1μM NMB | 9.616583824 | 31.855 | 21.03509742 |

|  | *18s* Ct value | *NES* Ct value | 2^-∆∆Ct^ |
| --- | --- | --- | --- |
| RSC96 Ctrl | 7.617802461 | 32.54294586 | 1 |
| RSC96 Ctrl | 8.049489975 | 32.41206932 | 1.476891718 |
| RSC96 Ctrl | 8.049489975 | 32.44458961 | 0.688165518 |
|  |  |  |  |
| RSC96-0.01μM NMB | 8.155412356 | 31.12072182 | 3.890171989 |
| RSC96-0.01μM NMB | 8.075476011 | 31.2634004 | 3.333918913 |
| RSC96-0.01μM NMB | 9.504295349 | 32.088 | 5.06897831 |
|  |  |  |  |
| RSC96-0.25μM NMB | 9.26765728 | 31.03675779 | 8.913814225 |
| RSC96-0.25μM NMB | 8.96778965 | 30.71786308 | 9.03215327 |
| RSC96-0.25μM NMB | 9.616583824 | 31.402 | 8.813400851 |
|  |  |  |  |
| RSC96-1μM NMB | 8.822463671 | 29.90197817 | 14.37638353 |
| RSC96-1μM NMB | 8.709063848 | 29.9304568 | 13.02986699 |
| RSC96-1μM NMB | 10.14922047 | 31.168 | 14.99218647 |

Figure S7l

|  | Number of migration cells/HPF | Number of invasion cells/HPF |
| --- | --- | --- |
| RSC96 Ctrl | 294.1111111 | 141.85 |
| RSC96 Ctrl | 294.8 | 140.9642857 |
| RSC96 Ctrl | 296.9125 | 140.5555556 |
|  |  |  |
| RSC96-0.01μM NMB | 576.15 | 337.25 |
| RSC96-0.01μM NMB | 662.15 | 366.4196429 |
| RSC96-0.01μM NMB | 534.7 | 342.5625 |
|  |  |  |
| RSC96-0.25μM NMB | 895.95 | 531 |
| RSC96-0.25μM NMB | 876.3 | 566.2555556 |
| RSC96-0.25μM NMB | 865.4833333 | 557 |
|  |  |  |
| RSC96-1μM NMB | 886.5 | 632.4642857 |
| RSC96-1μM NMB | 951.4166667 | 736.175 |
| RSC96-1μM NMB | 1108.433333 | 708.6125 |

Figure S7m

|  | *18s* Ct value | *GFAP* Ct value | 2^-∆∆Ct^ |
| --- | --- | --- | --- |
| RSC96 Ctrl | 7.617802461 | 26.41813469 | 1.017451287 |
| RSC96 Ctrl | 8.049489975 | 26.87478193 | 1 |
| RSC96 Ctrl | 8.506700516 | 27.073 | 1.197036748 |
|  |  |  |  |
| RSC96-0.01μM NMB | 8.822463671 | 27.20640882 | 1.357871354 |
| RSC96-0.01μM NMB | 8.075476011 | 26.99032275 | 0.939812729 |
| RSC96-0.01μM NMB | 9.616583824 | 28.168 | 1.209469448 |
|  |  |  |  |
| RSC96-0.25μM NMB | 9.26765728 | 27.5025533 | 1.505659898 |
| RSC96-0.25μM NMB | 8.709063848 | 26.85441271 | 1.602076551 |
| RSC96-0.25μM NMB | 10.14922047 | 28.277 | 1.62188362 |
|  |  |  |  |
| RSC96-1μM NMB | 9.623436292 | 27.2130146 | 2.354978095 |
| RSC96-1μM NMB | 8.96778965 | 26.62345568 | 2.249533604 |
| RSC96-1μM NMB | 9.504295349 | 27.115 | 2.320897641 |

Figure S7n

|  | High (%) | Medium (%) | Low (%) |
| --- | --- | --- | --- |
| RSC96-HeLa sgNC | 70.83 | 20.83 | 8.33 |
| RSC96-HeLa sgNC | 72.22 | 5.56 | 22.22 |
| RSC96-HeLa sgNC | 16.67 | 70.83 | 12.5 |
|  |  |  |  |
| RSC96-HeLa sgNMB | 80.77 | 15.38 | 3.85 |
| RSC96-HeLa sgNMB | 90.91 | 9.09 | 0 |
| RSC96-HeLa sgNMB | 75 | 25 | 0 |
|  |  |  |  |
| RSC96-ME180 sgNC | 14.71 | 20.59 | 64.71 |
| RSC96-ME180 sgNC | 14.29 | 42.86 | 42.86 |
| RSC96-ME180 sgNC | 0 | 59.26 | 40.74 |
|  |  |  |  |
| RSC96-ME180 sgNMB | 91.3 | 8.7 | 0 |
| RSC96-ME180 sgNMB | 35.29 | 58.82 | 5.88 |
| RSC96-ME180 sgNMB | 78.26 | 17.39 | 4.35 |

Figure S7o

|  | Filopodia (%) | Lamellipodia (%) |
| --- | --- | --- |
| RSC96-HeLa sgNC | 25 | 75 |
| RSC96-HeLa sgNC | 27.27 | 72.73 |
| RSC96-HeLa sgNC | 30.77 | 69.23 |
|  |  |  |
| RSC96-HeLa sgNMB | 0 | 100 |
| RSC96-HeLa sgNMB | 6.25 | 93.75 |
| RSC96-HeLa sgNMB | 8.33 | 91.67 |
|  |  |  |
| RSC96-ME180 sgNC | 40 | 60 |
| RSC96-ME180 sgNC | 42.86 | 57.14 |
| RSC96-ME180 sgNC | 43.75 | 56.25 |
|  |  |  |
| RSC96-ME180 sgNMB | 5.88 | 94.12 |
| RSC96-ME180 sgNMB | 6.25 | 93.75 |
| RSC96-ME180 sgNMB | 8.33 | 91.67 |

Figure S7p

|  | EdU positive cells (%) |
| --- | --- |
| RSC96-HeLa sgNC | 10.16 |
| RSC96-HeLa sgNC | 4.51 |
| RSC96-HeLa sgNC | 11.69 |
|  |  |
| RSC96-HeLa sgNMB | 0.43 |
| RSC96-HeLa sgNMB | 0.31 |
| RSC96-HeLa sgNMB | 0.53 |
|  |  |
| RSC96-ME180 sgNC | 12.22 |
| RSC96-ME180 sgNC | 19.78 |
| RSC96-ME180 sgNC | 19.91 |
|  |  |
| RSC96-ME180 sgNMB | 0.58 |
| RSC96-ME180 sgNMB | 0.74 |
| RSC96-ME180 sgNMB | 1.97 |

Figure S7r

|  | *18s* Ct value | *GFAP* Ct value | 2^-∆∆Ct^ |
| --- | --- | --- | --- |
| RSC96 Ctrl | 8.491376559 | 27.1740036 | 0.929007578 |
| RSC96 Ctrl | 8.956150055 | 27.53253937 | 1 |
| RSC96 Ctrl | 7.914620717 | 26.65303675 | 0.893768607 |
|  |  |  |  |
| RSC96-HeLa sgNC | 8.147723516 | 25.72797712 | 1.994650127 |
| RSC96-HeLa sgNC | 9.09617424 | 26.69622866 | 1.967460798 |
| RSC96-HeLa sgNC | 7.683895588 | 25.38781166 | 1.830798785 |
|  |  |  |  |
| RSC96-HeLa sgNMB | 8.338922501 | 26.5780646 | 1.263343723 |
| RSC96-HeLa sgNMB | 9.114169439 | 27.82704353 | 0.909733079 |
| RSC96-HeLa sgNMB | 7.879453182 | 26.17614492 | 1.213940387 |
|  |  |  |  |
| RSC96-ME180 sgNC | 7.851986567 | 25.19276746 | 2.354806343 |
| RSC96-ME180 sgNC | 9.821755091 | 27.14474869 | 2.384018971 |
| RSC96-ME180 sgNC | 7.606717428 | 25.295777 | 1.84974928 |
|  |  |  |  |
| RSC96-ME180 sgNMB | 7.789795081 | 26.28222338 | 1.059924138 |
| RSC96-ME180 sgNMB | 9.587158839 | 28.11406708 | 1.034892614 |
| RSC96-ME180 sgNMB | 7.644901911 | 26.51135572 | 0.817865496 |

Figure S7s,t

|  | Number of migration cells/HPF | Number of invasion cells/HPF |
| --- | --- | --- |
| RSC96-HeLa sgNC | 393.4 | 614.25 |
| RSC96-HeLa sgNC | 378.1 | 611.75 |
| RSC96-HeLa sgNC | 397.6 | 608.6666667 |
|  |  |  |
| RSC96-HeLa sgNMB | 130.2 | 119 |
| RSC96-HeLa sgNMB | 145.4 | 115.2 |
| RSC96-HeLa sgNMB | 116.2 | 110 |
|  |  |  |
| RSC96-ME180 sgNC | 581.9333333 | 817.25 |
| RSC96-ME180 sgNC | 471.2083333 | 822.6 |
| RSC96-ME180 sgNC | 598.9 | 829.8333333 |
|  |  |  |
| RSC96-ME180 sgNMB | 240 | 120.5 |
| RSC96-ME180 sgNMB | 225.9 | 111.4 |
| RSC96-ME180 sgNMB | 243.1 | 120.8888889 |

Figure S7q

|  | *18s* Ct value | *GAP43* Ct value | 2^-∆∆Ct^ |
| --- | --- | --- | --- |
| RSC96 Ctrl | 8.491376559 | 36.40862274 | 1 |
| RSC96 Ctrl | 8.956150055 | 36.32851601 | 1.458899225 |
| RSC96 Ctrl | 7.914620717 | 35.55634308 | 1.2104335 |
|  |  |  |  |
| RSC96-HeLa sgNC | 8.338922501 | 33.65518951 | 6.066982581 |
| RSC96-HeLa sgNC | 9.114169439 | 34.60460154 | 5.37704699 |
| RSC96-HeLa sgNC | 7.879453182 | 33.43344116 | 5.145310721 |
|  |  |  |  |
| RSC96-HeLa sgNMB | 8.147723516 | 34.72937775 | 2.523790141 |
| RSC96-HeLa sgNMB | 9.09617424 | 36.16656303 | 1.798578825 |
| RSC96-HeLa sgNMB | 7.683895588 | 34.08687401 | 2.85653808 |
|  |  |  |  |
| RSC96-ME180 sgNC | 7.789795081 | 33.12121073 | 6.003611117 |
| RSC96-ME180 sgNC | 9.587158839 | 34.60995293 | 7.435615116 |
| RSC96-ME180 sgNC | 7.644901911 | 32.77872467 | 6.884841415 |
|  |  |  |  |
| RSC96-ME180 sgNMB | 7.851986567 | 33.44135857 | 5.020650099 |
| RSC96-ME180 sgNMB | 9.821755091 | 35.31385612 | 5.370830342 |
| RSC96-ME180 sgNMB | 7.606717428 | 33.37077904 | 4.448085665 |

|  | *18s* Ct value | *GDNF* Ct value | 2^-∆∆Ct^ |
| --- | --- | --- | --- |
| RSC96 Ctrl | 8.491376559 | 27.37663269 | 1 |
| RSC96 Ctrl | 8.956150055 | 27.13881683 | 1.627423076 |
| RSC96 Ctrl | 7.914620717 | 25.96869342 | 1.779144185 |
|  |  |  |  |
| RSC96-HeLa sgNC | 8.338922501 | 23.40789318 | 14.08693118 |
| RSC96-HeLa sgNC | 9.114169439 | 24.06781069 | 15.25927883 |
| RSC96-HeLa sgNC | 7.879453182 | 23.03835487 | 13.23562521 |
|  |  |  |  |
| RSC96-HeLa sgNMB | 8.147723516 | 24.23204708 | 6.968907793 |
| RSC96-HeLa sgNMB | 9.09617424 | 25.17013645 | 7.019138251 |
| RSC96-HeLa sgNMB | 7.683895588 | 23.54241816 | 8.149624329 |
|  |  |  |  |
| RSC96-ME180 sgNC | 7.789795081 | 24.20110607 | 5.555609284 |
| RSC96-ME180 sgNC | 9.587158839 | 25.61963717 | 7.223899385 |
| RSC96-ME180 sgNC | 7.644901911 | 24.09398079 | 5.412057825 |
|  |  |  |  |
| RSC96-ME180 sgNMB | 7.851986567 | 22.95323372 | 13.77527275 |
| RSC96-ME180 sgNMB | 9.821755091 | 25.12209765 | 11.99959303 |
| RSC96-ME180 sgNMB | 7.606717428 | 23.00908343 | 11.18032429 |

|  | *18s* Ct value | *NGFR* Ct value | 2^-∆∆Ct^ |
| --- | --- | --- | --- |
| RSC96 Ctrl | 8.491376559 | 31.10604858 | 1 |
| RSC96 Ctrl | 8.956150055 | 31.26013438 | 1.240298782 |
| RSC96 Ctrl | 7.914620717 | 30.15080833 | 1.299975477 |
|  |  |  |  |
| RSC96-HeLa sgNC | 8.338922501 | 29.84077644 | 2.162676805 |
| RSC96-HeLa sgNC | 9.114169439 | 30.40322876 | 2.506393101 |
| RSC96-HeLa sgNC | 7.879453182 | 29.45995808 | 2.047931032 |
|  |  |  |  |
| RSC96-HeLa sgNMB | 8.147723516 | 30.66156642 | 1.072389593 |
| RSC96-HeLa sgNMB | 9.09617424 | 31.54606628 | 1.120995107 |
| RSC96-HeLa sgNMB | 7.683895588 | 30.20238686 | 1.068939912 |
|  |  |  |  |
| RSC96-ME180 sgNC | 7.789795081 | 29.03163528 | 2.589784083 |
| RSC96-ME180 sgNC | 9.587158839 | 30.81986936 | 2.606224756 |
| RSC96-ME180 sgNC | 7.644901911 | 28.90618992 | 2.555107549 |
|  |  |  |  |
| RSC96-ME180 sgNMB | 7.851986567 | 30.15097809 | 1.244598579 |
| RSC96-ME180 sgNMB | 9.821755091 | 31.93048096 | 1.420054362 |
| RSC96-ME180 sgNMB | 7.606717428 | 30.11065197 | 1.079780062 |

|  | *18s* Ct value | *MBP* Ct value | 2^-∆∆Ct^ |
| --- | --- | --- | --- |
| RSC96 Ctrl | 8.491376559 | 33.60627365 | 1 |
| RSC96 Ctrl | 8.956150055 | 33.55577596 | 1.429262776 |
| RSC96 Ctrl | 7.914620717 | 32.45431709 | 1.489884731 |
|  |  |  |  |
| RSC96-HeLa sgNC | 8.338922501 | 31.36556625 | 4.252329346 |
| RSC96-HeLa sgNC | 9.114169439 | 31.70913696 | 5.735540977 |
| RSC96-HeLa sgNC | 7.879453182 | 30.82636261 | 4.493961187 |
|  |  |  |  |
| RSC96-HeLa sgNMB | 8.147723516 | 32.57814598 | 1.607116626 |
| RSC96-HeLa sgNMB | 9.09617424 | 33.62396622 | 1.502229376 |
| RSC96-HeLa sgNMB | 7.683895588 | 32.76490148 | 1.023769691 |
|  |  |  |  |
| RSC96-ME180 sgNC | 7.789795081 | 31.2748003 | 3.094898021 |
| RSC96-ME180 sgNC | 9.587158839 | 33.72468758 | 5.301780619 |
| RSC96-ME180 sgNC | 7.644901911 | 31.24477482 | 2.858036191 |
|  |  |  |  |
| RSC96-ME180 sgNMB | 7.851986567 | 31.4636488 | 2.834776242 |
| RSC96-ME180 sgNMB | 9.821755091 | 32.53017521 | 1.96887068 |
| RSC96-ME180 sgNMB | 7.606717428 | 31.9070797 | 1.758730982 |

|  | *18s* Ct value | *NES* Ct value | 2^-∆∆Ct^ |
| --- | --- | --- | --- |
| RSC96 Ctrl | 8.491376559 | 32.20694033 | 1 |
| RSC96 Ctrl | 8.956150055 | 32.67067337 | 1.000721451 |
| RSC96 Ctrl | 7.914620717 | 31.81038666 | 0.882579311 |
|  |  |  |  |
| RSC96-HeLa sgNC | 8.338922501 | 29.09700966 | 7.767641491 |
| RSC96-HeLa sgNC | 9.114169439 | 29.63622665 | 9.148318275 |
| RSC96-HeLa sgNC | 7.879453182 | 29.11724981 | 5.570346798 |
|  |  |  |  |
| RSC96-HeLa sgNMB | 8.147723516 | 29.79899597 | 4.182284829 |
| RSC96-HeLa sgNMB | 9.09617424 | 31.08841578 | 3.301959059 |
| RSC96-HeLa sgNMB | 7.683895588 | 31.09256045 | 1.237045797 |
|  |  |  |  |
| RSC96-ME180 sgNC | 7.789795081 | 28.01716709 | 11.22148547 |
| RSC96-ME180 sgNC | 9.587158839 | 29.75411669 | 11.70137303 |
| RSC96-ME180 sgNC | 7.644901911 | 27.79327679 | 11.85307026 |
|  |  |  |  |
| RSC96-ME180 sgNMB | 7.851986567 | 29.73511314 | 3.561382018 |
| RSC96-ME180 sgNMB | 9.821755091 | 31.59940434 | 3.831513876 |
| RSC96-ME180 sgNMB | 7.606717428 | 30.7342968 | 1.503145227 |

Figure 4a

Sciatic nerve Ctrl

DAPI

NMBR

GFAP

Merge

Sciatic nerve-HeLa

DAPI

NMBR

GFAP

Merge

Sciatic nerve-ME180

DAPI

NMBR

GFAP

Merge

Figure 4b

HeLa

| Distance | GFAP | NMBR |
| --- | --- | --- |
| 0 | 12 | 10 |
| 0.641 | 15.854 | 11.83 |
| 1.282 | 18.761 | 13.426 |
| 1.922 | 13.735 | 15.761 |
| 2.563 | 23.163 | 17.873 |
| 3.204 | 49.091 | 17.428 |
| 3.845 | 105.851 | 36.874 |
| 4.486 | 100.674 | 45.336 |
| 5.127 | 86.716 | 45.494 |
| 5.767 | 77.431 | 50.003 |
| 6.408 | 73.823 | 53.031 |
| 7.049 | 84.446 | 52.977 |
| 7.69 | 90.921 | 51.991 |
| 8.331 | 81.103 | 54.843 |
| 8.971 | 93.833 | 60.679 |
| 9.612 | 104.649 | 80.838 |
| 10.253 | 117.802 | 96.22 |
| 10.894 | 130.703 | 101.401 |
| 11.535 | 141.081 | 95.499 |
| 12.175 | 144.689 | 96.527 |
| 12.816 | 137.439 | 113.398 |
| 13.457 | 98.802 | 127.818 |
| 14.098 | 93.476 | 122.839 |
| 14.739 | 109.694 | 114.211 |
| 15.38 | 128.327 | 100.291 |
| 16.02 | 137.47 | 76.896 |
| 16.661 | 160.464 | 68.773 |
| 17.302 | 190.26 | 80.955 |
| 17.943 | 103.525 | 132.053 |
| 18.584 | 39.572 | 175.282 |
| 19.224 | 18.465 | 205.259 |
| 19.865 | 14.337 | 203.648 |
| 20.506 | 15.826 | 170.607 |
| 21.147 | 15.368 | 132.916 |
| 21.788 | 11.016 | 121.995 |
| 22.429 | 15.037 | 125.383 |
| 23.069 | 19.49 | 123.239 |
| 23.71 | 16.772 | 124.951 |
| 24.351 | 12.997 | 123.645 |
| 24.992 | 15.018 | 121.509 |
| 25.633 | 18.428 | 130.689 |
| 26.273 | 21.18 | 144.497 |
| 26.914 | 20.552 | 159.741 |
| 27.555 | 21.263 | 167.203 |
| 28.196 | 25.079 | 161.149 |
| 28.837 | 30.755 | 145.781 |
| 29.477 | 41.594 | 138.263 |
| 30.118 | 57.775 | 136.579 |
| 30.759 | 71.651 | 133.436 |
| 31.4 | 95.393 | 153.781 |
| 32.041 | 158.592 | 172.971 |
| 32.682 | 196.651 | 170.415 |
| 33.322 | 212.159 | 172.869 |
| 33.963 | 225.082 | 168.171 |
| 34.604 | 232.593 | 147.941 |
| 35.245 | 239.168 | 142.285 |
| 35.886 | 245.838 | 155.888 |
| 36.526 | 249.667 | 156.284 |
| 37.167 | 253.238 | 151.307 |
| 37.808 | 253.498 | 147.984 |
| 38.449 | 250.887 | 143.213 |
| 39.09 | 248.751 | 149.476 |
| 39.731 | 248.294 | 152.923 |
| 40.371 | 250.129 | 139.397 |
| 41.012 | 251.784 | 125.476 |
| 41.653 | 250.594 | 122.572 |
| 42.294 | 245.77 | 127.028 |
| 42.935 | 240.415 | 132.406 |
| 43.575 | 235.81 | 130.922 |
| 44.216 | 236.63 | 129.826 |
| 44.857 | 238.599 | 132.53 |
| 45.498 | 239.919 | 135.61 |
| 46.139 | 243.016 | 138.61 |
| 46.779 | 248.362 | 142.303 |
| 47.42 | 251.294 | 142.647 |
| 48.061 | 250.032 | 138.514 |
| 48.702 | 246.795 | 133.433 |
| 49.343 | 242.171 | 127.619 |
| 49.984 | 243.895 | 123.363 |
| 50.624 | 249.721 | 119.945 |
| 51.265 | 251.531 | 118.07 |
| 51.906 | 251.25 | 119.364 |
| 52.547 | 250.105 | 118.324 |
| 53.188 | 250.482 | 112.997 |
| 53.828 | 252.805 | 111.498 |
| 54.469 | 251.963 | 117.741 |
| 55.11 | 247.669 | 121.522 |
| 55.751 | 241.009 | 118.354 |
| 56.392 | 230.557 | 119.949 |
| 57.033 | 224.053 | 122.107 |
| 57.673 | 228.725 | 119.171 |
| 58.314 | 238.063 | 114.606 |
| 58.955 | 244.202 | 116.378 |
| 59.596 | 245.749 | 124.089 |
| 60.237 | 247.172 | 127.769 |
| 60.877 | 244.72 | 124.085 |
| 61.518 | 239.595 | 112.031 |
| 62.159 | 231.412 | 97.051 |
| 62.8 | 219.565 | 95.669 |
| 63.441 | 208.129 | 102.18 |
| 64.082 | 201.803 | 102.865 |
| 64.722 | 187.322 | 101.165 |
| 65.363 | 170.794 | 99.9 |
| 66.004 | 155.599 | 100.006 |
| 66.645 | 151.196 | 97.416 |
| 67.286 | 156.573 | 86.86 |
| 67.926 | 161.987 | 69.032 |
| 68.567 | 146.747 | 49.045 |
| 69.208 | 100.681 | 34.684 |
| 69.849 | 82.312 | 36.811 |
| 70.49 | 72.968 | 55.803 |
| 71.13 | 61.671 | 76.039 |
| 71.771 | 50.25 | 78.957 |
| 72.412 | 39.205 | 69.535 |
| 73.053 | 27.012 | 61.62 |
| 73.694 | 11.464 | 61.905 |
| 74.335 | 11.368 | 73.572 |
| 74.975 | 12.402 | 98.289 |
| 75.616 | 14.693 | 123.662 |
| 76.257 | 16.503 | 135.709 |
| 76.898 | 16.497 | 136.226 |
| 77.539 | 13.904 | 129.215 |
| 78.179 | 17.546 | 122.665 |
| 78.82 | 20.459 | 148.671 |
| 79.461 | 17.681 | 176.058 |
| 80.102 | 12.69 | 174.218 |
| 80.743 | 11.131 | 138.113 |
| 81.384 | 13.506 | 105.212 |
| 82.024 | 17.049 | 116.233 |
| 82.665 | 17.25 | 155.399 |
| 83.306 | 20.353 | 209.319 |
| 83.947 | 20.118 | 227.425 |
| 84.588 | 14.928 | 203.055 |
| 85.228 | 10.855 | 172.223 |
| 85.869 | 12.523 | 180.393 |
| 86.51 | 16.693 | 212.76 |
| 87.151 | 17.713 | 206.653 |
| 87.792 | 12.36 | 52.935 |
| 88.432 | 8.875 | 18.59 |
| 89.073 | 5.988 | 19.868 |
| 89.714 | 5.65 | 16.685 |
| 90.355 | 9.501 | 8.656 |
| 90.996 | 12.95 | 6.079 |
| 91.637 | 9.807 | 11.357 |
| 92.277 | 6.092 | 14.367 |
| 92.918 | 7.164 | 20.258 |
| 93.559 | 5.897 | 19.343 |
| 94.2 | 7.578 | 9.869 |
| 94.841 | 10.374 | 7.612 |
| 95.481 | 10.332 | 16.256 |
| 96.122 | 7.072 | 27.356 |
| 96.763 | 9.82 | 60.217 |
| 97.404 | 9.58 | 78.274 |
| 98.045 | 11.756 | 95.104 |
| 98.686 | 17.683 | 114.237 |
| 99.326 | 17.817 | 131.708 |
| 99.967 | 14.014 | 129.506 |
| 100.608 | 14.153 | 106.155 |
| 101.249 | 8.406 | 48.543 |
| 101.89 | 6.27 | 24.935 |
| 102.53 | 5.145 | 12.205 |
| 103.171 | 4.762 | 5.982 |
| 103.812 | 8.157 | 3.816 |
| 104.453 | 12.01 | 4.362 |
| 105.094 | 12.7 | 4.449 |
| 105.734 | 10.338 | 12.214 |
| 106.375 | 11.09 | 118.938 |
| 107.016 | 14.488 | 181.36 |
| 107.657 | 18.677 | 176.885 |
| 108.298 | 17.167 | 150.186 |
| 108.939 | 13.555 | 142.195 |
| 109.579 | 13.322 | 141.708 |
| 110.22 | 12.797 | 127.512 |
| 110.861 | 14.564 | 165.931 |
| 111.502 | 14.624 | 191.212 |
| 112.143 | 11.034 | 169.054 |
| 112.783 | 12.466 | 149.421 |
| 113.424 | 18.201 | 162.006 |
| 114.065 | 18.661 | 168.865 |
| 114.706 | 13.039 | 138.74 |
| 115.347 | 13.765 | 92.038 |
| 115.988 | 17.346 | 118.651 |
| 116.628 | 18.56 | 160.023 |
| 117.269 | 16.873 | 173.746 |
| 117.91 | 15.933 | 162.488 |
| 118.551 | 16.374 | 146.977 |
| 119.192 | 16.856 | 147.958 |
| 119.832 | 16.016 | 182.791 |
| 120.473 | 14.091 | 194.456 |
| 121.114 | 11.912 | 184.051 |
| 121.755 | 14.183 | 163.982 |
| 122.396 | 15.224 | 149.757 |
| 123.036 | 15.032 | 160.193 |
| 123.677 | 17.051 | 187.672 |
| 124.318 | 22.498 | 188.458 |
| 124.959 | 24.569 | 183.457 |
| 125.6 | 21.148 | 176.172 |
| 126.241 | 16.235 | 140.103 |
| 126.881 | 11.481 | 82.657 |
| 127.522 | 9.114 | 47.897 |
| 128.163 | 9.103 | 54.921 |
| 128.804 | 12.392 | 79.611 |
| 129.445 | 15.912 | 85.026 |
| 130.085 | 13.435 | 83.734 |
| 130.726 | 13.028 | 91.525 |
| 131.367 | 14 | 97.798 |
| 132.008 | 13.771 | 85.92 |
| 132.649 | 13.379 | 63.371 |
| 133.29 | 13.395 | 72.172 |
| 133.93 | 14.014 | 158.827 |
| 134.571 | 12.943 | 195.681 |
| 135.212 | 12.091 | 162.201 |
| 135.853 | 14.355 | 163.146 |
| 136.494 | 19.312 | 190.635 |
| 137.134 | 22.769 | 189.995 |
| 137.775 | 21.894 | 166.449 |
| 138.416 | 22.163 | 120.457 |
| 139.057 | 20.876 | 62.576 |
| 139.698 | 16.129 | 48.682 |
| 140.338 | 14.912 | 88.832 |
| 140.979 | 16.469 | 136.369 |
| 141.62 | 15.898 | 137.152 |
| 142.261 | 11.706 | 106.892 |
| 142.902 | 11.739 | 132.058 |
| 143.543 | 17.633 | 198.142 |
| 144.183 | 21.703 | 223.269 |
| 144.824 | 22.081 | 222.682 |
| 145.465 | 23.321 | 209.364 |
| 146.106 | 22.683 | 177.851 |
| 146.747 | 17.093 | 132.025 |
| 147.387 | 12.833 | 92.151 |
| 148.028 | 13.388 | 83.428 |
| 148.669 | 12.7 | 101.843 |
| 149.31 | 12.234 | 105.231 |
| 149.951 | 15.583 | 88.139 |
| 150.592 | 19.559 | 85.747 |
| 151.232 | 21.552 | 113.183 |
| 151.873 | 21.403 | 144.455 |
| 152.514 | 16.987 | 137.087 |
| 153.155 | 11.889 | 81.016 |
| 153.796 | 11.111 | 40.141 |
| 154.436 | 13.534 | 30.841 |
| 155.077 | 19.402 | 36.237 |
| 155.718 | 22.258 | 44.702 |
| 156.359 | 19.537 | 74.784 |
| 157 | 20.189 | 143.92 |
| 157.64 | 21.794 | 209.143 |
| 158.281 | 20.342 | 192.141 |
| 158.922 | 20.482 | 178.339 |
| 159.563 | 22.625 | 188.814 |
| 160.204 | 23.31 | 203.673 |
| 160.845 | 18.837 | 185.711 |
| 161.485 | 11.582 | 128.035 |
| 162.126 | 7.707 | 67.37 |
| 162.767 | 8.408 | 50.689 |
| 163.408 | 9.739 | 44.678 |
| 164.049 | 13.523 | 69.674 |
| 164.689 | 18.42 | 134.037 |
| 165.33 | 18.126 | 182.38 |
| 165.971 | 15.004 | 146.392 |
| 166.612 | 14.453 | 45.9 |
| 167.253 | 12.051 | 69.744 |
| 167.894 | 11.984 | 104.549 |
| 168.534 | 14.537 | 110.459 |
| 169.175 | 16.406 | 103.315 |
| 169.816 | 17.597 | 126.546 |
| 170.457 | 20.631 | 178.483 |
| 171.098 | 16.931 | 183.458 |
| 171.738 | 9.169 | 87.277 |
| 172.379 | 5.404 | 21.517 |
| 173.02 | 4.355 | 7.123 |
| 173.661 | 4.92 | 5.533 |
| 174.302 | 5.508 | 2.32 |
| 174.943 | 5.037 | 1.33 |
| 175.583 | 8.227 | 1.648 |
| 176.224 | 12.816 | 6.63 |
| 176.865 | 23.17 | 22.342 |
| 177.506 | 45.586 | 45.413 |
| 178.147 | 79.3 | 70.119 |
| 178.787 | 99.631 | 82.601 |
| 179.428 | 97.75 | 85.653 |
| 180.069 | 86.934 | 80.406 |
| 180.71 | 62.979 | 59.169 |
| 181.351 | 39.614 | 53.638 |
| 181.991 | 21.673 | 50.192 |
| 182.632 | 18.746 | 57.164 |
| 183.273 | 23.491 | 68.159 |
| 183.914 | 35.179 | 72.104 |
| 184.555 | 52.124 | 72.126 |
| 185.196 | 38.881 | 76.571 |
| 185.836 | 36.209 | 67.749 |
| 186.477 | 32.71 | 56.948 |
| 187.118 | 30.522 | 54.181 |
| 187.759 | 32.533 | 65.988 |
| 188.4 | 54.184 | 83.995 |
| 189.04 | 87.63 | 88.287 |
| 189.681 | 67.865 | 82.722 |
| 190.322 | 31.235 | 60.93 |
| 190.963 | 13.808 | 56.207 |
| 191.604 | 13.6 | 76.308 |
| 192.245 | 14.512 | 92.029 |
| 192.885 | 23.127 | 85.712 |
| 193.526 | 45.732 | 78.48 |
| 194.167 | 134.031 | 108.589 |
| 194.808 | 194.811 | 137.126 |
| 195.449 | 236.115 | 142.885 |
| 196.089 | 251.13 | 123.324 |
| 196.73 | 251.389 | 109.406 |
| 197.371 | 251.465 | 105.28 |
| 198.012 | 254.51 | 100.693 |
| 198.653 | 251.464 | 114.047 |
| 199.293 | 236.133 | 123.65 |
| 199.934 | 204.108 | 142.411 |
| 200.575 | 167.564 | 183.861 |
| 201.216 | 119.035 | 228.446 |
| 201.857 | 62.601 | 243.953 |
| 202.498 | 28.351 | 225.791 |
| 203.138 | 17.904 | 183.711 |
| 203.779 | 23.173 | 152.864 |
| 204.42 | 21.611 | 151.74 |
| 205.061 | 19.088 | 159.411 |
| 205.702 | 15.674 | 164.351 |
| 206.342 | 14.258 | 166.525 |
| 206.983 | 16.035 | 183.203 |
| 207.624 | 15.549 | 214.852 |
| 208.265 | 9.781 | 218.274 |
| 208.906 | 7.254 | 194.749 |
| 209.547 | 10.235 | 195.156 |
| 210.187 | 15.594 | 219.025 |
| 210.828 | 18.201 | 237.325 |
| 211.469 | 21.457 | 237.857 |
| 212.11 | 25.928 | 222.664 |
| 212.751 | 43.94 | 167.545 |
| 213.391 | 61.377 | 110.674 |
| 214.032 | 83.109 | 71.574 |
| 214.673 | 101.027 | 59.265 |
| 215.314 | 106.522 | 69.732 |
| 215.955 | 105.993 | 88.407 |
| 216.595 | 86.716 | 106.518 |
| 217.236 | 44.193 | 104.981 |
| 217.877 | 28.233 | 93.79 |
| 218.518 | 31.017 | 73.585 |
| 219.159 | 32.652 | 46.422 |
| 219.8 | 29.031 | 28.852 |
| 220.44 | 24.071 | 28.639 |
| 221.081 | 23.433 | 40.388 |
| 221.722 | 20.59 | 37.52 |
| 222.363 | 15.626 | 24.372 |
| 223.004 | 13.001 | 19 |
| 223.644 | 14.799 | 24.892 |
| 224.285 | 18.808 | 34.097 |
| 224.926 | 18.822 | 34.482 |
| 225.567 | 15.052 | 25.917 |
| 226.208 | 11.005 | 16.685 |
| 226.849 | 10.97 | 10.007 |
| 227.489 | 10.898 | 8.904 |
| 228.13 | 8.345 | 11.655 |
| 228.771 | 7.605 | 12.062 |
| 229.412 | 9.331 | 10.62 |
| 230.053 | 12.704 | 10.569 |
| 230.693 | 19.768 | 11.447 |
| 231.334 | 45.709 | 14.277 |
| 231.975 | 84.977 | 18.335 |
| 232.616 | 117.258 | 20.26 |
| 233.257 | 137.028 | 19.865 |
| 233.897 | 135.851 | 21.088 |
| 234.538 | 120.033 | 30.934 |
| 235.179 | 116.49 | 47.605 |
| 235.82 | 107.193 | 48.232 |
| 236.461 | 64.779 | 29.79 |
| 237.102 | 29.253 | 17.463 |
| 237.742 | 16.567 | 15.406 |
| 238.383 | 15.576 | 22.228 |
| 239.024 | 20.602 | 28.188 |
| 239.665 | 25.188 | 29.835 |
| 240.306 | 21.882 | 27.864 |
| 240.946 | 18.064 | 22.952 |
| 241.587 | 22.982 | 20.669 |
| 242.228 | 26.908 | 23.132 |
| 242.869 | 26.042 | 25.298 |
| 243.51 | 22.053 | 21.845 |
| 244.151 | 19.132 | 17.92 |
| 244.791 | 19.601 | 18.074 |
| 245.432 | 22.554 | 15.718 |
| 246.073 | 26.769 | 13.122 |
| 246.714 | 30.564 | 13.756 |
| 247.355 | 30.517 | 11.291 |
| 247.995 | 26.746 | 6.339 |
| 248.636 | 22.581 | 4.387 |
| 249.277 | 18.618 | 4.621 |
| 249.918 | 11.849 | 5.198 |
| 250.559 | 8.234 | 6.455 |
| 251.199 | 6.723 | 7.304 |
| 251.84 | 6.558 | 8.237 |
| 252.481 | 7.647 | 8.744 |
| 253.122 | 8.012 | 8.24 |
| 253.763 | 9.103 | 6.555 |
| 254.404 | 9.532 | 5.641 |
| 255.044 | 6.743 | 6.098 |
| 255.685 | 7.271 | 6.379 |
| 256.326 | 10.227 | 7.549 |
| 256.967 | 13.742 | 11.406 |
| 257.608 | 15.801 | 17.078 |
| 258.248 | 16.588 | 20.238 |
| 258.889 | 19.422 | 26.095 |
| 259.53 | 30.748 | 46.823 |
| 260.171 | 28.298 | 59.148 |
| 260.812 | 14.853 | 61.766 |
| 261.453 | 10.058 | 65.227 |
| 262.093 | 13.24 | 69.938 |
| 262.734 | 14.808 | 80.169 |
| 263.375 | 15.344 | 107.371 |
| 264.016 | 22.327 | 166.216 |
| 264.657 | 21.54 | 168.642 |
| 265.297 | 20.175 | 153.359 |
| 265.938 | 23.22 | 142.506 |
| 266.579 | 22.04 | 128.831 |
| 267.22 | 16.667 | 94.553 |
| 267.861 | 13.366 | 62.032 |
| 268.501 | 14.645 | 46.777 |
| 269.142 | 12.252 | 44.046 |
| 269.783 | 14.59 | 53.689 |
| 270.424 | 16.257 | 73.837 |
| 271.065 | 14.059 | 86.128 |
| 271.706 | 12.188 | 76.514 |
| 272.346 | 9 | 59.335 |
| 272.987 | 9.567 | 48.222 |
| 273.628 | 17.131 | 51.085 |
| 274.269 | 22.389 | 57.525 |
| 274.91 | 28.638 | 62.373 |
| 275.55 | 34.097 | 75.159 |
| 276.191 | 29.772 | 60.71 |
| 276.832 | 16.216 | 26.172 |
| 277.473 | 13.717 | 14.869 |
| 278.114 | 36.444 | 17.063 |
| 278.755 | 31.671 | 17.034 |
| 279.395 | 26.172 | 21.357 |
| 280.036 | 23.9 | 26.046 |
| 280.677 | 21.694 | 30.205 |
| 281.318 | 19.723 | 32.747 |
| 281.959 | 15.103 | 30.831 |
| 282.599 | 16.101 | 23.745 |
| 283.24 | 20.33 | 19.191 |
| 283.881 | 17.056 | 13.265 |
| 284.522 | 12.099 | 10.28 |
| 285.163 | 12.222 | 9.789 |
| 285.803 | 17.714 | 13.484 |
| 286.444 | 24.017 | 19.497 |
| 287.085 | 16.771 | 27.626 |
| 287.726 | 14.363 | 34.611 |
| 288.367 | 14.283 | 56.705 |
| 289.008 | 16.934 | 88.132 |
| 289.648 | 21.973 | 85.379 |
| 290.289 | 26.51 | 58.125 |
| 290.93 | 26.31 | 43.142 |
| 291.571 | 23.835 | 52.957 |
| 292.212 | 25.902 | 73.931 |
| 292.852 | 27.855 | 88.344 |
| 293.493 | 20.175 | 97.083 |
| 294.134 | 11.572 | 79.801 |
| 294.775 | 17.848 | 50.297 |
| 295.416 | 32.21 | 41.118 |
| 296.057 | 74.931 | 61.952 |
| 296.697 | 114.016 | 82.067 |
| 297.338 | 120.533 | 78.041 |
| 297.979 | 89.857 | 48.718 |
| 298.62 | 45.034 | 25.73 |
| 299.261 | 18.904 | 18.283 |
| 299.901 | 13.458 | 18.016 |
| 300.542 | 11.661 | 18.734 |
| 301.183 | 10.086 | 20.666 |
| 301.824 | 18.525 | 23.853 |
| 302.465 | 25.927 | 23.81 |
| 303.105 | 21.58 | 24.489 |
| 303.746 | 12.688 | 26.323 |
| 304.387 | 9.739 | 27.401 |
| 305.028 | 12.74 | 30.689 |
| 305.669 | 14.74 | 43.046 |
| 306.31 | 13.171 | 44.422 |
| 306.95 | 11.878 | 35.871 |
| 307.591 | 11.477 | 28.825 |
| 308.232 | 14.329 | 27.164 |
| 308.873 | 17.051 | 27.923 |
| 309.514 | 17.938 | 26.78 |
| 310.154 | 18.655 | 26.757 |
| 310.795 | 14.455 | 34.76 |
| 311.436 | 11.218 | 46.249 |
| 312.077 | 11.231 | 58.161 |
| 312.718 | 11.976 | 58.807 |
| 313.359 | 12.586 | 40.982 |
| 313.999 | 12.496 | 23.911 |
| 314.64 | 10.051 | 14.154 |
| 315.281 | 8.518 | 11.588 |
| 315.922 | 8.007 | 13.824 |
| 316.563 | 9.165 | 17.293 |
| 317.203 | 11.188 | 16.804 |
| 317.844 | 11.609 | 14.745 |
| 318.485 | 11.048 | 15.343 |
| 319.126 | 12.314 | 19.572 |
| 319.767 | 11.979 | 21.531 |
| 320.408 | 12.231 | 19.389 |
| 321.048 | 14.653 | 15.808 |
| 321.689 | 16.101 | 13.154 |
| 322.33 | 16.193 | 12.289 |
| 322.971 | 16.902 | 12.547 |
| 323.612 | 18.571 | 14.071 |
| 324.252 | 23.697 | 17.347 |
| 324.893 | 26.859 | 20.027 |
| 325.534 | 22.137 | 21.074 |
| 326.175 | 14.988 | 24.617 |
| 326.816 | 12.508 | 29.557 |
| 327.456 | 16.624 | 28.989 |
| 328.097 | 24.85 | 25.461 |
| 328.738 | 36.468 | 26.671 |
| 329.379 | 45.671 | 29.23 |
| 330.02 | 41.322 | 27.723 |
| 330.661 | 28.974 | 25.645 |
| 331.301 | 18.132 | 23.879 |
| 331.942 | 12.141 | 22.583 |
| 332.583 | 11.202 | 20.953 |
| 333.224 | 10.741 | 15.763 |
| 333.865 | 12.957 | 9.728 |
| 334.505 | 20.257 | 11.134 |
| 335.146 | 28.631 | 13.579 |
| 335.787 | 35.777 | 14.03 |
| 336.428 | 35.877 | 16.249 |
| 337.069 | 27.646 | 19.665 |
| 337.71 | 18.188 | 20.835 |
| 338.35 | 15.623 | 18.434 |
| 338.991 | 16.108 | 14.194 |
| 339.632 | 14.183 | 11.015 |
| 340.273 | 12.306 | 9.239 |
| 340.914 | 16.547 | 10.138 |
| 341.554 | 25.538 | 13.435 |
| 342.195 | 29.59 | 15.945 |
| 342.836 | 23.827 | 17.157 |
| 343.477 | 21.402 | 16.461 |
| 344.118 | 21.46 | 14.702 |
| 344.758 | 19.941 | 14.203 |
| 345.399 | 17.692 | 12.557 |
| 346.04 | 17.569 | 12.198 |
| 346.681 | 24.607 | 15.041 |
| 347.322 | 38.226 | 17.736 |
| 347.963 | 53.689 | 20.647 |
| 348.603 | 62.191 | 18.633 |
| 349.244 | 57.584 | 13.797 |
| 349.885 | 34.345 | 10.261 |
| 350.526 | 18.722 | 11.806 |
| 351.167 | 19.897 | 19.072 |
| 351.807 | 25.07 | 29.517 |
| 352.448 | 23.533 | 37.941 |
| 353.089 | 21.737 | 31.821 |
| 353.73 | 29.137 | 27.452 |
| 354.371 | 38.531 | 23.17 |
| 355.012 | 38.069 | 13.501 |
| 355.652 | 28.425 | 6.31 |
| 356.293 | 25.387 | 7.185 |
| 356.934 | 32.91 | 11.751 |
| 357.575 | 34.492 | 9.914 |
| 358.216 | 40.134 | 10.401 |
| 358.856 | 36.745 | 12.238 |
| 359.497 | 23.394 | 7.734 |
| 360.138 | 18.437 | 4.138 |
| 360.779 | 30.566 | 4.368 |
| 361.42 | 72.182 | 7.343 |
| 362.06 | 54.828 | 8.366 |
| 362.701 | 28.127 | 8.014 |
| 363.342 | 14.302 | 7.8 |
| 363.983 | 12.265 | 10.494 |
| 364.624 | 15.284 | 14.455 |
| 365.265 | 15.033 | 18.06 |
| 365.905 | 6.019 | 11.445 |
| 366.546 | 6.781 | 8.555 |
| 367.187 | 6.537 | 8.397 |
| 367.828 | 6.088 | 9.632 |
| 368.469 | 7.901 | 10.42 |
| 369.109 | 9.861 | 11.488 |
| 369.75 | 7.839 | 10.798 |
| 370.391 | 5.843 | 13.018 |
| 371.032 | 6.598 | 13.783 |
| 371.673 | 8.471 | 10.176 |
| 372.314 | 10.582 | 9.459 |
| 372.954 | 10.003 | 6.36 |
| 373.595 | 8.549 | 6.232 |
| 374.236 | 8.442 | 7.096 |
| 374.877 | 6.084 | 6.044 |
| 375.518 | 11.458 | 5.287 |
| 376.158 | 13.424 | 4.055 |
| 376.799 | 10.657 | 4 |
| 377.44 | 13.07 | 2.563 |
| 378.081 | 27.806 | 7.664 |
| 378.722 | 52.526 | 21.173 |
| 379.362 | 72.089 | 44.265 |
| 380.003 | 57.292 | 110.629 |
| 380.644 | 61.338 | 114.679 |
| 381.285 | 123.791 | 86.12 |
| 381.926 | 208.093 | 61.269 |
| 382.567 | 242.396 | 62.459 |
| 383.207 | 242.115 | 77.716 |
| 383.848 | 244.782 | 85.124 |
| 384.489 | 166.36 | 69.925 |
| 385.13 | 69.287 | 42.383 |
| 385.771 | 16.44 | 19.651 |
| 386.411 | 6.493 | 9.11 |
| 387.052 | 6.019 | 7.648 |
| 387.693 | 6.167 | 5.451 |
| 388.334 | 6.792 | 3.735 |
| 388.975 | 9.725 | 5.539 |
| 389.616 | 8.801 | 5.803 |
| 390.256 | 7.332 | 5.781 |
| 390.897 | 6.999 | 5.195 |
| 391.538 | 6.723 | 6.525 |
| 392.179 | 8.157 | 6.159 |
| 392.82 | 8.492 | 5.216 |
| 393.46 | 6.578 | 5.777 |
| 394.101 | 5.779 | 5.54 |
| 394.742 | 5.386 | 3.54 |
| 395.383 | 5.92 | 3 |
| 396.024 | 6.044 | 3.816 |
| 396.664 | 6.115 | 4.63 |
| 397.305 | 6.195 | 3.873 |
| 397.946 | 6.774 | 3.1 |
| 398.587 | 8.246 | 3.314 |
| 399.228 | 10.36 | 4.588 |
| 399.869 | 10.63 | 13.825 |
| 400.509 | 6.988 | 29.247 |
| 401.15 | 4.445 | 28.799 |
| 401.791 | 4.033 | 14.548 |
| 402.432 | 7.084 | 9.473 |
| 403.073 | 10.472 | 12.212 |
| 403.713 | 8.462 | 13.117 |
| 404.354 | 8.336 | 13.699 |
| 404.995 | 16.45 | 22.881 |
| 405.636 | 28.096 | 47.744 |
| 406.277 | 35.493 | 74.311 |
| 406.918 | 38.27 | 74.776 |
| 407.558 | 40.823 | 44.223 |
| 408.199 | 35.228 | 26.815 |
| 408.84 | 27.054 | 27.262 |
| 409.481 | 20.902 | 28.112 |
| 410.122 | 17.418 | 27.714 |
| 410.762 | 15.027 | 26.45 |
| 411.403 | 11.291 | 21.879 |
| 412.044 | 9.65 | 17.242 |
| 412.685 | 14.179 | 19.647 |
| 413.326 | 27.51 | 27.275 |
| 413.966 | 42.97 | 32.308 |
| 414.607 | 45.532 | 33.132 |
| 415.248 | 43.679 | 38.483 |
| 415.889 | 50.715 | 56.741 |
| 416.53 | 43.432 | 73.657 |
| 417.171 | 44.219 | 75.163 |
| 417.811 | 96.846 | 70.251 |
| 418.452 | 178.152 | 62.319 |
| 419.093 | 214.536 | 48.14 |
| 419.734 | 167.849 | 34.823 |
| 420.375 | 96.505 | 30.424 |
| 421.015 | 92.174 | 34.972 |
| 421.656 | 174.465 | 60.392 |
| 422.297 | 228.435 | 82.247 |
| 422.938 | 201.718 | 73.937 |
| 423.579 | 169.709 | 65.394 |
| 424.22 | 133.205 | 62.698 |
| 424.86 | 91.625 | 62.369 |
| 425.501 | 63.36 | 61.276 |
| 426.142 | 69.181 | 59.147 |
| 426.783 | 84.604 | 52.74 |
| 427.424 | 86.89 | 45.74 |
| 428.064 | 107.284 | 56.581 |
| 428.705 | 150.416 | 75.873 |
| 429.346 | 187.41 | 83.966 |
| 429.987 | 197.33 | 86.716 |
| 430.628 | 194.754 | 90.915 |
| 431.268 | 186.168 | 101.543 |
| 431.909 | 162.403 | 108.938 |
| 432.55 | 139.878 | 99.721 |
| 433.191 | 134.884 | 93.142 |
| 433.832 | 132.914 | 96.9 |
| 434.473 | 112.213 | 96.839 |
| 435.113 | 67.657 | 74.145 |
| 435.754 | 14.037 | 41.602 |
| 436.395 | 10.149 | 33.376 |
| 437.036 | 9.893 | 21.75 |
| 437.677 | 10 | 12.548 |
| 438.317 | 9.828 | 8.922 |
| 438.958 | 9.888 | 12.539 |
| 439.599 | 9.544 | 19.642 |
| 440.24 | 8.337 | 27.101 |
| 440.881 | 9.399 | 28.054 |
| 441.522 | 8.547 | 21.696 |
| 442.162 | 8.356 | 15.282 |
| 442.803 | 8.212 | 14.999 |
| 443.444 | 7.902 | 16.86 |
| 444.085 | 9.375 | 24.775 |
| 444.726 | 15.417 | 36.584 |
| 445.366 | 19.5 | 54.554 |
| 446.007 | 19.568 | 73.845 |
| 446.648 | 19.794 | 76.05 |
| 447.289 | 19.035 | 60.927 |
| 447.93 | 19.385 | 46.835 |
| 448.571 | 16.673 | 33.196 |
| 449.211 | 11.142 | 19.613 |
| 449.852 | 8.186 | 8.728 |
| 450.493 | 8.916 | 7.811 |
| 451.134 | 8.523 | 8.454 |
| 451.775 | 11.559 | 15.702 |
| 452.415 | 15.022 | 37.351 |
| 453.056 | 15.246 | 66.582 |
| 453.697 | 16.95 | 82.198 |
| 454.338 | 20.42 | 67.728 |
| 454.979 | 22.156 | 53.375 |
| 455.619 | 17.453 | 43.519 |
| 456.26 | 12.51 | 52.966 |
| 456.901 | 10.33 | 76.558 |
| 457.542 | 12.413 | 97.7 |
| 458.183 | 33.786 | 110.721 |
| 458.824 | 125.469 | 121.623 |
| 459.464 | 158.016 | 124.616 |
| 460.105 | 170.231 | 134.723 |
| 460.746 | 187.396 | 130.389 |
| 461.387 | 216.048 | 129.407 |
| 462.028 | 238.995 | 136.752 |
| 462.668 | 248.883 | 133.061 |
| 463.309 | 243.756 | 83.858 |
| 463.95 | 179.941 | 75.048 |
| 464.591 | 73.135 | 98.973 |
| 465.232 | 16.484 | 95.747 |
| 465.873 | 0 | 0 |

Figure 4c

ME180

| Distance | GFAP | NMBR |
| --- | --- | --- |
| 0 | 2 | 12 |
| 1.351 | 2.008 | 9.099 |
| 2.703 | 2.466 | 12.35 |
| 4.054 | 2.705 | 13.084 |
| 5.405 | 3 | 13.146 |
| 6.757 | 3.626 | 14.241 |
| 8.108 | 3.104 | 15.518 |
| 9.459 | 2.338 | 10.33 |
| 10.811 | 2.006 | 6.255 |
| 12.162 | 2.233 | 8.04 |
| 13.514 | 30.798 | 17.674 |
| 14.865 | 141.513 | 60.594 |
| 16.216 | 205.451 | 85.171 |
| 17.568 | 95.849 | 39.701 |
| 18.919 | 22.68 | 18.578 |
| 20.27 | 83.146 | 37.748 |
| 21.622 | 179.909 | 63.644 |
| 22.973 | 174.362 | 61.126 |
| 24.324 | 123.881 | 51.153 |
| 25.676 | 44.878 | 25.174 |
| 27.027 | 0.027 | 7.256 |
| 28.378 | 0.363 | 9.457 |
| 29.73 | 12.584 | 13.954 |
| 31.081 | 27.721 | 24.578 |
| 32.432 | 34.194 | 25.111 |
| 33.784 | 106.806 | 39.675 |
| 35.135 | 140.894 | 56.943 |
| 36.486 | 188.31 | 65.127 |
| 37.838 | 204.44 | 73.59 |
| 39.189 | 52.81 | 27.931 |
| 40.541 | 0.227 | 4.664 |
| 41.892 | 3.037 | 4.802 |
| 43.243 | 25.619 | 17.299 |
| 44.595 | 126.257 | 44.597 |
| 45.946 | 180.129 | 59.567 |
| 47.297 | 212.114 | 67.134 |
| 48.649 | 171.872 | 48.747 |
| 50 | 47.019 | 27.03 |
| 51.351 | 6.202 | 16.082 |
| 52.703 | 8.36 | 11.179 |
| 54.054 | 115.765 | 46.587 |
| 55.405 | 160.153 | 75.371 |
| 56.757 | 67.145 | 34.754 |
| 58.108 | 7.784 | 10.078 |
| 59.459 | 22.635 | 17.818 |
| 60.811 | 113.066 | 36.561 |
| 62.162 | 95.38 | 31.417 |
| 63.514 | 52.946 | 21.072 |
| 64.865 | 57.404 | 28.726 |
| 66.216 | 78.535 | 45.012 |
| 67.568 | 133.51 | 61.016 |
| 68.919 | 155.023 | 53.661 |
| 70.27 | 66.088 | 30.056 |
| 71.622 | 3.071 | 11.512 |
| 72.973 | 16.117 | 15.827 |
| 74.324 | 40.16 | 21.579 |
| 75.676 | 43.011 | 23.989 |
| 77.027 | 20.525 | 16.178 |
| 78.378 | 38.575 | 20.275 |
| 79.73 | 68.635 | 28.65 |
| 81.081 | 54.509 | 25.38 |
| 82.432 | 162.739 | 53.082 |
| 83.784 | 201.764 | 79.666 |
| 85.135 | 133.963 | 49.642 |
| 86.486 | 98.905 | 30.75 |
| 87.838 | 219.359 | 115.021 |
| 89.189 | 237.929 | 164.06 |
| 90.541 | 244.246 | 185.562 |
| 91.892 | 245.648 | 167.497 |
| 93.243 | 251.805 | 187.611 |
| 94.595 | 252.017 | 194.696 |
| 95.946 | 253.269 | 200.096 |
| 97.297 | 252.057 | 200.523 |
| 98.649 | 253.792 | 205.872 |
| 100 | 248.832 | 193.986 |
| 101.351 | 247.9 | 194.869 |
| 102.703 | 253.375 | 223.687 |
| 104.054 | 255 | 230.568 |
| 105.405 | 255 | 232.445 |
| 106.757 | 243.044 | 194.209 |
| 108.108 | 165.417 | 92.207 |
| 109.459 | 111.378 | 33.078 |
| 110.811 | 182.975 | 69.45 |
| 112.162 | 226.507 | 89.721 |
| 113.514 | 218.359 | 76.988 |
| 114.865 | 215.199 | 82.471 |
| 116.216 | 234.113 | 110.328 |
| 117.568 | 238.011 | 142.877 |
| 118.919 | 231.155 | 132.99 |
| 120.27 | 198.724 | 84.006 |
| 121.622 | 194.764 | 82.076 |
| 122.973 | 244.152 | 167.502 |
| 124.324 | 253.594 | 180.44 |
| 125.676 | 248.058 | 147.688 |
| 127.027 | 248.495 | 173.88 |
| 128.378 | 206.03 | 91.363 |
| 129.73 | 52.842 | 21.764 |
| 131.081 | 183.868 | 76.936 |
| 132.432 | 236.18 | 133.282 |
| 133.784 | 246.431 | 157.89 |
| 135.135 | 248.621 | 130.831 |
| 136.486 | 252.847 | 177.183 |
| 137.838 | 254.349 | 201.177 |
| 139.189 | 249.55 | 150.382 |
| 140.541 | 250.693 | 127.604 |
| 141.892 | 253.851 | 198.137 |
| 143.243 | 253.642 | 219.81 |
| 144.595 | 252.43 | 198.401 |
| 145.946 | 253.34 | 197.42 |
| 147.297 | 254.286 | 187.212 |
| 148.649 | 253.414 | 173.808 |
| 150 | 254.571 | 192.99 |
| 151.351 | 254.364 | 215.949 |
| 152.703 | 254.856 | 226.111 |
| 154.054 | 254.611 | 218.661 |
| 155.405 | 254.711 | 220.132 |
| 156.757 | 244.354 | 181.61 |
| 158.108 | 215.04 | 115.339 |
| 159.459 | 152.83 | 57.058 |
| 160.811 | 127.448 | 46.678 |
| 162.162 | 236.896 | 114.342 |
| 163.514 | 238.491 | 136.38 |
| 164.865 | 188.672 | 79.278 |
| 166.216 | 217.703 | 120.134 |
| 167.568 | 213.268 | 124.271 |
| 168.919 | 142.355 | 55.538 |
| 170.27 | 135.65 | 51.593 |
| 171.622 | 180.713 | 73.752 |
| 172.973 | 206.789 | 84.583 |
| 174.324 | 215.887 | 80.324 |
| 175.676 | 231.914 | 86.399 |
| 177.027 | 148.527 | 46.87 |
| 178.378 | 52.515 | 28.304 |
| 179.73 | 107.549 | 49.4 |
| 181.081 | 88.059 | 41.797 |
| 182.432 | 19.522 | 15.197 |
| 183.784 | 1.343 | 8.181 |
| 185.135 | 2.834 | 8.631 |
| 186.486 | 2.782 | 8.343 |
| 187.838 | 2.319 | 9.945 |
| 189.189 | 4.16 | 11.351 |
| 190.541 | 42.303 | 17.463 |
| 191.892 | 137.83 | 34.887 |
| 193.243 | 192.503 | 55.963 |
| 194.595 | 182.349 | 59.669 |
| 195.946 | 121.644 | 42.466 |
| 197.297 | 47.252 | 21.223 |
| 198.649 | 2.006 | 5.712 |
| 200 | 6.646 | 11.023 |
| 201.351 | 38.919 | 35.324 |
| 202.703 | 85.402 | 56.19 |
| 204.054 | 72.728 | 54.029 |
| 205.405 | 22.435 | 26.966 |
| 206.757 | 20.756 | 9.997 |
| 208.108 | 76.255 | 22.76 |
| 209.459 | 122.456 | 113.418 |
| 210.811 | 99.673 | 106.622 |
| 212.162 | 2.564 | 9.525 |
| 213.514 | 15.314 | 12.188 |
| 214.865 | 36.963 | 27.29 |
| 216.216 | 173.622 | 85.125 |
| 217.568 | 126.336 | 50.232 |
| 218.919 | 182.121 | 83.509 |
| 220.27 | 200.397 | 79.119 |
| 221.622 | 60.334 | 29.011 |
| 222.973 | 5.368 | 8.998 |
| 224.324 | 0.475 | 8.459 |
| 225.676 | 1.409 | 9.719 |
| 227.027 | 1.403 | 11.337 |
| 228.378 | 1.775 | 8.097 |
| 229.73 | 2 | 7.654 |
| 231.081 | 2.813 | 11.147 |
| 232.432 | 4.116 | 11.628 |
| 233.784 | 3.188 | 9.249 |
| 235.135 | 1.397 | 8.792 |
| 236.486 | 4.474 | 9.882 |
| 237.838 | 18.472 | 14.558 |
| 239.189 | 48.562 | 20.043 |
| 240.541 | 80.198 | 31.429 |
| 241.892 | 105.718 | 50.977 |
| 243.243 | 72.522 | 44.564 |
| 244.595 | 37.544 | 21.018 |
| 245.946 | 32.016 | 14.423 |
| 247.297 | 12.163 | 9.263 |
| 248.649 | 1.447 | 6.747 |
| 250 | 8.898 | 9.708 |
| 251.351 | 11.34 | 13.125 |
| 252.703 | 3.54 | 9.216 |
| 254.054 | 0.992 | 9.792 |
| 255.405 | 0.024 | 7.489 |
| 256.757 | 0.587 | 5.966 |
| 258.108 | 4.641 | 8.008 |
| 259.459 | 56.948 | 24.878 |
| 260.811 | 122.261 | 52.907 |
| 262.162 | 147.576 | 64.922 |
| 263.514 | 112.875 | 40.878 |
| 264.865 | 49.57 | 18.206 |
| 266.216 | 10.118 | 8.673 |
| 267.568 | 6.247 | 6.272 |
| 268.919 | 49.896 | 18.774 |
| 270.27 | 63.311 | 27.188 |
| 271.622 | 54.785 | 27.913 |
| 272.973 | 120.435 | 48.48 |
| 274.324 | 136.211 | 48.882 |
| 275.676 | 70.894 | 27.59 |
| 277.027 | 3.25 | 6.627 |
| 278.378 | 0.256 | 5.229 |
| 279.73 | 0.37 | 6.57 |
| 281.081 | 3.001 | 8.128 |
| 282.432 | 26.851 | 17.754 |
| 283.784 | 70.537 | 31.406 |
| 285.135 | 98.383 | 32.309 |
| 286.486 | 79.559 | 32.727 |
| 287.838 | 69.402 | 31.268 |
| 289.189 | 57.837 | 30.647 |
| 290.541 | 36.775 | 14.809 |
| 291.892 | 166.807 | 62.102 |
| 293.243 | 180.605 | 77.672 |
| 294.595 | 96.919 | 47.387 |
| 295.946 | 89.56 | 41.813 |
| 297.297 | 91.986 | 38.063 |
| 298.649 | 65.371 | 22.455 |
| 300 | 50.746 | 19.598 |
| 301.351 | 33.998 | 18.978 |
| 302.703 | 25.165 | 20.307 |
| 304.054 | 51.972 | 26.742 |
| 305.405 | 106.444 | 50.108 |
| 306.757 | 95.871 | 41.824 |
| 308.108 | 41.337 | 22.33 |
| 309.459 | 20.241 | 16.062 |
| 310.811 | 42.655 | 35.055 |
| 312.162 | 183.542 | 178.339 |
| 313.514 | 201.434 | 194.421 |
| 314.865 | 90.628 | 74.35 |
| 316.216 | 45.107 | 21.893 |
| 317.568 | 82.661 | 39.89 |
| 318.919 | 98.445 | 49.108 |
| 320.27 | 60.522 | 31.09 |
| 321.622 | 44.583 | 21.093 |
| 322.973 | 57.396 | 23.731 |
| 324.324 | 119.294 | 41.088 |
| 325.676 | 181.662 | 67.929 |
| 327.027 | 224.293 | 94.102 |
| 328.378 | 226.348 | 93.583 |
| 329.73 | 165.083 | 53.17 |
| 331.081 | 146.373 | 56.615 |
| 332.432 | 174.174 | 60.617 |
| 333.784 | 149.63 | 47.745 |
| 335.135 | 76.22 | 35.554 |
| 336.486 | 38.497 | 26.839 |
| 337.838 | 51.188 | 26.207 |
| 339.189 | 23 | 11 |

Figure 4d

NMB

NMBR

DAPI

Merge

Figure 4e

DAPI

Pan-CK

NMBR

NMB

GFAP

Merge

Figure 4f

Figure 4g

PBS

HeLa without PD168368

HeLa with PD168368

ME180 without PD168368

ME180 with PD168368

Figure 4h

| SFI | PBS  (n=5) | HeLa  (n=5) | HeLa PD168368  (n=5) | ME180  (n=5) | ME180 PD168368  (n=5) |
| --- | --- | --- | --- | --- | --- |
| 0 days | 10 | 10 | 10 | 10 | 10 |
|  | 10 | 9 | 10 | 10 | 11 |
|  | 10 | 9 | 10.5 | 10 | 10.5 |
|  | 11 | 10 | 10 | 11 | 10 |
|  | 10 | 10 | 10 | 10 | 10 |
| 3days | 10 | 7 | 9.5 | 6 | 8 |
|  | 10 | 8 | 9 | 5 | 11 |
|  | 8 | 7 | 10 | 7 | 9 |
|  | 10 | 8 | 9 | 4.5 | 10 |
|  | 10 | 5 | 9 | 6 | 9 |
| 1week | 10 | 3 | 7 | 3 | 8 |
|  | 10 | 6 | 10 | 7 | 9 |
|  | 10 | 7 | 9 | 5 | 7 |
|  | 11 | 3 | 8 | 5 | 10 |
|  | 10 | 4 | 8 | 4 | 12 |
| 2weeks | 9 | 0 | 6 | 0 | 5 |
|  | 10 | 2 | 5 | 0 | 6 |
|  | 10 | 5 | 10 | 3 | 8 |
|  | 7 | 3 | 5 | 4 | 10 |
|  | 10 | 3 | 6 | 3 | 6 |
| 5weeks | 8.5 | 0 | 5 | 3 | 6 |
|  | 9 | 0 | 8 | 2 | 7 |
|  | 9 | 3 | 8 | 2 | 8 |
|  | 10 | 3 | 6 | 1 | 8 |
|  | 9 | 3 | 5 |  |  |

Figure 4j

| Sciatic nerve score | PBS  (n=5) | HeLa  (n=5) | HeLa PD168368  (n=5) | ME180  (n=5) | ME180 PD168368  (n=5) |
| --- | --- | --- | --- | --- | --- |
| 0 days | 4 | 4 | 4 | 4 | 4 |
|  | 4 | 4 | 4 | 4 | 4 |
|  | 4 | 4 | 4 | 4 | 4 |
|  | 4 | 4 | 4 | 4 | 4 |
|  | 4 | 4 | 4 | 4 | 4 |
| 3days | 4 | 3 | 4 | 3 | 4 |
|  | 4 | 3 | 4 | 2 | 4 |
|  | 4 | 3 | 4 | 3 | 4 |
|  | 4 | 3 | 4 | 2 | 4 |
|  | 4 | 2 | 4 | 3 | 4 |
| 1week | 4 | 2 | 3 | 1 | 3 |
|  | 4 | 3 | 4 | 3 | 3 |
|  | 4 | 1 | 3 | 2 | 2 |
|  | 4 | 1 | 3 | 1 | 4 |
|  | 4 | 1 | 3 | 1 | 4 |
| 2weeks | 4 | 1 | 3 | 1 | 2 |
|  | 4 | 2 | 2 | 1 | 3 |
|  | 4 | 1 | 4 | 1 | 4 |
|  | 4 | 1 | 2 | 2 | 4 |
|  | 4 | 1 | 3 | 1 | 2 |
| 5weeks | 4 | 1 | 3 | 1 | 2 |
|  | 4 | 1 | 4 | 2 | 3 |
|  | 4 | 1 | 4 | 1 | 4 |
|  | 4 | 1 | 3 | 1 | 4 |
|  | 4 | 3 | 2 |  |  |

Figure 4l

| Length of PNI (mm) | HeLa  (n=5) | HeLa PD168368  (n=5) | ME180  (n=5) | ME180 PD168368  (n=5) |
| --- | --- | --- | --- | --- |
| 3 days | 1.693 | 1.271 | 2.008 | 1.164 |
|  | 1.675 | 1.661 | 2.711 | 1.328 |
|  | 1.964 | 1.155 | 3.374 | 1.092 |
|  | 1.477 | 0.967 | 3.231 | 1.289 |
|  | 1.955 | 1.513 | 3.551 | 1.037 |

Figure 4m

| Weight of tumor (g) | HeLa  (n=5) | HeLa PD168368  (n=5) | ME180  (n=5) | ME180 PD168368  (n=5) |
| --- | --- | --- | --- | --- |
| 3 days | 0.00628 | 0.005 | 0.00516 | 0.00367 |
|  | 0.00467 | 0.00579 | 0.00619 | 0.00378 |
|  | 0.00408 | 0.00462 | 0.00447 | 0.00269 |
|  | 0.00564 | 0.00423 | 0.00408 | 0.00256 |
|  | 0.00468 | 0.00502 | 0.00273 | 0.00313 |

Figure 4n

Vehicle

PD168368

Figure 4o

Intra-cervical cancer nerve number

| Vehicle  (n=5) | PD168368  (n=5) |
| --- | --- |
| 42 | 6 |
| 34 | 1 |
| 29 | 4 |
| 27 | 4 |
| 19 | 3 |

Figure S8a

NMBR

GAPDH

Figure S8b

NMBR

β-Tubulin

Figure S8c

Non-PNI

PNI

Figure S8d

PBS

HeLa

HeLa+PD168368

ME180

ME180+PD168368

Figure S8e

PBS

HeLa

HeLa+PD168368

ME180

ME180+PD168368

Figure S8f

Figure S8g

PBS

HeLa

HeLa+PD168368

ME180

ME180+PD168368

Figure S8h

PBS

HeLa

HeLa+PD168368

ME180

ME180+PD168368

Figure S8i

PBS

HeLa

HeLa+PD168368

ME180

ME180+PD168368

Figure S7j

PBS

HeLa

HeLa+PD168368

ME180

ME180+PD168368

Figure S8k

PBS

HeLa

HeLa+PD168368

ME180

ME180+PD168368

Figure S8l

Figure S8o

| Weight of tumor (g) | HeLa  (n=5) | HeLa PD168368  (n=5) | ME180  (n=5) | ME180 PD168368  (n=5) |
| --- | --- | --- | --- | --- |
| One week | 0.06936 | 0.02022 | 0.03542 | 0.11223 |
|  | 0.00321 | 0.00317 | 0.11639 | 0.01998 |
|  | 0.05451 | 0.1639 | 0.0272 | 0.01896 |
|  | 0.05147 | 0.01648 | 0.03231 | 0.01895 |
|  | 0.03859 | 0.02626 | 0.07282 | 0.00923 |

Figure S9a

PBS

HeLa

HeLa+PD168368

ME180

ME180+PD168368

Figure S9b

PBS

HeLa

HeLa+PD168368

ME180

ME180+PD168368

Figure S9c

PBS

HeLa

HeLa+PD168368

ME180

ME180+PD168368

Figure S9d

Figure S9g

| Weight of tumor (g) | HeLa  (n=5) | HeLa PD168368  (n=5) | ME180  (n=5) | ME180 PD168368  (n=5) |
| --- | --- | --- | --- | --- |
| Two weeks | 0.08809 | 0.01491 | 0.26964 | 0.023264 |
|  | 0.03899 | 0.11386 | 0.06948 | 0.08786 |
|  | 0.02926 | 0.003 | 0.05489 | 0.01228 |
|  | 0.02086 | 0.09515 | 0.02811 | 0.0377 |
|  | 0.2643 | 0.07513 | 0.04971 | 0.06739 |

Figure S9h

PBS

HeLa

HeLa+PD168368

ME180

ME180+PD168368

Figure S9i,j

PBS

HeLa

HeLa+PD168368

ME180

ME180+PD168368

Figure S9k

Figure S9n

| Weight of tumor (g) | HeLa  (n=5) | HeLa PD168368  (n=5) | ME180  (n=4) | ME180 PD168368  (n=4) |
| --- | --- | --- | --- | --- |
| Five weeks | 0.50128 | 0.0713 | 0.17628 | 0.12829 |
|  | 0.17838 | 0.03 | 0.41611 | 0.19332 |
|  | 0.59083 | 0.0024 | 0.32954 | 0.16555 |
|  | 0.25937 | 0.10151 | 0.35116 | 0.08703 |
|  | 0.22249 | 0.11901 |  |  |

Figure 5a

RSC96 Ctrl

RSC96+0.03μM PD168368

RSC96+1μM PD168368

RSC96+5μM PD168368

Figure 5b

RSC96 Ctrl

RSC96+0.03μM PD168368

RSC96+1μM PD168368

RSC96+5μM PD168368

Figure 5c

Migration

RSC96 Ctrl

RSC96+0.03μM PD168368

RSC96+1μM PD168368

RSC96+5μM PD168368

Invasion

RSC96 Ctrl

RSC96+0.03μM PD168368

RSC96+1μM PD168368

RSC96+5μM PD168368

Figure 5d

RSC96 Ctrl

RSC96+NMB

RSC96+PD168368

RSC96+NMB+PD168368

Figure 5e

RSC96 Ctrl

RSC96+NMB

RSC96+PD168368

RSC96+NMB+PD168368

Figure 5f

Migration

RSC96 Ctrl

RSC96+NMB

RSC96+PD168368

RSC96+NMB+PD168368

Invasion

RSC96 Ctrl

RSC96+NMB

RSC96+PD168368

RSC96+NMB+PD168368

Figure 5g

RSC96 siNC

RSC96 siNC +NMB

RSC96 siNMBR

RSC96 siNMBR +NMB

Figure 5h

RSC96 siNC

RSC96 siNC +NMB

RSC96 siNMBR

RSC96 siNMBR +NMB

Figure 5i

Migration

RSC96 siNC

RSC96 siNC +NMB

RSC96 siNMBR

RSC96 siNMBR +NMB

Invasion

RSC96 siNC

RSC96 siNC +NMB

RSC96 siNMBR

RSC96 siNMBR +NMB

Figure S10a

|  | High (%) | Medium (%) | Low (%) |
| --- | --- | --- | --- |
| RSC96-Ctrl | 84.38 | 9.38 | 6.25 |
| RSC96-Ctrl | 86.96 | 4.35 | 8.7 |
| RSC96-Ctrl | 85.71 | 7.14 | 7.14 |
|  |  |  |  |
| RSC96-0.03μM PD168368 | 80.95 | 9.52 | 9.52 |
| RSC96-0.03μM PD168368 | 83.33 | 10 | 6.67 |
| RSC96-0.03μM PD168368 | 84.21 | 5.26 | 10.53 |
|  |  |  |  |
| RSC96-1μM PD168368 | 93.33 | 6.67 | 0 |
| RSC96-1μM PD168368 | 86.67 | 6.67 | 6.67 |
| RSC96-1μM PD168368 | 87.5 | 6.25 | 6.25 |
|  |  |  |  |
| RSC96-5μM PD168368 | 83.33 | 16.67 | 0 |
| RSC96-5μM PD168368 | 90 | 10 | 0 |
| RSC96-5μM PD168368 | 100 | 0 | 0 |

Figure S10b

|  | Filopodia (%) | Lamellipodia (%) |
| --- | --- | --- |
| RSC96-Ctrl | 87.5 | 12.5 |
| RSC96-Ctrl | 90 | 10 |
| RSC96-Ctrl | 88.89 | 11.111 |
|  |  |  |
| RSC96-0.03μM PD168368 | 86.36 | 13.636 |
| RSC96-0.03μM PD168368 | 90.91 | 9.091 |
| RSC96-0.03μM PD168368 | 88.89 | 11.111 |
|  |  |  |
| RSC96-1μM PD168368 | 94.12 | 5.882 |
| RSC96-1μM PD168368 | 92.86 | 7.143 |
| RSC96-1μM PD168368 | 92.86 | 7.143 |
|  |  |  |
| RSC96-5μM PD168368 | 100 | 0 |
| RSC96-5μM PD168368 | 100 | 0 |
| RSC96-5μM PD168368 | 100 | 0 |

Figure S10c

|  | EdU positive cells (%) |
| --- | --- |
| RSC96-Ctrl | 34.60389081 |
| RSC96-Ctrl | 35.96864774 |
| RSC96-Ctrl | 55.70805595 |
|  |  |
| RSC96-0.03μM PD168368 | 41.69239094 |
| RSC96-0.03μM PD168368 | 40.10606402 |
| RSC96-0.03μM PD168368 | 42.36630428 |
|  |  |
| RSC96-1μM PD168368 | 23.96445359 |
| RSC96-1μM PD168368 | 23.30195259 |
| RSC96-1μM PD168368 | 21.75284374 |
|  |  |
| RSC96-5μM PD168368 | 5.819033401 |
| RSC96-5μM PD168368 | 7.837792649 |
| RSC96-5μM PD168368 | 6.868280673 |

Figure 10d

|  | Number of migration cells/HPF | Number of invasion cells/HPF |
| --- | --- | --- |
| RSC96-Ctrl | 698.6 | 756.0416667 |
| RSC96-Ctrl | 768.7 | 767.8263889 |
| RSC96-Ctrl | 781.7 | 772.3611111 |
|  |  |  |
| RSC96-0.03μM PD168368 | 460.8 | 307.5833333 |
| RSC96-0.03μM PD168368 | 653.4 | 315.1571429 |
| RSC96-0.03μM PD168368 | 458.4 | 275.45 |
|  |  |  |
| RSC96-1μM PD168368 | 220.6 | 158.6 |
| RSC96-1μM PD168368 | 376.6 | 96.32142857 |
| RSC96-1μM PD168368 | 366 | 100.7071429 |
|  |  |  |
| RSC96-5μM PD168368 | 25.8 | 7.35 |
| RSC96-5μM PD168368 | 34.6 | 6.016666667 |
| RSC96-5μM PD168368 | 48.2 | 4.6875 |

Figure 10e

|  | High (%) | Medium (%) | Low (%) |
| --- | --- | --- | --- |
| RSC96-Ctrl | 60 | 30 | 10 |
| RSC96-Ctrl | 75 | 20 | 5 |
| RSC96-Ctrl | 81.48 | 11.11 | 7.41 |
|  |  |  |  |
| RSC96-NMB | 58.33 | 20.83 | 20.83 |
| RSC96-NMB | 65.22 | 13.04 | 21.74 |
| RSC96-NMB | 52.63 | 26.32 | 21.05 |
|  |  |  |  |
| RSC96-PD168368 | 90 | 10 | 0 |
| RSC96-PD168368 | 92.31 | 7.69 | 0 |
| RSC96-PD168368 | 91.67 | 8.33 | 0 |
|  |  |  |  |
| RSC96-NMB+ PD168368 | 80.95 | 14.29 | 4.76 |
| RSC96-NMB+ PD168368 | 84.85 | 12.12 | 3.03 |
| RSC96-NMB+ PD168368 | 84.21 | 10.53 | 5.26 |

Figure S10f

|  | Filopodia (%) | Lamellipodia (%) |
| --- | --- | --- |
| RSC96-Ctrl | 88.89 | 11.11 |
| RSC96-Ctrl | 90 | 10 |
| RSC96-Ctrl | 94.12 | 5.88 |
|  |  |  |
| RSC96-NMB | 53.85 | 46.154 |
| RSC96-NMB | 69.23 | 30.769 |
| RSC96-NMB | 75 | 25 |
|  |  |  |
| RSC96-PD168368 | 92.86 | 7.143 |
| RSC96-PD168368 | 100 | 0 |
| RSC96-PD168368 | 100 | 0 |
|  |  |  |
| RSC96-NMB+ PD168368 | 90.91 | 9.091 |
| RSC96-NMB+ PD168368 | 92.86 | 7.143 |
| RSC96-NMB+ PD168368 | 83.33 | 16.667 |

Figure S10g

|  | EdU positive cells (%) |
| --- | --- |
| RSC96-Ctrl | 32.57036408 |
| RSC96-Ctrl | 32.79604684 |
| RSC96-Ctrl | 32.76825094 |
|  |  |
| RSC96-NMB | 60.69857549 |
| RSC96-NMB | 58.88594492 |
| RSC96-NMB | 61.56327656 |
|  |  |
| RSC96-PD168368 | 19.71491656 |
| RSC96-PD168368 | 20.34643483 |
| RSC96-PD168368 | 17.4269952 |
|  |  |
| RSC96-NMB+ PD168368 | 27.95706756 |
| RSC96-NMB+ PD168368 | 29.31891314 |
| RSC96-NMB+ PD168368 | 29.25812982 |

Figure S10h

|  | Number of migration cells/HPF | Number of invasion cells/HPF |
| --- | --- | --- |
| RSC96-Ctrl | 98.625 | 91.76923077 |
| RSC96-Ctrl | 97.5 | 88.83333333 |
| RSC96-Ctrl | 100.3333333 | 87.11111111 |
|  |  |  |
| RSC96-NMB | 453.7777778 | 842.3684211 |
| RSC96-NMB | 485.4 | 866.4285714 |
| RSC96-NMB | 494.2857143 | 822.6 |
|  |  |  |
| RSC96-PD168368 | 22.4 | 20.11111111 |
| RSC96-PD168368 | 27 | 19.69230769 |
| RSC96-PD168368 | 19.8 | 36 |
|  |  |  |
| RSC96-NMB+ PD168368 | 48.83333333 | 41.8 |
| RSC96-NMB+ PD168368 | 29.5 | 51.75 |
| RSC96-NMB+ PD168368 | 32.5 | 57.33333333 |

Figure S10i

|  | *18s* Ct value | *cJUN* Ct value | 2^-∆∆Ct^ |
| --- | --- | --- | --- |
| RSC96-Ctrl | 20.05140877 | 7.617802461 | 1 |
| RSC96-Ctrl | 20.04067993 | 8.049489975 | 1.358878389 |
| RSC96-Ctrl | 20.412 | 8.506700516 | 1.441752583 |
|  |  |  |  |
| RSC96-NMB | 19.2830677 | 8.822463671 | 3.925842446 |
| RSC96-NMB | 19.33803749 | 8.709063848 | 3.493401987 |
| RSC96-NMB | 19.968 | 9.616583824 | 4.2358046 |
|  |  |  |  |
| RSC96-PD168368 | 20.79960632 | 8.512650172 | 1.106996112 |
| RSC96-PD168368 | 20.10548846 | 7.981579622 | 1.239447762 |
| RSC96-PD168368 | 22.111 | 10.22934055 | 1.466367675 |
|  |  |  |  |
| RSC96-NMB+ PD168368 | 20.12462425 | 8.155412356 | 1.379738069 |
| RSC96-NMB+ PD168368 | 19.8617293 | 8.075476011 | 1.566291802 |
| RSC96-NMB+ PD168368 | 21.038 | 9.504295349 | 1.86535038 |

|  | *18s* Ct value | *BDNF* Ct value | 2^-∆∆Ct^ |
| --- | --- | --- | --- |
| RSC96-Ctrl | 21.82262993 | 7.617802461 | 1.382692646 |
| RSC96-Ctrl | 22.72179794 | 8.049489975 | 1 |
| RSC96-Ctrl | 22.921 | 8.506700516 | 1.196232018 |
|  |  |  |  |
| RSC96-NMB | 22.00542068 | 9.26765728 | 3.822574352 |
| RSC96-NMB | 21.70177078 | 8.709063848 | 3.203393513 |
| RSC96-NMB | 22.839 | 10.22934055 | 4.178335098 |
|  |  |  |  |
| RSC96-PD168368 | 22.80850029 | 8.512650172 | 1.298150679 |
| RSC96-PD168368 | 22.57974688 | 8.075476011 | 1.123528792 |
| RSC96-PD168368 | 23.512 | 9.616583824 | 1.713805705 |
|  |  |  |  |
| RSC96-NMB+ PD168368 | 22.15996997 | 8.155412356 | 1.588593889 |
| RSC96-NMB+ PD168368 | 21.98736254 | 7.981579622 | 1.587245234 |
| RSC96-NMB+ PD168368 | 24.242 | 10.14922047 | 1.493882084 |

|  | *18s* Ct value | *GAP43* Ct value | 2^-∆∆Ct^ |
| --- | --- | --- | --- |
| RSC96-Ctrl | 29.22471237 | 7.617802461 | 1.410735386 |
| RSC96-Ctrl | 30.15284729 | 8.049489975 | 1 |
| RSC96-Ctrl | 31.091 | 8.506700516 | 0.716586477 |
|  |  |  |  |
| RSC96-NMB | 30.58553886 | 8.822463671 | 1.26600414 |
| RSC96-NMB | 30.00175858 | 8.709063848 | 1.754016826 |
| RSC96-NMB | 30.923 | 9.851142883 | 2.044515871 |
|  |  |  |  |
| RSC96-PD168368 | 30.91224003 | 8.155412356 | 0.635749198 |
| RSC96-PD168368 | 31.3133227 | 8.075476011 | 0.455496105 |
| RSC96-PD168368 | 32.759 | 9.504295349 | 0.450255535 |
|  |  |  |  |
| RSC96-NMB+ PD168368 | 30.49795151 | 8.512650172 | 1.085271482 |
| RSC96-NMB+ PD168368 | 30.51572482 | 7.981579622 | 0.741856534 |
| RSC96-NMB+ PD168368 | 32.122 | 9.616583824 | 0.756584581 |

|  | *18s* Ct value | *GDNF* Ct value | 2^-∆∆Ct^ |
| --- | --- | --- | --- |
| RSC96-Ctrl | 22.25418091 | 7.617802461 | 1 |
| RSC96-Ctrl | 22.7268486 | 8.049489975 | 0.971994341 |
| RSC96-Ctrl | 23.119 | 8.506700516 | 1.01667745 |
|  |  |  |  |
| RSC96-NMB | 22.30514717 | 8.512650172 | 1.794872604 |
| RSC96-NMB | 21.41599846 | 7.981579622 | 2.30051938 |
| RSC96-NMB | 23.716 | 10.14922047 | 2.099222219 |
|  |  |  |  |
| RSC96-PD168368 | 23.29759661 | 8.822463671 | 1.118252129 |
| RSC96-PD168368 | 23.37059466 | 8.256453832 | 0.718090519 |
| RSC96-PD168368 | 24.301 | 9.504295349 | 0.895121313 |
|  |  |  |  |
| RSC96-NMB+ PD168368 | 23.58030574 | 9.26765728 | 1.2515622 |
| RSC96-NMB+ PD168368 | 23.66135852 | 8.709063848 | 0.803340647 |
| RSC96-NMB+ PD168368 | 24.543 | 10.22934055 | 1.25084208 |

|  | *18s* Ct value | *NGFR* Ct value | 2^-∆∆Ct^ |
| --- | --- | --- | --- |
| RSC96-Ctrl | 30.17326355 | 7.617802461 | 1.196640776 |
| RSC96-Ctrl | 30.86394119 | 8.049489975 | 1 |
| RSC96-Ctrl | 29.708 | 7.506700516 | 1.530054043 |
|  |  |  |  |
| RSC96-NMB | 29.86597443 | 8.822463671 | 3.412763551 |
| RSC96-NMB | 30.08065033 | 8.709063848 | 2.718601581 |
| RSC96-NMB | 29.472 | 8.616583824 | 3.887072368 |
|  |  |  |  |
| RSC96-PD168368 | 30.66639264 | 8.155412356 | 1.234109946 |
| RSC96-PD168368 | 31.86671543 | 8.256453832 | 0.576019524 |
| RSC96-PD168368 | 30.153 | 7.851142883 | 1.426927264 |
|  |  |  |  |
| RSC96-NMB+ PD168368 | 31.09254074 | 8.512650172 | 1.176548382 |
| RSC96-NMB+ PD168368 | 30.76585007 | 7.981579622 | 1.021140068 |
| RSC96-NMB+ PD168368 | 30.538 | 8.229340553 | 1.419940783 |

|  | *18s* Ct value | *MBP* Ct value | 2^-∆∆Ct^ |
| --- | --- | --- | --- |
| RSC96-Ctrl | 34.29468536 | 7.617802461 | 1 |
| RSC96-Ctrl | 35.17786598 | 8.049489975 | 0.731285619 |
| RSC96-Ctrl | 34.439 | 8.506700516 | 1.675371742 |
|  |  |  |  |
| RSC96-NMB | 31.64448357 | 8.822463671 | 14.46869609 |
| RSC96-NMB | 31.61540031 | 8.709063848 | 13.64732636 |
| RSC96-NMB | 32.714 | 10.14922047 | 17.29212829 |
|  |  |  |  |
| RSC96-PD168368 | 34.99231148 | 8.155412356 | 0.895015009 |
| RSC96-PD168368 | 34.42480087 | 7.981579622 | 1.17581546 |
| RSC96-PD168368 | 37.263 | 10.22934055 | 0.780760583 |
|  |  |  |  |
| RSC96-NMB+ PD168368 | 35.0583636 | 8.512650172 | 1.095181118 |
| RSC96-NMB+ PD168368 | 33.67985408 | 8.256453832 | 2.384162647 |
| RSC96-NMB+ PD168368 | 35.498 | 9.851142883 | 2.041648874 |

|  | *18s* Ct value | *NES* Ct value | 2^-∆∆Ct^ |
| --- | --- | --- | --- |
| RSC96-Ctrl | 32.60548401 | 7.617802461 | 1 |
| RSC96-Ctrl | 32.97322464 | 8.049489975 | 1.045321613 |
| RSC96-Ctrl | 33.05690765 | 8.049489975 | 1.823265748 |
|  |  |  |  |
| RSC96-NMB | 30.73504829 | 9.26765728 | 11.47395241 |
| RSC96-NMB | 30.42084122 | 8.96778965 | 11.58856465 |
| RSC96-NMB | 30.98613548 | 8.709063848 | 10.8230794 |
|  |  |  |  |
| RSC96-PD168368 | 33.67660395 | 8.512650172 | 0.884986754 |
| RSC96-PD168368 | 34.0052007 | 8.256453832 | 0.590060454 |
| RSC96-PD168368 | 34.10381317 | 8.256453832 | 0.825090879 |
|  |  |  |  |
| RSC96-NMB+ PD168368 | 33.59302521 | 9.623436292 | 2.025239647 |
| RSC96-NMB+ PD168368 | 32.92346954 | 7.981579622 | 1.032249433 |
| RSC96-NMB+ PD168368 | 32.58732605 | 7.981579622 | 1.303088546 |

|  | *18s* Ct value | *GFAP* Ct value | 2^-∆∆Ct^ |
| --- | --- | --- | --- |
| RSC96-Ctrl | 26.47989273 | 7.617802461 | 1 |
| RSC96-Ctrl | 26.85465431 | 8.049489975 | 1.040246859 |
| RSC96-Ctrl | 27.116 | 8.506700516 | 1.191371801 |
|  |  |  |  |
| RSC96-NMB | 27.36542892 | 9.26765728 | 1.698567587 |
| RSC96-NMB | 26.65577602 | 8.709063848 | 1.886063296 |
| RSC96-NMB | 28.129 | 10.14922047 | 1.843942394 |
|  |  |  |  |
| RSC96-PD168368 | 28.11556117 | 8.512650172 | 0.598398836 |
| RSC96-PD168368 | 27.37900734 | 7.981579622 | 0.689997267 |
| RSC96-PD168368 | 29.547 | 9.851142883 | 0.561184219 |
|  |  |  |  |
| RSC96-NMB+ PD168368 | 27.26989619 | 8.155412356 | 0.839502448 |
| RSC96-NMB+ PD168368 | 28.03377533 | 8.256453832 | 0.530258877 |
| RSC96-NMB+ PD168368 | 28.918 | 10.22934055 | 1.12745893 |

Figure S10j

|  | High (%) | Medium (%) | Low (%) |
| --- | --- | --- | --- |
| siNC RSC96 | 100 | 0 | 0 |
| siNC RSC96 | 95 | 5 | 0 |
| siNC RSC96 | 85.71 | 9.52 | 4.76 |
| siNC RSC96 | 78.13 | 15.63 | 6.25 |
| siNC RSC96 | 87.5 | 12.5 | 0 |
| siNC RSC96 | 93.55 | 3.23 | 3.23 |
|  |  |  |  |
| siNMBR RSC96 | 81.25 | 18.75 | 0 |
| siNMBR RSC96 | 87.5 | 8.33 | 4.17 |
| siNMBR RSC96 | 71.43 | 7.14 | 21.43 |
| siNMBR RSC96 | 96.55 | 3.45 | 0 |
| siNMBR RSC96 | 86.67 | 6.67 | 6.67 |
| siNMBR RSC96 | 87.5 | 6.25 | 6.25 |
|  |  |  |  |
| siNC RSC96+NMB | 84.21 | 5.26 | 10.53 |
| siNC RSC96+NMB | 85.71 | 8.57 | 5.71 |
| siNC RSC96+NMB | 25 | 25 | 50 |
| siNC RSC96+NMB | 78.57 | 10.71 | 10.71 |
| siNC RSC96+NMB | 80 | 10 | 10 |
| siNC RSC96+NMB | 50 | 27.78 | 22.22 |
|  |  |  |  |
| siNMBR RSC96+NMB | 95.45 | 4.55 | 0 |
| siNMBR RSC96+NMB | 91.67 | 4.17 | 4.17 |
| siNMBR RSC96+NMB | 93.75 | 6.25 | 0 |
| siNMBR RSC96+NMB | 93.33 | 6.67 | 0 |
| siNMBR RSC96+NMB | 86.67 | 13.33 | 0 |
| siNMBR RSC96+NMB | 92.31 | 7.69 | 0 |

Figure S10k

|  | Filopodia (%) | Lamellipodia (%) |
| --- | --- | --- |
| siNC RSC96 | 100 | 0 |
| siNC RSC96 | 100 | 0 |
| siNC RSC96 | 100 | 0 |
| siNC RSC96 | 94.12 | 5.88 |
| siNC RSC96 | 100 | 0 |
| siNC RSC96 | 95.45 | 4.55 |
|  |  |  |
| siNMBR RSC96 | 100 | 0 |
| siNMBR RSC96 | 100 | 0 |
| siNMBR RSC96 | 91.67 | 8.33 |
| siNMBR RSC96 | 100 | 0 |
| siNMBR RSC96 | 100 | 0 |
| siNMBR RSC96 | 100 | 0 |
|  |  |  |
| siNC RSC96+NMB | 86.67 | 13.33 |
| siNC RSC96+NMB | 83.33 | 16.67 |
| siNC RSC96+NMB | 57.14 | 42.86 |
| siNC RSC96+NMB | 82.35 | 17.65 |
| siNC RSC96+NMB | 78.95 | 21.05 |
| siNC RSC96+NMB | 76.47 | 23.53 |
|  |  |  |
| siNMBR RSC96+NMB | 100 | 0 |
| siNMBR RSC96+NMB | 92.86 | 7.14 |
| siNMBR RSC96+NMB | 91.67 | 8.33 |
| siNMBR RSC96+NMB | 92.31 | 7.69 |
| siNMBR RSC96+NMB | 93.33 | 6.67 |
| siNMBR RSC96+NMB | 94.44 | 5.56 |

Figure S10l

|  | EdU positive cells (%) |
| --- | --- |
| siNC RSC96 | 3.72 |
| siNC RSC96 | 19.95 |
| siNC RSC96 | 11.82 |
|  |  |
| siNMBR RSC96 | 51.63 |
| siNMBR RSC96 | 57.75 |
| siNMBR RSC96 | 45.24 |
|  |  |
| siNC RSC96+NMB | 5.03 |
| siNC RSC96+NMB | 14.77 |
| siNC RSC96+NMB | 15.66 |
|  |  |
| siNMBR RSC96+NMB | 14.84 |
| siNMBR RSC96+NMB | 22.41 |
| siNMBR RSC96+NMB | 13.38 |

Figure S10m

|  | Number of migration cells/HPF | Number of invasion cells/HPF |
| --- | --- | --- |
| siNC RSC96 | 339 | 64.33333333 |
| siNC RSC96 | 344.5 | 63.25 |
| siNC RSC96 | 377.3333333 | 74.2 |
|  |  |  |
| siNMBR RSC96 | 681 | 519.125 |
| siNMBR RSC96 | 590.5 | 521.5454545 |
| siNMBR RSC96 | 643.5 | 561.5 |
|  |  |  |
| siNC RSC96+NMB | 304.5 | 60.5 |
| siNC RSC96+NMB | 306 | 66.4 |
| siNC RSC96+NMB | 251 | 65.8 |
|  |  |  |
| siNMBR RSC96+NMB | 278 | 56.75 |
| siNMBR RSC96+NMB | 289 | 52.5 |
| siNMBR RSC96+NMB | 298 | 66.71428571 |

Figure 6a

| time | RSC96+Ca^2+^ | | | RSC96+NMB | | | RSC96+NMB+Ca^2+^ | | | RSC96+PD168368+NMB+Ca^2+^ | | |
| --- | --- | --- | --- | --- | --- | --- | --- | --- | --- | --- | --- | --- |
| 0 | 0 | 0 | 0 | 0 | 0 | 0 | 0 | 0 | 0 | 0 | 0 | 0 |
| 1 | 2.702801224 | -4.118553276 | -1.489400481 | 1.142610411 | 1.543786382 | 1.57546594 | -3.686034135 | -8.736601113 | 0.742572811 | 0.398051519 | 4.238703819 | 0.252049451 |
| 2 | 0.46575536 | -1.145360225 | 7.905115221 | 4.119589417 | 7.629355234 | -2.168288697 | -5.977879858 | -5.054376735 | 1.301342139 | -0.808213917 | -4.861060472 | 3.141242973 |
| 3 | 1.141020239 | -1.910261991 | 5.592282396 | -0.112029526 | 8.264484458 | 1.941467908 | -7.7933661 | -3.72536431 | 2.215303982 | 2.406999112 | 0.270979344 | 1.110149014 |
| 4 | -0.230838133 | -1.040638067 | -0.419673057 | -0.052045945 | 4.456116525 | -0.987721532 | -2.354947474 | -7.911223558 | 2.207678449 | 1.611017622 | -1.06202396 | -6.013333269 |
| 5 | 0.054277101 | -3.905224516 | 0.320844671 | 4.795044764 | 4.228629395 | -2.658045364 | -5.615864502 | -11.89693569 | -0.675511774 | 1.066908898 | 4.076282642 | -4.71557132 |
| 6 | 2.974022389 | -4.049629951 | 5.689140955 | 0.378062673 | 5.403468739 | -1.626229342 | -10.14054845 | -7.112531691 | -2.399181821 | 0.541608779 | 7.203878345 | -5.67776461 |
| 7 | -4.7209724 | -2.815653327 | 9.317725571 | 1.136394842 | 2.068464736 | -1.572207723 | -15.92441493 | -6.028746166 | -1.119401877 | -0.728928178 | 3.472234557 | -0.190490775 |
| 8 | -11.63184285 | 17.98106588 | 50.62035671 | -0.035783794 | 4.108825924 | -0.366002516 | -22.1915449 | -6.481082476 | -3.943796361 | -1.847077309 | 4.105679274 | -10.15642135 |
| 9 | -36.74187581 | -31.92549166 | 61.58892219 | -0.400389749 | -1.76825548 | 3.111781754 | -25.95371908 | -4.611425599 | -17.93898462 | -9.010055031 | 1.070709245 | -6.262163523 |
| 10 | -58.25648207 | 93.26250092 | 45.98735781 | 1.436759605 | 1.684046406 | -1.024191361 | -12.02797839 | 7.258640798 | -34.03540095 | -1.223074865 | 1.109893087 | 3.826898467 |
| 11 | 15.63470163 | 83.74166952 | 27.92479197 | 2.05506084 | 2.028078412 | -0.511555433 | 0.021945532 | 24.52872877 | -16.94305794 | 9.582454887 | -4.586084474 | 4.808512972 |
| 12 | 17.85326986 | 74.22401533 | 11.73217682 | 3.757397563 | 5.675482255 | 1.900703125 | 19.84287391 | 56.35841485 | 59.72110775 | 7.666599458 | -1.220647269 | -13.07602855 |
| 13 | 26.30701736 | 63.72531545 | 1.120165133 | 0.148219545 | -4.269445237 | 2.860874714 | 39.92111227 | 97.1784114 | 113.5797532 | 8.059265443 | 0.334669136 | -13.15889026 |
| 14 | 36.69594736 | 59.65902027 | -8.252329091 | 1.41975049 | 2.220388711 | -5.071035491 | 58.2208837 | 151.1336841 | 166.5307762 | 7.970644212 | -6.891473034 | -11.50839246 |
| 15 | 43.61189155 | 58.87583954 | -16.9616549 | 1.522728044 | 3.378878515 | -9.439100155 | 88.29636902 | 211.2416727 | 230.2264159 | 8.960941148 | -5.743883193 | -10.87395715 |
| 16 | 48.67145117 | 55.05746789 | -23.01455288 | 2.268445212 | 2.59089961 | -7.621043194 | 105.407088 | 270.3311242 | 313.6262537 | 7.584807963 | -5.039851376 | -12.94656951 |
| 17 | 58.68653957 | 55.88873807 | -29.33987203 | 2.206108397 | -4.853719007 | -12.59503714 | 149.2674408 | 319.3825096 | 397.6129468 | 8.776172682 | -4.337537401 | -15.50509025 |
| 18 | 72.91478831 | 55.44040425 | -34.92288507 | 1.36406613 | -8.57294258 | -10.66340345 | 203.7277052 | 375.8527187 | 471.8373598 | 9.176413193 | -5.921758511 | -14.95370729 |
| 19 | 76.7467566 | 56.58379982 | -38.03067269 | 0.637767811 | -2.946417347 | -12.83528416 | 261.1762483 | 414.4920532 | 507.1710832 | 7.152536072 | -1.488069339 | -17.21801824 |
| 20 | 83.68300184 | 57.91014124 | -37.96333379 | 5.989933994 | -4.430375335 | -12.86432042 | 322.9057745 | 437.3970642 | 534.1792466 | 9.972019875 | -2.843721965 | -14.67617497 |
| 21 | 85.98445276 | 56.31824073 | -39.43946764 | 1.793598945 | -0.856056013 | -12.61659133 | 370.1204615 | 458.4600761 | 551.8900211 | 9.391623991 | -5.260097255 | -14.16551168 |
| 22 | 90.53666927 | 53.3879938 | -36.5858238 | 4.397877302 | -6.020701805 | -13.0857479 | 411.4739787 | 457.1891969 | 566.9954132 | 8.245451713 | -2.769481483 | -15.94783294 |
| 23 | 88.46323944 | 56.16379352 | -37.05600205 | 1.410601173 | -4.419479425 | -16.09840488 | 457.6640602 | 460.3680655 | 577.9500388 | 8.84164722 | -4.1829613 | -16.18581523 |
| 24 | 90.89566071 | 57.64485157 | -40.55461257 | 3.0655301 | -6.004270859 | -17.43684578 | 487.1715015 | 460.928053 | 575.3026101 | 9.553236497 | -11.46671528 | -14.55583054 |
| 25 | 89.41051349 | 57.57020903 | -39.63265838 | 4.223433385 | -9.856649309 | -16.26343279 | 515.3544288 | 452.1559246 | 576.3928219 | 9.898864671 | -2.355096302 | -13.60857784 |
| 26 | 93.70719496 | 57.03152039 | -36.72681604 | 0.370145431 | -14.99811529 | -17.88170723 | 525.2724954 | 458.921983 | 578.0577588 | 10.10371546 | -3.52209148 | -13.5792075 |
| 27 | 89.80480553 | 61.38632501 | -36.1633938 | 1.215600436 | -6.955534907 | -16.49608481 | 537.7211482 | 443.6923974 | 582.0980559 | 8.455905418 | -1.895191969 | -10.83070953 |
| 28 | 95.08906736 | 60.44312787 | -38.35478505 | 4.034647415 | -11.29889462 | -21.60134685 | 553.7742056 | 432.8574107 | 568.4183126 | 10.89108524 | -3.958599069 | -8.168991246 |
| 29 | 95.53136972 | 62.58599504 | -36.01831512 | 6.970442825 | -13.91523019 | -18.63107754 | 563.068448 | 426.5510052 | 571.2085991 | 10.06741896 | -10.3087906 | -13.6512851 |
| 30 | 96.65135367 | 61.2435159 | -35.42613493 | 2.561151064 | -13.22673798 | -18.74419553 | 580.3142743 | 416.5893381 | 567.6196362 | 9.465358469 | -9.389596771 | -7.835947791 |
| 31 | 97.03465458 | 63.62419816 | -37.15918508 | -1.143182444 | -10.61085169 | -20.10425972 | 573.9139937 | 406.9467773 | 563.7737376 | 8.421423332 | -11.14132346 | -13.09617509 |
| 32 | 95.06359421 | 63.35440131 | -38.35329376 | 2.764667354 | -11.68846971 | -20.15293938 | 592.2524468 | 396.4377005 | 553.4138226 | 8.145516484 | -12.80791425 | -7.71382118 |
| 33 | 96.08466139 | 66.86567486 | -37.08574549 | 0.377154014 | -7.429067029 | -17.93813671 | 581.4424662 | 382.6026422 | 553.1083528 | 8.86650339 | -7.427056457 | -8.01923018 |
| 34 | 94.31410832 | 65.92093453 | -36.31770794 | 2.933256186 | -15.84605619 | -20.32321481 | 593.0303585 | 376.5091187 | 543.0385448 | 8.387677723 | -12.70032377 | -5.527039061 |
| 35 | 94.41085742 | 67.05420071 | -39.26422408 | 2.030283439 | -13.23926344 | -20.65480486 | 596.2975073 | 367.4697136 | 538.171849 | 8.191044481 | -10.60603548 | -14.72631986 |
| 36 | 96.47196876 | 68.31146069 | -41.02818663 | -0.245227799 | -12.62774142 | -18.98231102 | 588.8137727 | 350.0791944 | 536.3424437 | 8.703034817 | -12.89363473 | -6.13673752 |
| 37 | 93.84106066 | 66.91913247 | -40.05136697 | 2.310848738 | -13.03012933 | -21.84980683 | 600.0554129 | 339.6629503 | 530.0714119 | 9.216969487 | -11.87749407 | -6.829277696 |
| 38 | 93.39298726 | 70.40084793 | -41.02823705 | 3.593116622 | -6.635973825 | -17.6728575 | 586.8112876 | 333.4283512 | 521.1795777 | 9.772647336 | -15.49413477 | -9.696788917 |
| 39 | 89.34649662 | 70.00346598 | -38.8614763 | 0.914253083 | -9.230703258 | -17.87404252 | 586.3623611 | 325.092138 | 522.2022072 | 11.52279273 | -15.65765598 | -8.182039412 |
| 40 | 91.00562882 | 67.15902796 | -40.91270795 | -0.118701956 | -6.530352543 | -19.79461732 | 572.7300025 | 313.1822963 | 524.2118541 | 7.671371889 | -13.81875402 | -11.05702871 |
| 41 | 88.80187565 | 68.76423234 | -39.41167315 | 3.032410075 | -10.53497062 | -19.740734 | 567.5145683 | 303.4656136 | 510.6548135 | 7.504120382 | -13.22968648 | -7.739324113 |
| 42 | 82.64085667 | 67.43825201 | -42.39636263 | 2.900972964 | -7.913881411 | -20.71221566 | 569.6977645 | 298.8063199 | 501.766413 | 9.733707734 | -18.04112738 | -9.503934745 |
| 43 | 85.98379776 | 67.98782067 | -41.48839913 | 3.017072952 | -6.199974758 | -19.19839466 | 564.9650702 | 292.0992339 | 496.9600819 | 7.925392203 | -18.1191773 | -5.752107257 |
| 44 | 82.72533766 | 70.44260221 | -42.18886648 | 2.583323209 | -11.25605789 | -18.34364656 | 562.699965 | 281.6117539 | 487.3565517 | 9.381215544 | -17.1822073 | -6.598671096 |
| 45 | 83.62660708 | 66.0517591 | -42.64815033 | 0.080477382 | -6.248095061 | -19.76281341 | 552.2181582 | 273.321873 | 491.365477 | 8.422943713 | -18.40354526 | -9.752943258 |
| 46 | 76.44886398 | 66.57912983 | -42.32952298 | -0.046394474 | -7.621563135 | -22.39931158 | 550.4476663 | 263.2011106 | 483.5507574 | 9.612095751 | -20.2457956 | -7.585327501 |
| 47 | 69.57981988 | 68.63290046 | -44.96094821 | -0.422449403 | -10.85977699 | -21.85329545 | 536.0453662 | 262.9701199 | 487.3586678 | 8.887156344 | -17.41327739 | -9.423191598 |
| 48 | 71.77745216 | 68.28086564 | -42.58948736 | 0.264476071 | -10.07467298 | -24.41253264 | 546.2907425 | 253.1724666 | 482.2659445 | 9.86254967 | -20.70518452 | -12.75677281 |
| 49 | 69.82497283 | 73.73639973 | -42.34865254 | 2.484750389 | -0.655856879 | -22.07624905 | 541.4328648 | 246.2543377 | 482.8461711 | 8.71247038 | -19.42512865 | -12.33409593 |
| 50 | 64.61556574 | 70.25279298 | -42.8929546 | 3.155851217 | -6.008302385 | -21.78250377 | 539.6959925 | 243.2091427 | 479.0737051 | 7.330471622 | -23.1525056 | -12.93016392 |
| 51 | 62.07071668 | 69.32170252 | -43.20613743 | 3.035053774 | -4.985701061 | -21.48461243 | 529.532879 | 233.7697429 | 481.8526822 | 7.824485982 | -18.24428623 | -5.694896001 |
| 52 | 59.57355325 | 72.80717358 | -41.33482883 | 1.743605686 | -11.03130208 | -20.12577582 | 530.989396 | 225.5191852 | 478.3292701 | 8.85679236 | -24.25767275 | -14.7791231 |
| 53 | 55.38610198 | 69.05727376 | -44.88820721 | -0.08915524 | -9.012859128 | -21.70315917 | 526.6668756 | 218.8707968 | 468.5859497 | 7.38867178 | -24.62517379 | -15.15383861 |
| 54 | 53.23280075 | 72.51600594 | -44.77144702 | 0.931916683 | -7.189145007 | -22.82771729 | 533.8964061 | 212.1839346 | 470.0606371 | 8.534304246 | -22.07166042 | -16.42195969 |
| 55 | 47.97896207 | 72.14336572 | -44.67404631 | -1.133470054 | -6.931150647 | -23.35453895 | 522.7826478 | 201.43166 | 467.4519445 | 8.537749043 | -25.48600556 | -9.882965415 |
| 56 | 45.79774001 | 71.75508958 | -44.5162502 | 0.632569543 | -10.53181655 | -22.89680628 | 517.8473077 | 199.4594596 | 466.9059655 | 8.499519783 | -26.56932844 | -9.591831485 |
| 57 | 46.43429436 | 72.00705967 | -43.06741134 | 1.231332723 | -10.2845554 | -25.82905841 | 504.2393803 | 193.9557234 | 464.9707193 | 7.785418834 | -22.37162246 | -11.58127971 |
| 58 | 42.54839944 | 69.75164238 | -45.31979685 | -1.565592422 | -13.77602361 | -24.6057635 | 510.5526135 | 193.2290991 | 468.9560966 | 9.000989997 | -26.14747043 | -17.2676852 |
| 59 | 30.00656525 | 71.4730478 | -45.36072347 | 0.98955774 | -11.6217718 | -25.4151018 | 520.3912391 | 187.4773924 | 459.7147786 | 8.054027141 | -27.35538534 | -9.126403698 |
| 60 | 29.30726494 | 69.30184856 | -43.37131806 | 3.170176701 | -11.62711722 | -24.52794518 | 506.1365394 | 178.565156 | 463.913644 | 8.324787069 | -24.71666421 | -14.62290659 |
| 61 | 28.02916176 | 73.90801124 | -41.73290652 | 1.981286654 | -8.896788045 | -27.17204934 | 517.3418295 | 171.8207957 | 467.3796781 | 7.44496561 | -26.61509749 | -10.64613448 |
| 62 | 27.57478815 | 69.01524366 | -43.22212616 | -0.37805671 | -11.64256355 | -27.40147153 | 502.1532178 | 171.3373916 | 461.9046627 | 9.433153965 | -29.14623773 | -11.82301901 |
| 63 | 22.0718761 | 69.87775351 | -42.54235997 | 2.441762373 | -8.905432922 | -24.36591472 | 522.6337837 | 166.0147084 | 459.2535292 | 9.060763823 | -28.63633461 | -12.40076911 |
| 64 | 20.68619513 | 72.50996137 | -43.53842492 | 0.107764242 | -9.04857496 | -25.8959547 | 511.912551 | 161.5511202 | 457.3053467 | 7.900222306 | -27.37823605 | -15.46219425 |
| 65 | 18.46230852 | 68.84004906 | -46.98032464 | 4.599336783 | -11.77463982 | -24.97178854 | 511.8162958 | 161.9964282 | 460.5162128 | 9.940785369 | -31.0737434 | -9.655716415 |
| 66 | 17.92282788 | 71.75628769 | -43.64450757 | 0.978301179 | -12.61090218 | -27.87390743 | 497.2307424 | 158.079067 | 457.7954697 | 9.111521268 | -33.50795149 | -12.95296509 |
| 67 | 15.89982566 | 67.4883258 | -43.13348401 | -0.55786036 | -12.10243259 | -23.40724556 | 504.5554066 | 155.0550778 | 452.2894482 | 9.298394033 | -29.66677287 | -11.99206624 |
| 68 | 12.57239694 | 70.82638287 | -43.22452873 | 2.330615666 | -8.776416197 | -27.7324087 | 515.9391168 | 151.7549376 | 451.3409795 | 7.5008143 | -29.56895632 | -13.16316281 |
| 69 | 16.46773481 | 65.74004097 | -43.93204673 | -2.15591343 | -7.625562157 | -24.91948512 | 490.319797 | 154.8454599 | 449.7184312 | 8.283162789 | -32.16558776 | -11.95526219 |
| 70 | 13.80780846 | 67.03037126 | -44.13253669 | 0.127186768 | -6.460257112 | -26.15395684 | 497.3618978 | 147.3885444 | 455.1518173 | 9.346070638 | -31.35160506 | -8.276875185 |
| 71 | 8.745871699 | 64.13026181 | -44.81272698 | 0.501180778 | -10.15260417 | -27.05519784 | 491.4339204 | 139.5864115 | 453.5711108 | 8.39488429 | -32.24914676 | -8.893874459 |
| 72 | 10.66122469 | 67.25099028 | -44.10715848 | -0.677499537 | -9.496833769 | -26.80297166 | 489.2258547 | 141.5894591 | 453.946089 | 7.288810614 | -35.18390703 | -8.590136006 |
| 73 | 9.967799852 | 68.51728707 | -44.02846324 | 0.462273341 | -12.1660455 | -29.23047386 | 482.2410197 | 142.494569 | 450.9234899 | 7.026785553 | -32.60547606 | -11.30584509 |
| 74 | 12.87019938 | 61.87012378 | -43.54566433 | -0.914040141 | -10.63587356 | -26.75640264 | 479.6505296 | 136.0067843 | 446.8577022 | 8.936858345 | -36.01965789 | -12.3771705 |
| 75 | 10.03835531 | 63.50217644 | -43.50245997 | -0.218347524 | -12.68285773 | -22.84771996 | 480.2837993 | 135.6604257 | 447.1900458 | 9.329167993 | -35.07291789 | -10.34636956 |
| 76 | 10.56666888 | 66.54931711 | -43.65569887 | -0.362791049 | -10.36999815 | -26.51498904 | 486.367285 | 137.2551786 | 446.0305757 | 6.80022323 | -37.17702802 | -9.133073108 |
| 77 | 10.24241585 | 67.30608752 | -42.56679458 | 2.647690968 | -13.84097997 | -23.70928738 | 480.4524266 | 136.8618009 | 448.8083615 | 6.725347326 | -35.04022658 | -8.526994823 |
| 78 | 8.487017311 | 63.59105891 | -44.45251848 | -1.379866586 | -9.508855194 | -24.91279068 | 478.6785847 | 134.9089727 | 443.661505 | 8.171189013 | -37.0829429 | -9.321770501 |
| 79 | 8.232403996 | 62.71333538 | -43.85360201 | 0.454920654 | -11.81252965 | -25.72377167 | 481.6973087 | 133.1234622 | 447.502057 | 6.973283497 | -40.8869311 | -9.708534753 |
| 80 | 4.945837822 | 67.20460786 | -46.00754116 | 0.900514915 | -12.25000112 | -24.38081198 | 481.7907713 | 133.1469028 | 442.0693575 | 6.060556751 | -37.00741326 | -13.01459961 |
| 81 | 9.014218247 | 65.14001783 | -45.81424209 | -1.129249858 | -13.88985149 | -29.87055545 | 486.6394502 | 128.9716441 | 433.8339781 | 7.82896036 | -34.25477633 | -11.18930578 |
| 82 | 2.436713266 | 67.16634086 | -43.87952155 | 1.679245943 | -12.35315412 | -26.2193659 | 473.0232122 | 127.9118698 | 436.6950806 | 6.84014659 | -40.51024423 | -9.651780756 |
| 83 | 7.998915384 | 61.7388224 | -45.02089766 | 1.015449633 | -8.243676868 | -25.92256378 | 486.877492 | 123.7580017 | 441.3716034 | 7.087361889 | -39.93478098 | -8.158358681 |
| 84 | 5.897704222 | 62.81670182 | -42.81068305 | -0.793324513 | -11.2956161 | -25.90104339 | 480.4142349 | 135.2472503 | 436.9274105 | 9.103559483 | -38.902458 | -4.667013384 |
| 85 | 5.616413892 | 66.08616528 | -44.74098084 | 2.925450505 | -13.23609662 | -25.61666775 | 478.8936792 | 128.2480912 | 436.4590634 | 7.422182713 | -37.33420001 | -12.76068691 |
| 86 | 3.175560573 | 66.90428563 | -45.44636425 | -2.544598409 | -9.199796613 | -24.81939323 | 483.3180413 | 125.1959819 | 431.1442093 | 7.364115284 | -41.06975502 | -8.589953533 |
| 87 | 3.572840556 | 64.68396055 | -46.01166443 | -4.360121044 | -8.039037537 | -30.80871317 | 482.0146372 | 123.1878766 | 435.699981 | 9.817665325 | -39.80307062 | -8.65159264 |
| 88 | 3.177423107 | 65.34519215 | -44.62663743 | -2.076689128 | -8.171059832 | -25.97274266 | 484.5835343 | 123.245405 | 431.6040118 | 7.346727673 | -42.75405934 | -5.334252085 |
| 89 | 4.612087568 | 64.09349248 | -44.24222868 | -1.063463344 | -14.08514293 | -26.64123481 | 486.8252926 | 121.7679332 | 432.0224723 | 8.721418141 | -39.76869701 | -7.13035419 |
| 90 | 3.358488335 | 63.80561866 | -45.78158402 | 2.503920325 | -11.46658787 | -27.38662767 | 486.2898758 | 114.89597 | 432.2586809 | 8.697432926 | -41.95650837 | -5.162512747 |
| 91 | 1.330703894 | 64.21772826 | -44.80765577 | -0.909615551 | -13.00556706 | -25.68881014 | 471.0183421 | 112.7868452 | 435.7415695 | 6.587893884 | -42.49606921 | -8.408323802 |
| 92 | 2.928122391 | 60.42297876 | -46.72150382 | -2.907720803 | -11.18544238 | -26.35824475 | 466.3317046 | 109.8121699 | 433.6226039 | 9.585806194 | -39.32190227 | -8.194679813 |
| 93 | 1.294069674 | 64.69789555 | -44.94087003 | -3.079192511 | -8.79317548 | -26.66965961 | 472.371378 | 106.4415582 | 428.3366097 | 7.420036795 | -39.46048916 | -9.267620167 |
| 94 | 4.082442998 | 63.13376878 | -45.20580531 | -0.646395565 | -17.52704182 | -25.93134636 | 469.2032496 | 111.2303808 | 426.9197089 | 8.849880147 | -42.38531203 | -8.916124605 |
| 95 | 1.394944131 | 59.43702266 | -44.83420177 | -0.897661491 | -13.36475122 | -26.17358654 | 463.6914362 | 101.6923028 | 427.2507326 | 10.18819129 | -38.15486808 | -7.58519295 |
| 96 | 2.552845118 | 61.80410801 | -45.00872271 | -3.728717129 | -14.06462273 | -28.41870531 | 467.8127305 | 103.5035449 | 429.1979939 | 9.828662458 | -39.72392952 | -8.896023388 |
| 97 | 4.645615307 | 63.97124043 | -46.50161816 | -0.801408806 | -12.79110671 | -28.34983131 | 468.9451399 | 98.9660834 | 425.3477865 | 9.223657868 | -41.48546141 | -3.854015568 |
| 98 | 4.683286683 | 60.77708526 | -45.66800729 | -6.295059336 | -11.3771389 | -26.59383549 | 455.1415486 | 98.03278361 | 422.0472256 | 10.20842081 | -39.35661194 | -5.483094338 |
| 99 | 0.799168699 | 62.28722385 | -43.21096912 | 1.793866995 | -11.94809469 | -24.30019876 | 443.8825255 | 92.26753045 | 417.3464601 | 10.00574416 | -41.94495394 | -7.819789764 |
| 100 | 0.913801276 | 59.36873632 | -45.13371955 | -1.718723297 | -16.83579971 | -29.70385089 | 445.8975488 | 91.78256409 | 418.7164793 | 9.275997249 | -45.95489876 | -7.980768153 |
| 101 | 1.021053931 | 58.22616354 | -44.6713736 | -0.453556393 | -13.78051632 | -28.17521502 | 444.4098903 | 89.22240679 | 412.2462596 | 7.859072752 | -45.52751336 | -5.968283372 |
| 102 | -2.152497401 | 60.833003 | -46.56340604 | -3.037668722 | -14.12428333 | -27.68989206 | 437.1994112 | 88.4949946 | 414.8150514 | 7.616110256 | -40.03610242 | -6.356988492 |
| 103 | 0.30920773 | 61.27611127 | -45.23337681 | -1.917591435 | -13.77902225 | -29.28156218 | 437.1908925 | 86.35086872 | 408.3603069 | 7.947512325 | -41.74615423 | -2.496086527 |
| 104 | 1.758519882 | 58.9380131 | -46.66633059 | -2.342516029 | -15.81773762 | -30.66345518 | 435.5257821 | 81.21598737 | 400.633991 | 8.44447135 | -41.83818688 | -10.93026099 |
| 105 | 1.73135548 | 61.39269 | -46.75401371 | -4.404306994 | -17.36842138 | -29.00821697 | 437.3119025 | 83.30207792 | 397.4580426 | 8.68677486 | -36.69274202 | -5.402305614 |
| 106 | 0.751725748 | 62.99702416 | -47.88297656 | -3.797992485 | -14.12557239 | -27.29853974 | 443.1597779 | 80.04509991 | 396.4439044 | 8.943505986 | -41.39856422 | -6.365395986 |
| 107 | -1.111070968 | 57.76137198 | -46.3850149 | -6.085312007 | -20.43512675 | -27.23137299 | 436.5801986 | 80.68127149 | 395.7208568 | 8.259054888 | -40.17025049 | -6.723244212 |
| 108 | 0.330864499 | 58.90768307 | -47.81822854 | -3.868693443 | -10.62263397 | -26.97747913 | 439.9413609 | 80.09031283 | 391.8228322 | 8.343032959 | -44.64343802 | -8.339057778 |
| 109 | -2.770509355 | 57.68261897 | -44.92269004 | -1.456771674 | -13.17967711 | -27.17143223 | 440.9337139 | 86.22215687 | 385.3469277 | 8.778826788 | -41.37652189 | -7.763395737 |
| 110 | -2.661822371 | 61.07794039 | -47.51533468 | 1.05969837 | -16.1523153 | -27.78530208 | 432.2382977 | 81.5162648 | 389.8196693 | 8.029917127 | -44.28422058 | -7.75836662 |
| 111 | 0.7529816 | 61.79902861 | -48.62025126 | -5.475695894 | -15.13666228 | -27.18679698 | 441.8482572 | 84.49616032 | 385.1470728 | 8.050736634 | -42.87033723 | -6.877976432 |
| 112 | 3.122200201 | 57.15748704 | -47.18046815 | -0.054122379 | -12.62381789 | -27.33495186 | 441.2582516 | 82.10608376 | 383.4968253 | 8.19030922 | -45.82896564 | -7.437018726 |
| 113 | 0.082623574 | 60.89625731 | -47.51201338 | -3.779353579 | -7.631784894 | -27.58757426 | 433.9679281 | 76.12988978 | 388.616103 | 8.45550667 | -47.02040822 | -8.875572583 |
| 114 | -3.599171567 | 57.84493871 | -49.18217972 | -5.782030228 | -15.21771216 | -25.78224259 | 426.8332651 | 78.07121864 | 390.643871 | 7.699441144 | -43.65280467 | -3.677294834 |
| 115 | -1.030695571 | 57.66913446 | -46.64064351 | -6.383717568 | -13.752331 | -27.70433559 | 421.6897178 | 79.17502226 | 382.2806987 | 9.159493079 | -44.67211556 | -10.31834822 |
| 116 | -3.761211057 | 59.03454847 | -48.41377317 | -3.398690769 | -13.34313188 | -30.0339485 | 442.6228313 | 82.87590181 | 375.6986552 | 11.47366604 | -46.65149576 | -6.063547487 |
| 117 | -2.464608753 | 55.73689867 | -47.47869294 | -4.56711028 | -15.77861376 | -26.39528091 | 429.4554065 | 81.46012947 | 372.8489944 | 8.119447625 | -42.43478655 | -11.49285665 |
| 118 | -3.284688074 | 57.40883657 | -45.94600685 | -3.238053023 | -17.2280803 | -28.06563269 | 429.6705059 | 80.49024172 | 373.0595253 | 7.668262069 | -49.13441533 | -7.399501192 |
| 119 | -0.823372471 | 57.9452374 | -47.83183054 | -3.968577663 | -12.03510105 | -28.36352258 | 425.0683579 | 76.89827409 | 374.7120626 | 8.627310779 | -44.40333241 | -8.522810344 |
| 120 | -1.461645509 | 56.2516712 | -47.26727651 | -2.395314985 | -13.65469833 | -26.20797598 | 436.3052394 | 75.72158779 | 368.0659381 | 8.361100329 | -47.00386525 | -8.492310601 |

Figure 6b

| RSC96+Ca^2+^ | RSC96+NMB | RSC96+NMB+Ca^2+^ | RSC96+PD168368+NMB+Ca^2+^ |
| --- | --- | --- | --- |
| 93.26250092 | 6.970442825 | 600.0554129 | 11.52279273 |
| 61.58892219 | 8.264484458 | 460.928053 | 7.203878345 |
| 97.03465458 | 3.111781754 | 582.0980559 | 4.808512972 |

Figure 6c

| time | RSC96 baseline | | | RSC96+NMB+Ca^2+^ | | | RSC96+ NMB+PD168398+Ca^2+^ | | | RSC96+Nifedipine | | | RSC96+NP118809 | | | RSC96+Trimethadione | | | RSC96+NiCl_2_ | | |
| --- | --- | --- | --- | --- | --- | --- | --- | --- | --- | --- | --- | --- | --- | --- | --- | --- | --- | --- | --- | --- | --- |
| 0 | 0 | 0 | 0 | 0 | 0 | 0 | 0 | 0 | 0 | 0 | 0 | 0 | 0 | 0 | 0 | 0 | 0 | 0 | 0 | 0 | 0 |
| 2 | 1.052334032 | 0.17789359 | -0.28344246 | -0.432907532 | -2.273912935 | -5.495250571 | 2.648126679 | 0.348602688 | -0.387971437 | 0.304084482 | 2.669338075 | 2.166171115 | 2.201948585 | 0.134474885 | -0.080049414 | 0.295722602 | 0.240295468 | -0.093364414 | 0.876054377 | 1.18186391 | 1.659740532 |
| 4 | 1.894160487 | -1.92709264 | 4.280049603 | 4.325795714 | 2.55297654 | -1.717430371 | 1.667222047 | 0.338260971 | -0.26613152 | 1.102444076 | 4.279827751 | 2.232700223 | 3.385929729 | 1.171857178 | 0.55255388 | 0.354305505 | 0.487540338 | 0.598239771 | 1.154570265 | 0.599416599 | 3.231315287 |
| 6 | 3.457610719 | -0.2075645 | 1.681785714 | 10.97846941 | -2.951283941 | 7.326561914 | 2.103043579 | -0.072715834 | 0.536278595 | 2.131777357 | 2.673126455 | 4.649493144 | 4.773515949 | 2.875681107 | 1.861455814 | 1.071046224 | -0.30092788 | 0.38048882 | 0.809009469 | 0.982161158 | 4.411906355 |
| 8 | -0.931995453 | -2.964717332 | 1.79515873 | 22.55218667 | 1.675209712 | 16.77144155 | 1.687979023 | 0.578814996 | -1.266978493 | 2.79125988 | 8.029756838 | 2.703913697 | 9.078212707 | 4.270955939 | 2.36098782 | -0.279899035 | 1.6437879 | -0.129484931 | 2.954893778 | -0.216567852 | 4.412223637 |
| 10 | -0.691522144 | 0.681837513 | 0.311785714 | 16.87355493 | 0.239315673 | 19.97722381 | 3.149892999 | 0.400785785 | -4.382119123 | 3.782394702 | 8.282969438 | 2.404925064 | 14.75785279 | 5.640549784 | 1.806007351 | 0.29701136 | 1.815409602 | -0.123550944 | 5.375417274 | -0.974329495 | 6.02639282 |
| 12 | 1.29287529 | -4.743587292 | 2.267579365 | 16.0618533 | 0.478631346 | 24.84250854 | 4.201069906 | 0.360316651 | -1.320321452 | 2.419533422 | 9.528095174 | 2.017346812 | 15.86323818 | 8.643196284 | 2.418444026 | -0.288213628 | 1.781453559 | -0.02807677 | 6.096653799 | -0.730206831 | 4.124891231 |
| 14 | 0.721623776 | -1.92709264 | -0.151170635 | 11.68194415 | -0.478631346 | 22.32396174 | 4.893819696 | 0.317907809 | 0.756479476 | 0.446393879 | 6.306805267 | 3.178374419 | 20.33784137 | 10.55983799 | 3.939363062 | 2.594254061 | 1.114631993 | -0.322373162 | 7.597662897 | -1.979236987 | 6.425950295 |
| 16 | 0.631386829 | -3.172281831 | 1.341656746 | 33.31420232 | 3.549987165 | 25.24339589 | 4.124214936 | -0.430757464 | -0.829596338 | 1.018526937 | -4.210014287 | 3.885099695 | 23.95465195 | 11.94005458 | 3.078978612 | 2.347819883 | 1.80290969 | 0.107602037 | 9.200599418 | -1.71888134 | 6.22056173 |
| 18 | -1.503246967 | -1.89742173 | 1.785714286 | 55.54334815 | 3.589735097 | 33.14265402 | 3.627547356 | -19.59937239 | -1.502879873 | -3.539446525 | 5.772101772 | 4.898752469 | 27.9509426 | 10.88563322 | 0.44774562 | -2.995002785 | 0.888476281 | -6.575279556 | 10.02643847 | -4.366888253 | -0.247212086 |
| 20 | -2.916392887 | 1.89742173 | 0.614136905 | 291.2910155 | 1.714957644 | -5.609789814 | 2.258011887 | -6.027726355 | -1.934980462 | 42.36256079 | 39.40872393 | 5.002679003 | 16.46656586 | 14.69222475 | 7.245030032 | 7.195148218 | 1.797749011 | -8.344750443 | 11.61658058 | -10.89487596 | -6.689237507 |
| 22 | 1.533416549 | -2.964717332 | 1.048759921 | 384.6383418 | 33.54642641 | 19.74814532 | 5.733923908 | -4.360319699 | -3.395855035 | 57.03973838 | 45.56634314 | 11.74075901 | 12.00330028 | 9.600015913 | -0.255279873 | 8.063136742 | -8.3553357 | 0.577040095 | 12.32669747 | -6.138510541 | -15.28074264 |
| 24 | -3.698084028 | -1.571305461 | 4.37452381 | 389.8857059 | 188.8316592 | 35.54666158 | 7.694521969 | -2.533635925 | -3.776949243 | 59.56031206 | 50.74502381 | 31.07963566 | 84.88789865 | 41.84645249 | -3.071664207 | 9.118296741 | -18.15355034 | 2.105409533 | 11.61096274 | -8.108535779 | -15.90746401 |
| 26 | -3.878489972 | -2.164262114 | 2.825019841 | 385.0171359 | 418.4687109 | 49.68501708 | 5.749963857 | -1.87381295 | -4.047131698 | 61.75394889 | 53.41485668 | 39.88233005 | 147.8995705 | 127.944988 | 105.3096173 | 10.83732354 | -14.23458978 | 1.060790551 | 8.980809732 | -10.17789143 | -15.45956428 |
| 28 | -3.397475404 | -3.379780396 | 1.927430556 | 384.6924553 | 528.8793567 | 68.91839408 | 6.644311319 | -1.321301141 | -3.744397638 | 60.21479537 | 54.52882384 | 40.9448813 | 185.2767884 | 177.3915572 | 157.3273359 | 11.2862043 | -15.93393978 | -0.235195103 | 8.337059071 | -11.3681296 | -16.72392136 |
| 30 | -1.202638343 | -4.180301549 | 2.295922619 | 357.3815654 | 516.7935012 | 71.60874974 | 7.04984884 | -2.526927135 | -2.904216242 | 58.30956261 | 52.54233708 | 39.56563411 | 207.3789682 | 205.0603703 | 169.950171 | 11.1584992 | -18.90287638 | 0.222467164 | 7.056910093 | -12.45931666 | -17.67785539 |
| 32 | -3.006561884 | -5.306873036 | 3.892668651 | 341.9674335 | 508.2170568 | 70.97878391 | 7.145330759 | -3.479292704 | -5.028746052 | 57.93197294 | 54.86827694 | 37.40797634 | 212.4877524 | 208.7519967 | 168.4017757 | 12.00982029 | -18.35551233 | -0.105056045 | 7.207446965 | -11.53666597 | -18.6681637 |
| 34 | -3.968726918 | -3.735567575 | 1.97468254 | 342.9955889 | 487.3957652 | 99.31342281 | 6.572812982 | -4.346263265 | -4.101420409 | 52.62865501 | 49.90303561 | 36.53481316 | 211.7973392 | 209.6790652 | 172.9009874 | 11.17588414 | -17.79563755 | -1.389550254 | 7.709160902 | -12.55613815 | -19.98098757 |
| 36 | -3.036663516 | -5.425424805 | 4.062738095 | 337.4792975 | 460.31169 | 106.754524 | 6.123836931 | -4.22306486 | -5.468738114 | 51.45919317 | 44.03293547 | 34.74258754 | 202.9288588 | 208.3768628 | 173.9912023 | 13.16326564 | -15.03814781 | -1.975478298 | 6.072989506 | -11.65314136 | -19.6642701 |
| 38 | -2.946426569 | -4.773192267 | 1.379444444 | 323.5245888 | 441.205356 | 123.9841225 | 7.253298072 | -4.136814126 | -5.809299759 | 45.63439487 | 45.20827388 | 34.48367898 | 196.5989693 | 204.9377859 | 172.9579889 | 15.85724945 | -13.44802802 | -2.569807306 | 6.457499327 | -12.57993641 | -19.71701307 |
| 40 | -5.201398944 | -6.048052368 | 6.519275794 | 325.2021055 | 425.2101258 | 139.5535603 | 8.286178787 | -6.420762395 | -7.384567975 | 40.86753144 | 42.68712644 | 32.97835412 | 184.6121497 | 201.3100832 | 163.6367711 | 17.42300648 | -12.84043376 | -3.654536349 | 6.159736255 | -12.9084352 | -20.31277391 |
| 42 | -4.780519691 | -3.883790254 | 3.779295635 | 308.1662103 | 404.9461333 | 141.6139501 | 7.75317247 | -8.631694435 | -7.627791242 | 36.94692438 | 38.7414545 | 31.89422992 | 171.862704 | 196.2182995 | 152.2604758 | 20.09081392 | -12.30255239 | -4.315956957 | 7.186346769 | -13.93438389 | -21.3162985 |
| 44 | -4.960857686 | -7.737909599 | 4.780803571 | 299.8360199 | 380.893666 | 154.3218816 | 9.384798986 | -6.446435682 | -7.58119401 | 32.61320663 | 35.5841366 | 29.6579424 | 164.0275091 | 190.446527 | 140.620071 | 20.27488028 | -11.72448796 | -4.10126431 | 7.089814715 | -13.74847166 | -22.95862193 |
| 46 | -2.405276687 | -6.048052368 | 1.965228175 | 286.8028795 | 362.9839104 | 158.7290094 | 9.059103754 | -3.312870174 | -8.140000763 | 26.45950584 | 34.25982369 | 28.42577936 | 151.0468151 | 185.945105 | 129.9665978 | 20.3414422 | -9.826506187 | -4.691113655 | 8.224816241 | -14.6051231 | -24.20437392 |
| 48 | -2.946426569 | -6.818902612 | 4.383978175 | 259.9773707 | 352.2942009 | 162.9077169 | 9.392418634 | -2.598976949 | -7.832201907 | 23.9171662 | 30.84419053 | 26.44355298 | 141.9544839 | 175.3675061 | 116.9033617 | 21.89321539 | -9.452030464 | -5.962846377 | 9.341475368 | -13.97874775 | -23.97733577 |
| 50 | -1.80392354 | -5.781211984 | 4.903630952 | 244.5632389 | 336.9771698 | 170.0631282 | 10.53935834 | -1.697666253 | -8.168232831 | 17.52407512 | 27.50159668 | 26.02889646 | 132.2989393 | 167.6383195 | 101.8520088 | 22.45726919 | -7.282679436 | -6.145372932 | 9.304031245 | -15.81520249 | -24.49399668 |
| 52 | -3.72818566 | -6.403839548 | 4.204464286 | 223.201548 | 323.1357806 | 183.5149066 | 10.91493715 | -1.357650684 | -8.631167857 | 15.03479272 | 24.84760444 | 25.21748824 | 118.3242409 | 158.5888004 | 89.29943476 | 21.0547549 | -5.762104353 | -8.589479591 | 8.906626727 | -15.48362893 | -26.28735273 |
| 54 | -4.900790321 | -6.018381458 | 4.412321429 | 210.437335 | 302.1944171 | 184.5451015 | 12.52511315 | -1.349356715 | -9.140630858 | 7.941549274 | 23.63624022 | 24.80892876 | 107.2168329 | 150.3499489 | 74.84260026 | 22.24949252 | -3.705266906 | -9.775251564 | 7.10644148 | -16.32575489 | -25.00363071 |
| 56 | -3.577881348 | -5.662660215 | 4.383978175 | 191.8862634 | 292.0222588 | 193.3027457 | 12.59162279 | -0.603719993 | -9.860746038 | 6.025554943 | 21.6819979 | 23.94900035 | 95.23402998 | 140.9909451 | 64.66770486 | 21.20341514 | -4.461310516 | -12.60391428 | 7.52172072 | -17.32336127 | -25.14817746 |
| 58 | 0.180405944 | -2.608996088 | 3.051775794 | 180.8536805 | 276.5056599 | 205.8961379 | 13.27064875 | 0.950484687 | -9.497915408 | 0.416283332 | 17.18185942 | 25.25125546 | 85.94660879 | 135.0331507 | 55.09131521 | 21.31871132 | -4.71646604 | -14.21365835 | 7.338937727 | -17.83812148 | -24.67852039 |
| 60 | -4.088997548 | -5.069703561 | 3.524186508 | 174.6339144 | 277.6227424 | 209.0439923 | 13.38618954 | 0.215600431 | -9.586065854 | -3.927164246 | 15.18025469 | 25.60227617 | 75.75475281 | 128.4081669 | 47.60066811 | 20.33411443 | -3.288867664 | -12.73952546 | 9.018434949 | -18.31222432 | -25.27524769 |
| 62 | -4.23930186 | -3.290833601 | 0.547996032 | 158.4638342 | 260.1121223 | 236.5768565 | 13.64913121 | 0.484445548 | -10.26788883 | -7.072741575 | 12.66182515 | 24.31626557 | 69.65570675 | 124.6392885 | 38.96154265 | 19.90687402 | -3.729830514 | -12.22013775 | 9.045567623 | -18.83751647 | -25.30515314 |
| 64 | -2.976528201 | -6.403839548 | 2.966736111 | 150.0270567 | 258.6356522 | 256.4395411 | 14.38133245 | 0.380786412 | -10.16329734 | -8.755266869 | 9.181022667 | 23.24947622 | 61.67155626 | 117.7291374 | 35.405012 | 19.63544352 | -4.554192084 | -10.53002125 | 10.21633546 | -18.40154025 | -25.0820976 |
| 66 | -7.095559432 | -6.463115432 | 3.788740079 | 142.3462276 | 260.7099974 | 249.1702488 | 13.36133711 | 0.440260915 | -10.32904543 | -11.27208931 | 7.834414677 | 22.85873892 | 59.05325375 | 113.1617693 | 30.31188172 | 19.85706243 | -4.863643934 | -12.23548959 | 12.15227235 | -20.1211069 | -25.56670351 |
| 68 | -3.487644401 | -3.587344895 | 2.910049603 | 136.4511421 | 247.1079239 | 253.8637246 | 14.05304186 | -0.371440309 | -11.54837085 | -12.00536787 | 3.096182642 | 23.1742635 | 53.49446217 | 108.5727524 | 28.51720665 | 19.82146102 | -5.297780316 | -12.31912336 | 13.7701753 | -20.39917095 | -25.67783557 |
| 70 | -6.073327032 | -5.988776484 | 2.825019841 | 127.2567765 | 244.7950911 | 282.427442 | 13.59108996 | -0.780145814 | -11.76725734 | -13.15378319 | 1.592854931 | 21.90078774 | 51.6470489 | 105.6038745 | 25.70725043 | 19.53555578 | -6.563871349 | -13.45949287 | 15.73935732 | -21.35305754 | -25.80824994 |
| 72 | -8.117859781 | -5.751541074 | 2.437638889 | 121.9569388 | 240.2472653 | 280.8245509 | 13.78177204 | 0.148352434 | -11.80439958 | -13.67368478 | -1.186346197 | 22.97207652 | 49.99970032 | 103.8797247 | 25.1648224 | 20.05833798 | -7.93420963 | -14.42805588 | 15.26068313 | -22.46618992 | -25.96599815 |
| 74 | -3.668050345 | -3.498398101 | 2.749424603 | 140.1833298 | 230.4750706 | 282.8849407 | 12.07515353 | 0.581310164 | -11.40472025 | -16.34463535 | -5.41701479 | 20.54231652 | 48.48904646 | 99.97101101 | 24.71668911 | 19.8394724 | -7.366711003 | -17.59690641 | 14.63496269 | -22.87699391 | -26.9015129 |
| 76 | -3.487644401 | -2.638601063 | 1.729027778 | 124.769198 | 235.5006997 | 293.0163975 | 13.6099402 | 0.188231194 | -11.84429359 | -17.38380524 | -5.14623395 | 20.89507605 | 48.05528788 | 99.97505278 | 24.12873508 | 19.37288715 | -5.844548591 | -18.20464318 | 15.48934622 | -22.63533815 | -26.49809665 |
| 78 | -4.419707804 | -0.711574358 | 2.56047619 | 117.5770297 | 224.4118548 | 295.2492545 | 12.96556634 | 1.167226589 | -12.63521325 | -18.29371017 | -5.390583209 | 21.50287417 | 47.00007377 | 99.59149643 | 24.07511602 | 19.67689881 | -6.521427042 | -18.80153028 | 16.17067087 | -25.04258422 | -25.58798706 |
| 80 | -1.563382282 | -8.212314483 | 2.125853175 | 114.7106571 | 233.7062462 | 290.3833115 | 12.52944464 | 0.643128595 | -11.70731147 | -19.95915781 | -7.779222026 | 21.51152138 | 45.37834284 | 100.7419716 | 23.37059322 | 20.628487 | -2.923903188 | -18.32745539 | 18.31752469 | -23.47963423 | -25.53512225 |
| 82 | -5.892921088 | -7.619357829 | 0.831448413 | 115.3583786 | 218.428963 | 305.2661721 | 11.86045244 | 1.117798186 | -12.33077836 | -21.53445054 | -8.869802679 | 21.37537748 | 47.04872835 | 99.99597942 | 24.34524494 | 20.96243377 | -3.451676829 | -18.60323199 | 18.69373776 | -25.3567672 | -24.93718322 |
| 84 | -1.503246967 | -7.856527304 | 1.49281746 | 114.818884 | 218.1093234 | 310.3605353 | 11.85317952 | 0.073366881 | -13.14734742 | -22.08066234 | -12.22896412 | 20.89190547 | 46.94695467 | 100.072989 | 26.44739956 | 20.77675127 | -3.292034674 | -19.41135351 | 19.91315732 | -24.85920236 | -25.51146398 |
| 86 | -2.285006058 | -2.668271973 | 1.549513889 | 111.7360576 | 210.7302854 | 310.5896138 | 12.13318627 | -0.7191063 | -13.07932687 | -22.68999363 | -15.14104057 | 19.75070605 | 47.46638976 | 99.72292719 | 27.87660655 | 21.16861788 | -3.706949879 | -20.44115024 | 20.0335989 | -25.17082161 | -25.91601515 |
| 88 | -3.457542769 | -4.061683844 | -0.633025794 | 103.5682075 | 212.1661795 | 302.4612772 | 12.16418816 | -0.828310123 | -12.77422306 | -25.43911709 | -16.78950591 | 20.02716208 | 49.34222915 | 98.62279597 | 29.74720343 | 21.4030749 | -2.462827688 | -20.16971379 | 18.09308001 | -24.09135883 | -25.5813946 |
| 90 | -2.946426569 | -0.355787179 | 1.048759921 | 116.0618533 | 204.7068176 | 296.7376064 | 12.75182376 | -2.133555245 | -12.65684669 | -24.39802151 | -17.20820392 | 19.92536143 | 49.16573362 | 96.40661577 | 30.55036457 | 21.61757939 | -0.566717693 | -21.08183931 | 12.70902713 | -23.07462748 | -25.3642609 |
| 92 | -8.208028778 | -5.099308536 | 0.377926587 | 115.9536264 | 207.5786057 | 296.6230672 | 12.85029163 | -3.722459382 | -13.3420627 | -24.80927291 | -17.47132971 | 18.745153 | 51.08854054 | 95.72787186 | 32.85269836 | 21.44345607 | -1.046390409 | -24.27940229 | 12.31522909 | -24.70378623 | -23.96572778 |
| 94 | -7.666810946 | -6.225945958 | 1.502261905 | 115.3583786 | 207.2597941 | 292.4443596 | 11.93741942 | -4.480383982 | -12.62511172 | -25.98648027 | -17.89101541 | 18.44440494 | 52.99976661 | 93.80309855 | 32.39715225 | 21.96318178 | -0.634566352 | -26.77139542 | 12.21247361 | -23.47787875 | -25.14699185 |
| 96 | -6.103428664 | -7.708304624 | 1.823501984 | 112.7084597 | 196.8094004 | 288.723809 | 14.30735974 | -5.480795996 | -13.04067909 | -28.33493242 | -19.44755496 | 15.31573579 | 53.12077927 | 92.99863233 | 34.48100703 | 22.52024331 | 1.23685259 | -31.17857585 | 10.53365547 | -23.46912779 | -25.05827599 |
| 98 | -6.103428664 | -3.350175421 | -0.434623016 | 114.4942033 | 200.3593876 | 271.6660194 | 14.31228548 | -6.62824924 | -13.34032205 | -28.70343164 | -17.7467023 | 14.84950228 | 57.04150078 | 92.88452151 | 35.36577644 | 23.13584097 | 0.409677225 | -35.29326465 | 8.653273605 | -23.17148467 | -25.48190502 |
| 100 | -8.117859781 | -4.447075998 | 2.711636905 | 104.4340226 | 190.7064367 | 268.4029675 | 14.05750036 | -7.181333998 | -14.76771078 | -27.62110715 | -20.6734399 | 16.57772883 | 58.46977006 | 93.64548672 | 37.2902419 | 23.4954642 | 0.372910403 | -37.36048087 | 5.574155432 | -24.65676107 | -23.66211013 |
| 102 | -10.01202027 | -2.638601063 | 0.519642857 | 112.6002329 | 192.7402059 | 270.6924358 | 14.10015893 | -8.480503919 | -14.86917546 | -28.24335447 | -20.81201664 | 16.07629618 | 62.7409265 | 93.75646231 | 39.33872221 | 24.71701218 | -1.239474451 | -40.35033926 | 4.238974301 | -25.00281224 | -24.19621416 |
| 104 | -6.103428664 | -8.152972663 | 2.258125 | 98.48482364 | 194.136352 | 258.3287803 | 13.80127706 | -10.59921078 | -15.46166019 | -29.97362178 | -23.76140672 | 15.48767767 | 60.79723313 | 92.82613642 | 40.32632545 | 24.10925836 | -1.111934817 | -42.7935319 | 3.701905223 | -24.79070129 | -23.58412127 |
| 106 | -6.313868291 | -6.433444522 | 1.63453373 | 90.48095371 | 197.4470235 | 249.9140956 | 14.87952867 | -11.87833026 | -15.60152583 | -28.8742282 | -24.36943624 | 16.41847181 | 62.57472722 | 92.03230969 | 40.25336583 | 24.73792533 | -1.602457297 | -43.17630268 | 7.499280958 | -26.41802929 | -23.47258636 |
| 108 | -6.283766659 | -6.611338112 | 3.713154762 | 99.94588656 | 191.5038796 | 251.2306386 | 15.18609603 | -12.8289019 | -15.95994927 | -29.66307695 | -24.43350618 | 15.72959301 | 64.86387908 | 94.08923712 | 42.712998 | 24.4548665 | -1.484457773 | -42.5946086 | 12.66333392 | -25.66458421 | -22.435907 |
| 110 | -3.668050345 | -5.57371342 | 2.220327381 | 98.91773117 | 194.4957395 | 241.5000691 | 15.13227579 | -12.78674271 | -16.19044336 | -28.90193338 | -25.56006036 | 15.21880873 | 65.05126914 | 91.67135455 | 41.88408233 | 24.33841989 | -0.48774964 | -40.92679074 | 14.39308905 | -25.05848325 | -21.57568693 |
| 112 | -6.404037288 | -5.277202125 | -0.160625 | 92.96853221 | 192.7402059 | 235.8330097 | 16.88271791 | -12.08233387 | -17.23883889 | -28.7729127 | -27.3297111 | 14.41924365 | 65.68012812 | 92.31952164 | 41.91947664 | 25.52049544 | -0.571414732 | -40.02873602 | 11.22584068 | -25.56662399 | -21.86567065 |
| 114 | -7.005390434 | -2.608996088 | 0.056686508 | 92.10271715 | 186.9966297 | 244.0186159 | 15.87139521 | -11.28852849 | -16.65220477 | -30.75583659 | -28.54485305 | 11.33579012 | 65.91450338 | 91.4024331 | 39.12343728 | 24.6801175 | -1.832738427 | -39.45627605 | 9.6675639 | -26.92985559 | -22.14367035 |
| 116 | -9.170125863 | -5.840487869 | 1.341656746 | 104.1093419 | 189.0709749 | 241.0991818 | 15.76253742 | -11.97651644 | -18.00631118 | -29.79507977 | -29.95395285 | 12.35037672 | 66.33021889 | 92.4001961 | 40.22367344 | 25.43641591 | -0.051182666 | -39.38908247 | 2.684063308 | -26.10187847 | -21.0705555 |
| 118 | -6.193597661 | -7.08567706 | 2.796676587 | 93.56378007 | 179.1787084 | 230.1659502 | 14.35576203 | -13.27518571 | -17.84718091 | -31.30321737 | -31.29597931 | 13.80200003 | 66.96165585 | 93.1648718 | 38.87541026 | 25.45284681 | -1.926988521 | -40.08331547 | 0.821261747 | -26.99333107 | -21.37529491 |

Figure 6d

| RSC96 baseline | RSC96+NMB+Ca^2+^ | RSC96+ NMB+PD168398+Ca^2+^ | RSC96+Nifedipine | RSC96+NP118809 | RSC96+Trimethadione | RSC96+NiCl_2_ |
| --- | --- | --- | --- | --- | --- | --- |
| 3.457610719 | 389.8857059 | 16.88271791 | 61.75394889 | 212.4877524 | 25.52049544 | 20.0335989 |
| 1.89742173 | 528.8793567 | 1.167226589 | 54.86827694 | 209.6790652 | 1.815409602 | 1.18186391 |
| 6.519275794 | 310.5896138 | 0.756479476 | 40.9448813 | 173.9912023 | 2.105409533 | 6.425950295 |

Figure 6e

| time | RSC96+NMB+Ca^2+^ | | | RSC96+NMB+Ca^2++^KT5720 | | |
| --- | --- | --- | --- | --- | --- | --- |
| 0 | 0 | 0 | 0 | 0 | 0 | 0 |
| 2 | -0.432907532 | -2.273912935 | -4.406267699 | 0.227242591 | -1.137277633 | 0.092835115 |
| 4 | 4.325795714 | 2.55297654 | 0.200113272 | 0.883534128 | -0.461992955 | 0.545247352 |
| 6 | 10.97846941 | -2.951283941 | -5.606947329 | 0.262158688 | -0.284953222 | 0.190372294 |
| 8 | 22.55218667 | 1.675209712 | -5.472909194 | 1.63037876 | 2.428488136 | 1.229609464 |
| 10 | 16.87355493 | 0.239315673 | -1.868982443 | 2.173912335 | 1.606912764 | 1.852034468 |
| 12 | 16.0618533 | 0.478631346 | -15.62016236 | 2.14395687 | 1.292049011 | 2.565134025 |
| 14 | 11.68194415 | -0.478631346 | -49.73192373 | 2.488819013 | -4.75400381 | 2.485943173 |
| 16 | 33.31420232 | 3.549987165 | -8.144232584 | 3.079546106 | 0.783900948 | 2.384759433 |
| 18 | 55.54334815 | 3.589735097 | 23.4321314 | 2.574413459 | 1.717399181 | 2.101080518 |
| 20 | 291.2910155 | 1.714957644 | 198.1329054 | 0.358125 | 9.24581027 | 2.934637255 |
| 22 | 384.6383418 | 33.54642641 | 208.2121956 | 5.451547272 | 5.295316815 | 4.919739876 |
| 24 | 389.8857059 | 188.8316592 | 188.7861053 | 7.029978034 | -1.987055444 | 11.72495279 |
| 26 | 385.0171359 | 418.4687109 | 180.5097225 | 9.494817666 | -5.284338848 | 14.53865486 |
| 28 | 384.6924553 | 528.8793567 | 170.896734 | 11.72487977 | -9.073489747 | 16.85808431 |
| 30 | 357.3815654 | 516.7935012 | 147.6647159 | 12.84530233 | -10.40788284 | 17.61987615 |
| 32 | 341.9674335 | 508.2170568 | 129.7734567 | 13.86738857 | -11.10673961 | 18.32799357 |
| 34 | 342.9955889 | 487.3957652 | 112.6845384 | 14.50713504 | -10.60743675 | 19.09733343 |
| 36 | 337.4792975 | 460.31169 | 104.2061544 | 14.85815167 | -9.344336445 | 19.81520148 |
| 38 | 323.5245888 | 441.205356 | 94.72720408 | 15.93722876 | -6.990717416 | 20.25498796 |
| 40 | 325.2021055 | 425.2101258 | 87.18331131 | 16.16989801 | -7.108415092 | 20.59566901 |
| 42 | 308.1662103 | 404.9461333 | 85.11421559 | 16.40810784 | -5.570719125 | 22.52074627 |
| 44 | 299.8360199 | 380.893666 | 81.37625071 | 17.34490992 | -5.621125714 | 23.87687599 |
| 46 | 286.8028795 | 362.9839104 | 84.78006419 | 17.74961218 | -4.445356132 | 24.5738406 |
| 48 | 259.9773707 | 352.2942009 | 84.24579951 | 17.67104857 | -2.88295489 | 24.51024689 |
| 50 | 244.5632389 | 336.9771698 | 78.37266377 | 18.05243788 | -2.274118274 | 26.20042204 |
| 52 | 223.201548 | 323.1357806 | 76.83594487 | 16.98545824 | -1.689699124 | 26.61142713 |
| 54 | 210.437335 | 302.1944171 | 73.09797999 | 16.31198141 | -0.375188005 | 26.77425525 |
| 56 | 191.8862634 | 292.0222588 | 83.9796111 | 17.19864287 | 0.098541321 | 27.74324168 |
| 58 | 180.8536805 | 276.5056599 | 72.16348877 | 16.12792357 | 0.965789495 | 27.94978736 |
| 60 | 174.6339144 | 277.6227424 | 65.75608835 | 16.83993868 | 1.240117729 | 27.7357774 |
| 62 | 158.4638342 | 260.1121223 | 61.61600906 | 16.36145261 | 1.352209123 | 29.1005121 |
| 64 | 150.0270567 | 258.6356522 | 59.21276194 | 16.52160934 | 2.098229805 | 29.12554186 |
| 66 | 142.3462276 | 260.7099974 | 54.4062677 | 16.85747709 | 2.089003804 | 29.97164625 |
| 68 | 136.4511421 | 247.1079239 | 48.5992071 | 16.44441279 | 2.341734 | 29.32363447 |
| 70 | 127.2567765 | 244.7950911 | 44.79327921 | 16.80514577 | 3.317838109 | 30.4736969 |
| 72 | 121.9569388 | 240.2472653 | 41.65565414 | 16.24018045 | 4.123577922 | 30.92571108 |
| 74 | 140.1833298 | 230.4750706 | 40.58901265 | 16.11527223 | 3.776469503 | 32.01341213 |
| 76 | 124.769198 | 235.5006997 | 28.50481405 | 16.12890763 | 5.485502437 | 32.10774974 |
| 78 | 117.5770297 | 224.4118548 | 27.7704361 | 16.41935667 | 5.333810096 | 32.85085331 |
| 80 | 114.7106571 | 233.7062462 | 18.09137247 | 14.99181583 | 5.378134017 | 33.00780176 |
| 82 | 115.3583786 | 218.428963 | 7.143666226 | 15.35925023 | 5.796253726 | 33.32013516 |
| 84 | 114.818884 | 218.1093234 | 2.737398527 | 15.60931847 | 5.630704401 | 33.45719683 |
| 86 | 111.7360576 | 210.7302854 | -5.138757787 | 14.6723775 | 6.072044086 | 34.26784057 |
| 88 | 103.5682075 | 212.1661795 | -3.403813479 | 14.0841115 | 6.372580257 | 33.98637231 |
| 90 | 116.0618533 | 204.7068176 | -12.61657542 | 13.17849685 | 6.627679892 | 34.84080757 |
| 92 | 115.9536264 | 207.5786057 | -16.48857844 | 12.47577401 | 7.622649565 | 34.89793826 |
| 94 | 115.3583786 | 207.2597941 | -18.02340948 | 13.31041319 | 7.123014474 | 35.3996918 |
| 96 | 112.7084597 | 196.8094004 | -21.36114782 | 12.68379266 | 6.776985506 | 34.91314248 |
| 98 | 114.4942033 | 200.3593876 | -23.43024353 | 12.22161938 | 7.15475094 | 34.63199471 |
| 100 | 104.4340226 | 190.7064367 | -21.42722296 | 11.66550782 | 7.204824161 | 33.98800733 |
| 102 | 112.6002329 | 192.7402059 | -22.62979045 | 11.29778873 | 7.313856091 | 34.15481284 |
| 104 | 98.48482364 | 194.136352 | -17.88937134 | 10.29241632 | 7.262175892 | 34.41828819 |
| 106 | 90.48095371 | 197.4470235 | -20.36058146 | 10.45630442 | 7.752907476 | 34.15437156 |
| 108 | 99.94588656 | 191.5038796 | -22.16160091 | 10.59019927 | 7.283762138 | 34.8907345 |
| 110 | 98.91773117 | 194.4957395 | -23.89843308 | 11.679568 | 7.192465221 | 35.82976354 |
| 112 | 92.96853221 | 192.7402059 | -23.23013026 | 11.39258839 | 7.184179032 | 35.50779799 |
| 114 | 92.10271715 | 186.9966297 | -20.96092128 | 11.49323735 | 6.394862523 | 37.0621156 |
| 116 | 104.1093419 | 189.0709749 | -24.76684916 | 11.38599758 | 6.321260399 | 37.37247061 |
| 118 | 93.56378007 | 179.1787084 | -20.82688314 | 11.23425801 | 6.462476509 | 37.94694638 |

Figure 6f

| RSC96+NMB+Ca^2+^ | RSC96+NMB+Ca^2++^KT5720 |
| --- | --- |
| 389.8857059 | 9.24581027 |
| 528.8793567 | 37.94694638 |
| 208.2121956 | 18.05243788 |

Figure 6g

| RSC96 Ctrl | RSC96+NMB | RSC96+PD168368 | RSC96+NMB+PD168368 |
| --- | --- | --- | --- |
| 8.606819 | 38.962618 | 8.045258 | 12.705854 |
| 7.223523 | 43.218691 | 6.073254 | 9.890763 |
| 9.736193 | 19.11233 | 5.85451 | 12.255168 |
| 9.165125 | 36.916182 | 9.804665 | 14.583765 |
| 9.043739 | 27.840938 | 7.795057 | 11.112498 |

Figure 6h

| RSC96 Ctrl | RSC96+HeLa | RSC96+HeLa+PD168368 | RSC96+ME180 | RSC96+ME180+PD168368 |
| --- | --- | --- | --- | --- |
| 6.940155 | 20.728138 | 11.801519 | 16.090261 | 11.631499 |
| 8.178352 | 12.285174 | 8.893837 | 15.399388 | 8.329631 |
| 8.963238 | 11.945548 | 7.128024 | 18.455356 | 6.152965 |
| 9.966607 | 20.360171 | 4.568473 | 13.429947 | 4.617536 |
| 8.00253 | 12.522003 | 7.068022 | 13.754546 | 8.08525 |

Figure 6i

pCREB

Tubulin

Figure 7a

DRG +HeLa sgNC

DRG +HeLa sgNC+PD168368

DRG +HeLa sgNMB

DRG +HeLa sgNMB+PD168368

DRG +ME180 sgNC

DRG + ME180 sgNC+PD168368

DRG + ME180 sgNMB

DRG + ME180 sgNMB+PD168368

Figure 7b

DRG +HeLa sgNC

DRG +HeLa sgNC+PD168368

DRG +HeLa sgNMB

DRG +HeLa sgNMB+PD168368

DRG +ME180 sgNC

DRG + ME180 sgNC+PD168368

DRG + ME180 sgNMB

DRG + ME180 sgNMB+PD168368

Figure 7c

HeLa

| sgNC | sgNC+PD168368 | sgNMB | sgNMB+PD168368 |
| --- | --- | --- | --- |
| 587.8921069 | 240.284134 | 358.7497882 | 204.4354141 |
| 544.9685616 | 271.3465186 | 332.6410148 | 181.8783188 |
| 549.4454347 | 274.7894659 | 349.917298 | 187.5323696 |

ME180

| sgNC | sgNC+PD168368 | sgNMB | sgNMB+PD168368 |
| --- | --- | --- | --- |
| 489.618419 | 267.4897873 | 293.6928305 | 224.3716241 |
| 463.4493071 | 271.5023131 | 291.8444904 | 190.6069435 |
| 463.9476187 | 271.9718661 | 299.8581724 | 195.1921519 |

Figure 7d

DRG Ctrl

DRG+NMB

DRG+PD168368

DRG+NMB+PD168368

Figure 7e

DRG +SiHa pNC

DRG +SiHa pNMB

DRG +CaSki pNC

DRG + CaSki pNMB

Figure 7f

|  | *CCL2* Ct value | *18S* Ct value | 2^-ΔΔCt^ |
| --- | --- | --- | --- |
| RSC96 Ctrl | 21.322 | 8.335653146 | 1.25799553 |
| RSC96 Ctrl | 21.336 | 8.033972263 | 1.010859624 |
| RSC96 Ctrl | 25.900 | 12.23585415 | 0.78637564 |
|  |  |  |  |
| RSC96 +HeLa | 16.963 | 7.066020807 | 10.70958974 |
| RSC96 +HeLa | 18.708 | 9.023713748 | 12.40962822 |
| RSC96 +HeLa | 20.209 | 10.83108393 | 15.33822889 |
|  |  |  |  |
| RSC96 +ME180 | 15.905 | 6.974963347 | 20.93382335 |
| RSC96 +ME180 | 19.317 | 10.03351879 | 16.38411191 |
| RSC96 +ME180 | 15.887 | 6.974963347 | 21.19611988 |

Figure 7g

|  | *CCL2* Ct value | *18S* Ct value | 2^-ΔΔCt^ |
| --- | --- | --- | --- |
| RSC96 Ctrl | 21.6882356 | 7.617802461 | 1 |
| RSC96 Ctrl | 22.72615369 | 8.256453832 | 0.758243579 |
| RSC96 Ctrl | 23.122 | 9.504295349 | 1.368919622 |
|  |  |  |  |
| RSC96 +0.01μM NMB | 22.55117098 | 9.623436292 | 2.207936142 |
| RSC96 +0.01μM NMB | 21.24133237 | 8.049489975 | 1.838578459 |
| RSC96 +0.01μM NMB | 21.847 | 8.506700516 | 1.658505356 |
|  |  |  |  |
| RSC96 +0.25μM NMB | 20.5485611 | 8.822463671 | 5.078265118 |
| RSC96 +0.25μM NMB | 20.71183205 | 8.709063848 | 4.1920762 |
| RSC96 +0.25μM NMB | 21.545 | 9.616583824 | 4.413185626 |
|  |  |  |  |
| RSC96 +1μM NMB | 20.63128153 | 9.26765728 | 6.528759442 |
| RSC96 +1μM NMB | 20.44732348 | 8.96778965 | 6.024741361 |
| RSC96 +1μM NMB | 21.507 | 10.14922047 | 6.553330528 |

Figure 7h

|  | *CCL2* Ct value | *18S* Ct value | 2^-ΔΔCt^ |
| --- | --- | --- | --- |
| RSC96 Ctrl | 22.66489538 | 7.617802461 | 0.430905419 |
| RSC96 Ctrl | 21.88202604 | 8.049489975 | 1 |
| RSC96 Ctrl | 22.296 | 8.506700516 | 1.030604451 |
|  |  |  |  |
| RSC96 + NMB | 21.1869119 | 8.822463671 | 2.766549682 |
| RSC96 + NMB | 21.40513547 | 8.709063848 | 2.198416042 |
| RSC96 + NMB | 21.599 | 9.616583824 | 3.605094155 |
|  |  |  |  |
| RSC96 + PD168368 | 23.3022213 | 8.155412356 | 0.402128117 |
| RSC96 + PD168368 | 22.86954371 | 8.075476011 | 0.513511454 |
| RSC96 + PD168368 | 25.001 | 9.851142883 | 0.401297045 |
|  |  |  |  |
| RSC96 + NMB+PD168368 | 23.65464401 | 8.512650172 | 0.403472492 |
| RSC96 + NMB+PD168368 | 21.88225301 | 7.981579622 | 0.953868749 |
| RSC96 + NMB+PD168368 | 24.542 | 10.22934055 | 0.716970426 |

Figure 7i

|  | *CCL2* Ct value | *18S* Ct value | 2^-ΔΔCt^ |
| --- | --- | --- | --- |
| RSC96 Ctrl | 22.06624158 | 8.956150055 | 1 |
| RSC96 Ctrl | 21.39028041 | 7.914620717 | 0.776163147 |
| RSC96 Ctrl | 22.33314196 | 8.956150055 | 0.831103248 |
|  |  |  |  |
| RSC96 +HeLa sgNC | 18.81034787 | 9.114169439 | 10.65835651 |
| RSC96 +HeLa sgNC | 18.25934029 | 7.879453182 | 6.635496519 |
| RSC96 +HeLa sgNC | 18.85432243 | 9.114169439 | 10.33838216 |
|  |  |  |  |
| RSC96 +HeLa sgNMB | 20.5293026 | 9.09617424 | 3.197541692 |
| RSC96 +HeLa sgNMB | 19.57738686 | 7.683895588 | 2.323984198 |
| RSC96 +HeLa sgNMB | 20.71467527 | 9.09617424 | 2.811988121 |
|  |  |  |  |
| RSC96 +ME180 sgNC | 19.10357221 | 9.587158839 | 12.07271421 |
| RSC96 +ME180 sgNC | 18.15761757 | 7.644901911 | 6.05184849 |
| RSC96 +ME180 sgNC | 19.52702395 | 9.587158839 | 9.001880512 |
|  |  |  |  |
| RSC96 +ME180 sgNMB | 21.42417526 | 9.821755091 | 2.843507001 |
| RSC96 +ME180 sgNMB | 20.22100957 | 7.606717428 | 1.410101862 |
| RSC96 +ME180 sgNMB | 21.66069285 | 9.821755091 | 2.413545071 |

Figure 7j

Serum level of CCL2 (pg./ mL)

| Normal  (n=8) | Non-PNI  (n=18) | PNI  (n=20) |
| --- | --- | --- |
| 4.705882 | 19.11765 | 390 |
| 16.17647059 | 9.117647 | 203.2352941 |
| 21.17647059 | 10.29412 | 137.9412 |
| 4.117647059 | 10.88235 | 13.52941 |
| 56.17647059 | 6.470588 | 7.058824 |
| 11.47059 | 42.35294 | 371.1765 |
| 44.70588235 | 42.64706 | 14.70588 |
| 20.58823529 | 21.76471 | 137.9412 |
|  | 21.17647 | 11.47059 |
|  | 5.882353 | 77.64706 |
|  | 10.29411765 | 10.58824 |
|  | 29.70588235 | 62.35294 |
|  | 42.05882 | 218.8235 |
|  | 93.82352941 | 42.05882 |
|  | 77.64706 | 178.5294 |
|  | 22.35294 | 14.41176 |
|  | 5.588235 | 136.4706 |
|  | 13.82353 | 431.1765 |
|  |  | 107.6470588 |
|  |  | 136.4705882 |

Figure 7l

Combination CCL2 with NMB

| Non-PNI | PNI |
| --- | --- |
| 0.20502 | 0.99998 |
| 0.13836 | 0.99954 |
| 0.11502 | 0.8885 |
| 0.10859 | 0.93954 |
| 0.0407 | 0.26748 |
| 0.37088 | 0.99957 |
| 0.28002 | 0.20631 |
| 0.17832 | 0.86187 |
| 0.14193 | 0.53197 |
| 0.04239 | 0.82763 |
| 0.05818 | 0.18366 |
| 0.64287 | 0.52302 |
| 0.0754 | 0.98904 |
| 0.66118 | 0.50878 |
| 0.16034 | 0.9465 |
| 0.8255 | 0.94626 |
| 0.29653 | 0.87288 |
| 0.18777 | 0.9997 |
|  | 0.97924 |
|  | 0.99952 |

Figure S11a

NMB

GAPDH

Figure S11b

NMB

GAPDH
