# Supplementary figures and images for: Cervical cancer-produced neuromedin-B reprograms Schwann cells to initiate perineural invasion
Source: Cell Death Dis. 2024 Aug 30;15(8):636. doi: 10.1038/s41419-024-07030-9 (PMC11364772; doi:10.1038/s41419-024-07030-9)

**Fig 6i**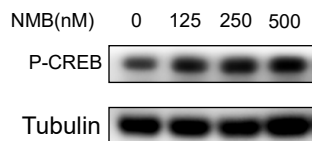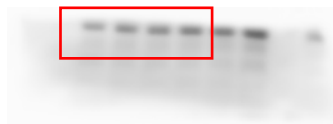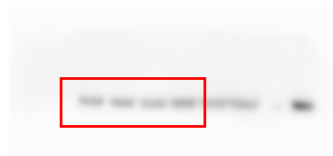**Fig S2c**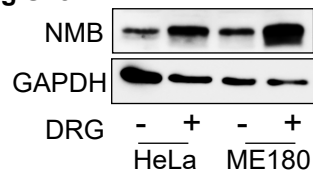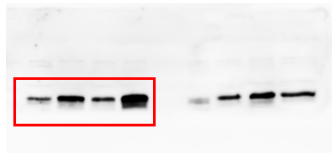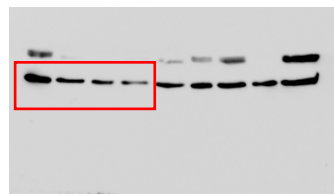**Fig S3a**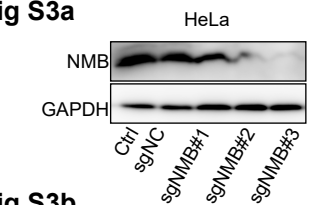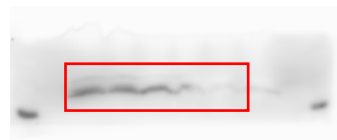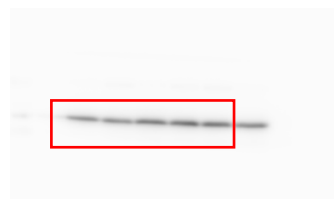**Fig S3b**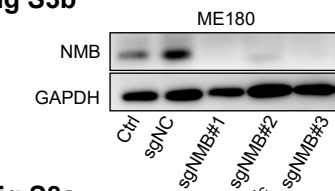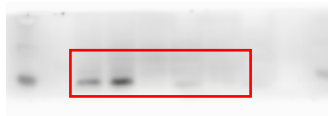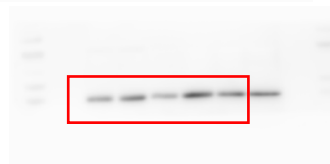**Fig S8a**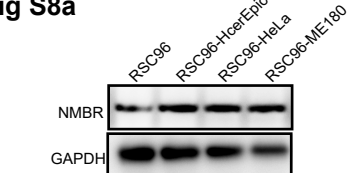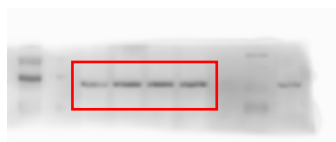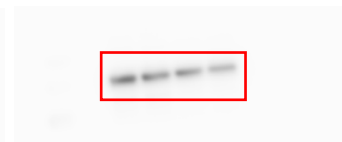**Fig S8b**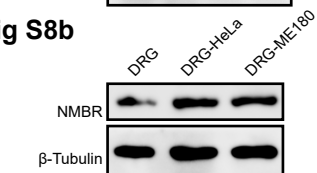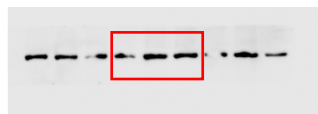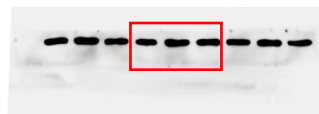**Fig S11a**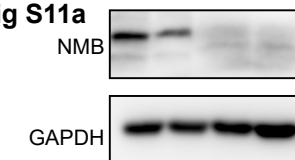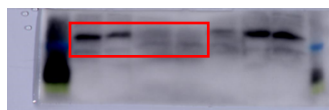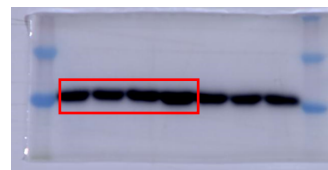**Fig S11b**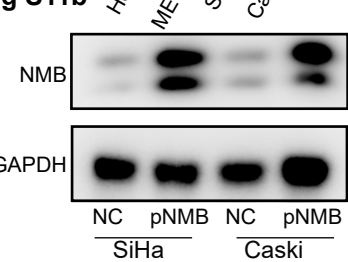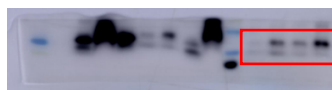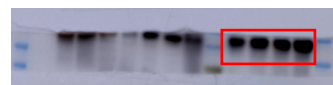

Supplement: Supplementary file 3 — Full and uncropped western blots [file 41419_2024_7030_MOESM3_ESM.pdf]
